# Nitrosoarenes as Versatile Precursors for ^18^F‑Fluorination

**DOI:** 10.1021/acsomega.6c02349

**Published:** 2026-05-26

**Authors:** Markus Laube, Silvia Roscales, Jens Pietzsch, Aurelio G. Csáky

**Affiliations:** † Institute of Radiopharmaceutical Cancer Research, 28414 Helmholtz-Zentrum Dresden-Rossendorf, Dresden 01328, Germany; ‡ Instituto Pluridisciplinar, 16734 Universidad Complutense de Madrid, Madrid 28040, Spain; § Faculty of Chemistry and Food Chemistry, School of Science, Technische Universität Dresden, Dresden 01062, Germany

## Abstract

Fluorine-18 is a central radionuclide for positron-emission tomography. The [^18^F]­fluoro-for-nitroso exchange has not yet been utilized for radiofluorination. Herein, we report the first insights into this transformation and its substrate scope based on the evaluation of 25 model compounds. Since nitrosoarenes are readily obtained from anilines or nitroarenes, this approach creates a direct link between these common functional groups and ^18^F labeling and now allows versatile access to labeling precursors from aromatic amines.

## Graphical abstract

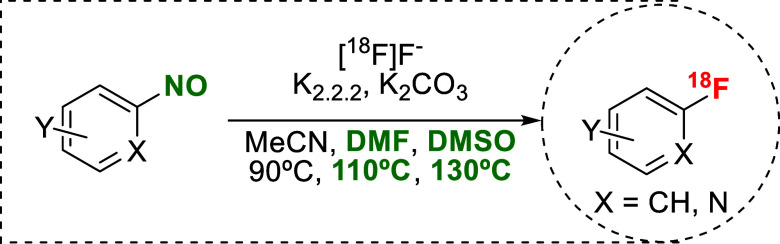

## Introduction

Positron emission tomography (PET) represents a powerful noninvasive diagnostic tool that is today widely used in oncology for tumor staging and response evaluation.^1^ Fluorine-18 represents a widely used radionuclide in PET.^2–4^ The physical properties of the nuclide are ideal. The half-life of 109.7 min and the possibility of producing high activity amounts, e.g., up to the terabecquerel level with commercially available cyclotron devices, allow for the production and delivery of radiopharmaceuticals in batches for more than 100 patients at a time and delivery in a relatively large area around the production site. The low energy (0.635 MeV) of the emitted β^+^-particles further sets the basis for high image resolution compared to other PET nuclides like gallium-68 or carbon-11. From a medicinal chemistry perspective, the introduction of fluorine into a molecule is a widely used strategy for drug development.^5,6^ It is based on the fact that metabolic stabilization of the molecule can be achieved, and the exchange of, e.g., hydrogen atoms or hydroxyl groups with fluorine is often well tolerated in terms of binding affinity to the target enzyme or receptor. Hence, a variety of fluorinated compounds have been developed so far and tested for their biological properties. However, the radiolabeling of molecules with fluorine-18 still represents a challenge based on the fact that radiofluorination of the molecule must be achieved at a late stage during the synthetic process. Today, for this purpose, several radiofluorination techniques^2–4,7^ have been developed for aliphatic or aromatic organic compounds as well as for molecules bearing chelators that were initially developed for radiometal complexation.

Within this portfolio of techniques, radiolabeling strategies for accessing ^18^F-labeled aromatic compounds have gained major importance because of the wide occurrence of fluoroarenes in pharmacologically active molecules.^2,5^ Nucleophilic aromatic substitution reactions (S_N_Ar) are among the most widely used reaction classes, with a plethora of described methods in the literature for their use, each having a definite reaction scope.^8–10^ As leaving groups for the nucleophilic displacement (Scheme 1), most often trimethylammonium-, diazo-, or nitro-groups have been employed.^2,3,11^ More recent developments include radiofluorination of *N*-aryl sydnones,^7^ pyridinium salts,^12^ triarylsulfonium salts,^13,14^ diaryliodononium salts,^15,16^ spirocyclic hypervalent iodine­(III)-ylides,^17^ or oxidized iodarenes^18^ as well as ruthenium-mediated deoxyfluorination of phenols.^19^

**Scheme 1:**
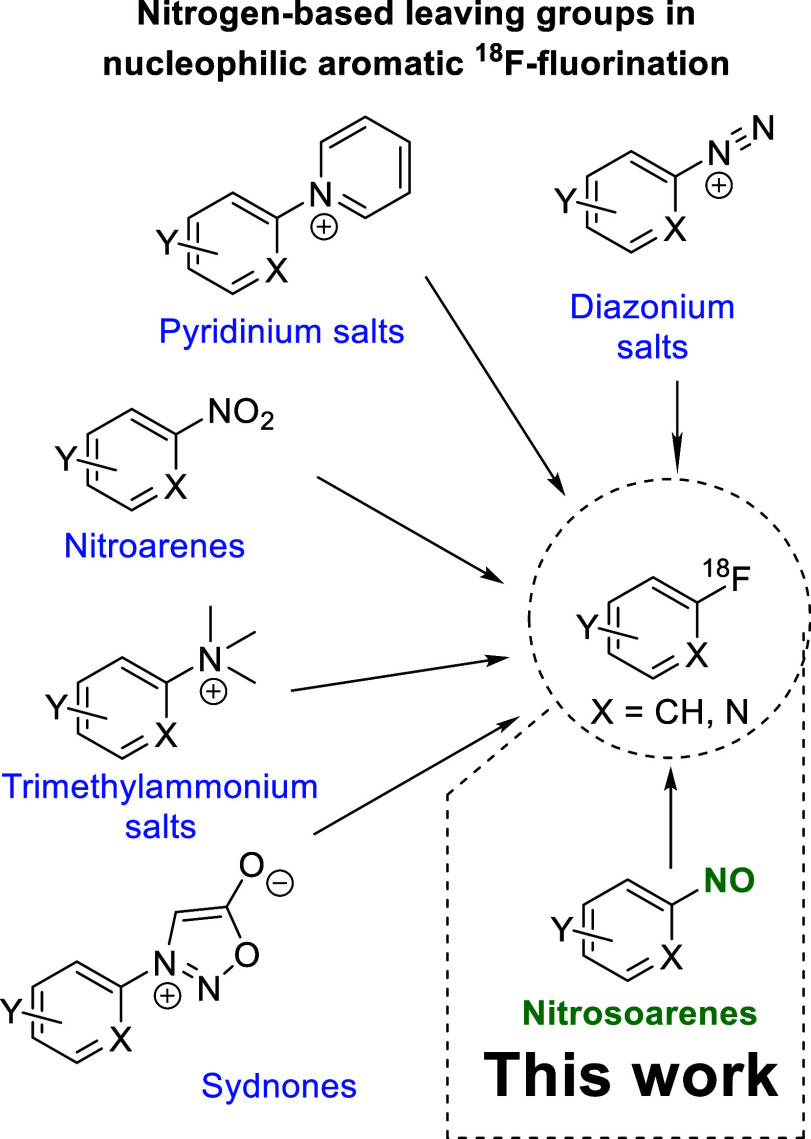
Overview about Nitrogen-Based Leaving Groups Used for ^18^F-Fluorinations and Aim of This Work

Nitro or trimethylammonium groups can be regarded as standard and widely used leaving groups to gain access to ^18^F-labeled arenes. Interestingly, nitrosoarenes that only have one oxygen less compared to the nitroarenes have not yet been explored for ^18^F-chemistry.

Nitroso compounds are well recognized as highly versatile synthetic intermediates, capable of functioning as both electrophiles and nucleophiles.^20–22^ Depending on the reaction conditions, they can undergo either oxidative or reductive transformations to yield a variety of products, including amines, hydroxylamines, azo compounds, and nitro derivatives.^23^ Furthermore, in the presence of suitable catalysts, nitrosoarenes participate in rearrangement and coordination processes. They are also extensively employed in cyclization reactions aimed at the synthesis of structurally diverse and valuable molecular scaffolds.^24^ Nitroso species may also act as biradicals, participating in radical-mediated processes.^25^ However, the pronounced reactivity of nitrosoarenes presents significant challenges for fully elucidating their mechanistic pathways and optimizing reaction parameters. Over the past decade, advances in computational chemistry have provided profound theoretical insights into the complex mechanisms underlying nitrosoarene reactivity, substantially contributing to the progress of this field.^26^

The ability of the nitroso group to assume different conjugation states with an aromatic ring can influence the electron deficiency of the ring. With regard to S_N_Ar reactions, due to π-electron delocalization, protonated nitrosoarenes can give rise to stable cations. Consequently, the nucleophilic reaction site undergoes a transfer from the nitroso group to the aryl group. Guided by this principle, diarylamines have been synthesized by reactions between anilines and protonated *p*-nitrosophenyl ethers.^27,28^ Additionally, nitrosobenzenes have been transformed into *N*-(tert-alkyl)-*ortho*-nitrosoanilines by oxidative S_N_Ar of hydrogen with amines.^29^ On the other hand, S_N_Ar of halogens in *N*-aryl-2-nitrosoanilines with nitrogen nucleophiles and alkoxide ions proceeds highly regioselectively in the position *para* to the nitroso group.^30,31^ In these types of reactions, the nitroso group acts as an activating group.

On the other hand, many nitrosoarenes display promising biological activities^32,33^ and, together with their diverse synthetic applications,^34–36^ highlight their relevance in organic synthesis. This is evidenced by their use as key intermediates in various total syntheses^37–39^ and in the development of nitrosation methodologies applied to natural products.^40–43^ In this context, a variety of synthetic strategies have been developed to access nitroso­(hetero)­arenes under mild conditions, enabling their preparation even in complex molecular settings while tolerating a broad functional group tolerance, including halogens, nitro, carbonyls, acids and their derivatives, alcohols, and amines.^44^ This functional group compatibility is particularly advantageous for complex molecule synthesis and late-stage functionalization, as it allows the introduction of the nitroso group without significant perturbation of pre-existing functionalities.^24,32,33^

Numerous complementary methodologies have been developed for the synthesis of nitroarenes (Scheme 2),^44^ including direct nitrosation of electron-rich aromatics^45^ or electrophilic nitrosation of aryl metallic compounds,^46^ boronic acids,^47,48^ and phenols.^49^ A common method for synthesizing nitrosoarenes is the reduction of nitro compounds, using approaches that utilize transition metal oxides,^50^ organic catalysts,^51^ electrochemical reduction,^52^ organometallic reagents, and single-electron transfer,^53^ among others. Alternatively, the oxidation of anilines has become a well-established method for generating nitrosoarenes. This approach encompasses a number of different techniques, such as photochemical rearrangements^54,55^ and, most prominently, metal-catalyzed transformations using principally hydrogen peroxide as the oxidant.^56–61^ Additionally, the oxidation of anilines with inexpensive Oxone proved to be an efficient method for the synthesis of nitroso arenes in high yields and purity on a large scale.^62^

**Scheme 2:**
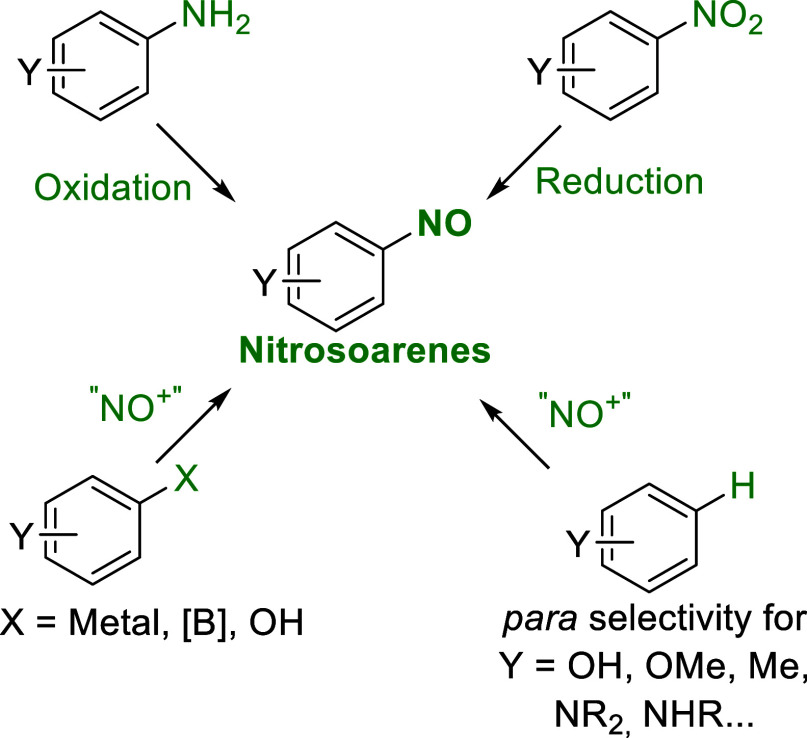
Overview about General Synthetic Approaches toward Nitrosoarenes

On the basis of the rich reactivity of nitroso compounds and their analogy with nitro groups, we decided to explore the use of these compounds in ^18^F chemistry. Herein, we report the first results of [^18^F]­fluoro-for-nitroso exchange as a novel radiofluorination approach.

## Results and Discussion

Our investigation began with the use of 4-chloronitrosobenzene **1a** as radiolabeling precursor (Scheme 3). ^18^F-Fluorination was tested using our recently described microliter-scale radiofluorination approach in HPLC vials^63^ comprising a concentration of K_2_CO_3_ for QMA elution (29.4 mM (normal) vs 7.3 mM (1 base)), reaction temperature (90 °C, 110 °C, 130 °C), and solvent (MeCN, DMF, DMSO) as parameters (for detailed results, see Supporting Information). Using this approach and by testing each resulting condition in a single but standardized experimental setup on the same day, information on optimal conditions for further radiolabeling and indication for maximal radiochemical conversion (RCC) could be derived at minimal time and precursor consumption. Of note, RCC was primarily determined by radio-TLC due to its speed and suitability for routine analysis. Radio-HPLC was additionally used to account for insufficient resolution or potential volatility that may affect TLC-based measurements. In cases where significant discrepancies between methods were observed, radiochemical conversion values obtained by radio-HPLC are reported in Scheme 4, while both data sets are provided in the Supporting Information. Further, product identification was generally performed by chromatographic comparison to the nonradioactive authentic reference expected from fluoro-for-nitroso exchange unless otherwise stated.

**Scheme 3:**
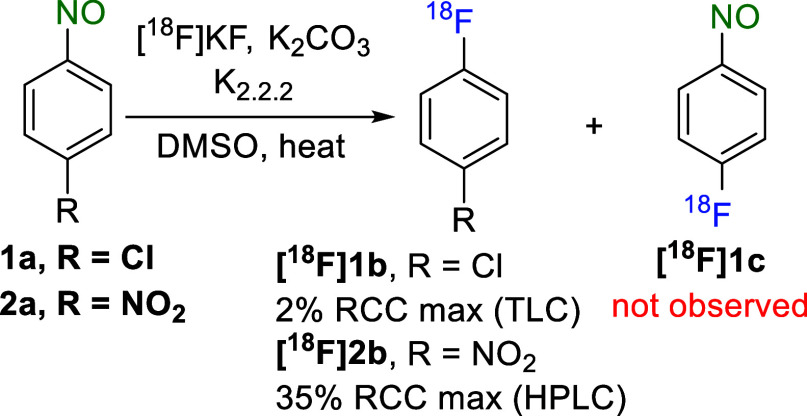
Initial Radiolabeling Attempt and Observed ^18^F-Labeled Products^a^

a RCC max: maximum Radiochemical Conversion (RCC) as determined by Radio-TLC (TLC) or Radio-HPLC (HPLC). Identification was performed by standard addition of the authentic non-radioactive references and analysis by radio-HPLC.

**Scheme 4:**
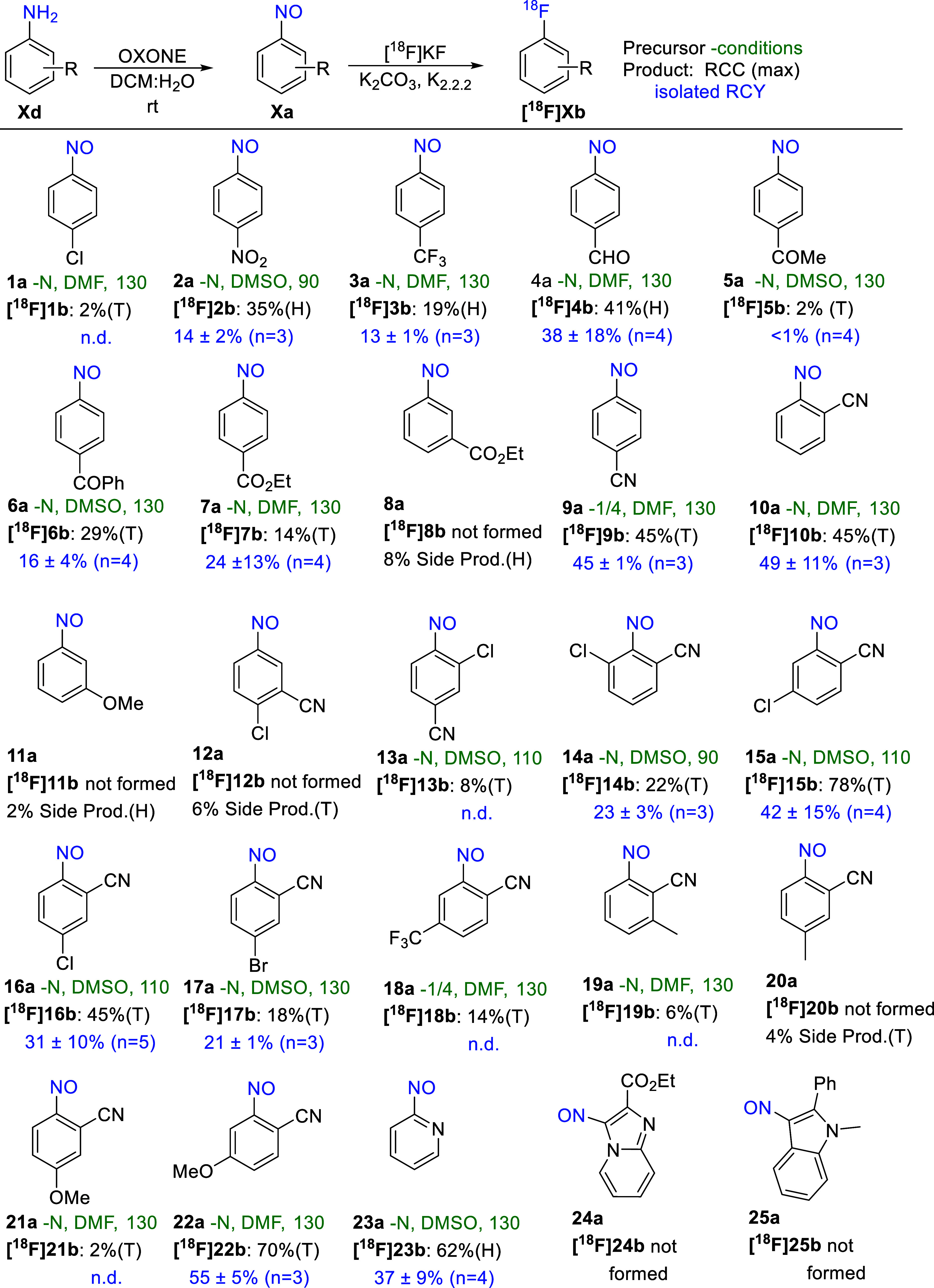
Substrate Scope^a^

a Optimal radiolabeling conditions, radiochemical conversion determined by HPLC (H) or TLC (T) during screening (each condition was tested with *n* = 1), and isolated RCY (mean ± SD) from radiosyntheses (*n* ≥ 3) under optimized conditions are given where appropriate. N: normal base. 1:1/4 base. N.d.: not determined. Side Prod.: side product.

Compound **1a** afforded ^18^F-labeled product **[** ^**18**^ **F]­1b** in 2% radiochemical conversion as analyzed by radio-TLC (RCC, TLC) at maximum that results from [^18^F]­fluoro-for-nitroso exchange. To enhance the electrophilicity of the aromatic ring, 4-nitronitrosobenzene **2a** was synthesized as the NO_2_ substituent withdraws electron density from the ring via inductive and mesomeric effects. Gratifyingly, we identified the desired product **[^18^F]­2b** in 35% (RCC, HPLC) at maximum.

Interestingly, we could not observe any formation of 1-nitroso-4-[^18^F]­fluorobenzene **[** ^**18**^ **F]­1c** from either precursor under these reaction conditions. This result was particularly noteworthy, given that nitro groups are typically used as leaving groups in S_N_Ar fluorinations.^8,10,64^ Halides have also been used as leaving groups for the introduction of [^18^F]­fluoride via halogen exchange but are typically not as effective.^8,10,65^ Furthermore, nitrosoarenes were previously halogenated using CuCl_2_ or CuBr_2_, leading to brominated or chlorinated nitrosoarenes; however, no halogen-for-nitroso exchange was observed or described by the authors.^66^

On the basis of these results, a set of nitroso-substituted compounds was synthesized in yields of 56–89% starting from commercially available amines (for details see Supporting Information). We evaluated 25 compounds in total to gain more insights into the structure–reactivity relationships for the [^18^F]­fluoro-for-nitroso exchange (Scheme 4).

For most of the compounds, all reaction parameters as given above were screened to obtain insights into the optimal reaction conditions. In general, however, application of normal K_2_CO_3_ concentration and higher temperatures of 110 or 130 °C in DMSO or DMF was found to be advantageous for the reaction. Isolated radiochemical yields (RCY) were determined at least in triplicate after semipreparative HPLC using the optimal reaction conditions obtained from screening experiments.

Initially, nitrosoarenes bearing a single electron-withdrawing group at the *ortho*, *meta*, or *para* positions were evaluated. Overall, the presence of such substituents at the *para* position was well tolerated, which is in agreement with the reactivity of [^18^F]­fluoride in S_N_Ar. Compounds substituted with trifluoromethyl (**3a**), formyl (**4a**), phenyl ketone (**6a**), ester (**7a**), or cyano (**9a**) groups enabled the formation of the corresponding [^18^F]­fluorobenzene derivatives with radiochemical conversions ranging between 8 and 45% and isolated RCY between 13 and 45%. Of note, HPLC-DAD-γ-high-resolution mass spectrometry analysis and ^19^F NMR (see discussion in Supporting Information Sections 9 and 10) of selected ^18^F-fluorinations starting from **4a** and **6a** or **9a** without (no carrier added) and with KF addition (carrier added) were performed and formation of the desired fluorinated products was further confirmed besides the chromatographic comparison to the nonradioactive authentic reference used throughout this work. Notably, a direct comparison of conversion obtained by ^19^F NMR and radio-TLC after carrier-added radiofluorination and fluorination at exactly the same substoichiometric fluoride level and reaction times indicates the potential of ^19^F NMR as a suitable substitute or additive for radioactive optimization experiments on this scale, which confirms the common practice^67–69^ using considerably higher fluoride/precursor concentration and/or reaction times in optimization studies. Interestingly, for nitrosobenzene with *para*-methyl ketone **5a**, the radiochemical conversion was very low (2%) and 4-[^18^F]­fluoroacetophenone **[** ^**18**^ **F]­5b** could not be isolated (RCY < 1%) in sufficient amounts for product identification. A cyano group in the *ortho* position (**10a**) gave the expected product with an RCC of 45% and could be isolated in 49 ± 11% RCY, which was comparable to the reactivity of the *para* (**9a**) derivative (45 ± 1% RCY). Conversely, substitution at the *meta* position with either electron-withdrawing CO_2_Et (**8a**) or CN (**12a**) or electron-donating OMe (**11a**) groups did not result in the formation of the desired fluorinated products, which is consistent with the reaction mechanism of S_N_Ar.^8,10^

Encouraged by the results observed for *ortho*-cyano-substituted derivative **10a**, we decided to explore the influence of further substituents at different positions of the phenyl ring, keeping the cyano group. Halogens showing the –I effect were well tolerated when installed in *ortho*- (**13a**, **14a**), *meta*- (**15a**), or *para*- (**16a**, **17a**) to the NO group, giving RCC ranging from 8% for **[** ^**18**^ **F]­13b** and 78% for **[** ^**18**^ **F]­15b**, as well as isolated RCY in the range of 21–42%. While compound with Cl in *para* (**16a**) gave moderately high yields (31 ± 10%), lower reactivity was observed for the respective Br-derivative **17a** with an RCC of 18% and an isolated RCY of 21 ± 1%. Notably, product **[** ^**18**^ **F]­14b**, bearing increased steric hindrance, was obtained with a RCY of 23 ± 3. Compound **18a** having a trifluoromethyl group (-I effect) in the *meta* position to the nitroso group showed medium reactivity with RCC of 14% and was the only compound in this subset where a decrease of base (1/4 base) led to optimal results in radiolabeling. Positioning the methyl group showing a + I effect *meta* to the nitroso leaving group and *ortho* to the cyano group in compound **19a** decreased reactivity to a low RCC of 6%. In line with this, no or only 2% RCC (TLC), respectively, was observed when the electron-donating methyl group (**[** ^**18**^ **F]­20b**) or methoxy group (**[** ^**18**^ **F]­21b**, showing additional +M effect) was positioned *para* to the NO functionality. In contrast, methoxy positioned in *meta* led to a high RCC of 70% and allowed isolation of **[** ^**18**^ **F]­22b** in high RCY 55 ± 5%.

Radiofluorinations were performed using a nonoptimized precursor concentration of 5 mg/mL (approximately 20–45 mM depending on the substrate). To assess the influence of precursor concentration, selected compounds (**6a**, **9a**, and **22a**) were evaluated over a range of 5–40 mM (see Supporting Information, Section 11). These studies indicate that the radiochemical conversion is largely maintained over a broad concentration range for most substrates, while a concentration-dependent decrease was observed in the specific case of **9a** below 20 mM.

This methodology was further extended to heterocycles. The commercially available and electron-poor 2-nitrosopyridine **23a** showed a high RCC of 62%, leading to a 37 ± 9% isolated RCY of **[** ^**18**^ **F]­23b**. Unfortunately, attempts to radiolabel imidazopyridine **24a** or electron-rich indole derivative **25a** (synthesized by direct nitrosation of corresponding heterocycles, see Supporting Information) showed no reactivity in accordance with the S_N_Ar mechanism.

Radiolabeling was performed under optimized conditions using K_2.2.2_/K_2_CO_3_. To evaluate the influence of the base and phase-transfer system, selected substrates (**5a**, **9a**, **22a**, and **25a**) were also tested using tetrabutylammonium tosylate (TBAOTs) and tetraethylammonium bicarbonate (TEAB) (see Supporting Information, Section 12). These experiments indicate that efficient [^18^F]­fluoro-for-nitroso exchange requires basic conditions, with K_2.2.2_/K_2_CO_3_ providing the highest radiochemical conversions among the systems tested.

The methodology currently shows the highest efficiency for electron-deficient aromatic substrates, while electron-rich and some heteroaromatic systems exhibit no reactivity. This defines a limitation of the present approach and indicates that further development is required to broaden the substrate scope. Despite these limitations, the approach provides a practical entry point to selected radiotracer-relevant motifs.

## Conclusions

This study provides the first demonstration of an ^18^F-fluoro-for-nitroso exchange, establishing nitrosoarenes as versatile and previously overlooked precursors in ^18^F chemistry. Because nitrosoarenes are readily obtained from anilines, this strategy effectively translates the ubiquity of this functional group into a promising radiolabeling opportunity. Access to nitrosoarenes by direct nitrosylation or reduction of nitroarenes expands the applicability of this approach. Mechanistic analysis supports a nucleophilic aromatic substitution pathway with the nitroso group acting as a leaving group. The requirement for an *ortho*- or *para*-electron-withdrawing substituent is consistent with this interpretation, while the approach displays notable tolerance toward a broad range of additional substituents. The method extends beyond simple arenes, enabling the efficient radiofluorination of electron-deficient heterocycles. Overall, the nitroso handle expands the synthetic toolbox for ^18^F chemistry, offering a conceptually simple and practical entry point for the development of new radiotracers.

## Supplementary Material



## Notes

Materials and methods, synthesis of nitrosoarenes, copies of ^1^H NMR and ^13^C NMR spectra, results of radiofluorination optimization, copies of radio-HPLC chromatograms, and discussion of radiosyntheses with subsequent HPLC-DAD-γ-high-resolution mass spectrometry and ^19^F NMR analysis (PDF)
