# Supplementary material for: Nitrosoarenes as Versatile Precursors for 18F‑Fluorination
Source: ACS Omega. 2026 May 26;11(22):32905–11. doi: 10.1021/acsomega.6c02349 (PMC13261467; doi:10.1021/acsomega.6c02349)
Supplement: Supplementary file 1 [file ao6c02349_si_001.pdf]

Nitrosoarenes as Versatile Precursors for ^{18}F-Fluorination

Markus Laube[1*‡], Silvia Roscales[2*‡], Jens Pietzsch[1,3], Aurelio G. Csáky[2]

[1]Helmholtz-Zentrum Dresden-Rossendorf, Institute of Radiopharmaceutical Cancer Research, Germany.

[2]Instituto Pluridisciplinar, Universidad Complutense de Madrid, Spain.

[3]Faculty of Chemistry and Food Chemistry, School of Science, Technische Universität Dresden, Germany.

Corresponding Author

* Markus Laube, m.laube@hzdr.de

* Silvia Roscales, silviaroscales@ucm.es

Contents

1. General remarks2

2. General procedure for the synthesis or nitrosoarenes by oxidation of anilines:4

3. Analytical data of isolated nitrosoarenes4

4. Synthesis of nitrosoarenes by direct nitrosation of heterocycles:11

5. Spectra of isolated products13

6. General procedure for the screening of reaction conditions for ^{18}F-labeling:38

7. General procedure for isolation of ^{18}F-labeled products including determination of RCY and product identification38

8. Analytical data of radiosyntheses39

9. Radiosyntheses followed by subsequent HPLC-DAD-γ-HRMS analysis119

10. Radiosynthesis followed by subsequent NMR analysis122

11. Analysis of RCC(TLC) at different precursor concentrations for selected substrates126

12. Analysis of different phase transfer agents for selected substrates128

1. General remarks

Synthesis of nitrosoarenes

All reactions were performed with no exclusion of humidity using commercial-grade solvents. All starting materials were commercially available research-grade chemicals and were used without further purification.

Aluminum-backed plates coated with silica gel 60 F254 were used for thin layer chromatography (TLC), and the spots were detected with UV light (254 and/or 366 nm) and/or vanillin solution. Flash column chromatography was carried out on silica gel 60 Å.

NMR analyses were performed at room temperature on a Bruker Fourier 300 MHz spectrometer in $CDCl_3$. Chemical shifts (δ) are reported in ppm, using the residual solvent peak in $CDCl_3$ (δ_H = 7.26 and δ_C = 77.16 ppm) as internal reference, and coupling constants (*J*) are given in Hertz (Hz). Data are reported as follows: chemical shift, multiplicity (s: singlet, d: doublet, t: triplet, q: quartet, p: quintuplet, sex: sextet, h: septet, m: multiplet, b: broad), coupling constants and integration.

Radiosynthesis

All commercial reagents and solvents were used without further purification. Visualization was carried out using UV (254 nm/366 nm). Analytical radio-HPLC was performed with the following systems: (*System 1*) column Kinetex C-18 (Phenomenex 250 x 4.6 mm, 5 µm, 100 Å, with Security Guard precolumn), Shimadzu Nexera X2 UHPLC system (Kyoto, Japan; degasser DGU-20A_{3R} and DGU-20A_{5R}, pump LC-30AD, autosampler SIL-30AC, column oven CTO-20AC with two column switching valves FCV-14AH, diode array detector SPD-M30A, fluorescence detector RF-20A, γ detector Gabi Star (Raytest, Straubenhardt, Germany), communication bus module CBM-20A), eluent: (A): MeCN, (B): 0.1% trifluoroacetic acid in H_2O; flow rate 0.5 mL/min), gradient 1: $t_{0\,min}$ 5/95 – $t_{3.0\,min}$ 5/95 - $t_{28.0\,min}$ 95/5 – $t_{34.0\,min}$ 95/5 – $t_{35.0\,min}$ 5/95 – $t_{40.0\,min}$ 5/95; gradient 2: $t_{0\,min}$ 25/75 – $t_{3.0\,min}$ 25/75 - $t_{28.0\,min}$ 75/25 - $t_{29.0\,min}$ 95/5 – $t_{34.0\,min}$ 95/5 – $t_{35.0\,min}$ 25/75 – $t_{40.0\,min}$ 25/75; gradient 3: $t_{0\,min}$ 5/95 – $t_{1.0\,min}$ 5/95 - $t_{21.0\,min}$ 95/5 - $t_{23.5\,min}$ 95/5 – $t_{25.0\,min}$ 5/95 – $t_{30.0\,min}$ 5/95. Unless otherwise given, gradient 1 was used for system 1. (*System 2*) column Purospher C18e (Merck, LiChroCART 150 x 3 mm, 5 µm) with guard column, Agilent 1200 HPLC: pump G1311A, auto sampler G1329A, column oven G1316A, degasser G1322A, UV detector G1315D, γ detector Gabi Star (Raytest); *gradient 1: 25→75 – 40 min,* flow rate = 0.75 mL/min, (A) MeCN / (B) H_2O + 0.1% TFA, gradient 1: $t_{0\,min}$ 25/75 – $t_{0.5\,min}$ 25/75 – $t_{25.5\,min}$ 75/25 – $t_{27.0\,min}$ 95/5 – $t_{30.0\,min}$ 95/5 – $t_{32.0\,min}$ 25/75 – $t_{40.0\,min}$ 25/75; *gradient 2: 25→75 – 13 min,* flow rate = 0.75 mL/min, (A) MeCN / (B) H_2O + 0.1% TFA, gradient $t_{0\,min}$ 25/75 – $t_{0.5\,min}$ 25/75 – $t_{5.5\,min}$ 75/25 – $t_{6.0\,min}$ 95/5 – $t_{7.5\,min}$ 95/5 – $t_{8.0\,min}$ 25/75 – $t_{13.0\,min}$ 25/75; *gradient 3: 5→95 – 15 min,* flow rate = 0.75 mL/min, (A) MeCN / (B) H_2O + 0.1% TFA, gradient $t_{0\,min}$ 5/95 – $t_{0.5\,min}$ 5/95 – $t_{7.0\,min}$ 95/25 – $t_{8.5\,min}$ 95/5 – $t_{9.5\,min}$ 5/95 – $t_{15.0\,min}$ 5/95; *gradient 4: 5→95 – 40 min,* flow rate = 0.75 mL/min, (A) MeCN / (B) H_2O + 0.1% TFA, gradient $t_{0\,min}$ 5/95 – $t_{3\,min}$ 5/95 – $t_{28\,min}$ 95/25 – $t_{34.0\,min}$ 95/5 – $t_{35.0\,min}$ 5/95 – $t_{40.0\,min}$ 5/95. Semi-preparative HPLC was performed using the following systems: (*System 3*): column (Nucleodur C18 ec, VP 50/8, Macherey Nagel), Jasco HPLC with communication module LC-NetII/ADC, degasser DG-2080-53, gradient mixer LG-2080-02, pump PU-2080-plus, UV detector UV-2075 plus (detection at 254 nm), γ detector Gabi Star (Raytest, Straubenhardt, Germany), eluent: (A): MeCN, (B): 0.1% trifluoroacetic acid in H_2O; flow rate 5 mL/min), gradient: $t_{0\,min}$ 5/95 – $t_{0.3\,min}$ 5/95 – $t_{14.3\,min}$ 95/5 – $t_{15.3\,min}$ 95/5 – $t_{16.0\,min}$ 5/95 – $t_{20\,min}$ 5/95. The products were monitored at λ = 254 nm and in case of radio-HPLC using additionally the γ detector unless otherwise specified.

Analyses including the HPLC-DAD-γ-High resolution mass spectrometer (HRMS) were obtained on System 4: Agilent 1260 Infinity II HPLC (Santa Clara, California, USA; pump G7104C, autosampler G7129C, column oven G7116A, DAD detector G7117C) coupled to γ detector Gabi Star (Raytest Isotopenmeßgeräte GmbH, Straubenhardt, Germany) followed by accurate mass Revident Q-TOF LC/Q-TOF G6575A using electrospray ionization in positive mode. Chromatographic separations were

performed using Poroshell 120 EC-C18 column (120A, 2.7 µm, 50x3.0 mm) and an eluent consisting of (A): MeCN and (B): 0.1% formic acid in H_2O; flow rate 0.4 mL/min) with the following gradient $t_{0\,min}$ 5/95 – $t_{2.0\,min}$ 5/95 – $t_{22.0\,min}$ 95/5 – $t_{23.0\,min}$ 95/5 – $t_{27.0\,min}$ 5/95 – $t_{30.0\,min}$ 5/95. A reference mass solution containing hexakis(1H,1H,3H-tetrafluoropropoxy)phosphazene, and purine was continuously co-injected via dual AJS ESI source. The system was operated using Agilent Masshunter Workstation 3.6 – LC/MS data aquisition software (Version 12.0) and data evaluation was performed using Agilent Masshunter Workstation 3.6 Qualitative Analysis software (Version 12.0 Update 1).

No-carrier-added aqueous [^{18}F]fluoride was produced in a TR-FLEX 18-30 MeV (ACSI, Richmond/Vancouver, Canada) by irradiation of [^{18}O]H_2O via the $^{18}O(p,n)^{18}F$ nuclear reaction.

Radio-thin layer chromatography (Radio-TLC) was performed on silica gel F-254 aluminum plates (Merck TLC silica gel 60 F_{254}, 1.05554.0001). Visualization was performed using a CR35bio scanner system (Raytest, Straubenhardt, Germany) and analyzed using advanced image data analyzer (AIDA) software (Version 5.1 SP4, Raytest, Straubenhardt, Germany).

Radiochemical conversion (RCC) was primarily determined by radio-TLC due to its rapid analysis time and suitability for routine screening of reaction conditions. This method is well suited for quantification of [^{18}F]fluoride and non-volatile radiolabeled products. For selected substrates, RCC was additionally determined by radio-HPLC for all tested reaction conditions when higher-resolution analysis was necessary or when volatility of radiolabeled products was of relevance. In the latter case, partial loss of volatile products during TLC analysis may lead to an underestimation of RCC. Of note, adsorption effects of [^{18}F]fluoride during HPLC analysis may influence quantification too and lead conversely to overestimation of RCC, so that in these cases both data sets are provided for information.

2. General procedure for the synthesis or nitrosoarenes by oxidation of anilines:

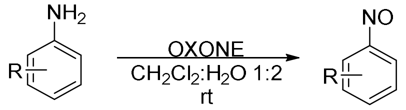

To a stirred solution of the corresponding aniline (2.0 mmol) in CH_2Cl_2 (10 mL) was slowly added a solution of oxone (2.51 g, 4.0 mmol) in H_2O (20 mL). The reaction mixture was stirred at room temperature until TLC analysis indicated complete consumption of the starting material. The aqueous layer was extracted with CH_2Cl_2 (3 x 10 mL). The combined organic layers were successively washed with HCl 1N (20 mL), H_2O (20 mL), $NaHCO_3$ 10% (20 mL) and brine (20 mL). The combined organics were dried over anhydrous $MgSO_4$, filtered, and concentrated *in vacu*o. The crude residue was purified by flash column chromatography on silica gel to afford the nitrosoarenes **1** in 56–89% yield.

The nitrosoarenes described herein are bench-stable and can be handled under standard laboratory conditions. They can be stored at 0 ° C or in a freezer for extended periods without noticeable decomposition. No special precautions are required beyond standard laboratory practice. However, to avoid potential losses of more volatile nitroso compounds (which may exhibit some tendency to sublime depending on the substrate), solvent removal under reduced pressure should be performed without heating the water bath.

3. Analytical data of isolated nitrosoarenes

1-chloro-4-nitrosobenzene (1a)

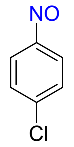

Following the general procedure, starting from 4-chloroaniline (255.1 mg) and stirring the reaction mixture overnight, nitrosobenzene **1a** was obtained as a dark yellow solid after column chromatography using a mixture hexane:CH_2Cl_2 1:1 as eluent (252.0 mg, 89%). ^{1}H NMR (300 MHz, $CDCl_3$): δ 7.86 (d, *J* = 8.6 Hz, 2H), 7.60 (d, *J* = 8.6 Hz, 2H) ppm. ^{13}C-NMR (75 MHz, $CDCl_3$): δ 163.9, 142.6, 129.8, 122.3 ppm. Anal. calcd. for C_6H_4ClNO: C, 50.91; H, 2.85. Found: C, 51.01; H, 2.83. The spectroscopic data are in agreement with reported literature values.[1]

---

[1] Fúster Fernández, I.; Hecquet, L.; Fessner, W.-D. *Adv. Synth. Catal.* **2022**, *364*, 612-621.

1-fluoro-4-nitrosobenzene (1c)

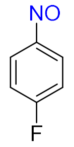

Following the general procedure, starting from 4-fluoroaniline (222.2 mg) and stirring the reaction mixturefor 4 hours, nitrosobenzene **1c** was obtained as a dark yellow oil after column chromatography using a mixture hexane:CH_2Cl_2 1:1 as eluent (200.2 mg, 80%). ^{1}H NMR (300 MHz, $CDCl_3$): δ 7.88-8.00 (m, 2H), 7.23-7.32 (m, 2H) ppm. ^{13}C-NMR (75 MHz, $CDCl_3$): δ 167.0 (d, *J* = 260.0 Hz), 163.7 (d, *J* = 2.4 Hz), 124.1 (d, *J* = 10.3 Hz), 116.5 (d, *J* = 23.4 Hz) ppm. ^{19}F-NMR (282 MHz, $CDCl_3$): δ -100.1 ppm. Anal. calcd. for C_6H_4FNO: C, 57.61; H, 3.22. Found: C, 57.55; H, 3.19. The spectroscopic data are in agreement with reported literature values.[2]

1-nitro-4-nitrosobenzene (2a)

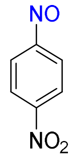

Following the general procedure, starting from 4-nitroaniline (276.3 mg) and stirring the reaction mixture overnight, nitrosobenzene **2a** was obtained as yellow plates after column chromatography using a mixture hexane:CH_2Cl_2 1:1 as eluent (182.5 mg, 60%). ^{1}H NMR (300 MHz, $CDCl_3$): δ 8.51 (d, *J* = 8.6 Hz, 2H), 8.06 (d, *J* = 8.6 Hz, 2H) ppm. ^{13}C-NMR (75 MHz, $CDCl_3$): δ 162.6, 150.5, 125.6, 121.4 ppm. Anal. calcd. for $C_6H_4N_2O_3$: C, 47.38; H, 2.65. Found: C, 47.45; H, 2.63. The spectroscopic data are in agreement with reported literature values.[1]

1-nitroso-4-(trifluoromethyl)benzene (3a)

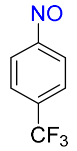

Following the general procedure, starting from 4-(trifluoromethyl)aniline (322.3 mg) and stirring the reaction mixture overnight, nitrosobenzene **3a** was obtained as a orange solid after column chromatography using a mixture hexane:CH_2Cl_2 1:1 as eluent (245.2 mg, 70%). ^{1}H NMR (300 MHz, $CDCl_3$): δ 8.00 (d, *J* = 8.4 Hz, 2H), 7.92 (d, *J* = 8.4 Hz, 2H) ppm. ^{13}C-NMR (75 MHz, $CDCl_3$): δ 164.2, 136.3, 135.4, 127.2, 127.1, 127.1, 127.0, 125.2, 121.6, 121.0 ppm. ^{19}F-NMR (282 MHz, $CDCl_3$): δ -63.1 ppm. Anal. calcd. for $C_7H_4F_3NO$: C, 48.01; H, 2.30. Found: C, 48.09; H, 2.27. The spectroscopic data are in agreement with reported literature values.[1]

---

[2] Kohlmeyer, C.; Klueppel, M.; Hilt, G. *J. Org. Chem*. **2018**, *83*, 3915-3920.

4-nitrosobenzaldehyde (4a)

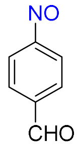

Following the general procedure, starting from 4-aminobenzaldehyde (242.3 mg) and stirring the reaction mixture overnight, nitrosobenzene **4a** was obtained as a light yellow solid after column chromatography using a mixture hexane:CH_2Cl_2 1:1 as eluent (183.8 mg, 68%). ^{1}H NMR (300 MHz, $CDCl_3$): δ 10.2 (s, 1H), 8.17 (d, *J* = 8.4 Hz, 2H), 8.03 (d, *J* = 8.4 Hz, 2H) ppm. ^{13}C-NMR (75 MHz, $CDCl_3$): δ 191.4, 163.9, 139.6, 131.2, 121.2 ppm. Anal. calcd. for $C_7H_5NO_2$: C, 62.22; H, 3.73. Found: C, 62.14; H, 3.76. The spectroscopic data are in agreement with reported literature values.[3]

1-(4-nitrosophenyl)ethan-1-one (5a)

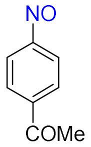

Following the general procedure, starting from 1-(4-aminophenyl)ethan-1-one (270.34 mg) and stirring the reaction mixture for 3 hours, nitrosobenzene **5a** was obtained as a light yellow solid after column chromatography using a mixture hexane:CH_2Cl_2 1:1 as eluent (214.8 mg, 72%). ^{1}H NMR (300 MHz, $CDCl_3$): δ 8.15 (d, *J* = 8.2 Hz, 2H), 7.90 (d, *J* = 8.2 Hz, 2H), 2.64 (s, 3H) ppm. ^{13}C-NMR (75 MHz, $CDCl_3$): δ 197.3, 164.2, 141.1, 129.8, 120.9, 27.3 ppm. Anal. calcd. for $C_8H_7NO_2$: C, 64.42; H, 4.73. Found: C, 64.48; H, 4.70. The spectroscopic data are in agreement with reported literature values.[4]

(4-nitrosophenyl)(phenyl)methanone (6a)

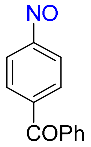

Following the general procedure, starting from (4-aminophenyl)(phenyl)methanone (394.5 mg) and stirring the reaction mixture overnight, nitrosobenzene **6a** was obtained as yellowish crystals after column chromatography using a mixture hexane:CH_2Cl_2 1:1 as eluent (280.1 mg, 71%). ^{1}H NMR (300 MHz, $CDCl_3$): δ 7.99-8.07 (m, 4H), 7.82-7.88 (m, 2H), 7.68 (tt, *J* = 7.5, 2.1 Hz, 1H), 7.55 (tm, *J* = 7.5 Hz, 2H) ppm. ^{13}C-NMR (75 MHz, $CDCl_3$): 160.2, 137.1, 136.9, 131.3, 115.9, 115.3, 113.1 ppm. Anal. calcd. for $C_{13}H_9NO_2$: C, 73.92; H, 4.30. Found: C, 73.60; H, 4.1. The spectroscopic data are in agreement with reported literature values.[5]

[3] Molander, G. A.; Cavalcanti, L. N. *J. Org. Chem*. **2012**, *77*, 4402-4413.
[4] Chavannavar, A. P.; Oliver, A. G.; Ashfeld, B. L. *Chem. Commun.* **2014**, *50*, 10853-10856.
[5]Defoin, A. *Synthesis* **2004**, 706-710.

Ethyl 4-nitrosobenzoate (7a)

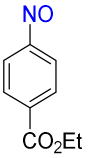

Following the general procedure, starting from ethyl 4-aminobenzoate (330.4 mg) and stirring the reaction mixture for 4 h, nitrosobenzene **7a** was obtained as a yellow solid after column chromatography using a mixture hexane:CH_2Cl_2 1:1 as eluent (211.4 mg, 59%). ^{1}H NMR (300 MHz, $CDCl_3$): δ 8.30 (d, *J* = 8.7 Hz, 2H), 7.93 (d, *J* = 8.7 Hz, 2H), 4.44 (q, *J* = 7.2 Hz, 2H), 1.43 (t, *J* = 7.2 Hz, 3H) ppm. ^{13}C-NMR (75 MHz, $CDCl_3$): δ 165.2, 164.5, 135.6, 131.0, 120.4, 61.8, 14.2 ppm. Anal. calcd. for $C_9H_9NO_3$: C, 60.33; H, 5.06. Found: C, 60.40; H, 5.03. The spectroscopic data are in agreement with reported literature values.[6]

Ethyl 3-nitrosobenzoate (8a)

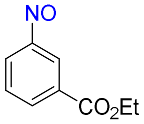

Following the general procedure, starting from ethyl 3-aminobenzoate (330.4 mg) and stirring the reaction mixture overnight, nitrosobenzene **8a** was obtained as a brown solid solid after column chromatography using a mixture hexane:CH_2Cl_2 1:1 as eluent (283.1 mg, 79%). ^{1}H NMR (300 MHz, $CDCl_3$): δ 8.63 (t, *J* = 1.6 Hz, 1H), 8.38 (dt, *J* = 7.8, 1.6 Hz, 1H), 7.98 (dt, *J* = 7.8, 1.6 Hz, 1H), 7.69 (t, *J* = 7.8 Hz, 1H), 4.45 (q, *J* = 7.1 Hz, 2H), 1.44 (t, *J* = 7.1 Hz, 3H) ppm. ^{13}C-NMR (75 MHz, $CDCl_3$): δ 165.3, 165.0, 135.8, 132.2, 129.6, 123.7, 122.6, 61.9, 14.4 ppm. Anal. calcd. for $C_9H_9NO_3$: C, 60.33; H, 5.06. Found: C, 60.40; H, 5.03.

4-nitrosobenzonitrile (9a)

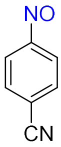

Following the general procedure, starting from 4-aminobenzonitrile (236.3 mg) and stirring the reaction mixture for 1.5 h, nitrosobenzene **9a** was obtained as a greenish-yellow solid after column chromatography using a mixture hexane:CH_2Cl_2 1:1 as eluent (235.2 mg, 89%). ^{1}H NMR (300 MHz, $CDCl_3$): δ 7.97 (s, 4H) ppm. ^{13}C-NMR (75 MHz, $CDCl_3$): δ 162.3, 134.1, 120.9, 118.5, 117.6 ppm. Anal. calcd. for $C_7H_4N_2O$: C, 63.64; H, 3.05. Found: C, 63.60; H, 3.07. The spectroscopic data are in agreement with reported literature values.[7]

---

[6] Blackburn, O. A.; Coe, B. J.; Helliwell, M. *Organometallics* **2011**, *30*, 4910-4923.
[7] Zarwell, S.; Rueck-Braun, K. *Tetrahedron Lett.* **2008**, *49*, 4020-4025.

2-nitrosobenzonitrile (10a)

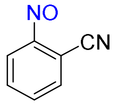

Following the general procedure, starting from 2-aminobenzonitrile (236.3 mg) and stirring the reaction mixture for 3 hours, nitrosobenzene **10a** was obtained as a light yellow solid after column chromatography using a mixture hexane:CH_2Cl_2 1:1 as eluent (171.8 mg, 65%). ^{1}H NMR (300 MHz, $CDCl_3$): δ 8.06 (dd, *J* = 7.6, 1.2 Hz, 1H), 7.86 (td, *J* = 7.6, 1.2 Hz, 1H), 7.76 (td, *J* = 7.8, 1.3 Hz, 1H), 6.98 (dd, *J* = 7.8, 1.2 Hz, 1H) ppm. ^{13}C-NMR (75 MHz, $CDCl_3$): δ 161.9, 135.4, 134.5, 133.6, 116.7, 114.1, 112.3 ppm. Anal. calcd. for $C_7H_4N_2O$: C, 63.64; H, 3.05. Found: C, 63.59; H, 3.02. The spectroscopic data are in agreement with reported literature values.[8]

1-methoxy-3-nitrosobenzene (11a)

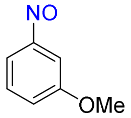

Following the general procedure, starting from 3-methoxyaniline (246.3 mg) and stirring the reaction mixture for 4, nitrosobenzene **11a** was obtained as a white powder after column chromatography using a mixture hexane:CH_2Cl_2 1:1 as eluent (219.5 mg, 80%). ^{1}H NMR (300 MHz, $CDCl_3$): δ 7.86 (dq, *J* = 7.9, 1.0 Hz, 1H), 7.60 (t, *J* = 7.9 Hz, 1H), 7.29 (ddd, *J* = 7.9, 2.6, 1.0 Hz, 1H), 6.90 (dd, *J* = 2.6, 1.0 Hz, 1H), 3.86 (s, 3H) ppm. ^{13}C-NMR (75 MHz, $CDCl_3$): δ 167.0, 160.5, 130.5, 122.9, 119.8, 99.8, 55.8 ppm. Anal. calcd. for $C_7H_7NO_2$: C, 61.31; H, 5.15. Found: C, 61.24; H, 5.13. The spectroscopic data are in agreement with reported literature values.[9]

2-chloro-5-nitrosobenzonitrile (12a)

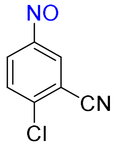

Following the general procedure, starting from 5-amino-2-chlorobenzonitrile (305.2 mg) and stirring the reaction mixture overnight, nitrosobenzene **12a** was obtained as a yellow solid after column chromatography using a mixture hexane:CH_2Cl_2 1:1 as eluent (196.5 mg, 59%). ^{1}H NMR (300 MHz, $CDCl_3$): δ 8.28 (d, *J* = 2.2 Hz, 1H), 7.99 (dd, *J* = 8.5, 2.2 Hz, 1H), 7.81 (d, *J* = 8.5 Hz, 1H) ppm. ^{13}C-NMR (75 MHz, $CDCl_3$): δ 161.1, 144.0, 131.6, 127.0, 124.4, 115.1, 114.7 ppm. Anal. calcd. for $C_7H_3ClN_2O$: C, 50.48; H, 1.82. Found: C, 50.44; H, 1.79.

[8] Jeffrey, J. L.; McClintock, S. P.; Haley, M. M. *J. Org. Chem*. **2008**, *73*, 3288-3291.
[9] Fountoulaki, S.; Gkizis, P. L.; Symeonidis, T. S.; Kaminioti, E.; Karina, A.; Tamiolakis, I.; Armatas, G. S.; Lykakis. I. N *Adv. Synth. Catal*. **2016**, *358*, 1500-1508.

3-chloro-4-nitrosobenzonitrile (13a)

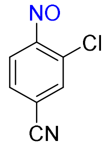

Following the general procedure, starting from 4-amino-3-chlorobenzonitrile (305.2 mg) and stirring the reaction mixture overnight, nitrosobenzene **13a** was obtained as a yellow solid after column chromatography using a mixture hexane:CH_2Cl_2 1:1 as eluent (196.5 mg, 59%). ^{1}H NMR (300 MHz, $CDCl_3$): δ 8.14 (d, *J* = 1.5 Hz, 1H), 7.56 (dd, *J* = 8.3, 1.5 Hz, 1H), 6.23 (d, *J* = 8.3 Hz, 1H) ppm. ^{13}C-NMR (75 MHz, $CDCl_3$): δ 157.8, 142.4, 136.6, 131.0, 120.0, 116.4, 109.4 ppm. Anal. calcd. for $C_7H_3ClN_2O$: C, 50.48; H, 1.82. Found: C, 50.53; H, 1.78.

3-chloro-2-nitrosobenzonitrile (14a)

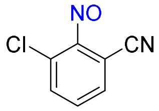

Following the general procedure, starting from 2-amino-3-chlorobenzonitrile (305.2 mg) and stirring the reaction mixture overnight, nitrosobenzene **14a** was obtained as a yellow solid after column chromatography using a mixture hexane:CH_2Cl_2 1:1 as eluent (193.2 mg, 58%). ^{1}H NMR (300 MHz, $CDCl_3$): δ 8.05 (dd, *J* = 8.1, 1.2 Hz, 1H), 7.75 (t, *J* = 8.1 Hz, 1H), 7.64 (dd, *J* = 7.6, 1.2 Hz, 1H) ppm. ^{13}C-NMR (75 MHz, $CDCl_3$): δ 158.4, 141.1, 137.1, 135.6, 133.0, 116.1, 96.0 ppm. Anal. calcd. for $C_7H_3ClN_2O$: C, 50.48; H, 1.82. Found: C, 50.52; H, 1.78.

4-chloro-2-nitrosobenzonitrile (15a)

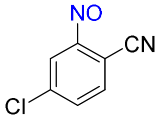

Following the general procedure, starting from 2-amino-4-chlorobenzonitrile (305.2 mg) and stirring the reaction mixture overnight, nitrosobenzene **15a** was obtained as a yellow solid after column chromatography using a mixture hexane:CH_2Cl_2 1:1 as eluent (219.5 mg, 66%). ^{1}H NMR (300 MHz, $CDCl_3$): δ 8.03 (d, *J* = 8.2 Hz, 1H), 7.81 (dd, *J* = 8.2, 2.1 Hz, 1H), 6.83 (d, *J* = 2.1 Hz, 1H) ppm. ^{13}C-NMR (75 MHz, $CDCl_3$): δ 161.0, 141.0, 135.5, 134.9, 115.9, 113.3, 111.9 ppm. Anal. calcd. for $C_7H_3ClN_2O$: C, 50.48; H, 1.82. Found: C, 50.55; H, 1.79.

5-chloro-2-nitrosobenzonitrile (16a)

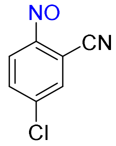

Following the general procedure, starting from 2-amino-5-chlorobenzonitrile (305.2 mg) and stirring the reaction mixture overnight, nitrosobenzene **16a** was obtained as a yellow solid after column

chromatography using a mixture hexane:CH_2Cl_2 1:1 as eluent (229.8 mg, 69%). ^{1}H NMR (300 MHz, $CDCl_3$): δ 8.02 (d, *J* = 2.1 Hz, 1H), 7.72 (dd, *J* = 8.6, 2.1 Hz, 1H), 6.91 (d, *J* = 8.6 Hz, 1H) ppm. ^{13}C-NMR (75 MHz, $CDCl_3$): δ 160.0, 142.6, 134.1, 133.9, 115.8, 115.4, 113.3 ppm. Anal. calcd. for $C_7H_3ClN_2O$: C, 50.48; H, 1.82. Found: C, 50.40; H, 1.77.

5-bromo-2-nitrosobenzonitrile (17a)

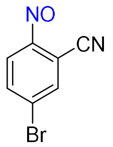

Following the general procedure, starting from 2-amino-5-bromobenzonitrile (394.1 mg) and stirring the reaction mixture overnight, nitrosobenzene **17a** was obtained as a yellow solid after column chromatography using a mixture hexane:EtOAc 9:1 as eluent (240.6 mg, 57%). ^{1}H NMR (300 MHz, $CDCl_3$): δ 8.20 (d, *J* = 1.9 Hz, 1H), 7.89 (dd, *J* = 8.6, 1.9 Hz, 1H), 6.82 (d, *J* = 8.6 Hz, 1H) ppm. ^{13}C-NMR (75 MHz, $CDCl_3$): δ 191.4, 163.9, 139.6, 131.2, 121.2 ppm. Anal. calcd. for $C_7H_3BrN_2O$: C, 39.84; H, 1.43. Found: C, 39.99; H, 1.39.

2-nitroso-4-(trifluoromethyl)benzonitrile (18a)

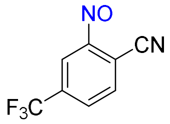

Following the general procedure, starting from 2-amino-4-(trifluoromethyl)benzonitrile (372.3 mg) and stirring the reaction mixture overnight, nitrosobenzene **18a** was obtained as a yellow solid after column chromatography using a mixture hexane:CH_2Cl_2 1:1 as eluent (244.1 mg, 61%). ^{1}H NMR (300 MHz, $CDCl_3$): δ 8.29 (d, *J* = 7.9 Hz, 1H), 8.12 (dd, *J* = 7.9, 0.7 Hz, 1H), 7.16 (d, *J* = 0.7 Hz, 1H) ppm. ^{13}C-NMR (75 MHz, $CDCl_3$): δ 160.3, 135.6 (c, *J* = 34.8 Hz), 135.5, 131.5 (c, *J* = 3.3 Hz), 122.3 (c, *J* = 273.0 Hz), 117.4, 115.4, 109.2 (c, *J* = 4.0 Hz) ppm. ^{19}F-NMR (282 MHz, $CDCl_3$): δ -63.5 ppm. Anal. calcd. for $C_8H_3F_3N_2O$: C, 48.02; H, 1.51. Found: C, 47.95; H, 1.54.

2-methyl-6-nitrosobenzonitrile (19a)

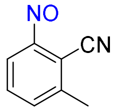

Following the general procedure, starting from 2-amino-6-methylbenzonitrile (264.3 mg) and stirring the reaction mixture overnight, nitrosobenzene **19a** was obtained as a green solid after column chromatography using a mixture hexane:CH_2Cl_2 1:1 as eluent (207.5 mg, 71%). ^{1}H NMR (300 MHz, $CDCl_3$): δ 7.71 (d, *J* = 7.8 Hz, 1H), 7.60 (d, *J* = 7.8 Hz, 1H), 6.73 (d, *J* = 7.8 Hz, 1H), 2.81 (s, 3H) ppm. ^{13}C-NMR (75 MHz, $CDCl_3$): δ 162.8, 144.8, 136.6, 132.7, 115.8, 115.1, 109.0, 20.4 ppm. Anal. calcd. for $C_8H_6N_2O$: C, 65.75; H, 4.14. Found: C, 65.70; H, 4.16.

5-methyl-2-nitrosobenzonitrile (20a)

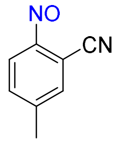

Following the general procedure, starting from 2-amino-5-methylbenzonitrile (264.3 mg) and stirring the reaction mixture overnight, nitrosobenzene **20a** was obtained as a green solid after column chromatography using a mixture hexane:CH_2Cl_2 1:1 as eluent (207.5 mg, 71%). ^{1}H NMR (300 MHz, $CDCl_3$): δ 7.82 (s, 1H), 7.52 (d, *J* = 8.2 Hz, 1H), 6.93 (d, *J* = 8.2 Hz, 1H), 2.51 (s, 3H) ppm. ^{13}C-NMR (75 MHz, $CDCl_3$): δ 161.7, 147.5, 134.6, 134.0, 116.9, 114.2, 112.6, 21.8 ppm. Anal. calcd. for $C_8H_6N_2O$: C, 65.75; H, 4.14. Found: C, 65.79; H, 4.18.

5-methoxy-2-nitrosobenzonitrile (21a)

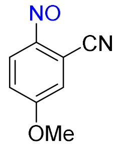

Following the general procedure, starting from 2-amino-5-methoxybenzonitrile (296.3 mg) and stirring the reaction mixture overnight, nitrosobenzene **21a** was obtained as a green solid after column chromatography using a mixture hexane:CH_2Cl_2 1:1 as eluent (217.3 mg, 67%). ^{1}H NMR (300 MHz, $CDCl_3$): δ 7.42 (d, *J* = 2.6 Hz, 1H), 7.14 (dd, *J* = 9.1, 2.6 Hz, 1H), 7.04 (d, *J* = 9.1 Hz, 1H), 4.00 (s, 3H) ppm. ^{13}C-NMR (75 MHz, $CDCl_3$): δ 164.9, 160.2, 118.5, 118.4, 117.4, 116.6, 115.3, 56.8 ppm. Anal. calcd. for $C_8H_6N_2O_2$: C, 59.26; H, 3.73. Found: C, 59.18; H, 3.78.

4-methoxy-2-nitrosobenzonitrile (22a)

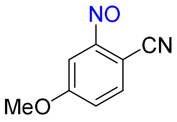

Following the general procedure, starting from 2-amino-4-methoxybenzonitrile (296.3 mg) and stirring the reaction mixture overnight, nitrosobenzene **22a** was obtained as a green solid after column chromatography using a mixture hexane:CH_2Cl_2 1:1 as eluent (201.1 mg, 62%). ^{1}H NMR (300 MHz, $CDCl_3$): δ 7.97 (d, *J* = 8.5 Hz, 1H), 7.34 (dd, *J* = 8.5, 2.7 Hz, 1H), 6.27 (d, *J* = 2.7 Hz, 1H), 3.89 (s, 3H) ppm. ^{13}C-NMR (75 MHz, $CDCl_3$): δ 163.4, 135.6, 121.9, 117.0, 107.6, 95.2, 56.4 ppm. Anal. calcd. for $C_8H_6N_2O_2$: C, 59.26; H, 3.73. Found: C, 59.19; H, 3.70.

4. Synthesis of nitrosoarenes by direct nitrosation of heterocycles:

2-Nitrosopyridine (**23a**) is commercially available (CAS: 79917-37-6).

Synthesis of ethyl 3-nitrosoimidazo[1,2-*a*]pyridine-2-carboxylate (24a):

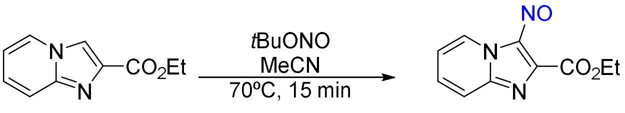

24a

A mixture of ethyl imidazo[1,2-*a*]pyridine-2-carboxylate (38.0 mg, 0.2 mmol) and *t*BuONO (28 mg, 33µL, 0.24 mmol) in acetonitrile (1 mL) was stirred for 15 min at 70 °C. After evaporation of the solvent under vacuum, imidazopyridine **24a** was purified by flash column chromatography (Hexane:AcOEt 1:1) and obtained as a green solid in 73% yield (32.0 mg). ^{1}H NMR (300 MHz, $CDCl_3$): δ 9.67 (d, *J* = 6.9 Hz, 1H), 8.00 (d, *J* = 8.8 Hz, 1H), 7.84-7.90 (m, 1H), 7.41 (td, *J* = 6.9, 1.1 Hz, 1H), 4.69 (c, *J* = 7.1 Hz, 2H), 1.54 (t, *J* = 7.1 Hz, 3H) ppm. ^{13}C NMR (75 MHz, $CDCl_3$): δ 161.5, 153.8, 143.7, 135.6, 125.8, 121.9, 118.9, 63.1, 14.4 ppm. Anal. calcd. for $C_{10}H_9N_3O_3$: C, 54.79; H, 4.14. Found: C, 54.86; H, 4.15.

Synthesis of 1-methyl-3-nitroso-2-phenyl-1*H*-indole (25a):

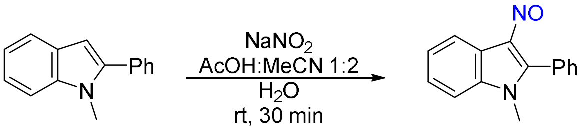

25a

To a solution of 1-methyl-2-phenylindole (500.0 mg, 2.5 mmol) in 12 mL of mixed AcOH:MeCN = 1:2 a solution of $NaNO_2$ (207.5 mg, 3 mmol) in water (2 mL) was added dropwise. The resulting mixture was stirred for 30 min at room temperature then diluted with water (15 mL). The mixture was filtered and the solid was washed with water. After purification by column chromatography on silica gel (Hexane:EtOAc 7:3) indole **25a** was obtained in 90% yield (535.0 mg) as a green solid. ^{1}H NMR (300 MHz, $CDCl_3$): δ 8.28-8.31 (m, 1H), 7.82-7.86 (m, 2H), 7.57-7.63 (m, 3H), 7.37-7.45 (m, 3H), 3.87 (s, 3H) ppm. ^{13}C NMR (75 MHz, $CDCl_3$): δ 157.1, 156.6, 136.3, 131.8, 130.5, 128.5, 127.9, 127.4, 126.1, 121.7, 113.4, 109.5, 31.9 ppm. Anal. calcd. for $C_{15}H_{12}N_2O$: C, 76.25; H, 5.12. Found: C, 76.20; H, 5.14. The spectroscopic data are in agreement with reported literature values.[10]

[10] Wu, Y.; Pi, C.; Cui, X.; Wu, Y. *Org. Lett.* **2020**, *22*, 361-364.

5. Spectra of isolated products

Figure S1. 1a, ^1H NMR (300MHz, $CDCl_3$)

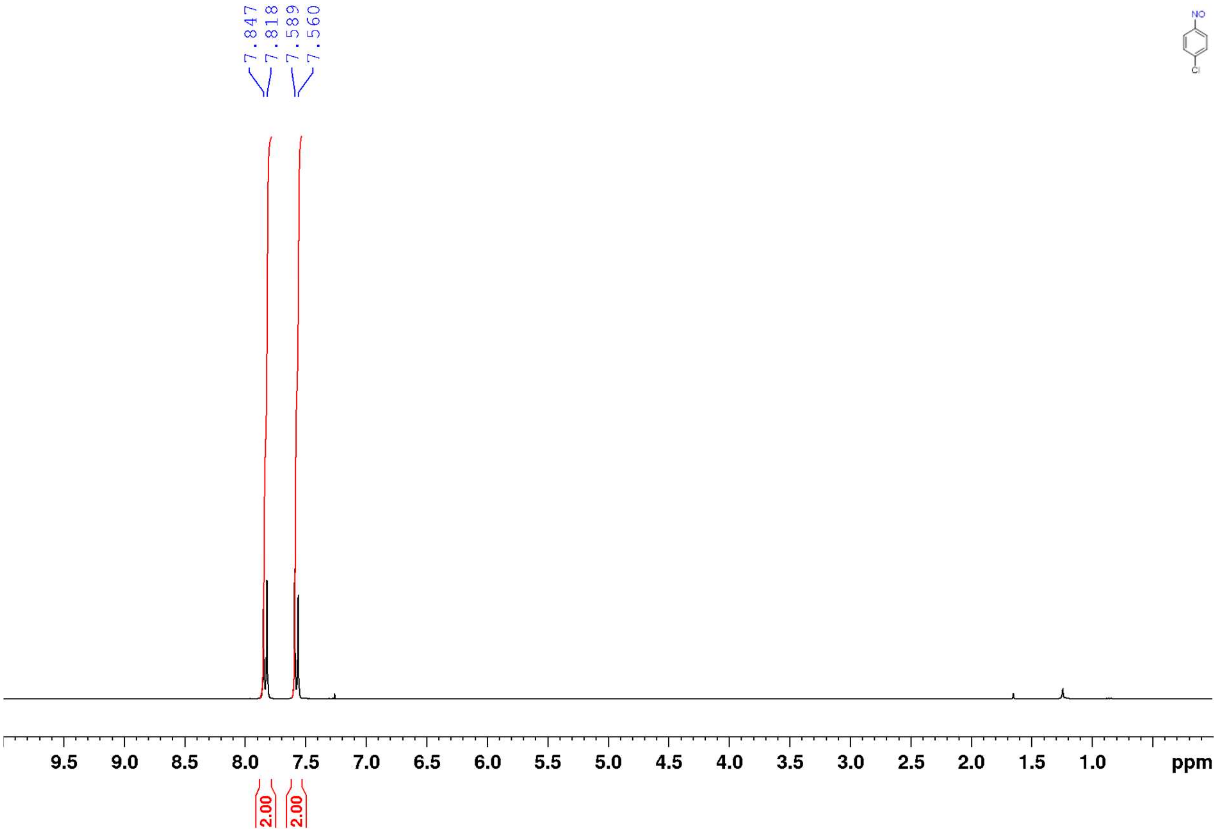

Figure S2. 1a, ^{13}C NMR (75MHz, $CDCl_3$)

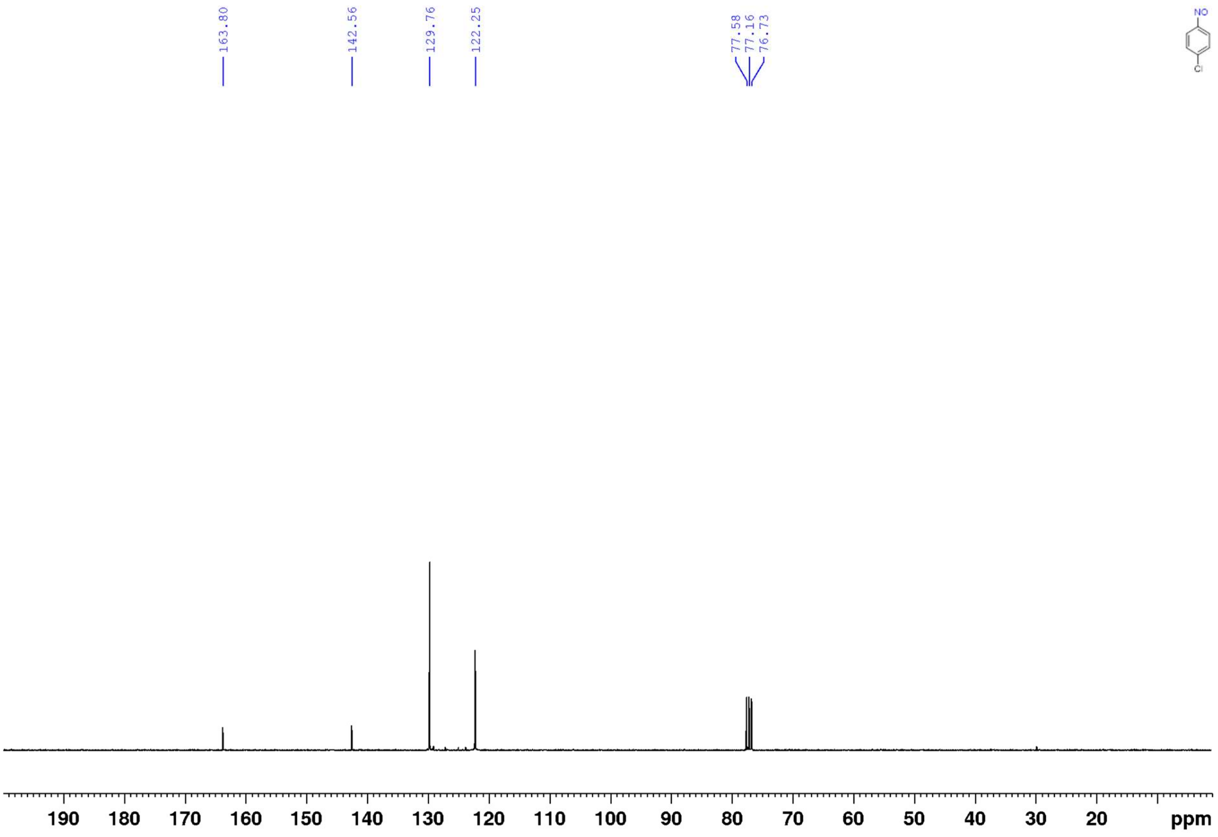

Figure S3. 1c, ^{1}H NMR (300MHz, $CDCl_3$)

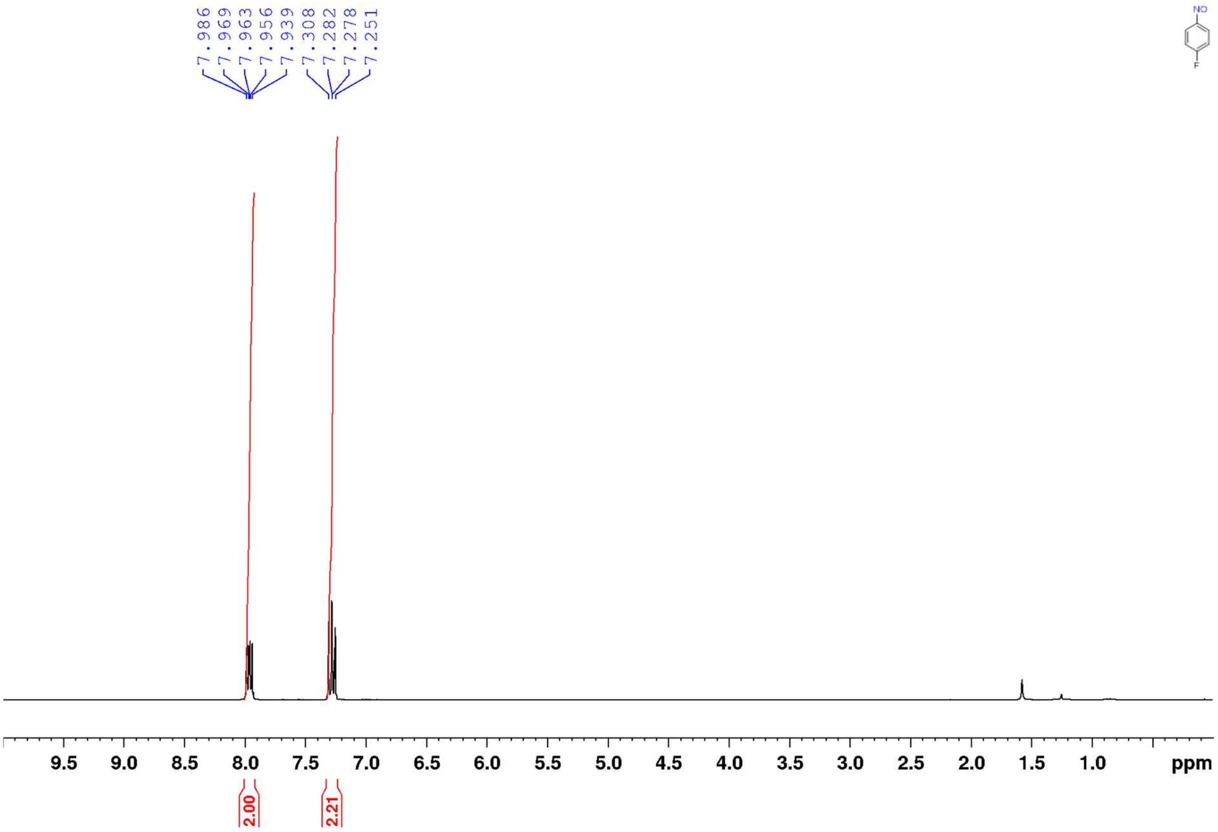

Figure S4. 1c, ^{13}C NMR (75MHz, $CDCl_3$)

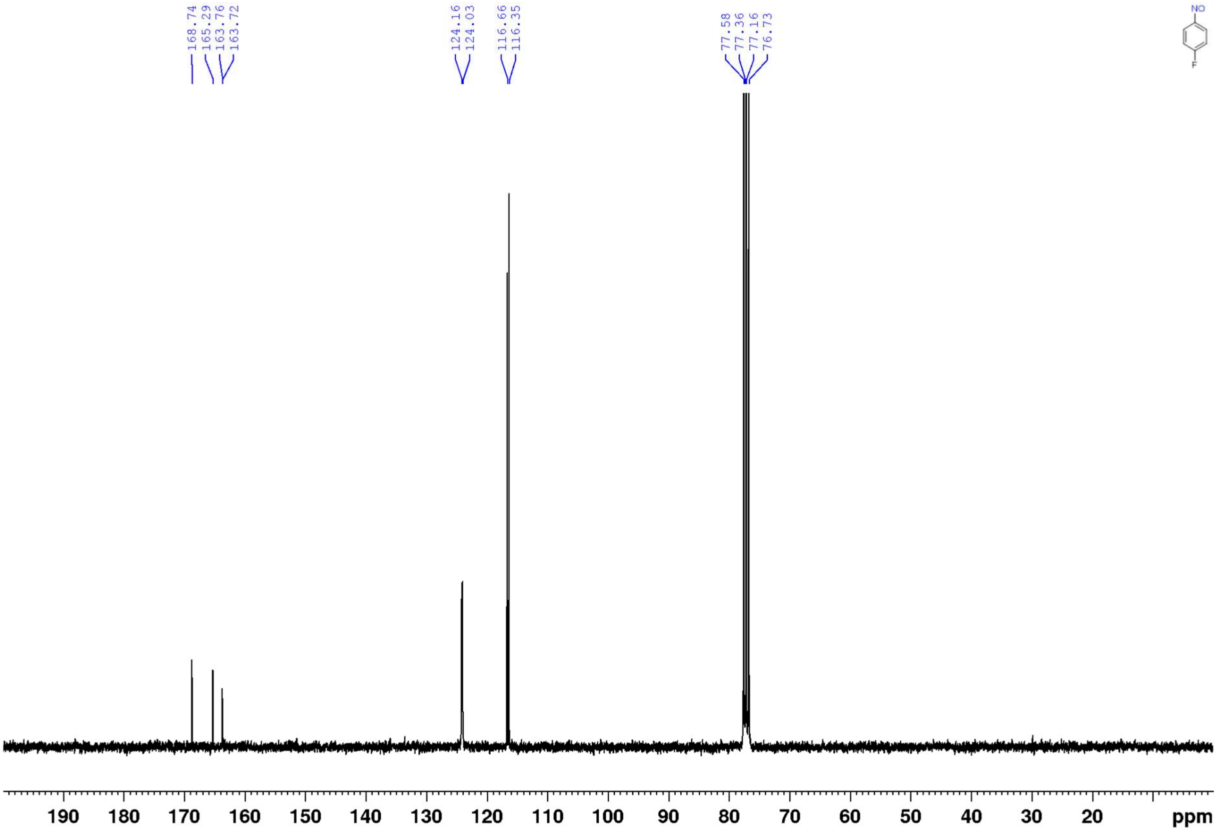

Figure S5. 2a, ^1H NMR (300MHz, $CDCl_3$)

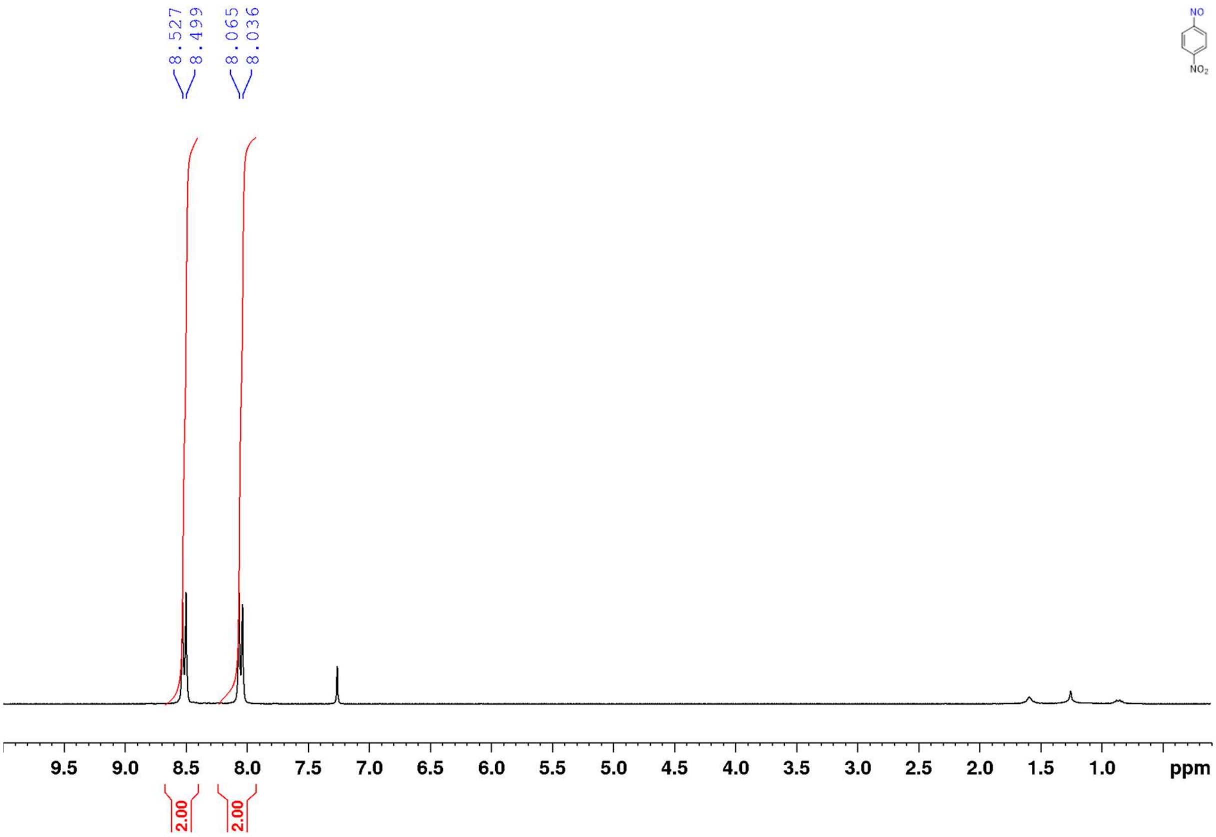

Figure S6. 2a, ^{13}C NMR (75MHz, $CDCl_3$)

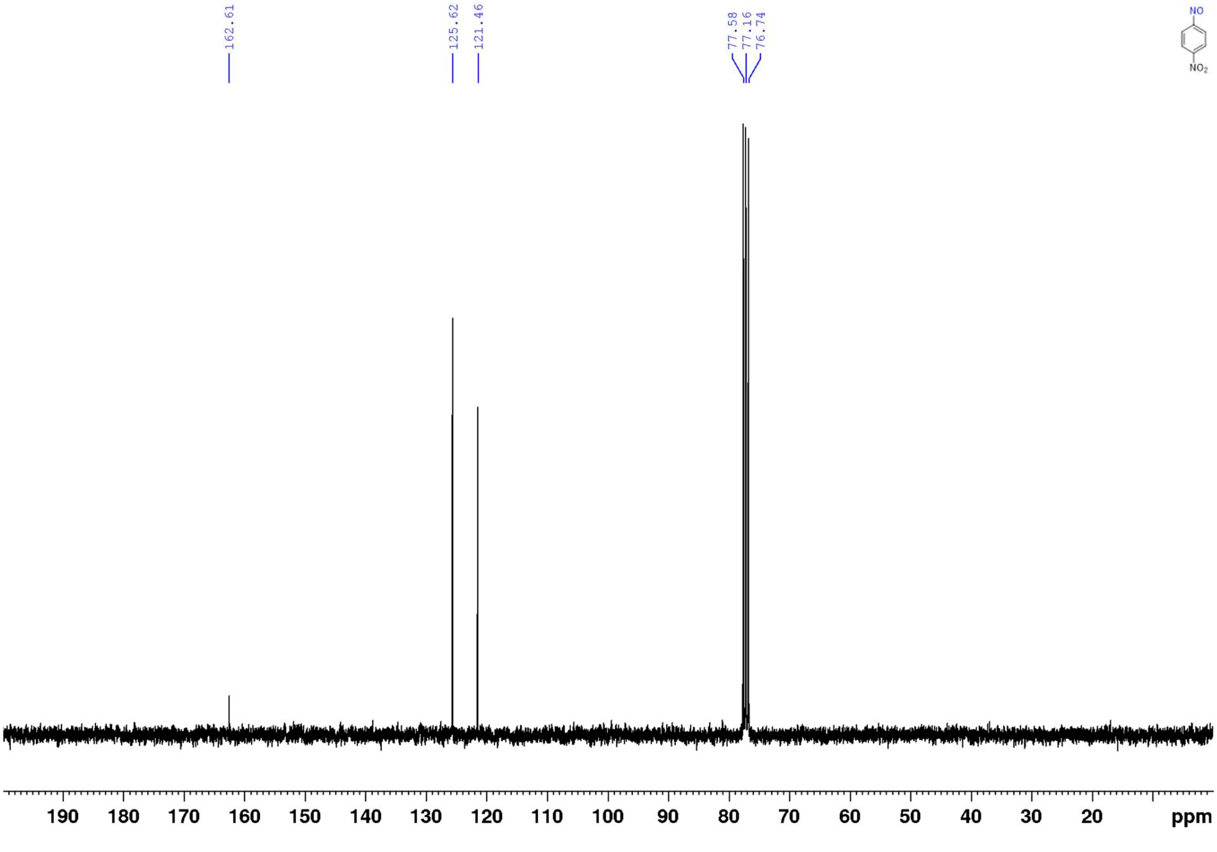

Figure S7. 3a, ^{1}H NMR (300MHz, $CDCl_3$)

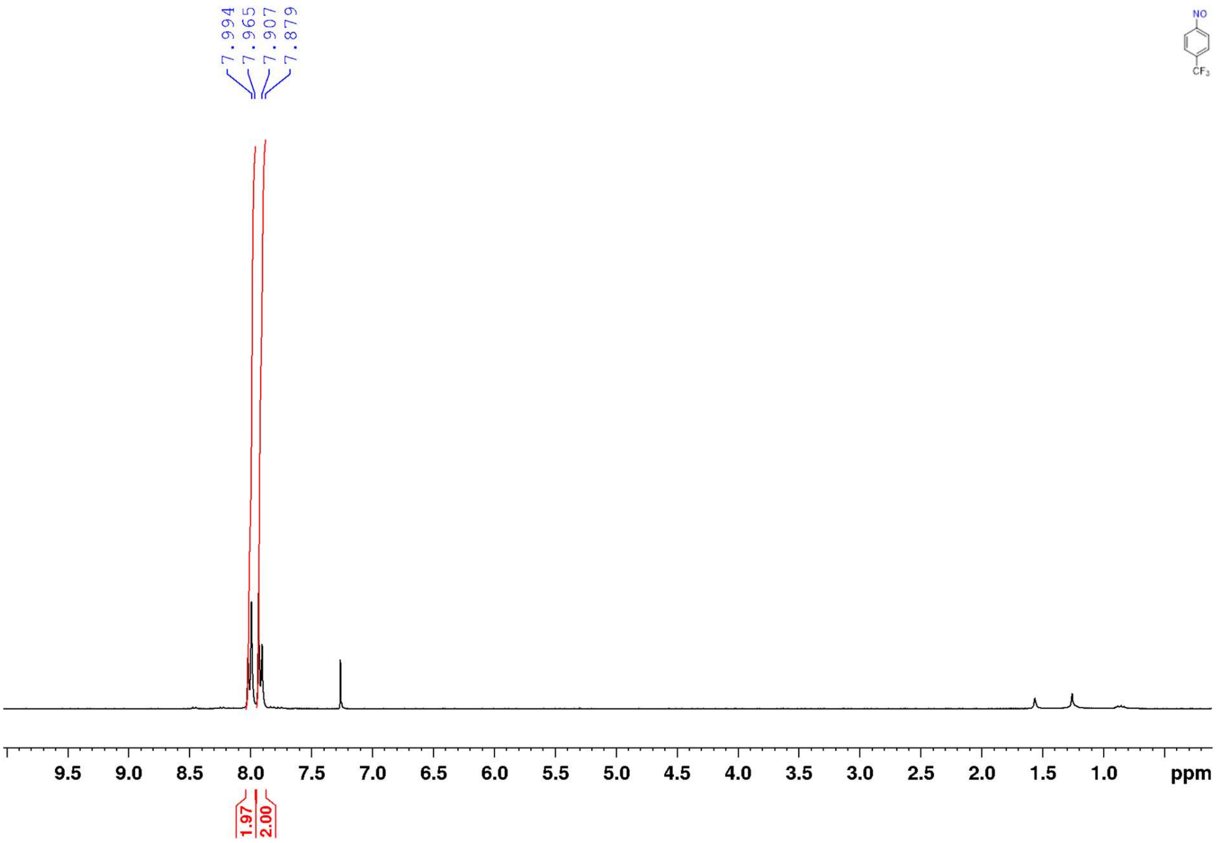

Figure S8. 3a, ^{13}C NMR (75MHz, $CDCl_3$)

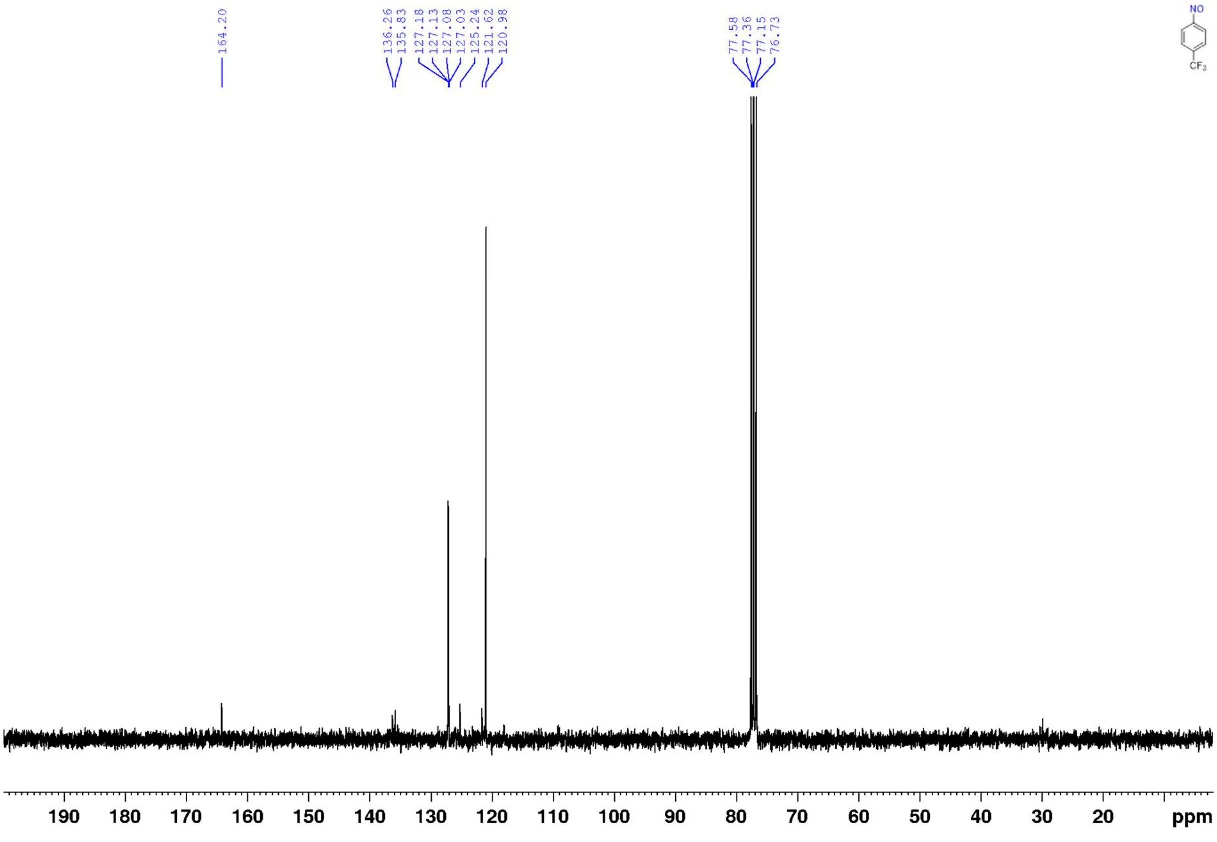

Figure S9. 4a, ^{1}H NMR (300MHz, $CDCl_3$)

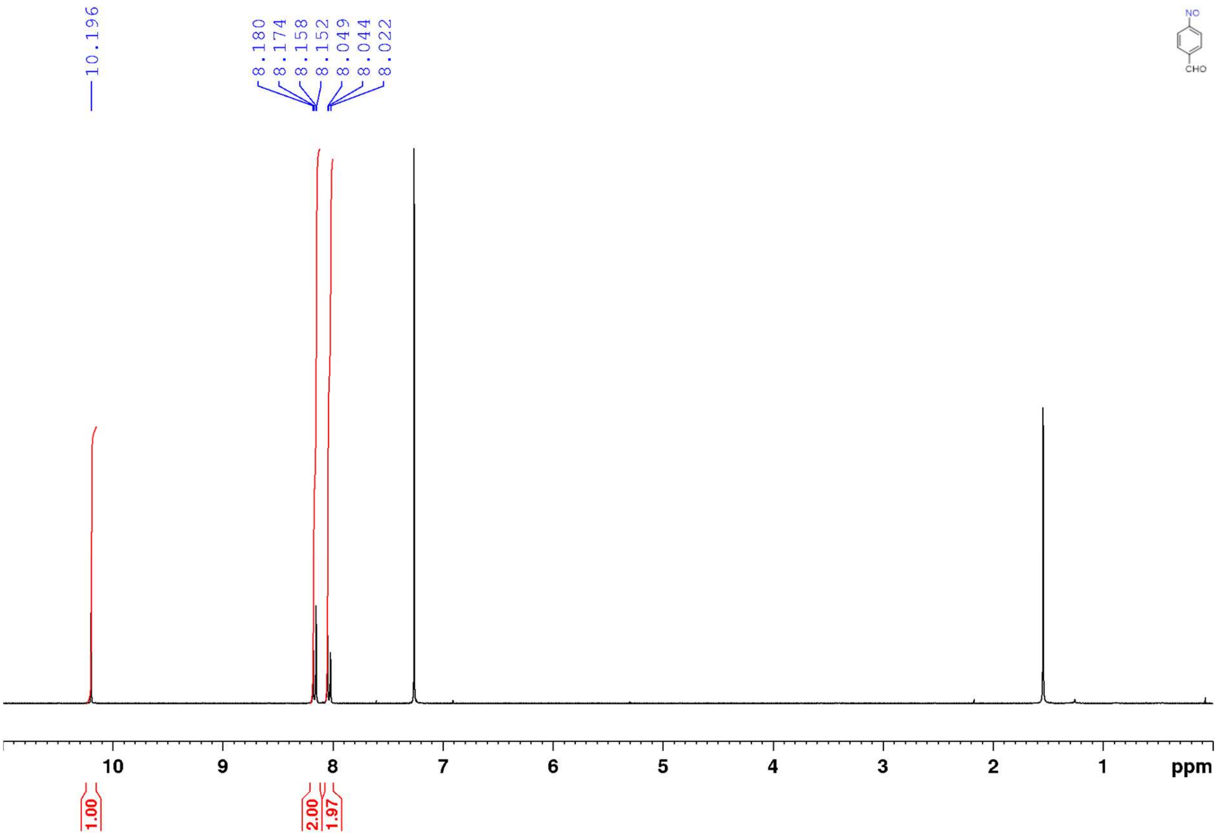

Figure S10. 4a, ^{13}C NMR (75MHz, $CDCl_3$)

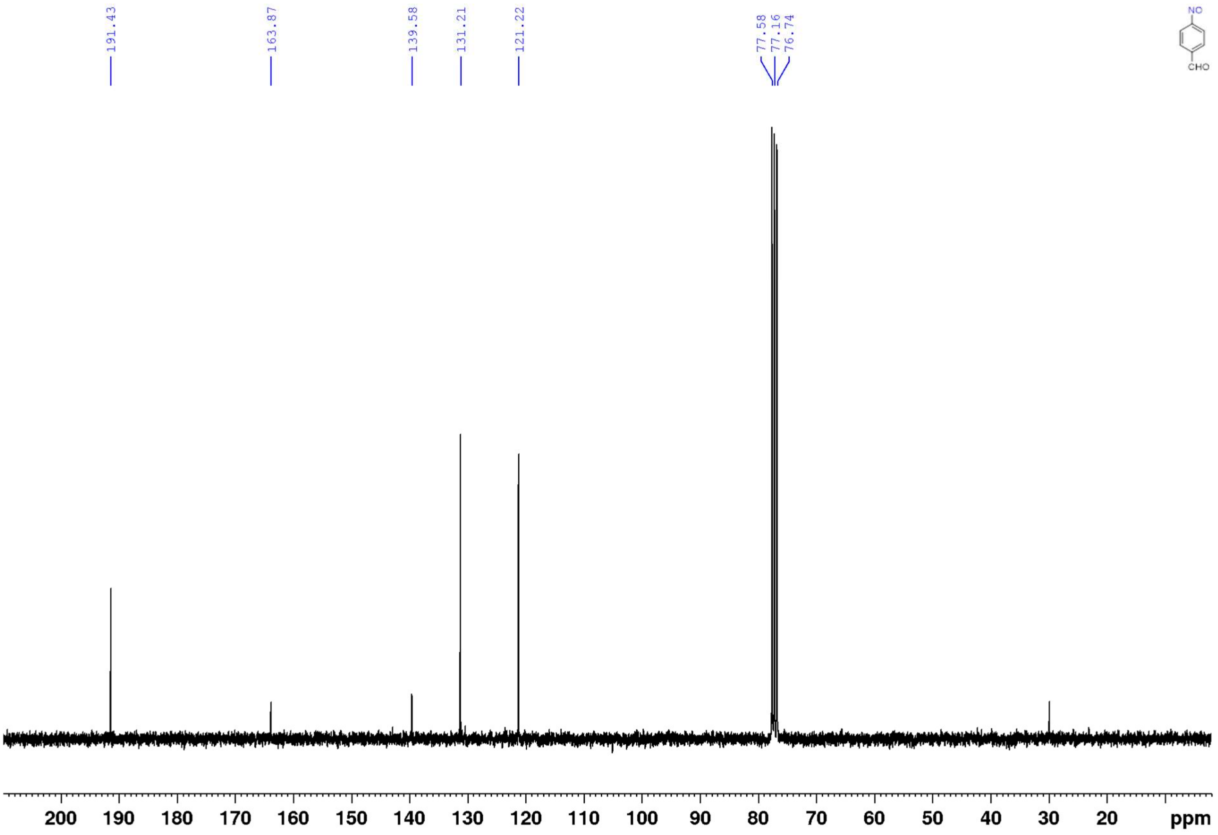

Figure S11. 5a, ^{1}H NMR (300MHz, $CDCl_3$)

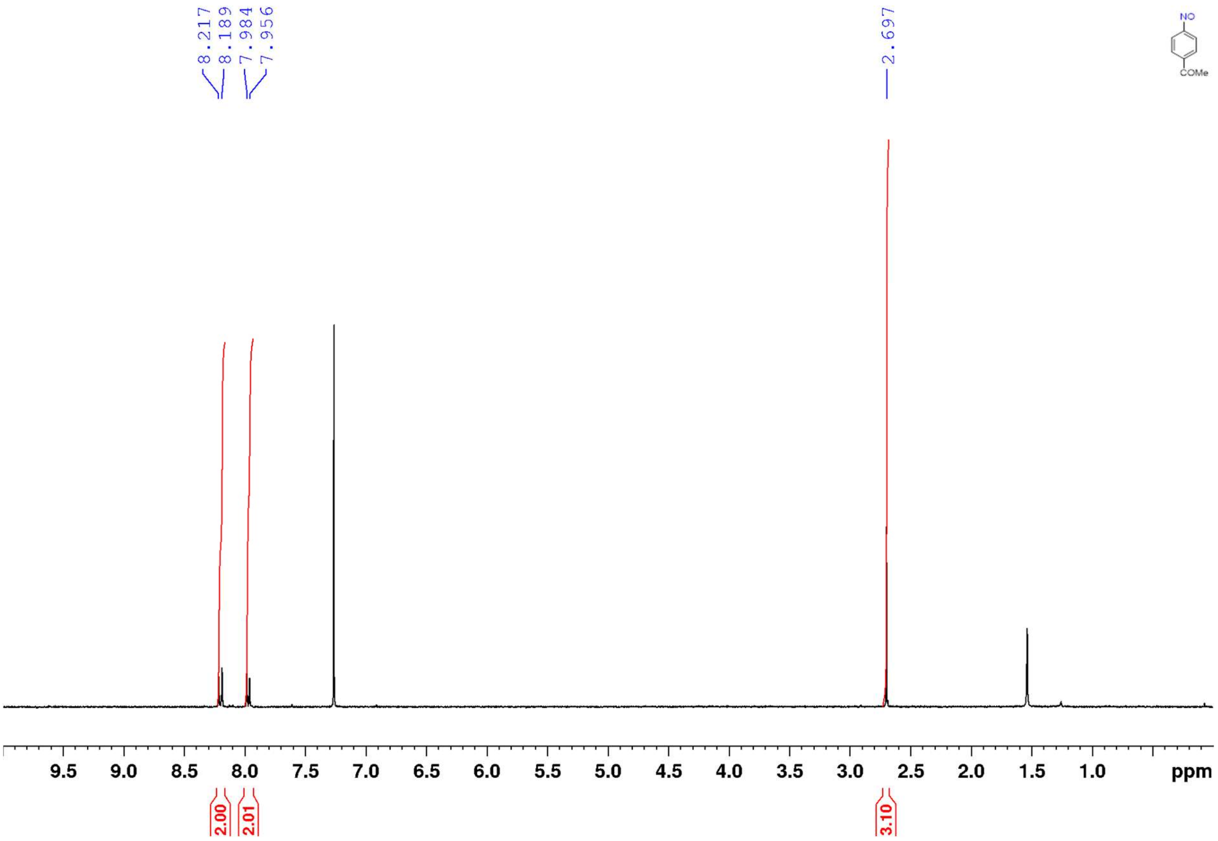

Figure S12. 5a, ^{13}C NMR (75MHz, $CDCl_3$)

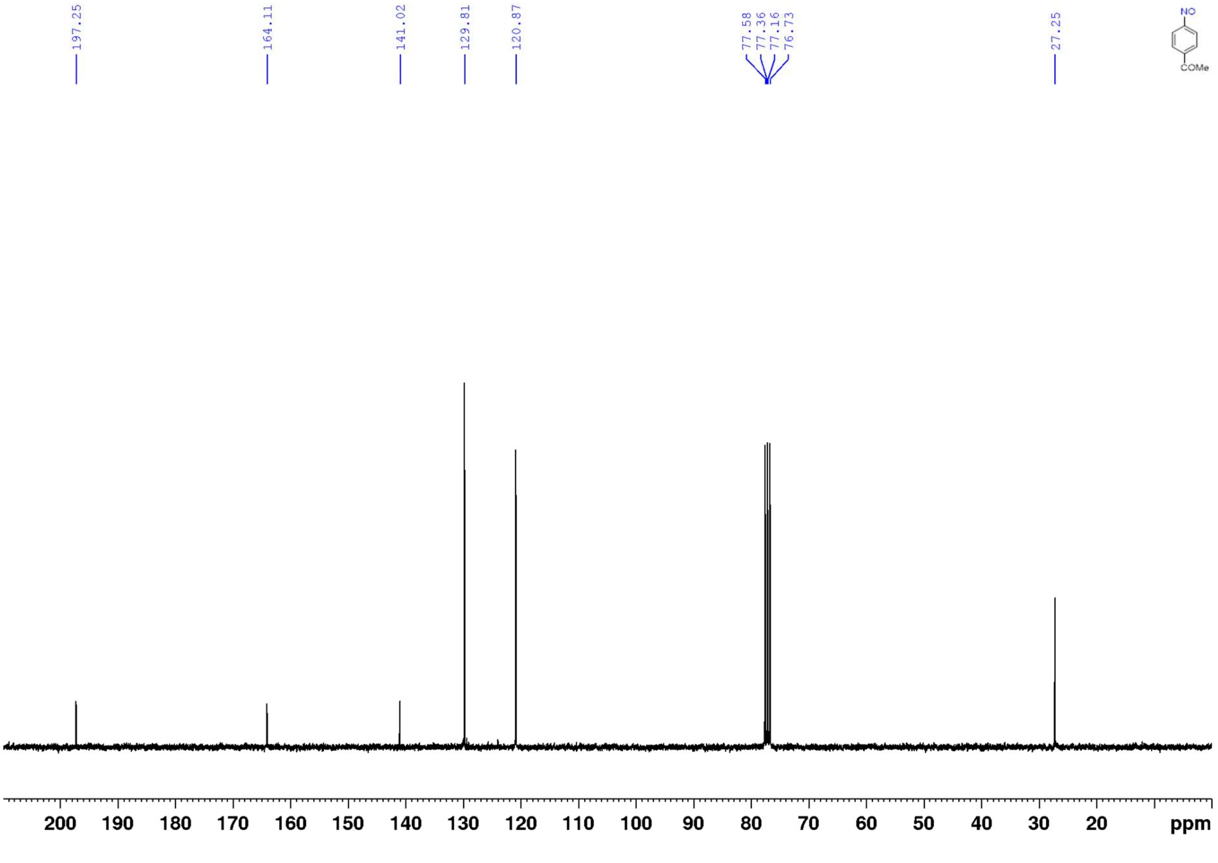

Figure S13. 6a, ^{1}H NMR (300MHz, $CDCl_3$)

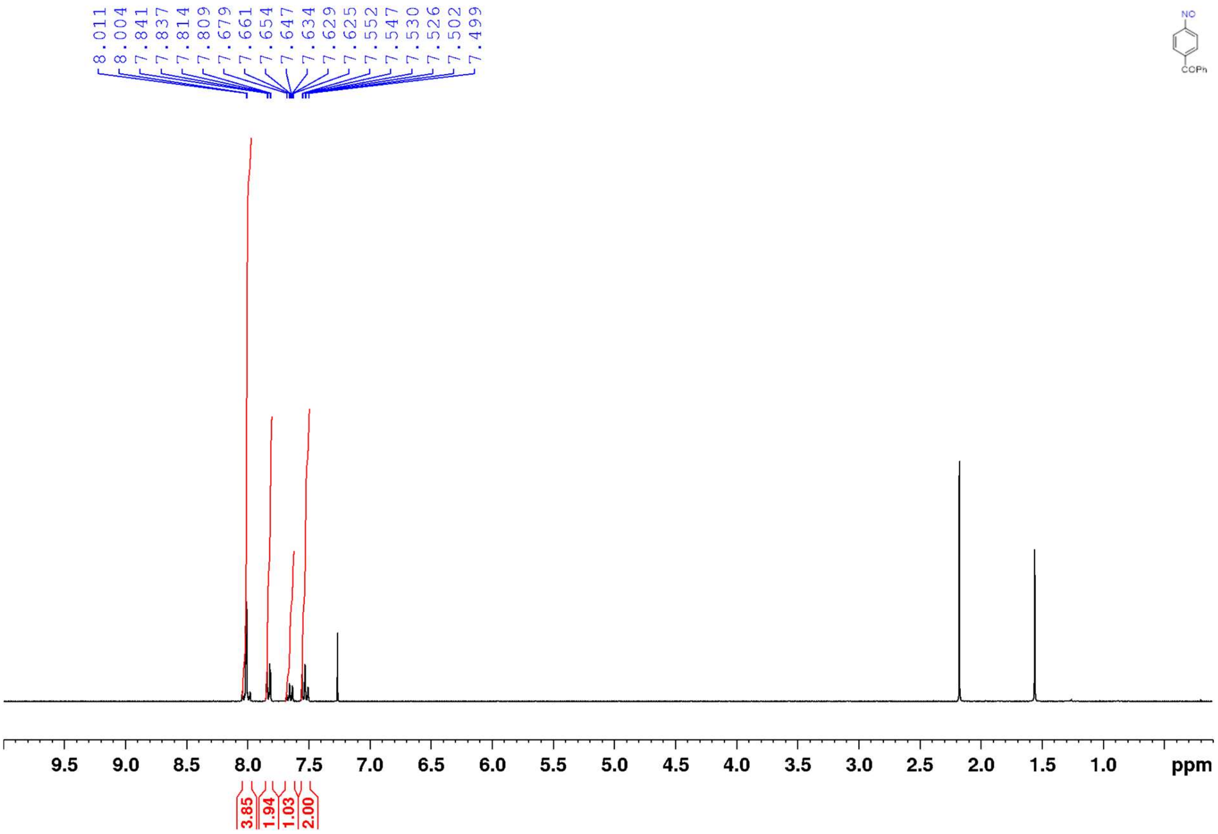

Figure S14. 6a, ^{13}C NMR (75MHz, $CDCl_3$)

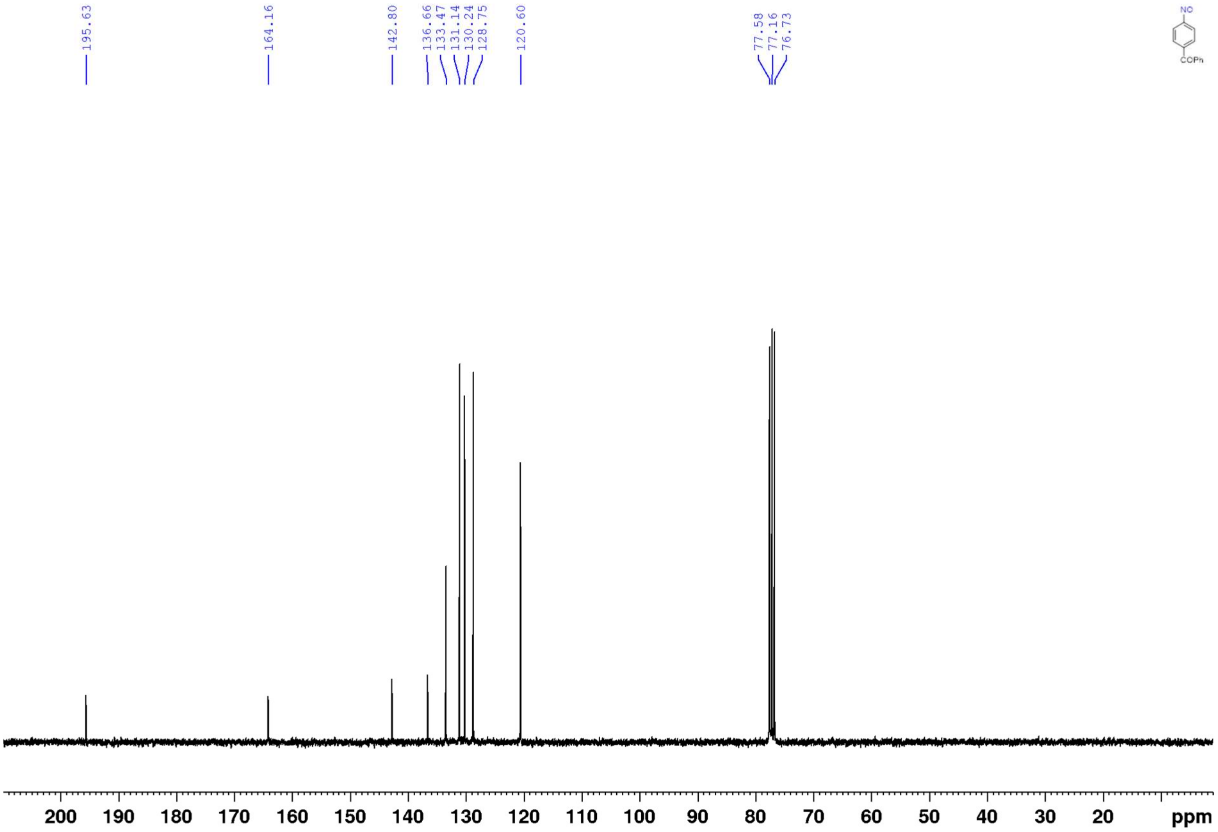

Figure S15. 7a, ^{1}H NMR (300MHz, CDCl$_3$)

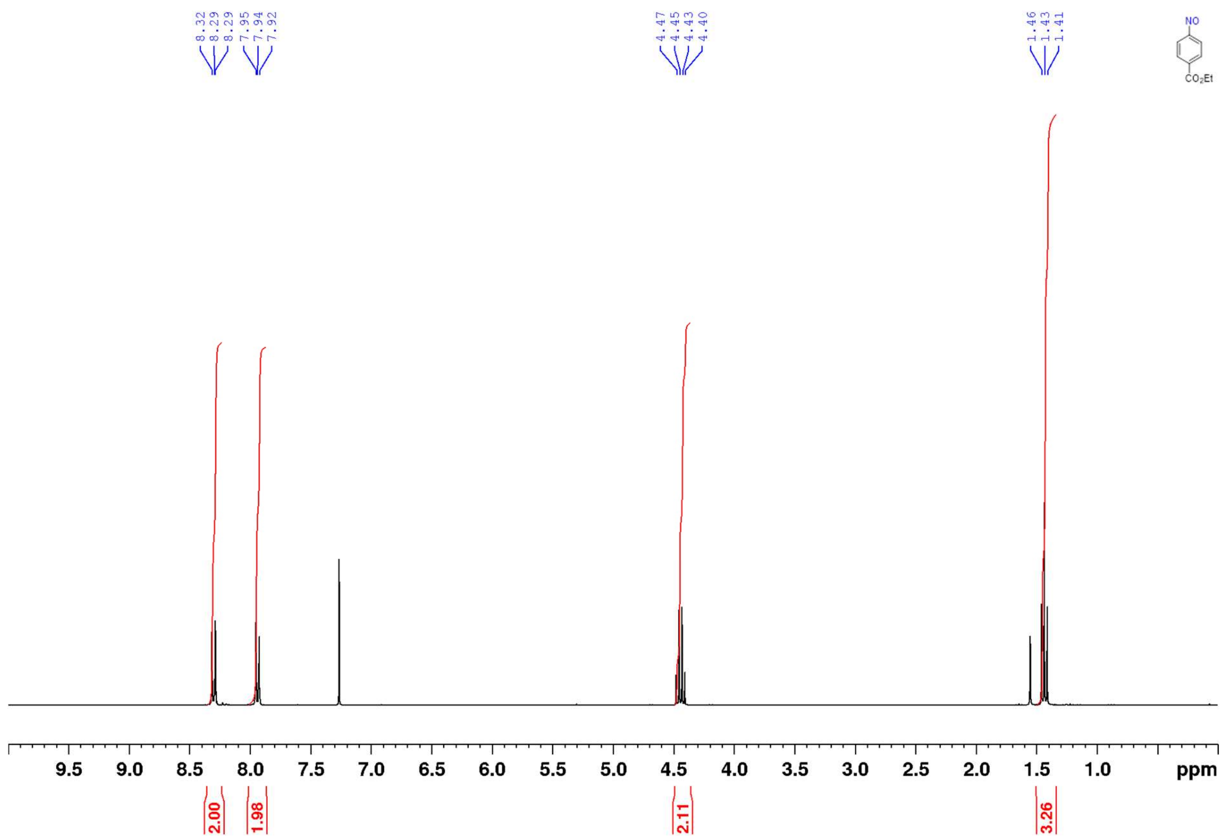

Figure S16. 7a, ^{13}C NMR (75MHz, CDCl$_3$)

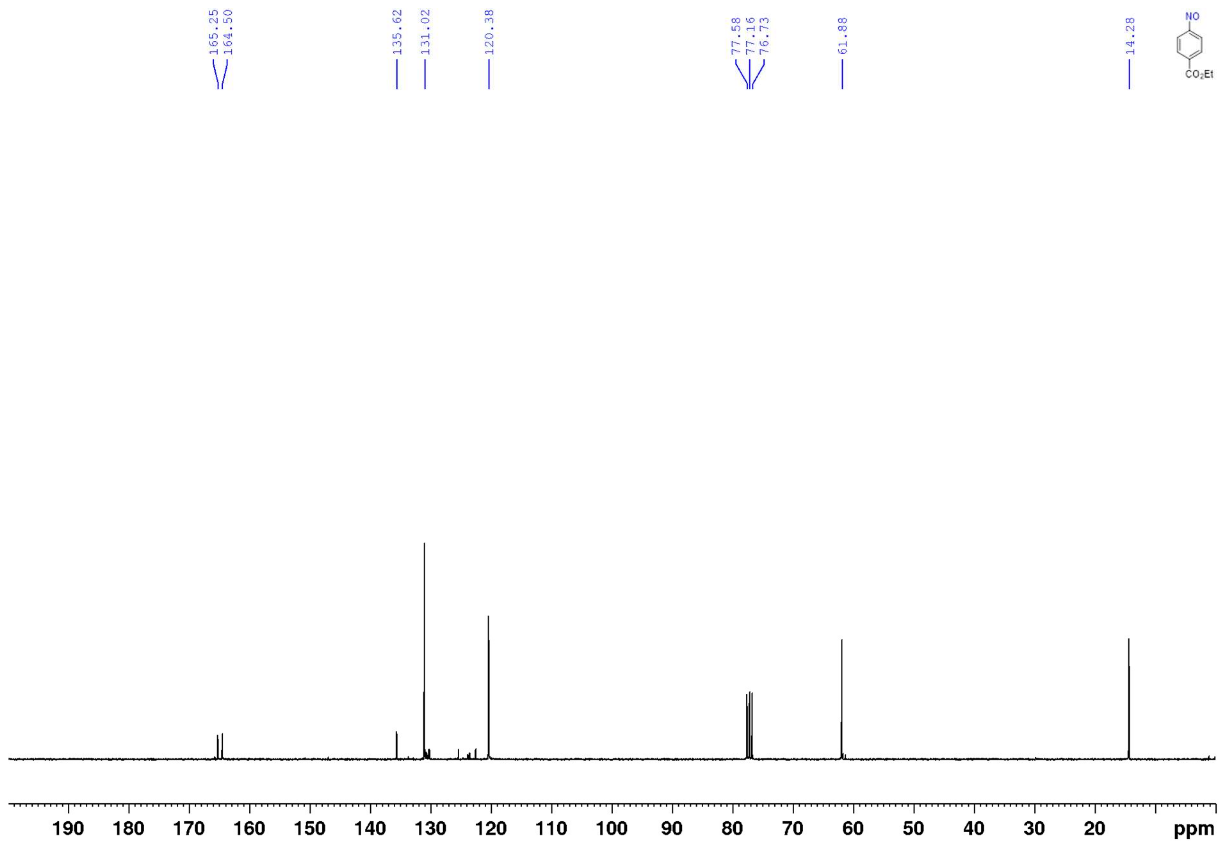

Figure S17. 8a, ^{1}H NMR (300MHz, $CDCl_3$)

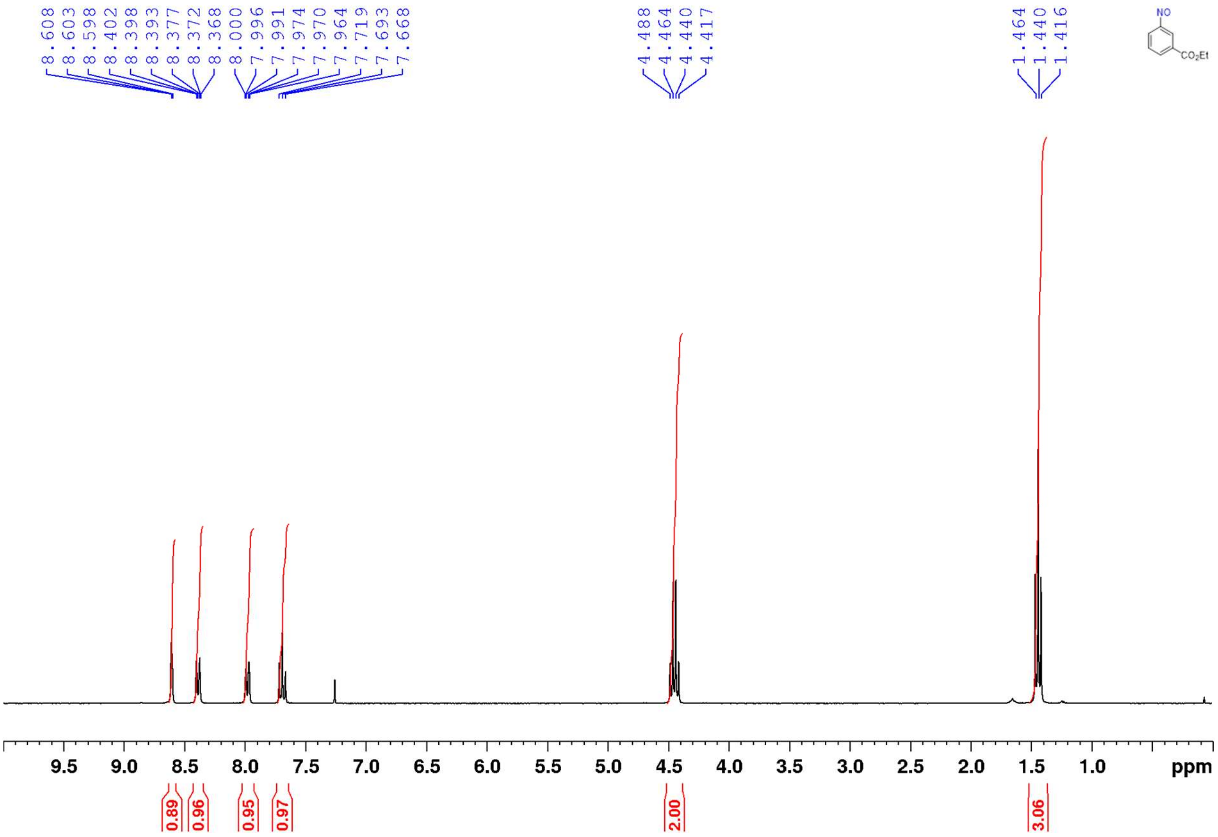

Figure S18. 8a, ^{13}C NMR (75MHz, $CDCl_3$)

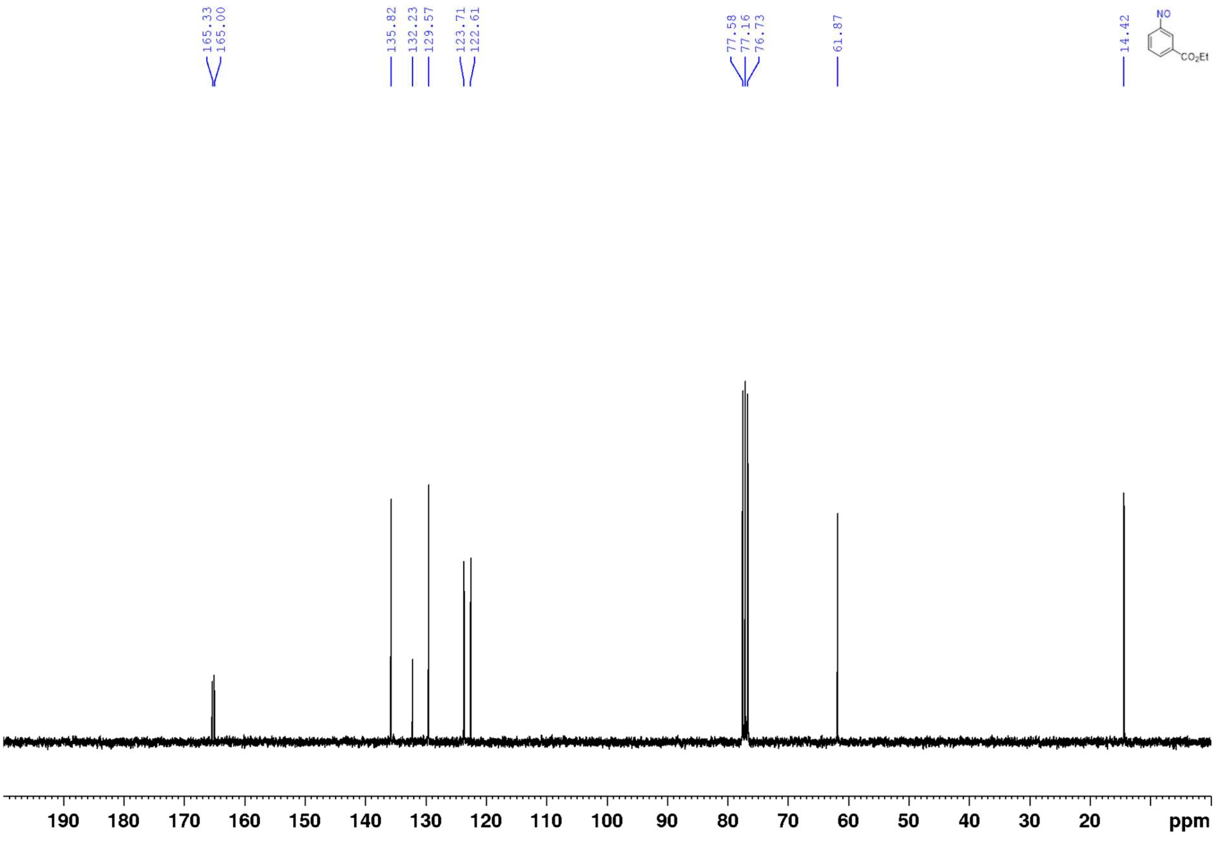

Figure S19. 9a, ^{1}H NMR (300MHz, $CDCl_3$)

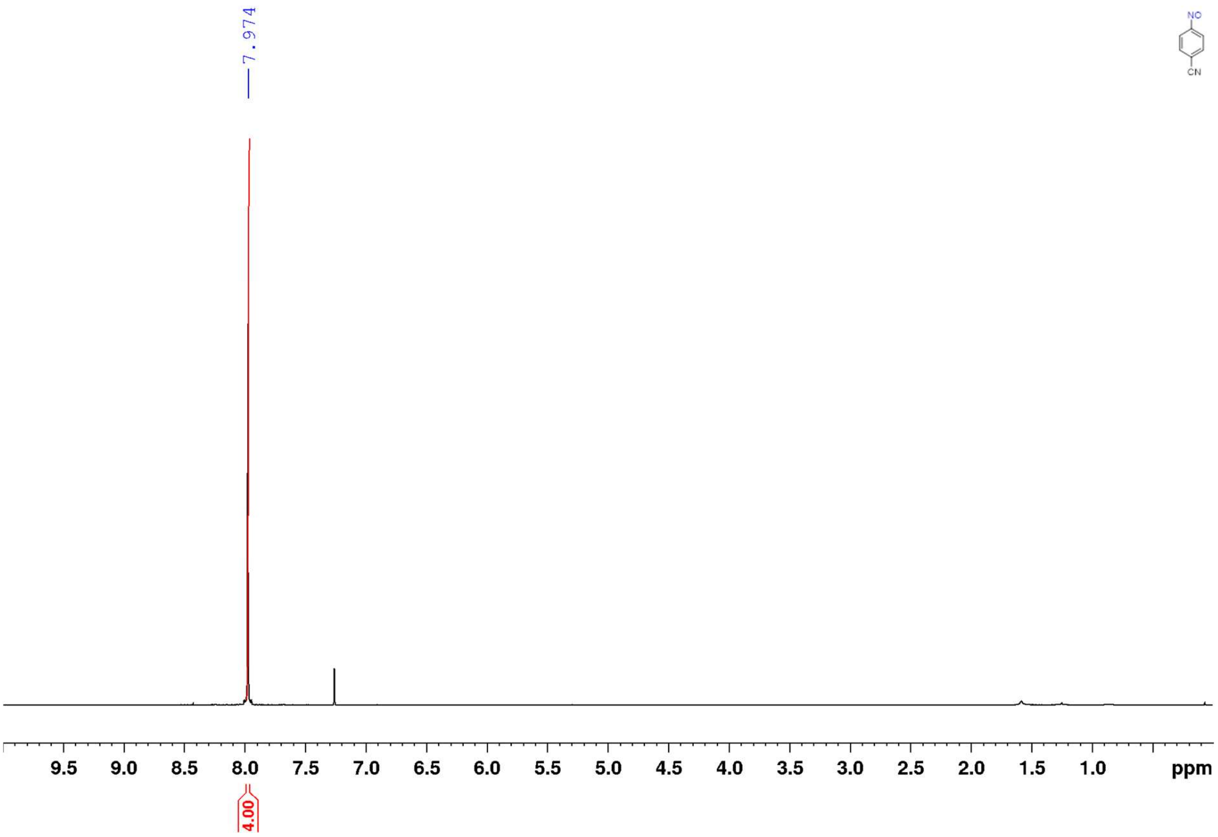

Figure S20. 9a, ^{13}C NMR (75MHz, $CDCl_3$)

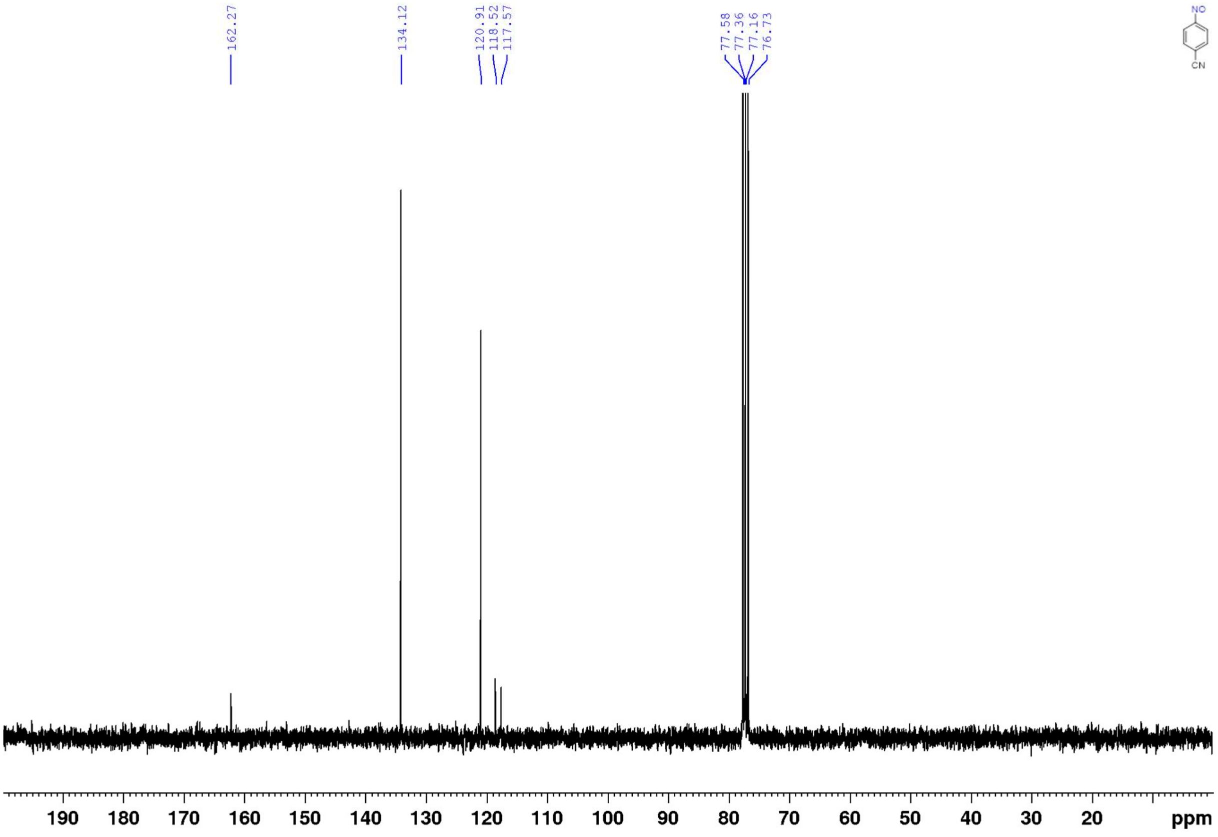

Figure S21. 10a, ^{1}H NMR (300MHz, $CDCl_3$)

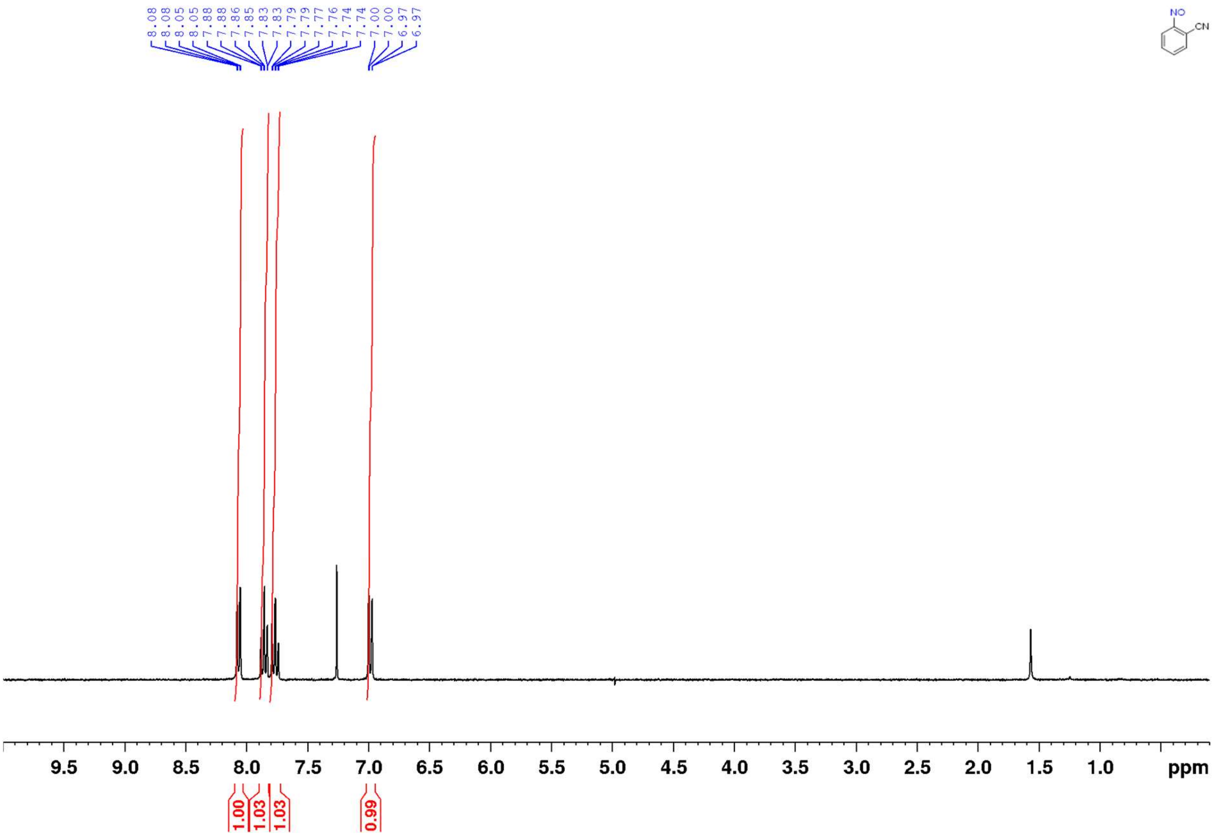

Figure S22. 10a, ^{13}C NMR (75MHz, $CDCl_3$)

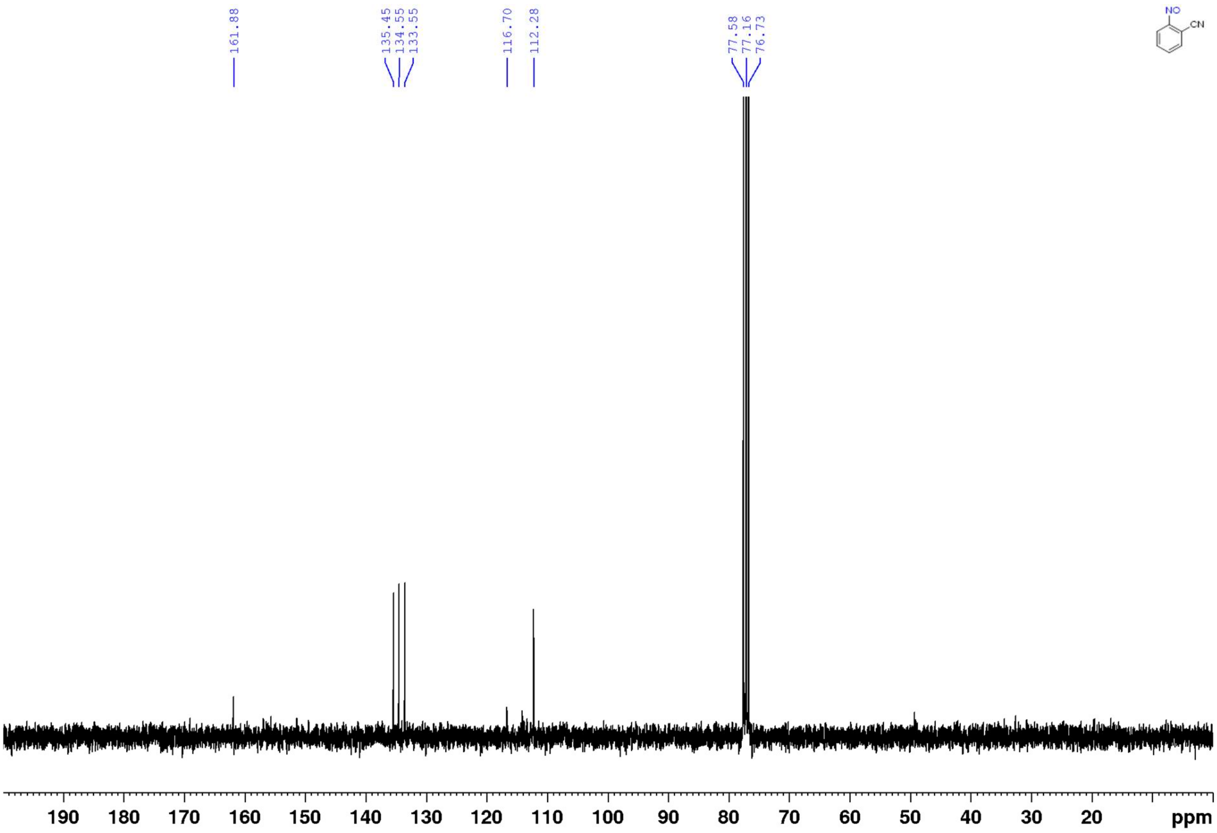

Figure S23. 11a, ^{1}H NMR (300MHz, $CDCl_3$)

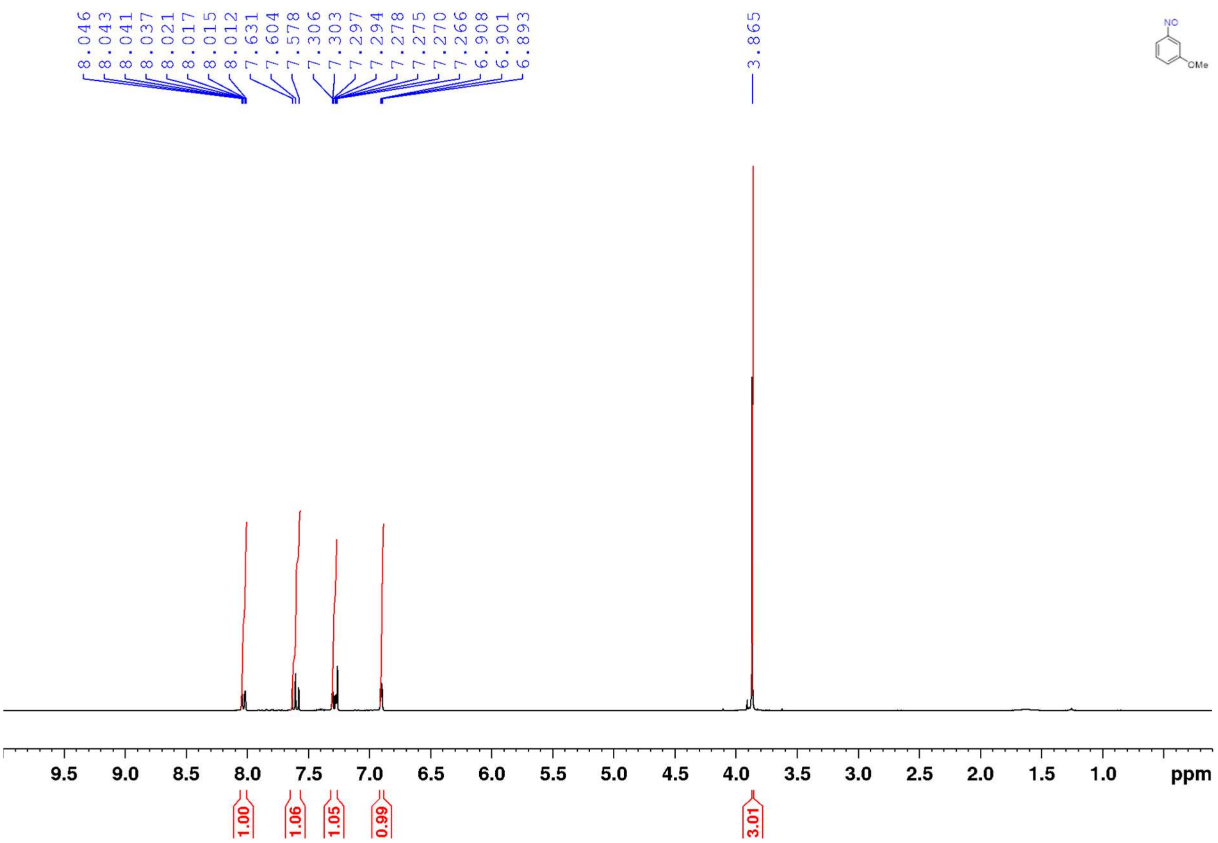

Figure S24. 11a, ^{13}C NMR (75MHz, $CDCl_3$)

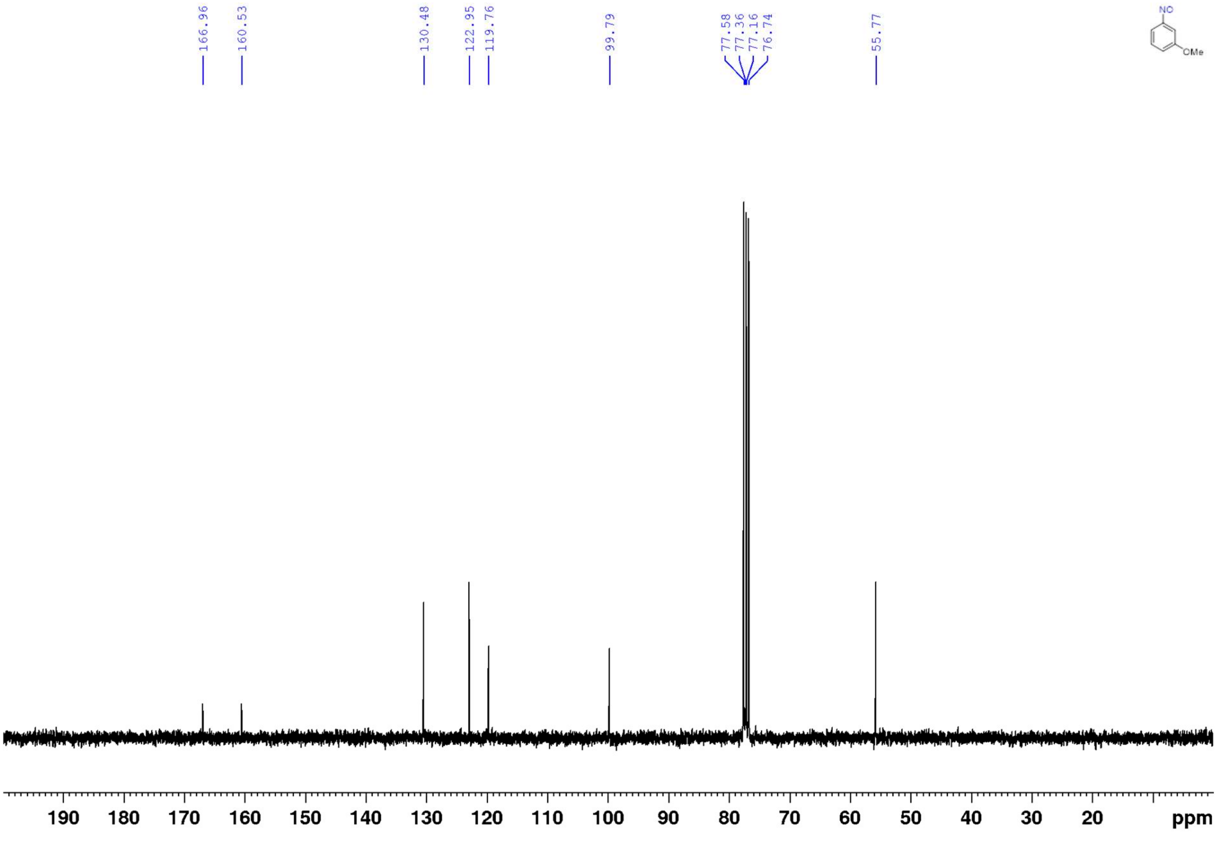

Figure S25. 12a, ^1H NMR (300MHz, $CDCl_3$)

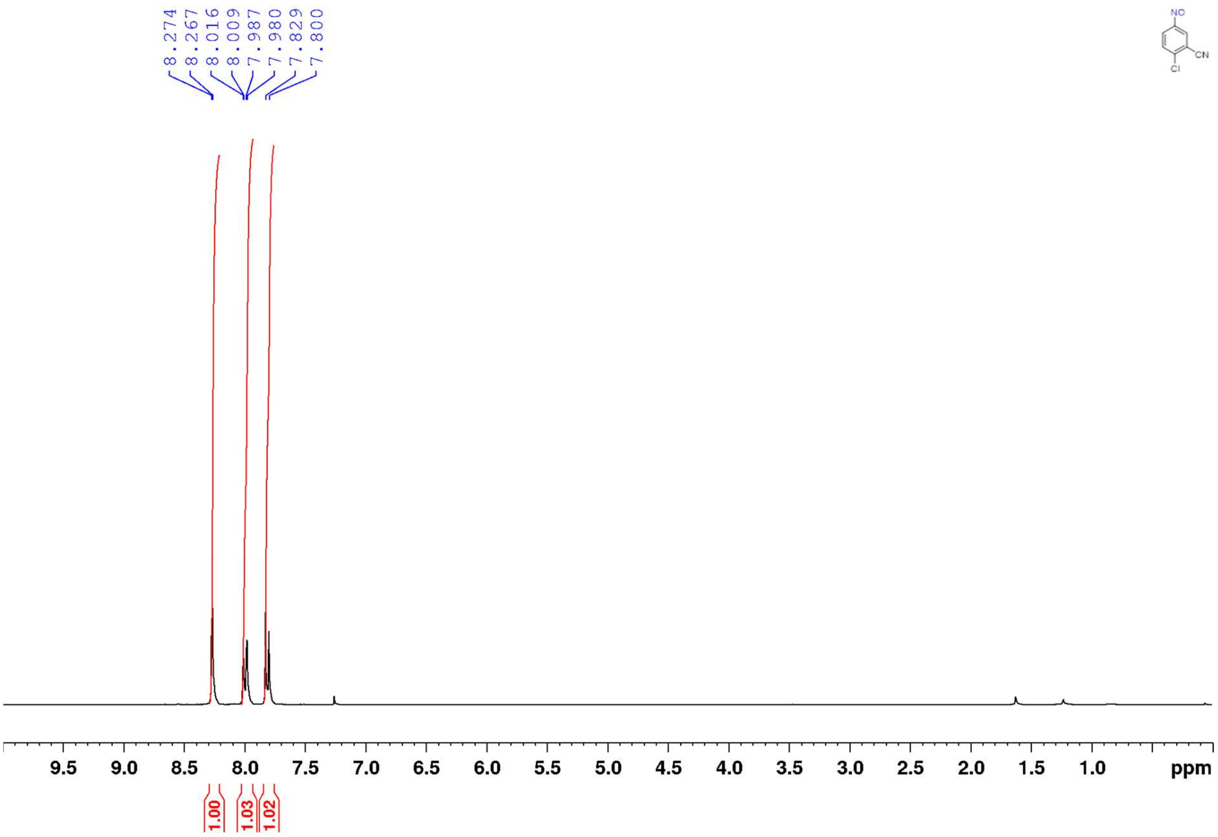

Figure S26. 12a, ^{13}C NMR (75MHz, $CDCl_3$)

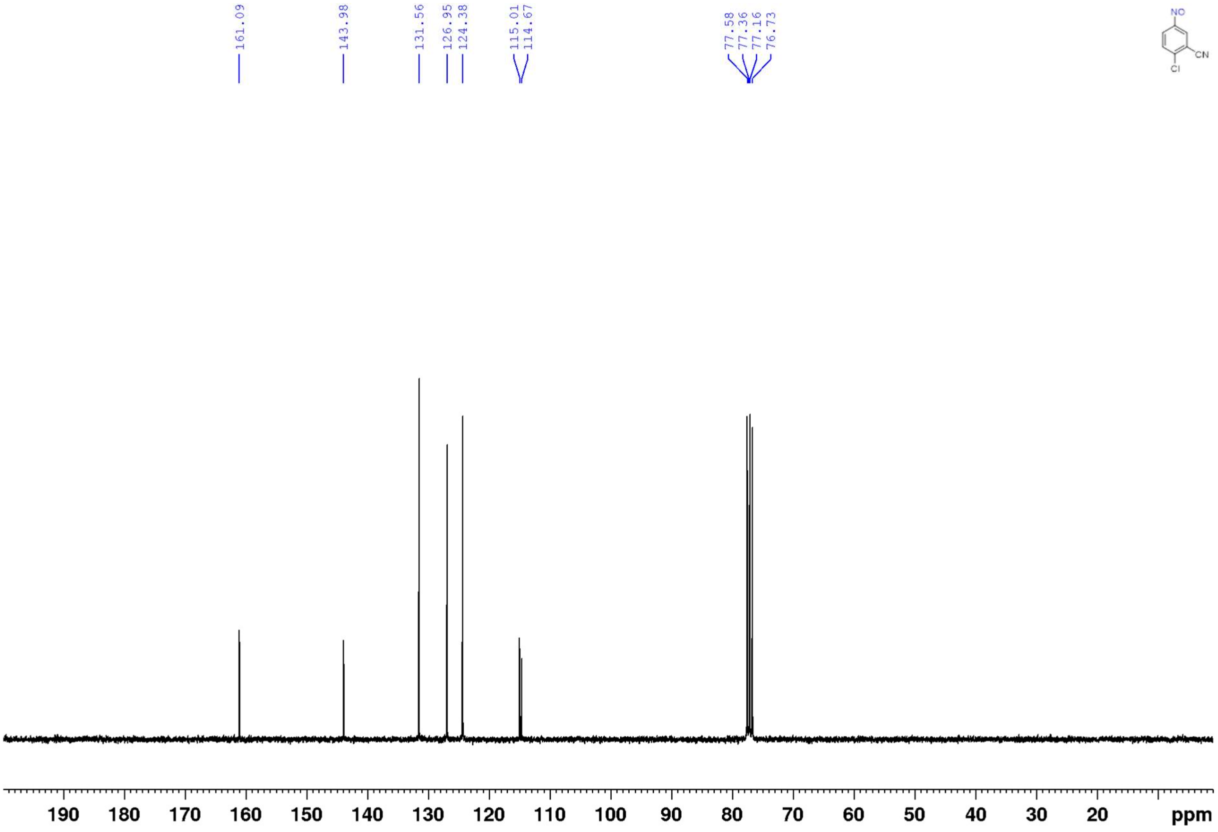

Figure S27. 13a, ^1H NMR (300MHz, $CDCl_3$)

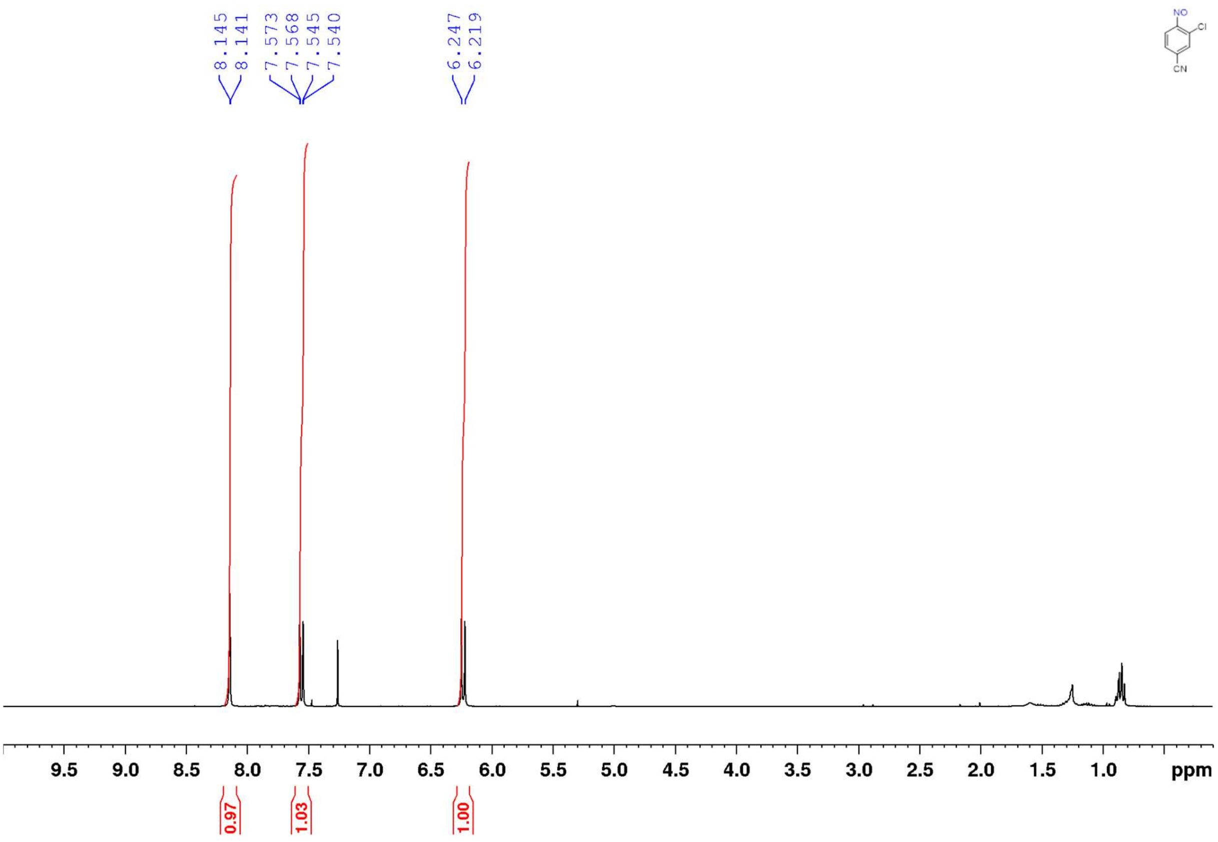

Figure S28. 13a, ^{13}C NMR (75MHz, $CDCl_3$)

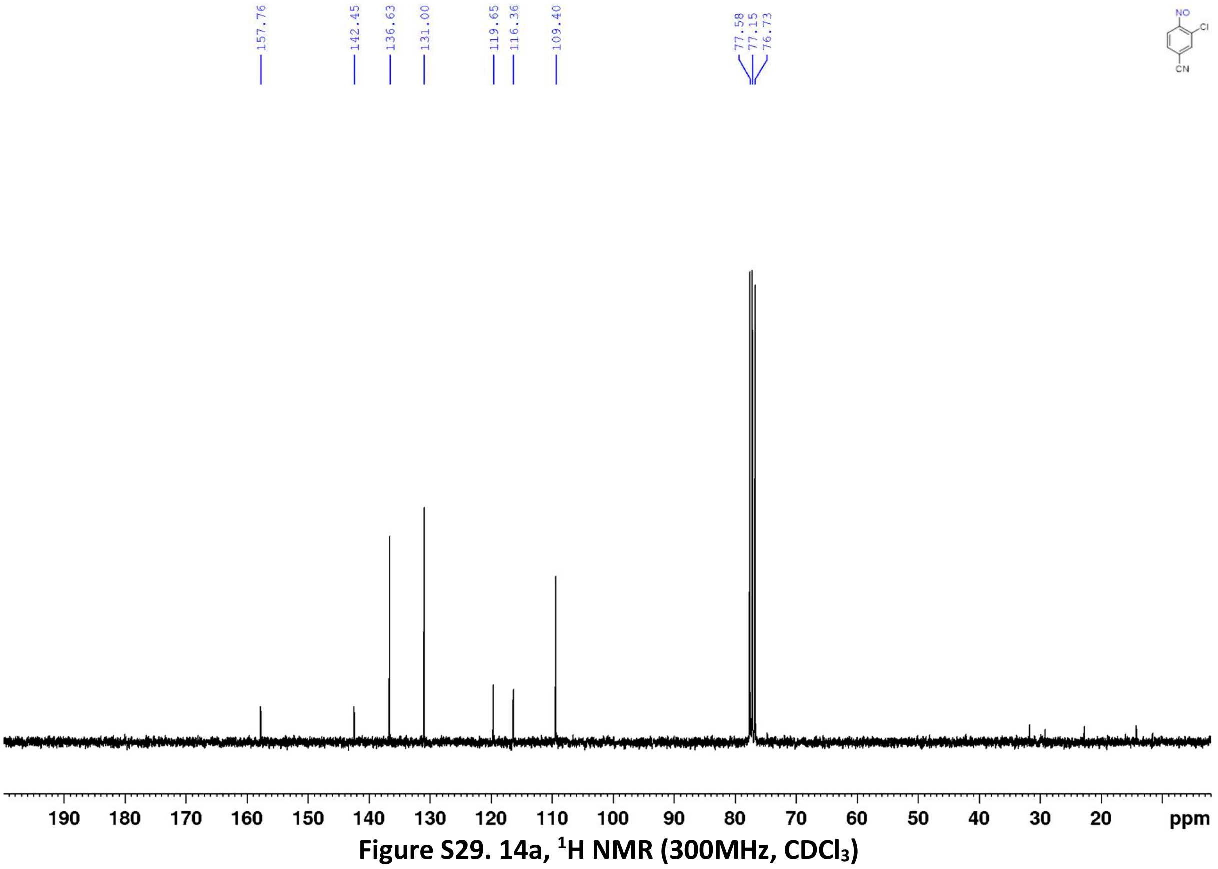

Figure S29. 14a, ^1H NMR (300MHz, $CDCl_3$)

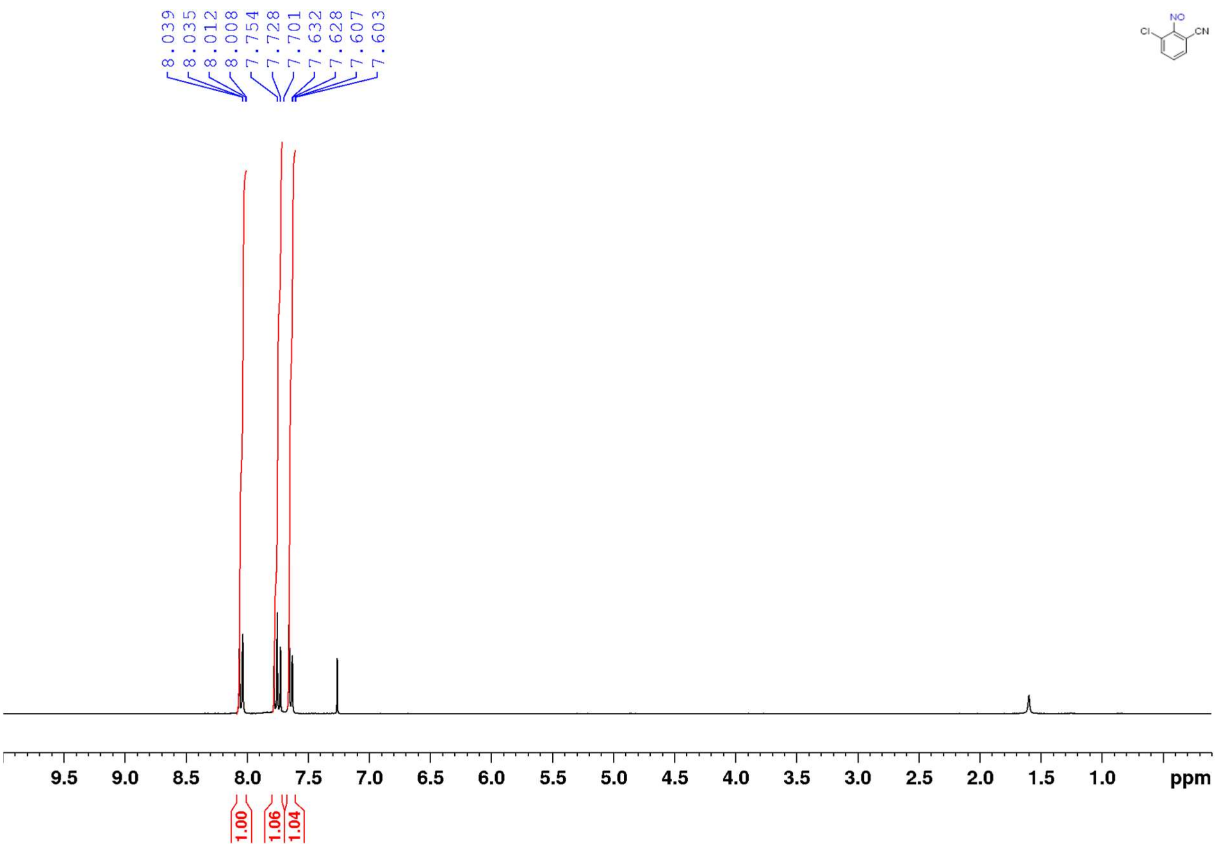

Figure S30. 14a, ^{13}C NMR (75MHz, $CDCl_3$)

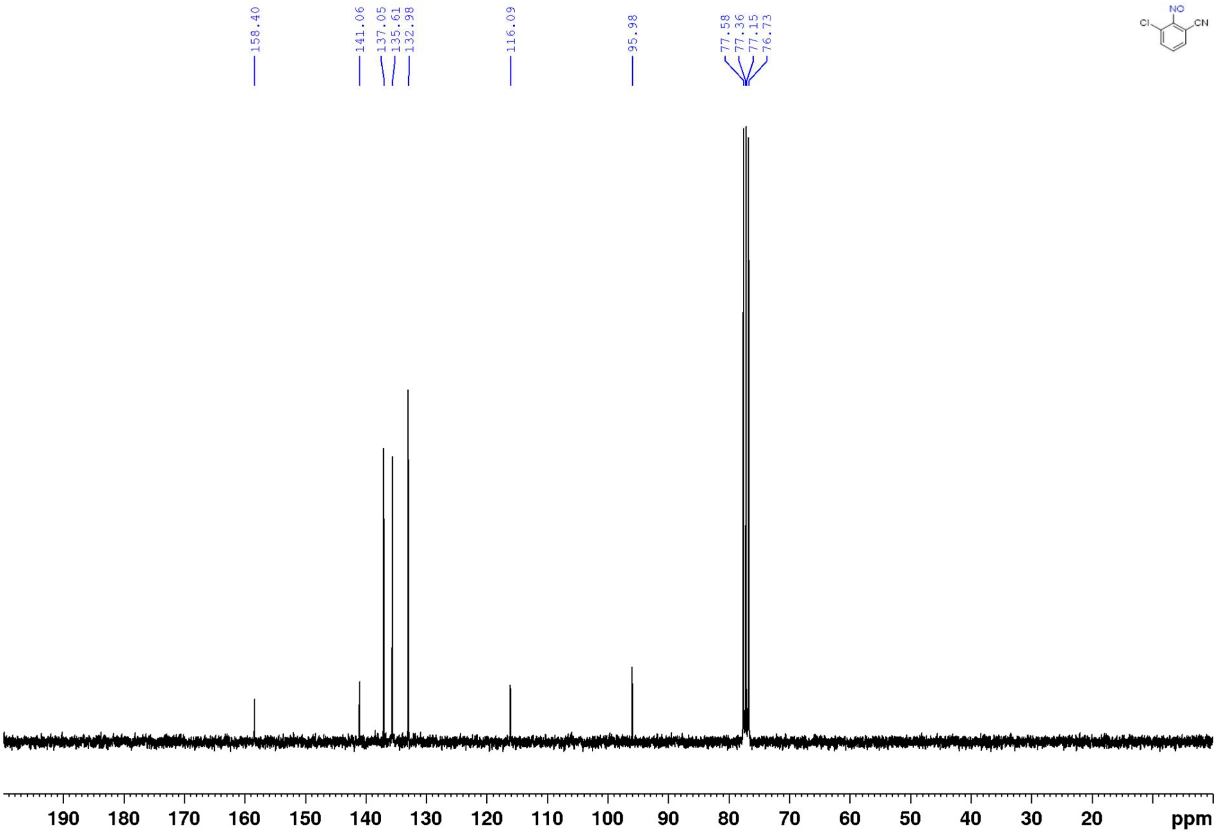

Figure S31. 15a, ^{1}H NMR (300MHz, $CDCl_3$)

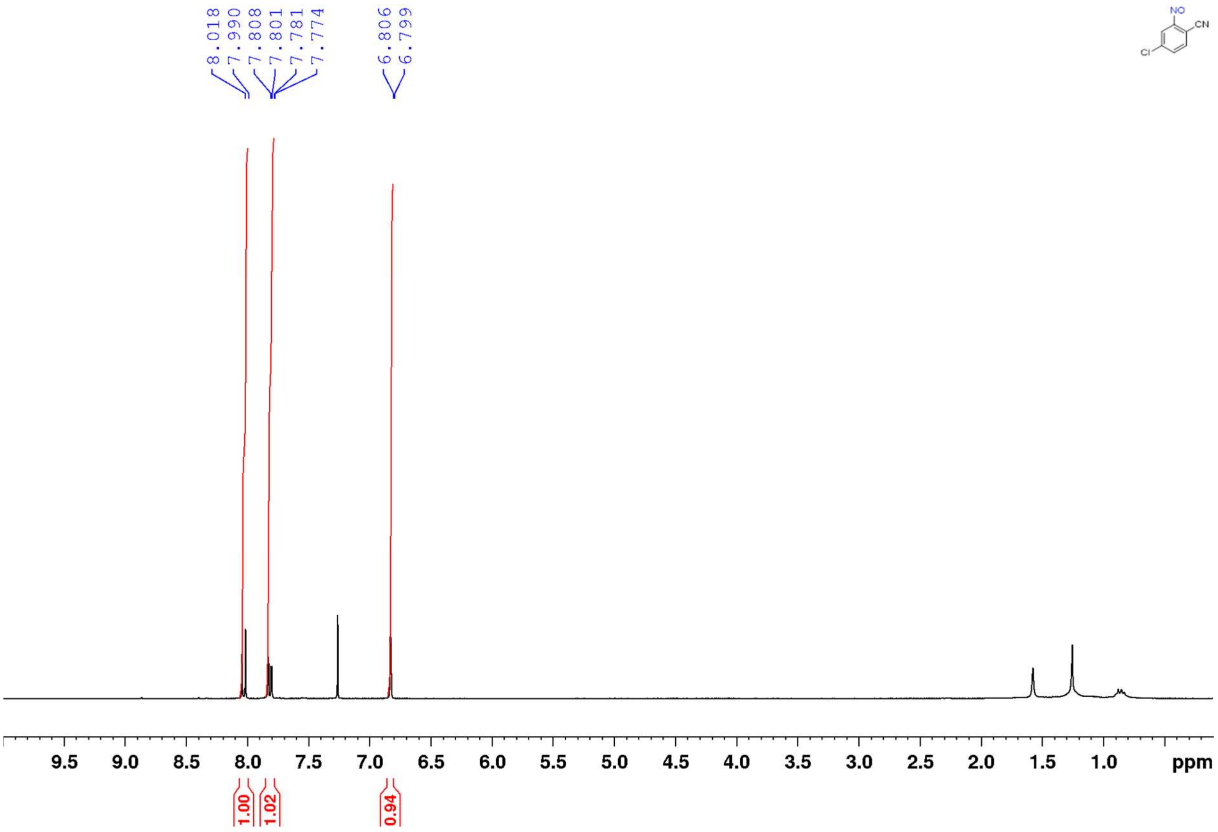

Figure S32. 15a, ^{13}C NMR (75MHz, $CDCl_3$)

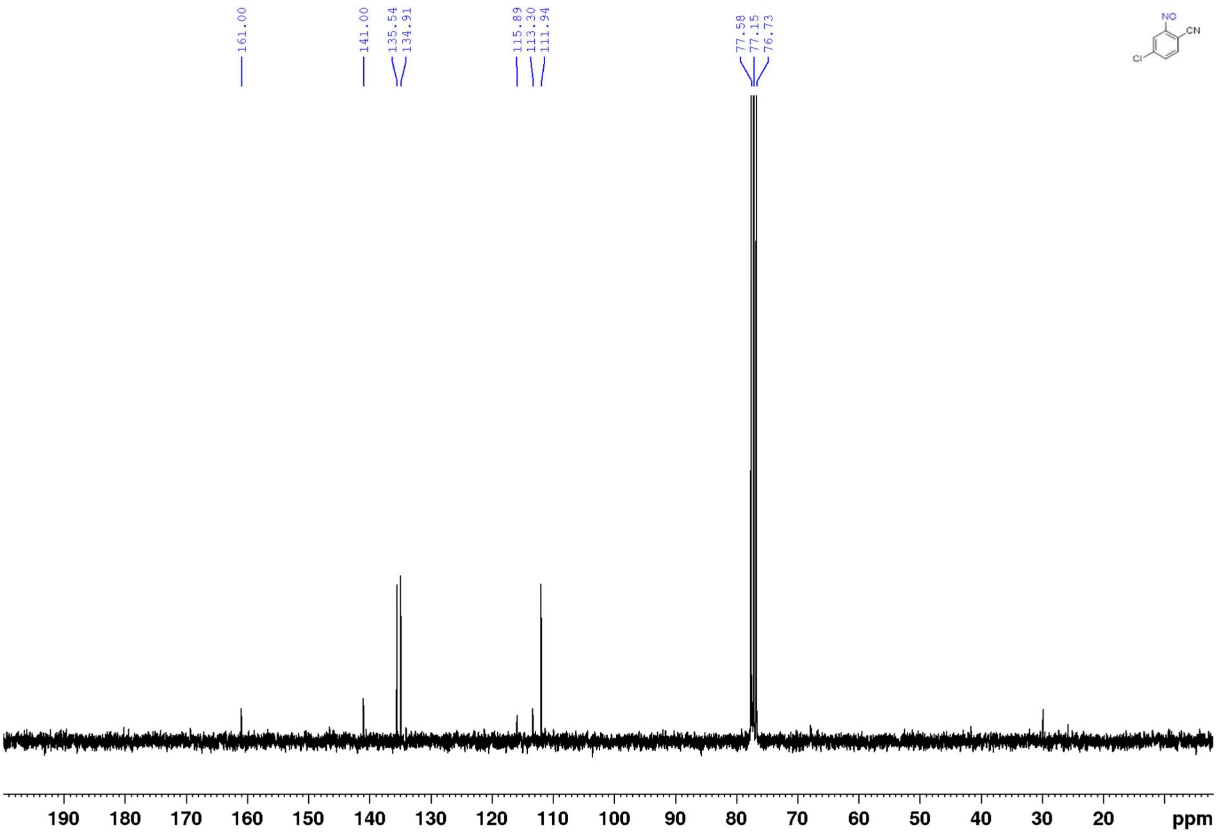

Figure S33. 16a, ^{1}H NMR (300MHz, $CDCl_3$)

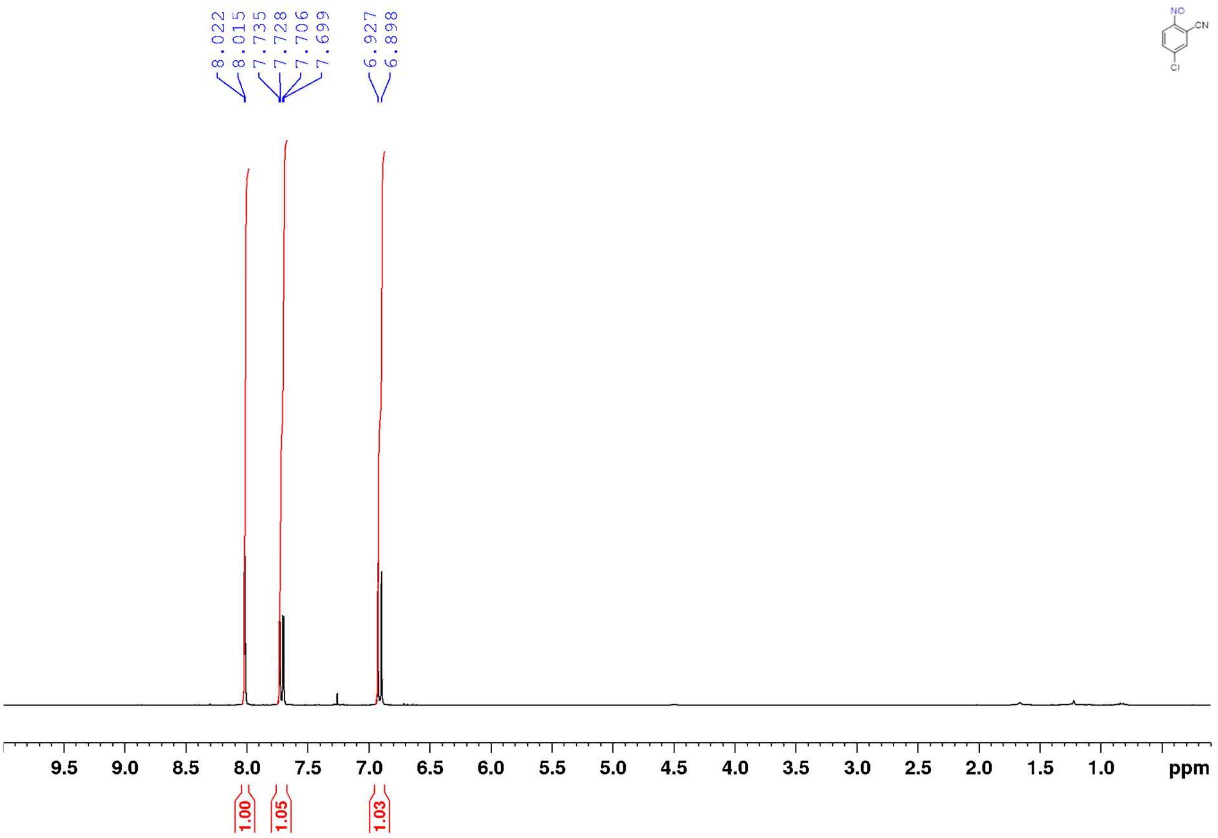

Figure S34. 16a, ^{13}C NMR (75MHz, $CDCl_3$)

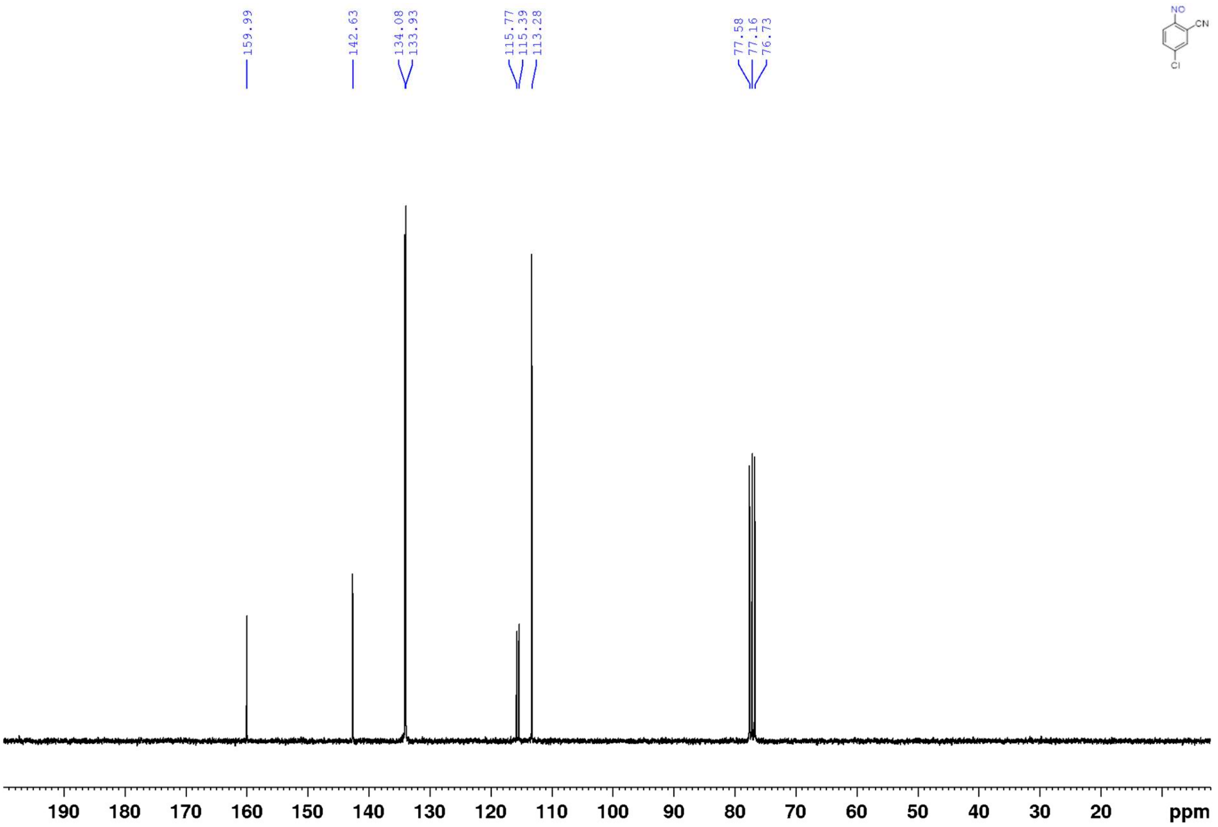

Figure S35. 17a, ^{1}H NMR (300MHz, $CDCl_3$)

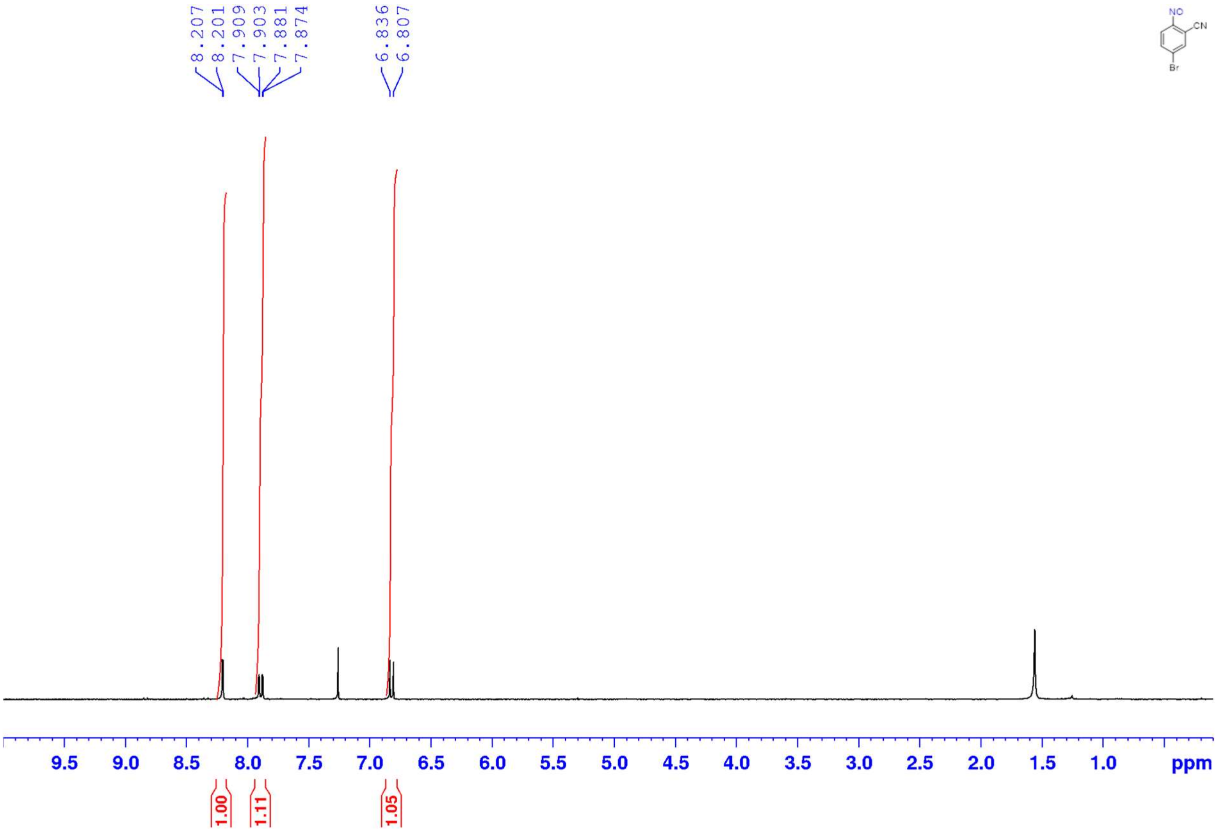

Figure S36. 17a, ^{13}C NMR (75MHz, $CDCl_3$)

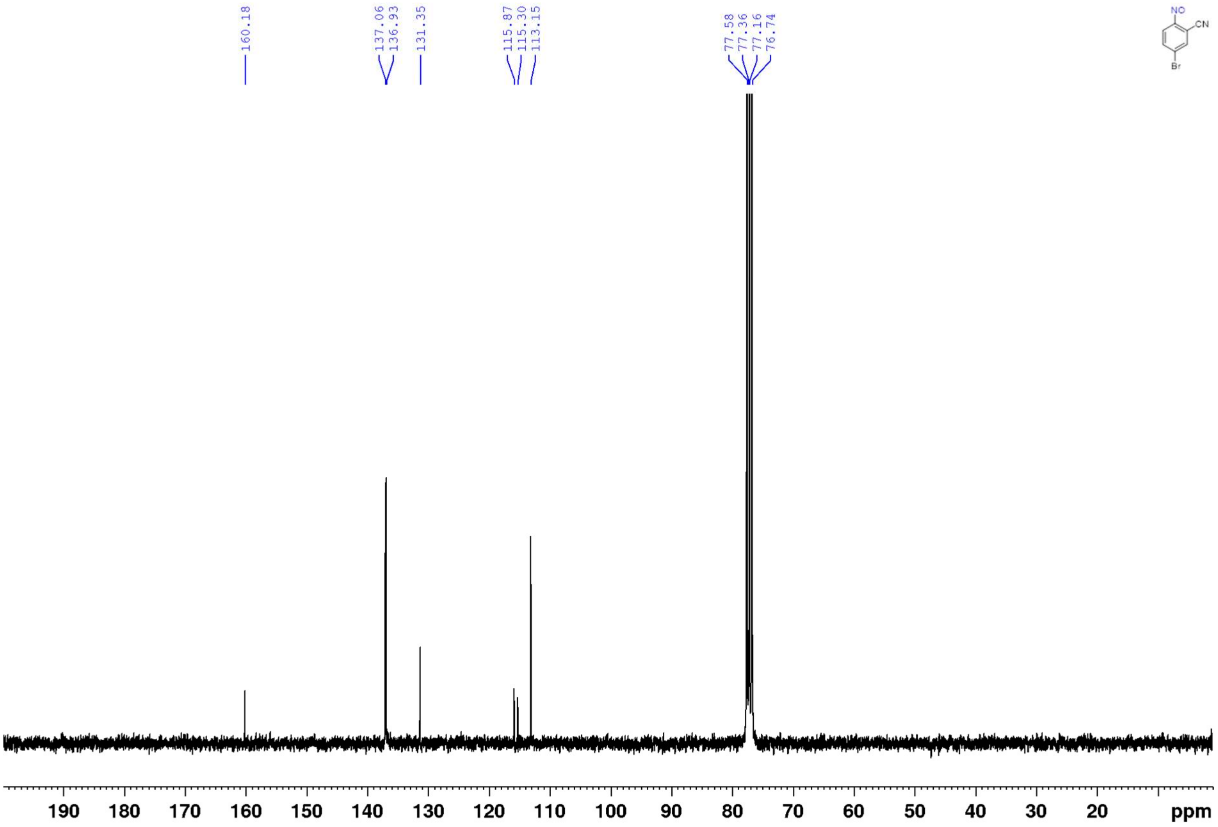

Figure S37. 18a, ^{1}H NMR (300MHz, $CDCl_3$)

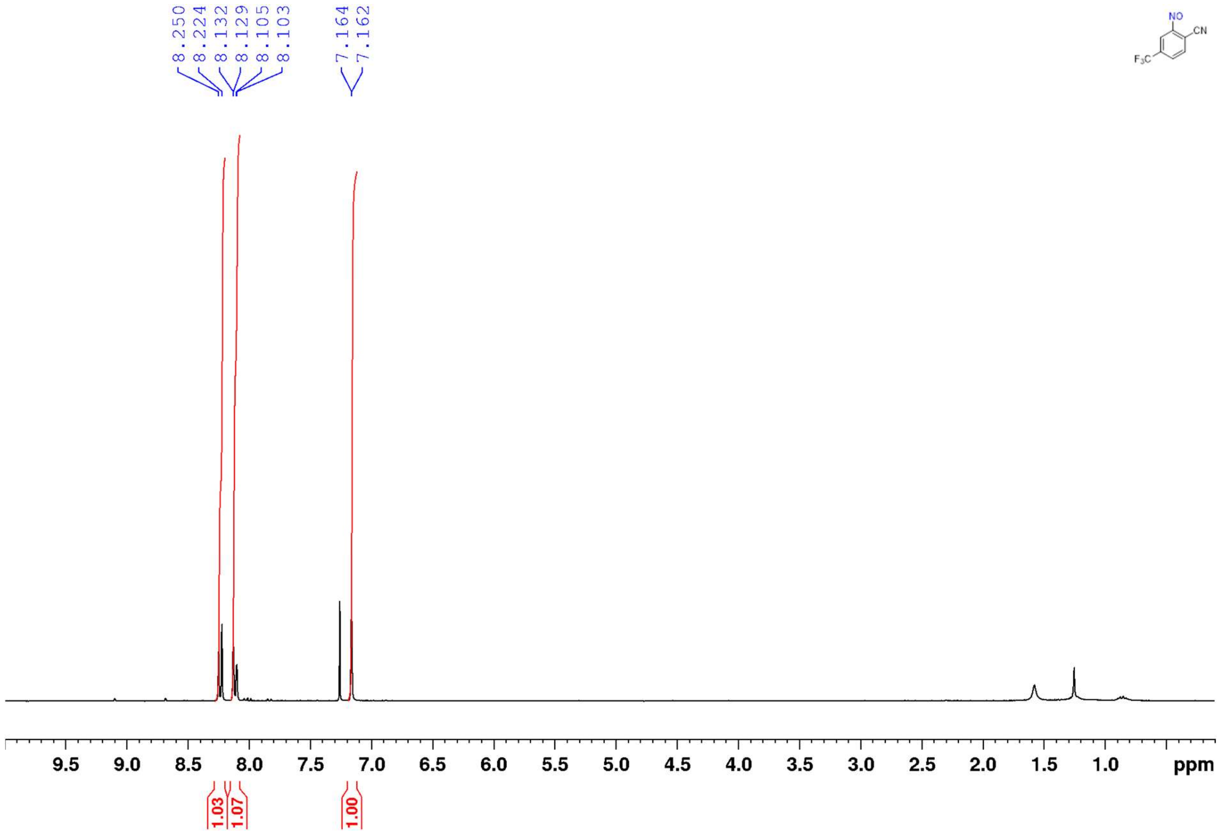

Figure S38. 18a, ^{13}C NMR (75MHz, $CDCl_3$)

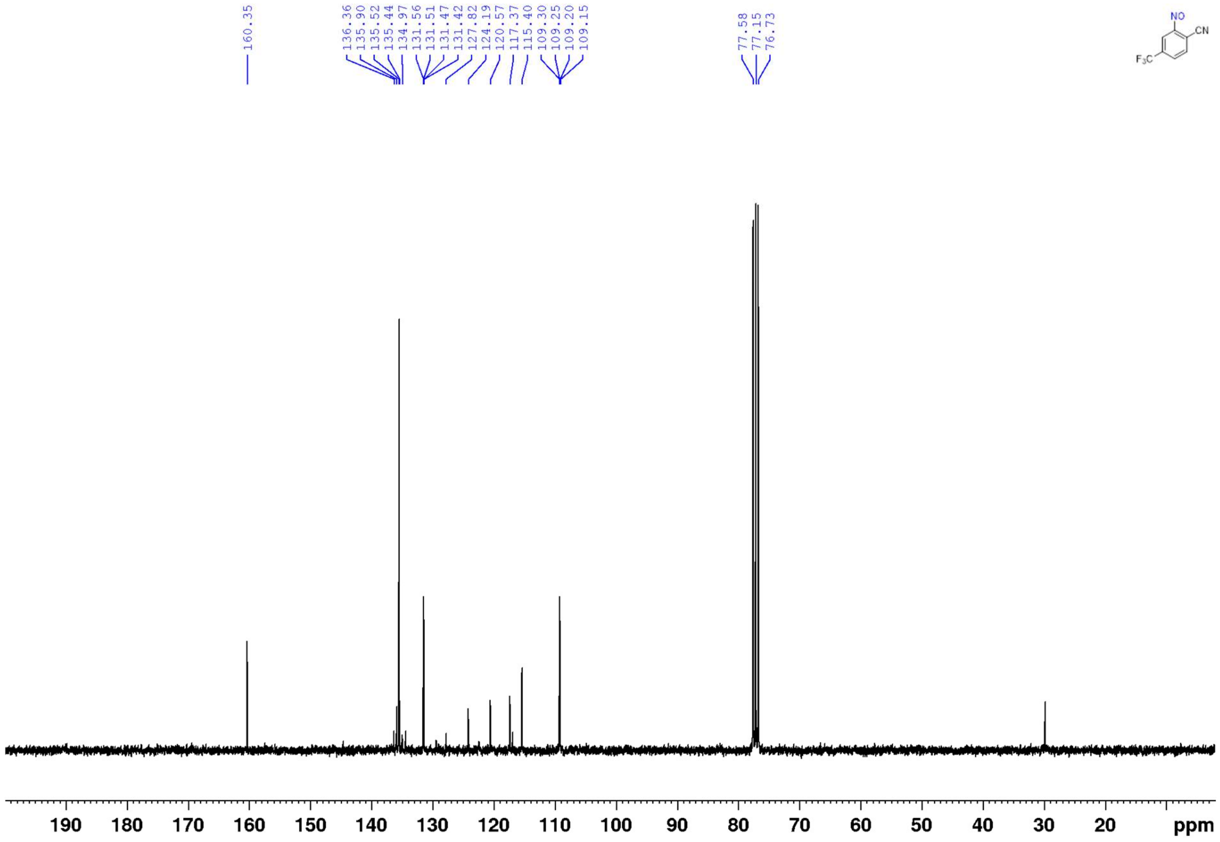

Figure S39. 19a, ^{1}H NMR (300MHz, $CDCl_3$)

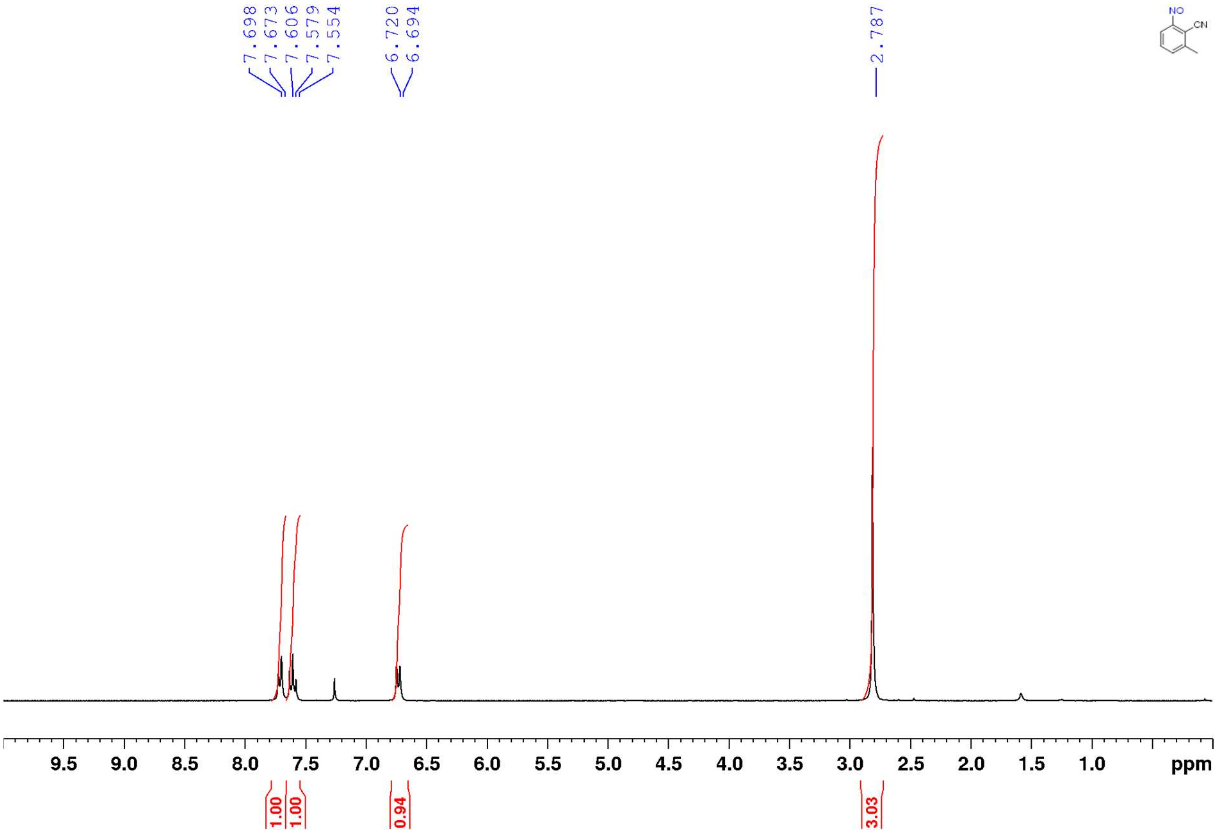

Figure S40. 19a, ^{13}C NMR (75MHz, $CDCl_3$)

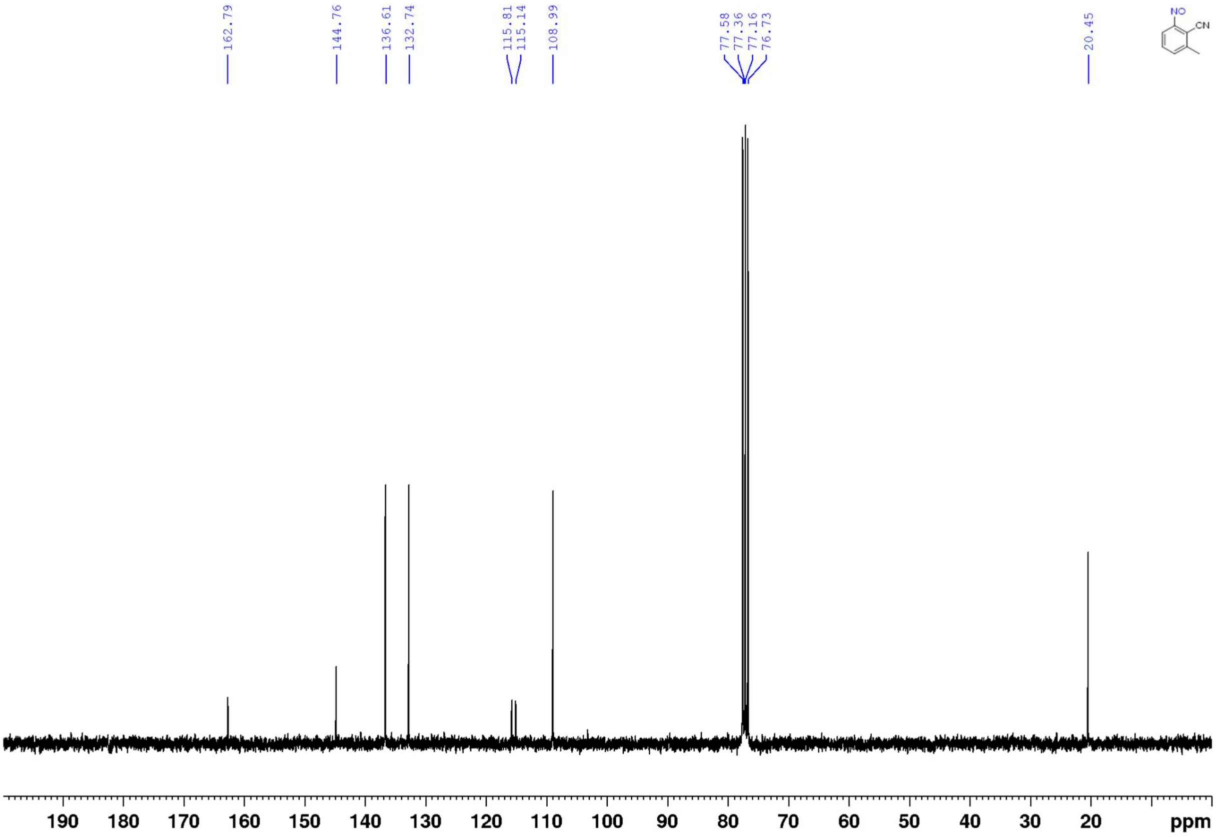

Figure S41. 20a, ^{1}H NMR (300MHz, $CDCl_3$)

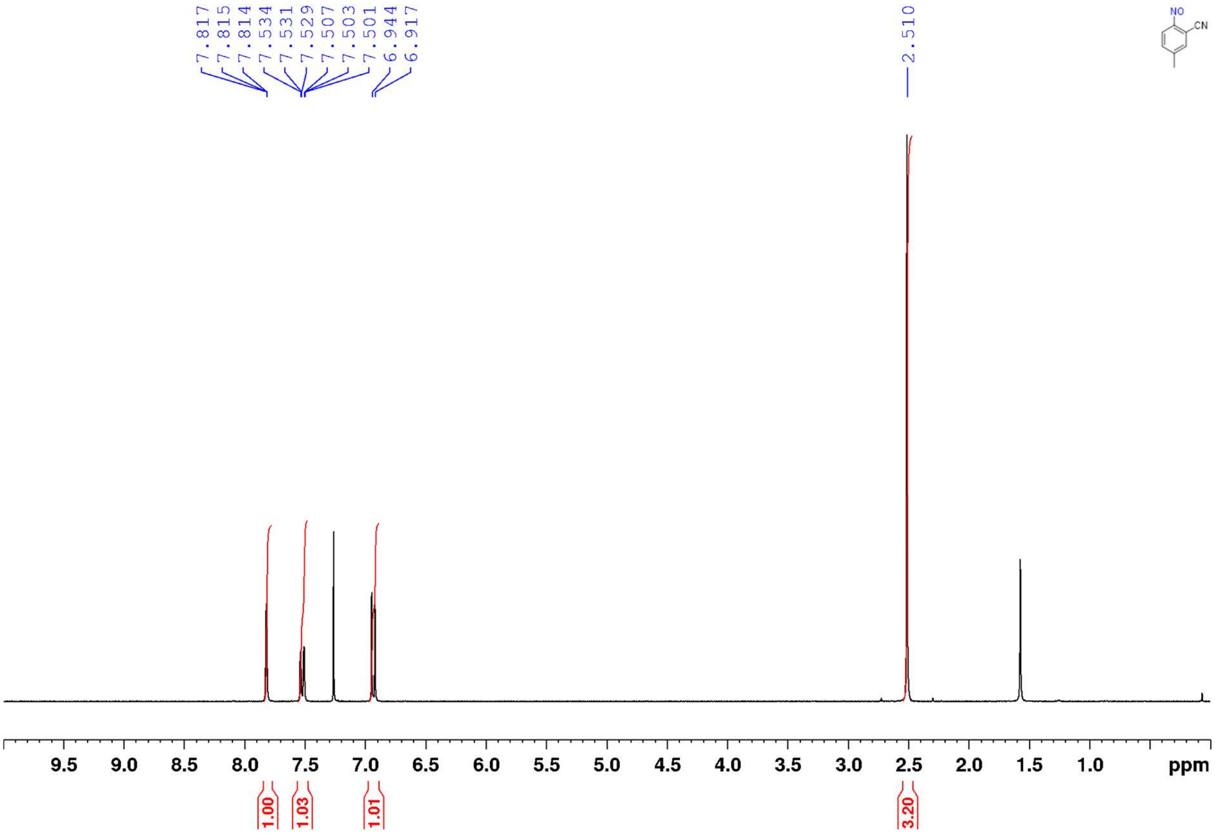

Figure S42. 20a, ^{13}C NMR (75MHz, $CDCl_3$)

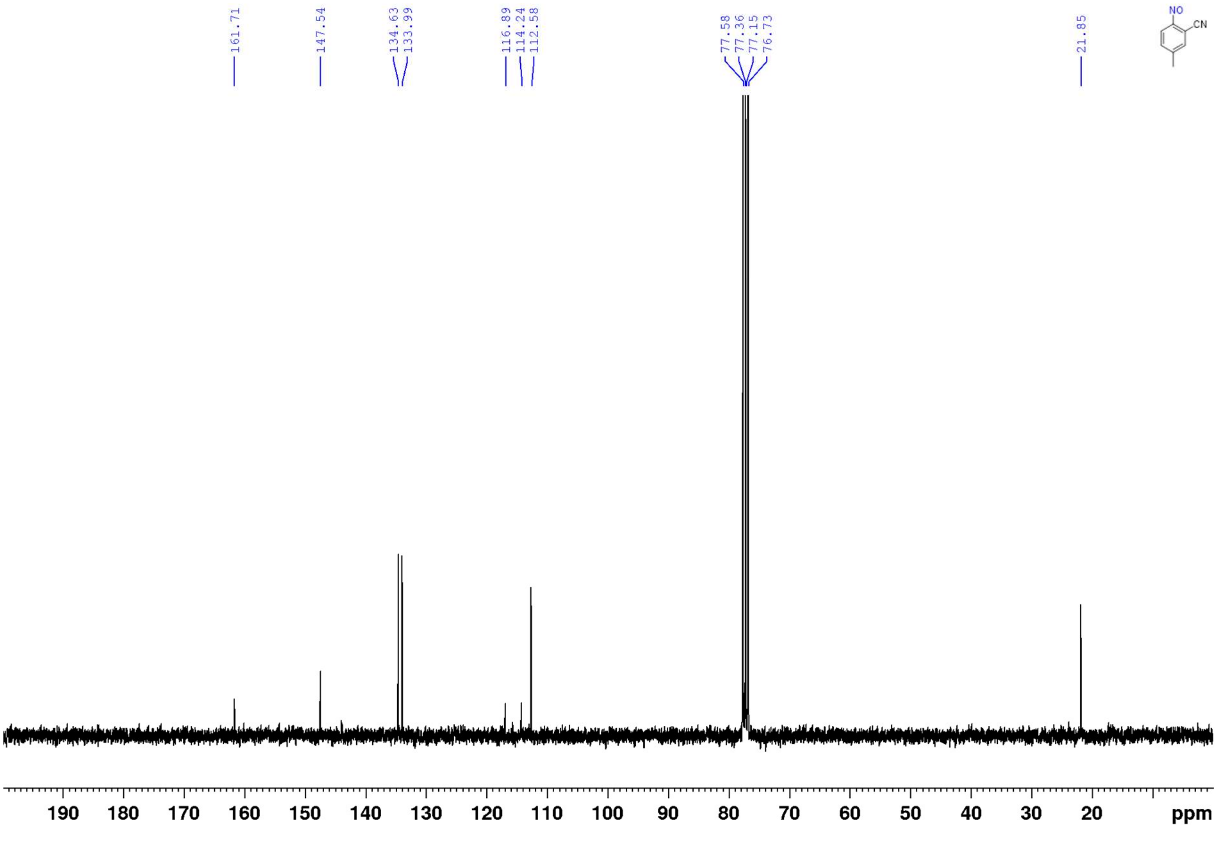

Figure S43. 21a, ^{1}H NMR (300MHz, $CDCl_3$)

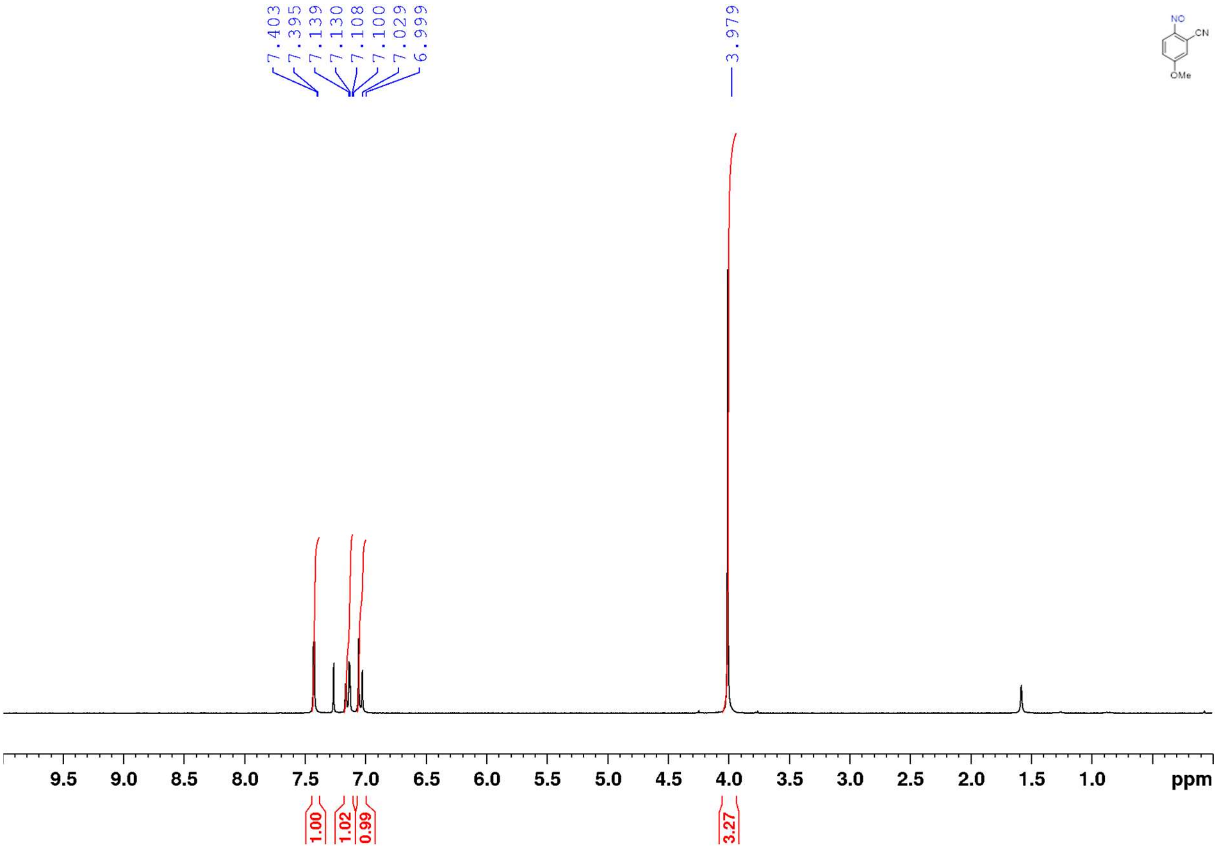

Figure S44. 21a, ^{13}C NMR (75MHz, $CDCl_3$)

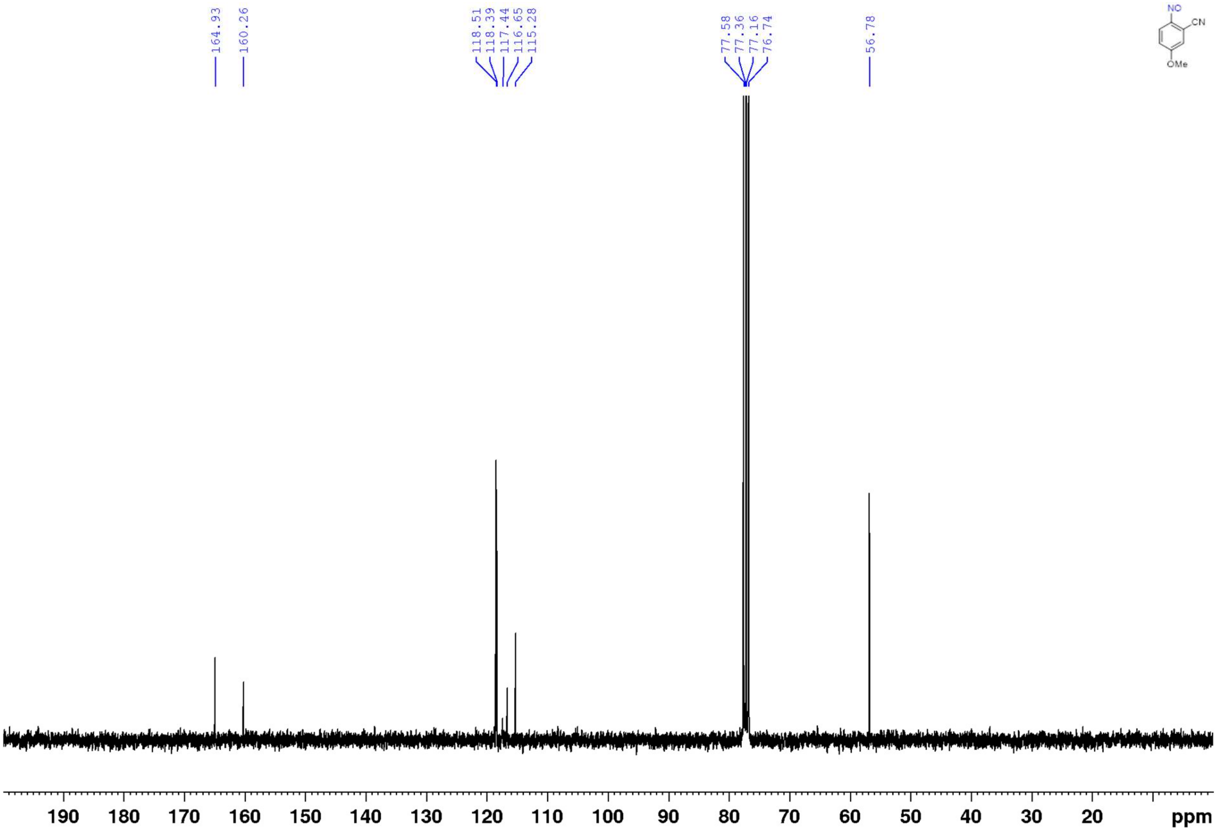

Figure S45. 22a, ^{1}H NMR (300MHz, $CDCl_3$)

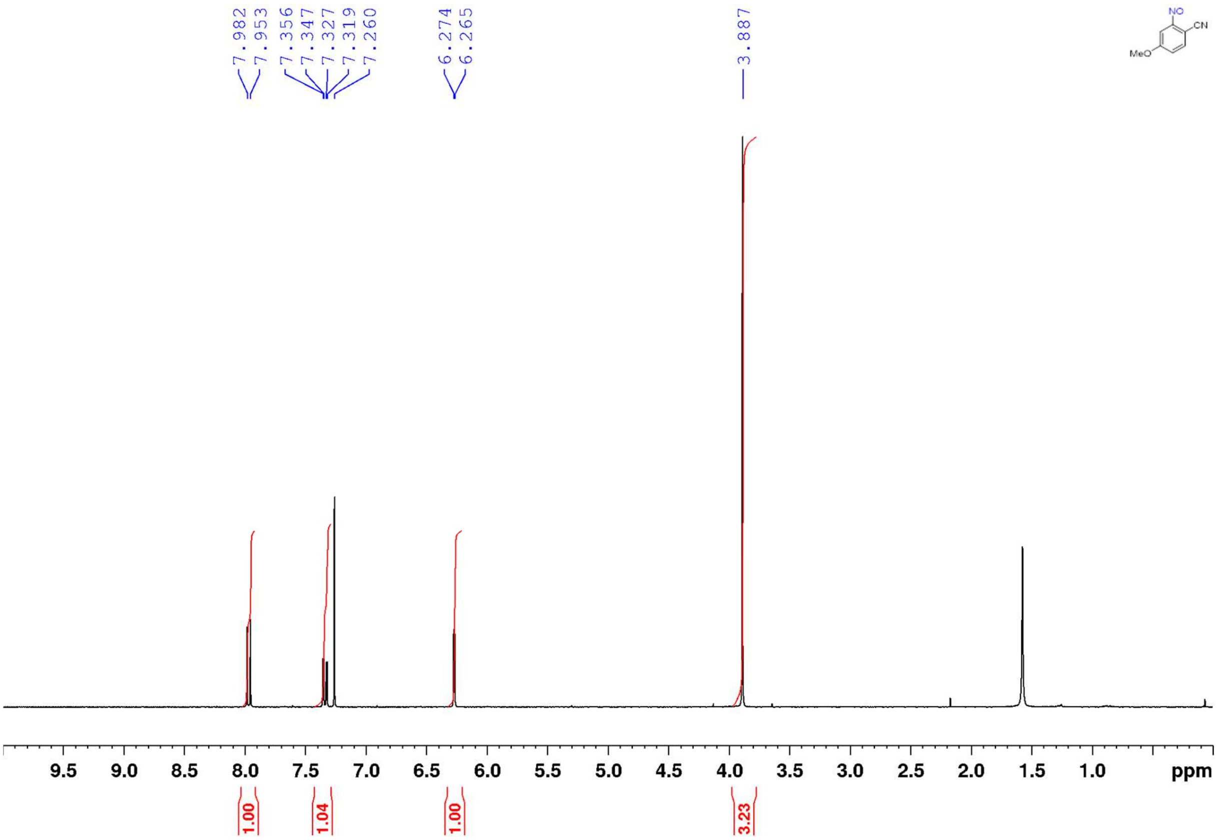

Figure S46. 22a, ^{13}C NMR (75MHz, $CDCl_3$)

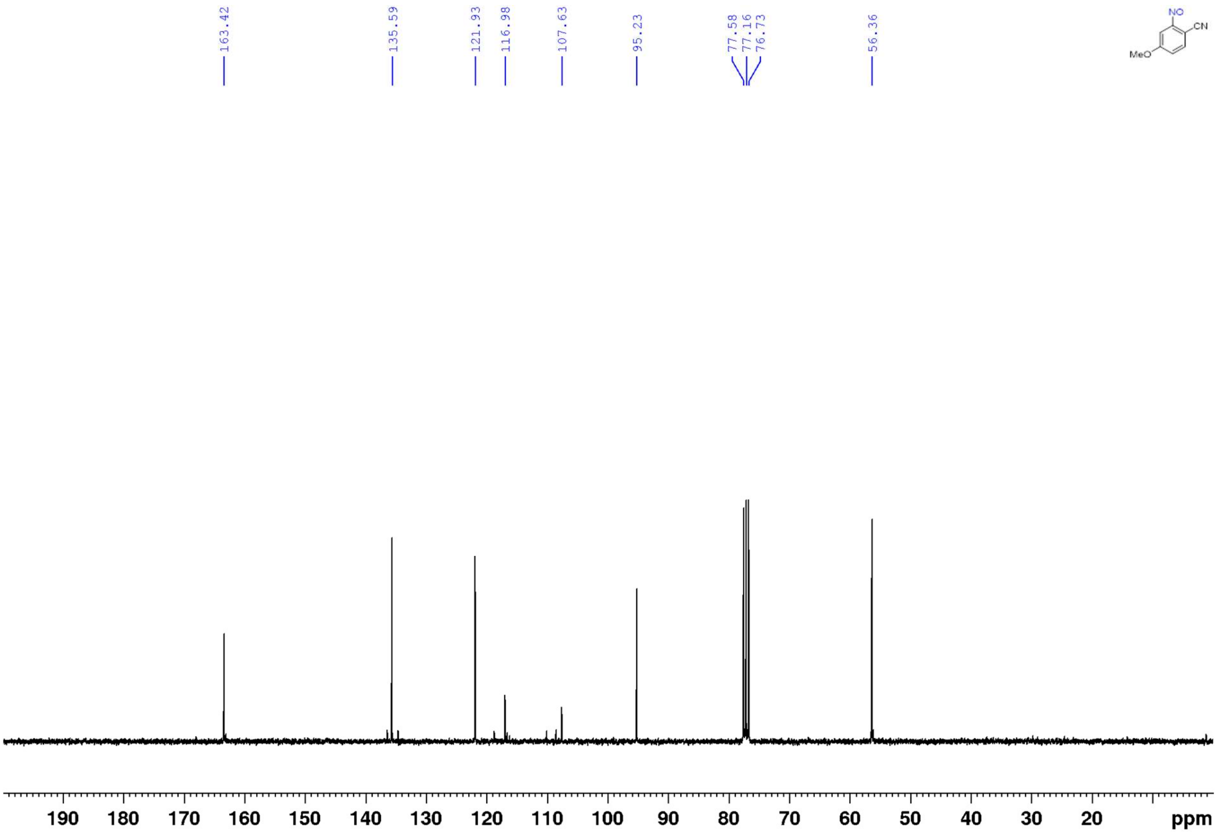

Figure S47. 24a, ^{1}H NMR (300MHz, $CDCl_3$)

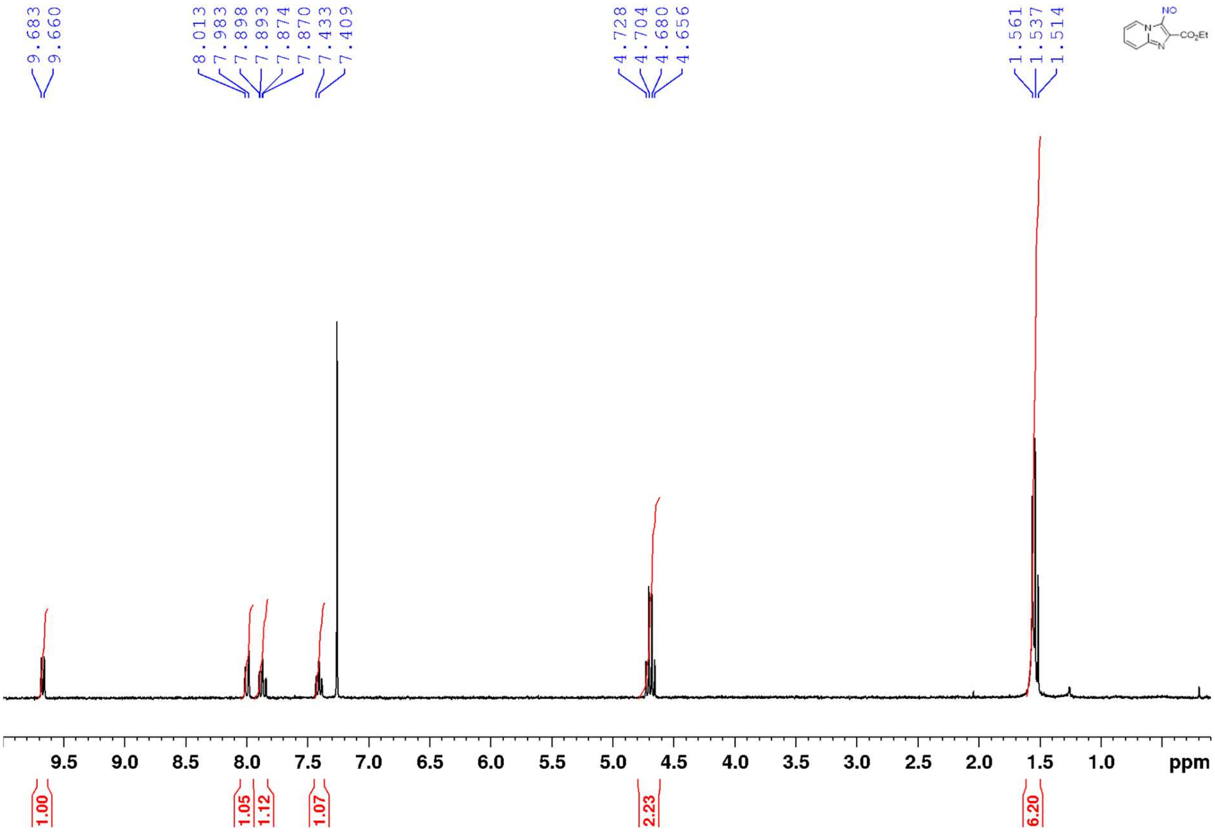

Figure S48. 24a, ^{13}C NMR (75MHz, $CDCl_3$)

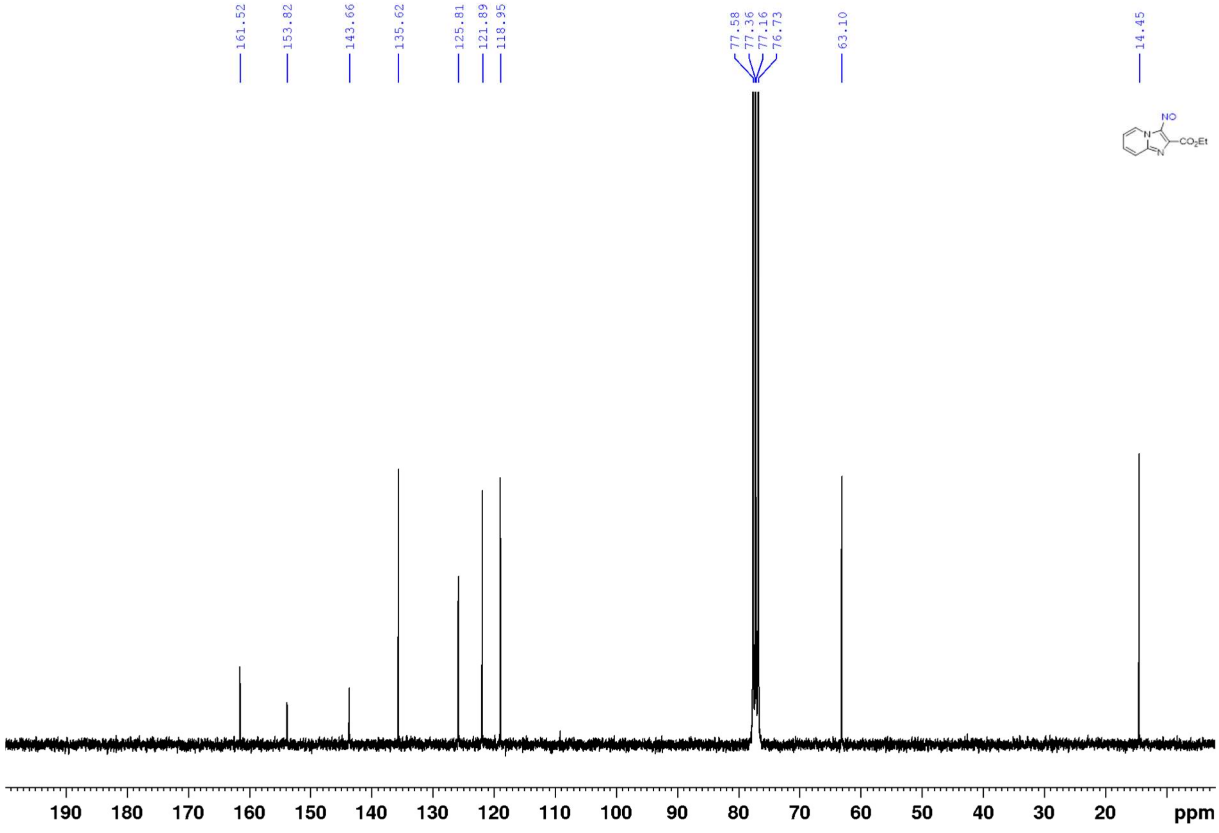

Figure S49. 25a, ^{1}H NMR (300MHz, $CDCl_3$)

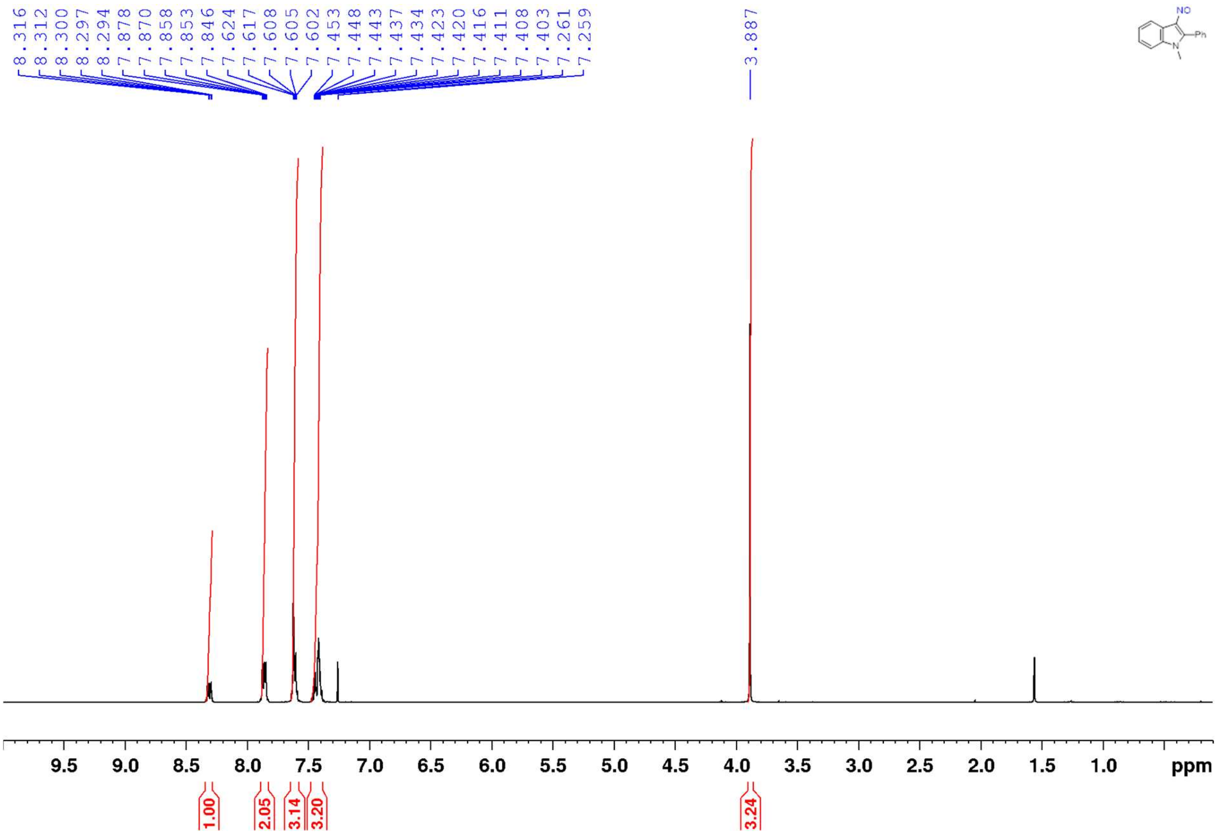

Figure S50. 25a, ^{13}C NMR (75MHz, $CDCl_3$)

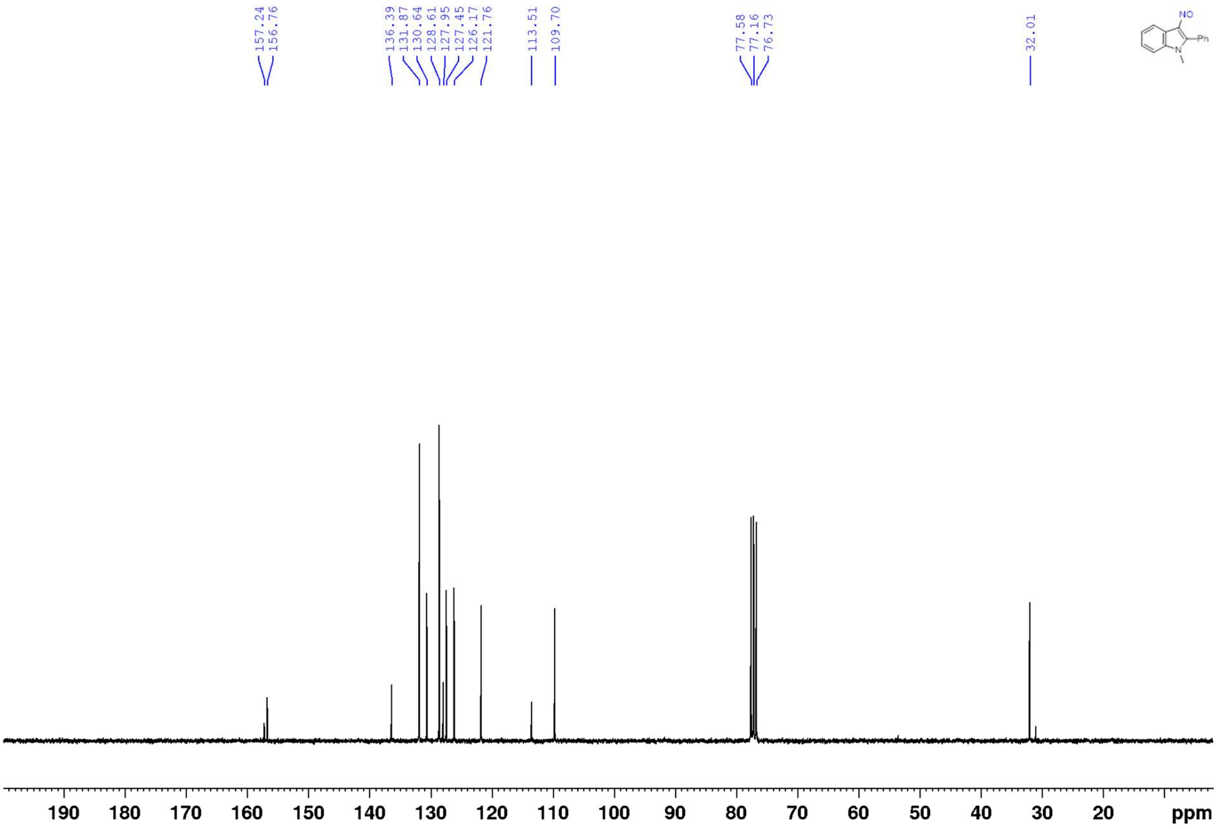

6. General procedure for the screening of reaction conditions for ^{18}F-labeling:

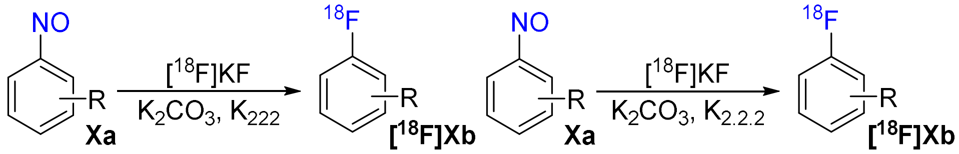

^{18}F-Fluorination was tested using our recently described microliter scale radiofluorination approach in HPLC vials. In brief, Sep-Pak AccellPlus QMA Carbonate Plus Light Cartridge (P.N. 186004540) was preconditioned by sequential elution with water (10 mL), 1 M $NaHCO_3$ (10 mL), and water (10 mL). [^{18}F]fluoride (100-300 MBq) was adsorbed on the QMA light, washed with 1 mL of MeCN, and eluted using a either a solution of $K_{2.2.2}$ (60 µmol, 22.59 mg) and K_2CO_3 (30 µmol) in 1.02 mL MeCN/water 98/2 denoted as 'HYFE normal', or a solution of $K_{2.2.2}$ (60 µmol, 22.59 mg) and K_2CO_3 (7.5 µmol) in 1.035 mL MeCN/water 97/3 denoted as 'HYFE ¼ 3% H_2O'. Elution efficiencies in both cases exceeds usually 90%. Aliquots of 50 µL for each reaction were transferred into an HPLC vials and in parallel dried for 3 min at 90°C with a gentle stream of helium. Then, 50 µL aliquots of the precursors were added at a concentration of 5 mg/mL in the given solvent and heated for 15 min at the given temperature. Finally, the reactions were quenched using 150 µL MeCN/H_2O 50/50 and analysed by radio-HPLC (System 2) or radio-TLC as indicated. For product identification, non-radioactive authentic reference was analysed by analytical radio-HPLC (system 2) alone or as dilution of stock solution (1 µL, 10 mg/mL in DMSO) in 100 µL of the quenched reaction mixture.

7. General procedure for isolation of ^{18}F-labeled products including determination of RCY and product identification

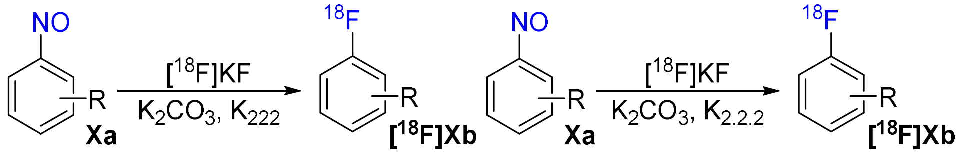

^{18}F-Fluorination was performed using our recently described microliter scale radiofluorination approach in HPLC vials. In brief, Sep-Pak AccellPlus QMA Carbonate Plus Light Cartridge (P.N. 186004540) was preconditioned by sequential elution with water (10 mL), 1 M $NaHCO_3$ (10 mL), and water (10 mL). [^{18}F]Fluoride (300-1000 MBq) was adsorbed on a QMA light, washed with 1 mL of MeCN, and eluted using either a solution of K_{222} (60 µmol, 22.59 mg) and K_2CO_3 (30 µmol) in 1.02 mL MeCN/water 98/2 denoted as 'normal', or a solution of K_{222} (60 µmol, 22.59 mg) and K_2CO_3 (7.5 µmol) in 1.035 mL MeCN/water 97/3 denoted as '¼'. An aliquot of 50 µL for each reaction was transferred into an HPLC vial, activity was measured (A_{start}) and in dried for 3 min at 90°C with a gentle stream of helium. Then, a 50 µL aliquot of the precursor was added at a concentration of 5 mg/mL in the given solvent and heated for 15 min at the given temperature. Finally, the reactions were quenched using 1400 µL MeCN/H_2O 50/50 and purified by semipreparative radio-HPLC (System 3). ^{18}F-labeled product was collected and activity of the fraction was determined ($A_{product}$) to determine decay-corrected radiochemical yield with reference to A_{start}. For product identification, fraction was analysed without and with standard-addition of the non-radioactive authentic reference (1 µL 10 mg/mL stock solution in DMSO diluted in 100 µL of the collected fraction) using analytical radio-HPLC (system 1).

8. Analytical data of radiosyntheses

Radiolabeling of compound 1a

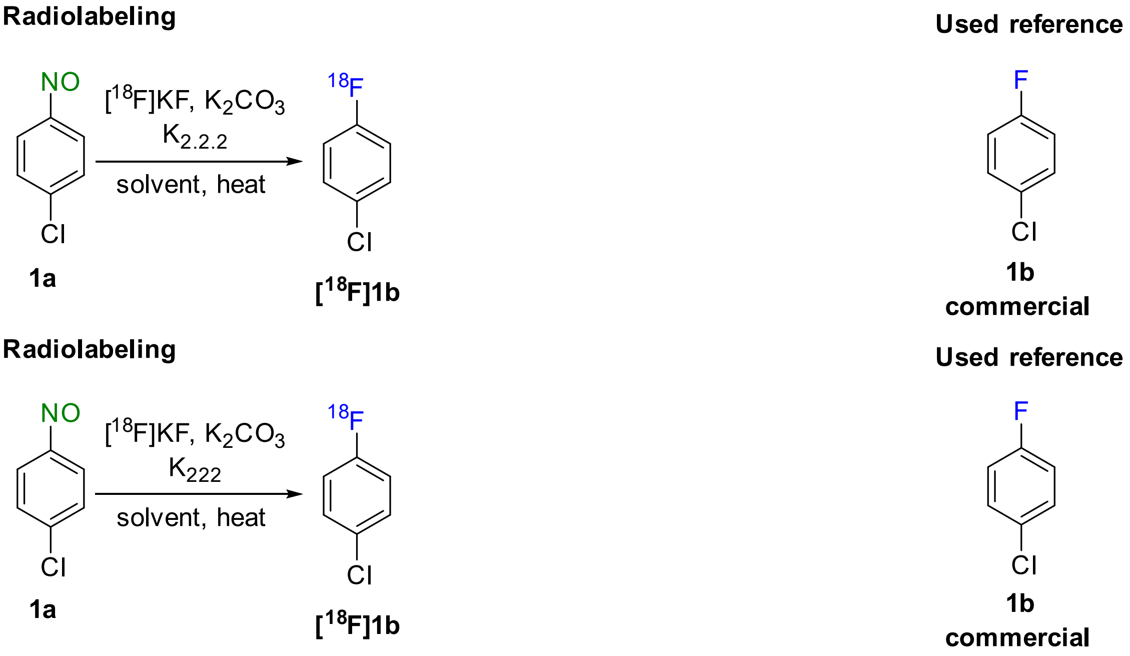

Figure S51. Overview for radiolabeling and used references

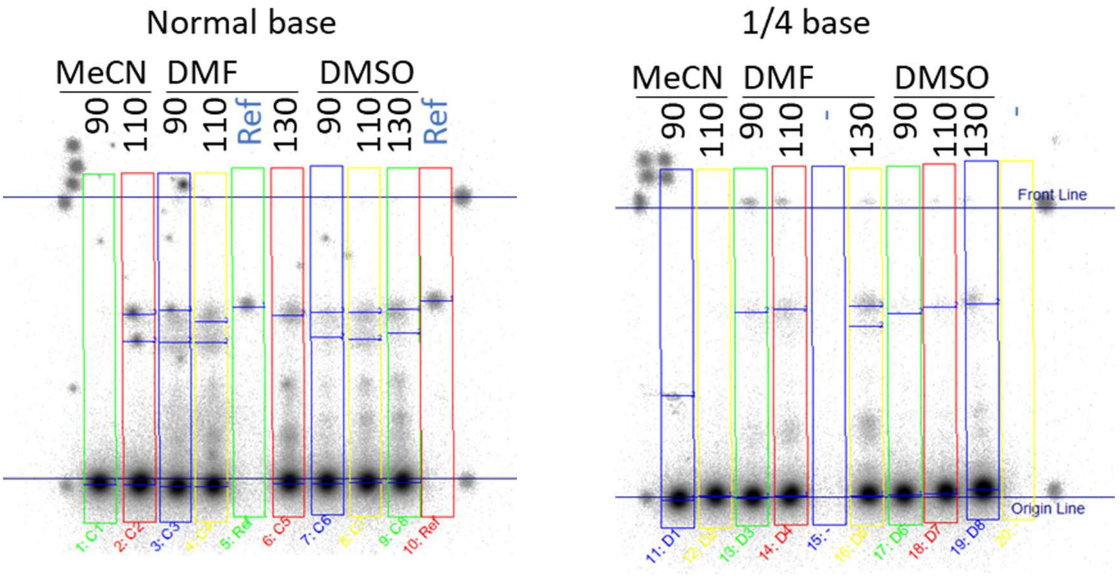

Figure S52. Copy of radio-TLC obtained for ^{18}F-labeling of compound **1a** using normal base (left) and ¼ base (right).

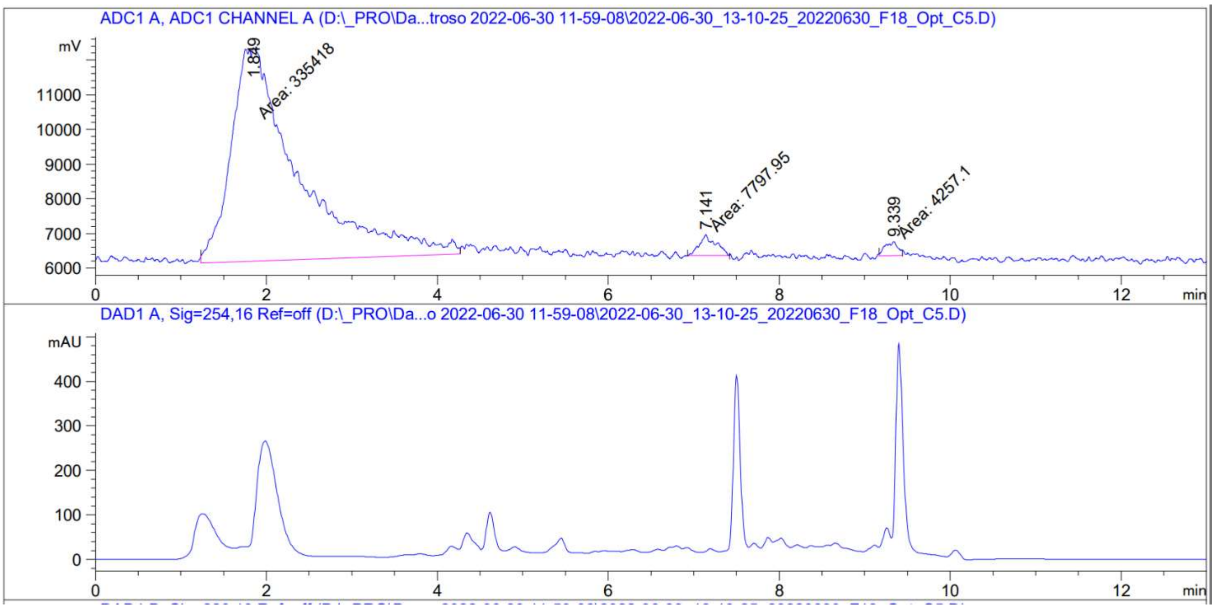

Figure S53. Exemplary analytical radio-HPLC chromatogram (System 2, gradient 2; upper panel: signal of gamma-detector; lower panel: UV-signal) of the crude reaction mixture obtained for ^{18}F-labeling of compound **1a** and reaction with normal base at 130°C in DMF.

A)				TLC		Set	C	D		
			1	2	3	4	5	6	7	8
	SRIM3B		MeCN		DMF			DMSO		
			90	110	90	110	130	90	110	130
C	**Normal**	Product	0	2	1,8	1,5	1,8	0,7	1,7	1,7
		Side Products		1,2	1,8	1,5	1,5	0,5	1	0,4
D	**1/4 Base**	Product	0	0	0,5	0,9	1,2	0,2	0,3	0,5
		Side Products								
B)				HPLC						
			1	2	3	4	5	6	7	8
	SRIM3B		MeCN		DMF			DMSO		
			90	110	90	110	130	90	110	130
C	**Normal**	Product	n.d	0	n.d.	n.d.	2,2	n.d.	n.d.	n.d.
		Side Products					1,2			
D	**1/4 Base**	Product	n.d	n.d.	n.d.	n.d.	n.d.	n.d.	n.d.	n.d.
		Side Products								

Figure S54. Detailed results of optimization experiments for ^{18}F-labeling of compound **1a** obtained by radio-TLC (A) and radio-HPLC (B) analysis. n.d. not determined.

Radiolabeling of compound 2a

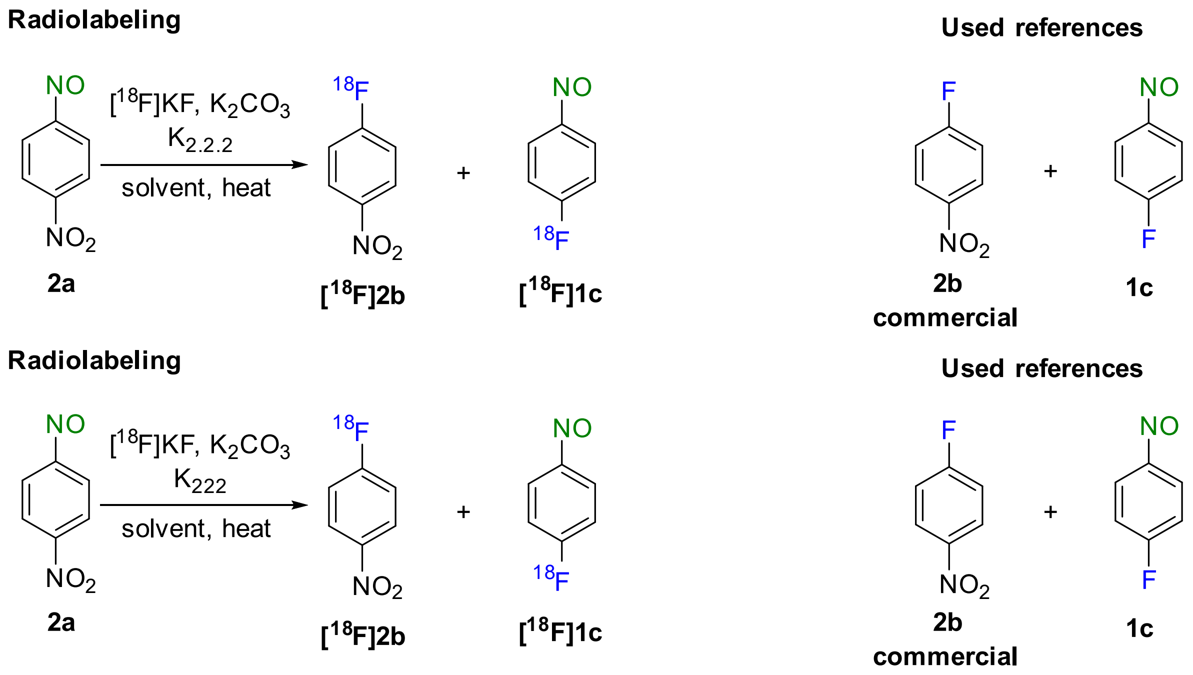

Figure S55. Overview for radiolabeling and used references

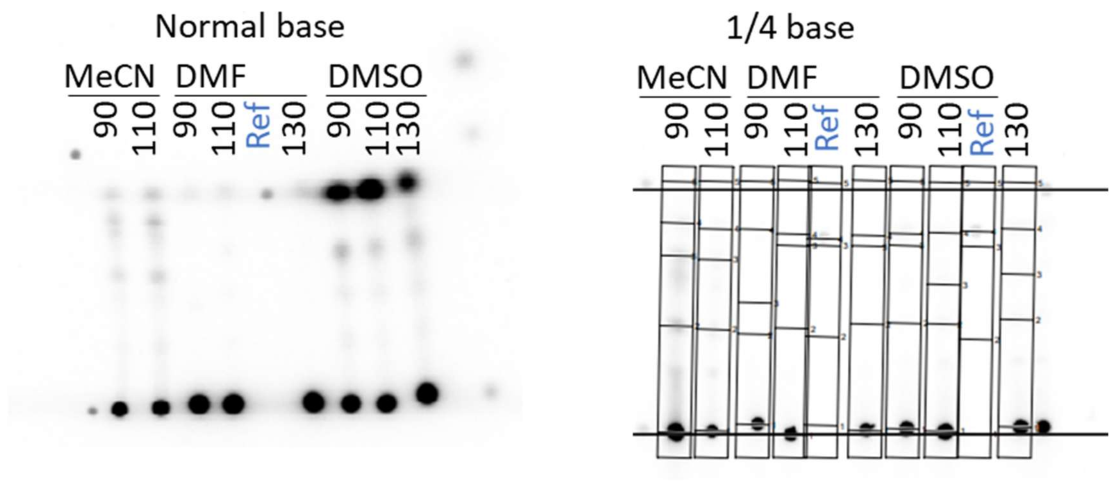

Figure S56. Copy of radio-TLC obtained for ^{18}F-labeling of compound **2a** using normal base (left) and ¼ base (right).

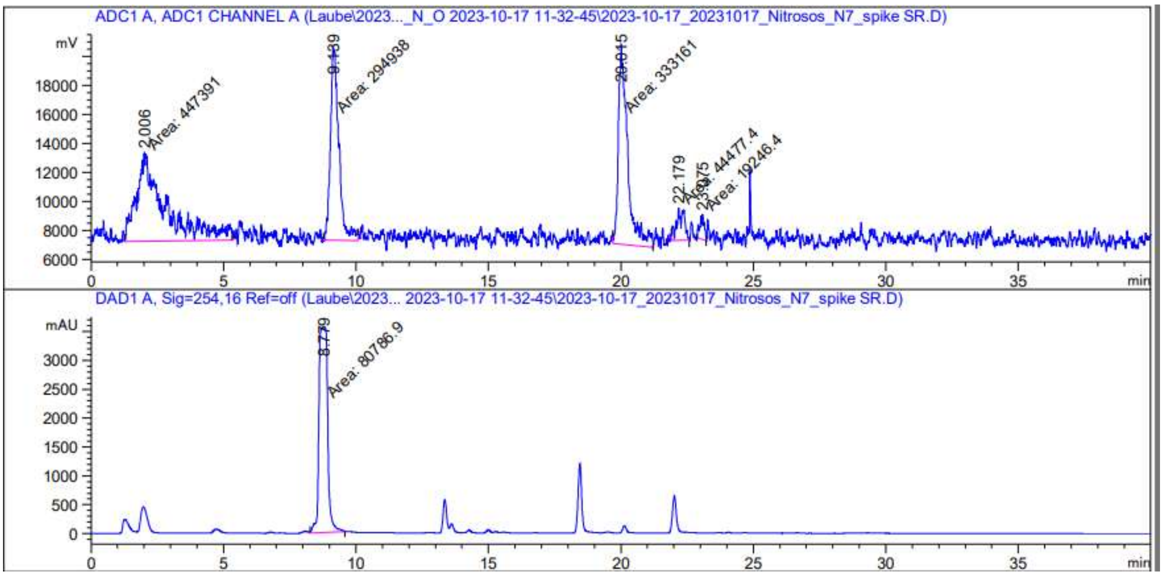

Figure S57. Exemplary analytical radio-HPLC chromatogram (System 2, gradient 1; upper panel: signal of gamma-detector; lower panel: UV-signal) of the crude reaction mixture obtained for ^{18}F-labeling of compound **2a** and reaction with normal base at 110°C in DMSO. Authentic non-radioactive reference **2b** was added to the reaction mixture (t_R 8.779 min).

A)	17. Okt 23			DC		Set				
			1	2	3	4	5	6	7	8
	**SR34/23**		**MeCN**		**DMF**			**DMSO**		
			90	110	90	110	130	90	110	130
E	**Normal**	Product	4,1	5,5	1,9	2,4	6,1	49,3	27,4	38,5
		Side Products	10,1	14	0	1	0,4	3,7	8,4	6,6
F	**1/4 Base**	Product	0,7	1,7	0,5	0,4	0,3	2,2	0,8	1,9
		Side Products	13,4	9,4	0,4	0,5	0,4	0,9	2,2	2,8

B)	17. Okt 23			HPLC		Set	E	F		
			1	2	3	4	5	6	7	8
	**SR34/23**		**MeCN**			**DMF**			**DMSO**	
			90	110	90	110	130	90	110	130
E	**Normal**	Product	3,3	0	0	0	0	34,7	29,2	0
		Side Products	13	17,4	0	0	0	24,2	38,5	36
F	**1/4 Base**	Product	0	0	0	0	0	0	0	0
		Side Products	8,6	5,1	0	0	0	0	0	0

Figure S58. Detailed results of optimization experiments for ^{18}F-labeling of compound **2a** obtained by radio-TLC (A) and radio-HPLC (B) analysis.

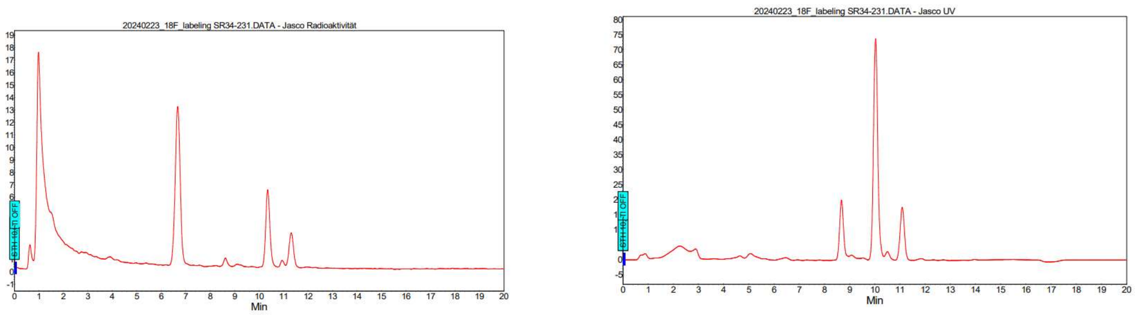

Figure S59. Copy of semi-preparative HPLC chromatograms (System 3; left: signal of gamma-detector; right: UV-signal) obtained for purification of compound **[^{18}F]2b** after labeling of **2a** with [^{18}F]fluoride under optimized conditions. Product was collected between 6.5-7.2 min.

	#1	#2	#3	#4	#5	#6	Mean ± SD (n)
RCY [%]	12	14	17				14.3 ± 2.1 (n=3)

Table S1. Detailed results of RCY and Mean ± SD (n) for the radiosynthesis and isolation of **[^{18}F]2b**.

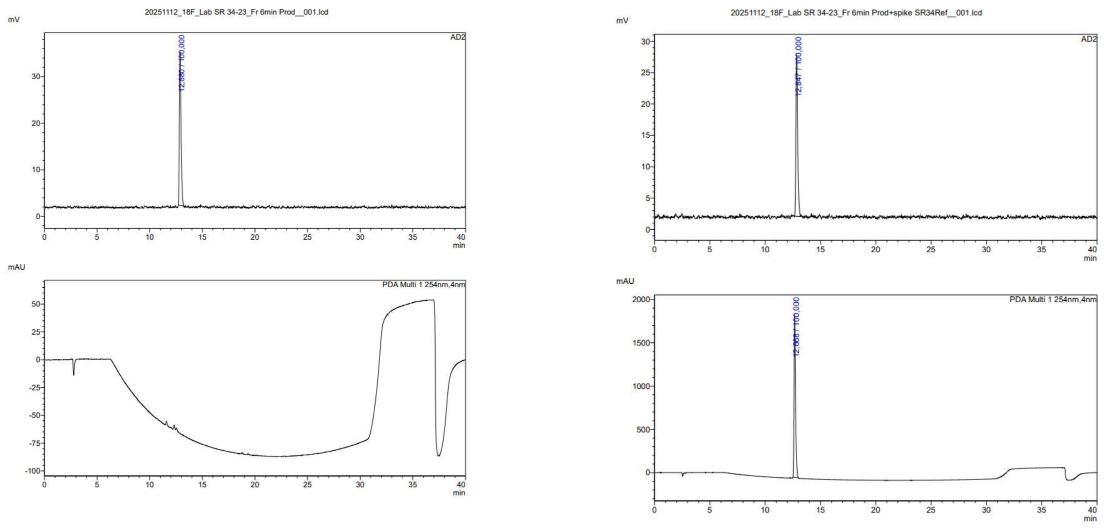

Figure S60. Copy of analytical HPLC chromatograms (System 1, gradient 2; upper panel: signal of gamma-detector; lower panel: UV-signal) obtained for compound **[^{18}F]2b** after semi-preparative purification without (left) and with (right) addition of the authentic non-radioactive reference. In the HPLC setup, the UV detector is in row before the γ-detector with Δt_R of 0.17-0.18 min between both detectors.

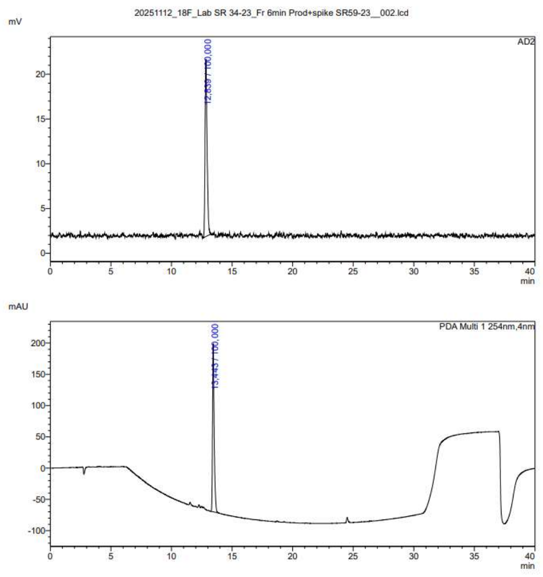

Figure S61. Copy of analytical HPLC chromatogram (System 1, gradient 2; upper panel: signal of gamma-detector; lower panel: UV-signal) obtained for compound **[^{18}F]2b** after semi-preparative purification with addition of 1-nitroso-4-fluorobenzene. In the HPLC setup, the UV detector is in row before the γ-detector with Δt_R of 0.17-0.18 min between both detectors. The UV signal of 1-Nitroso-4-fluorobenzene(**1c**; t_R 13.443) shows that the isolated ^{18}F-labeled product does not contain 1-Nitroso-4-[^{18}F]fluorobenzene.

Radiolabeling of compound 3a

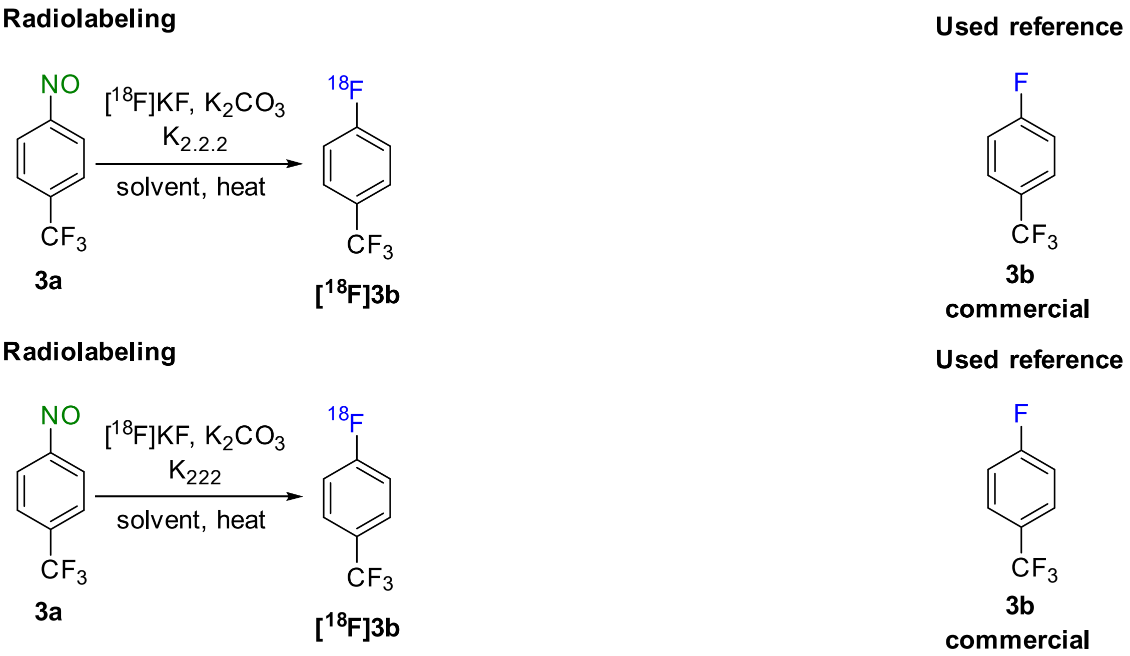

Figure S62. Overview for radiolabeling and used references

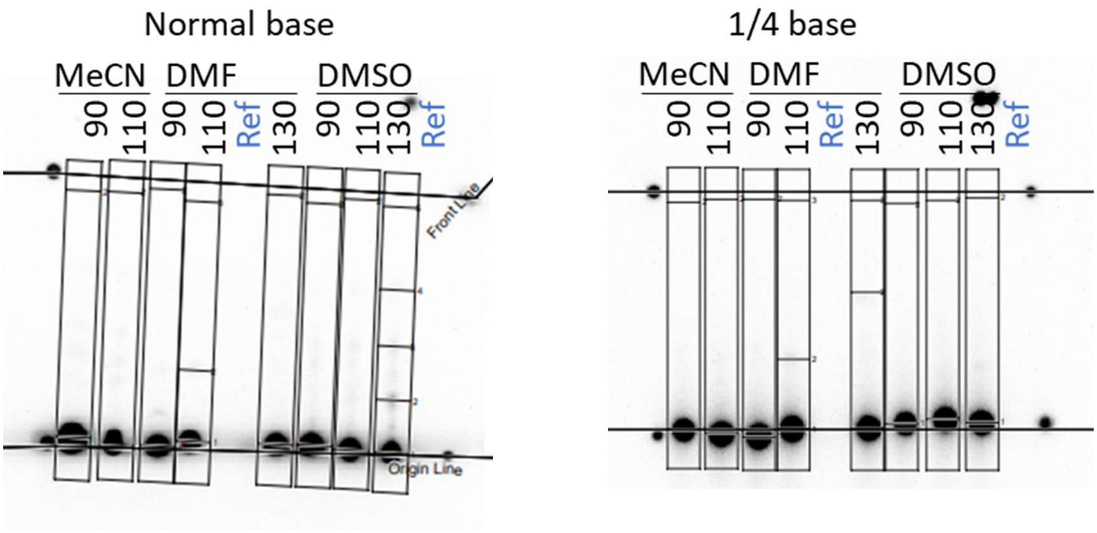

Figure S63. Copy of radio-TLC obtained for ^{18}F-labeling of compound **3a** using normal base (left) and ¼ base (right). Authentic non-radioactive reference is a liquid and was not visible on TLC by UV detection at 254 nm after development.

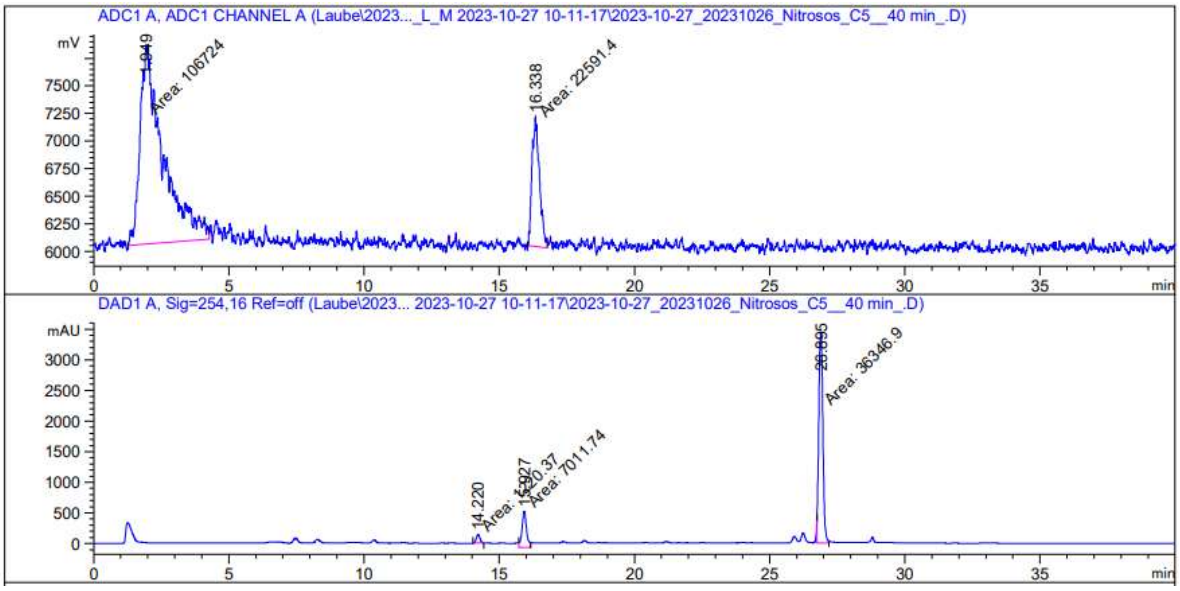

Figure S64. Exemplary analytical radio-HPLC chromatogram (System 2, gradient 1; upper panel: signal of gamma-detector; lower panel: UV-signal) of the crude reaction mixture spiked with authentic non-radioactive reference (t_R 15.927 min) obtained for ^{18}F-labeling of compound **3a** and reaction with normal base at 130°C in DMF.

A)				TLC		Set	C	D		
			1	2	3	4	5	6	7	8
	**SR 44/23**		**MeCN**		**DMF**			**DMSO**		
			90	110	90	110	130	90	110	130
C	**Normal**	Product	0	0	0	0	0	0	0	0,6
		Side Products	0	0	0	0,5	0	0	0	1,2
D	**1/4 Base**	Product	0	0	0	0	0,3	0	0	0
		Side Products	0	0	0	0,5	0	0	0	0

B)				HPLC						
			1	2	3	4	5	6	7	8
	**SR 44/23**		**MeCN**			**DMF**			**DMSO**	
			90	110	90	110	130	90	110	130
C	**Normal**	Product	0	0	0	2	19	0	0	2,6
		Side Products	0	0	0	0	0	0	0	0
D	**1/4 Base**	Product	0	0	0	0,7	1,7	0	0	0
		Side Products	0	0	0	0	0	0	0	0

Figure 65. Detailed results of optimization experiments for ^{18}F-labeling of compound **3a** obtained by radio-TLC (A) and radio-HPLC (B) analysis.

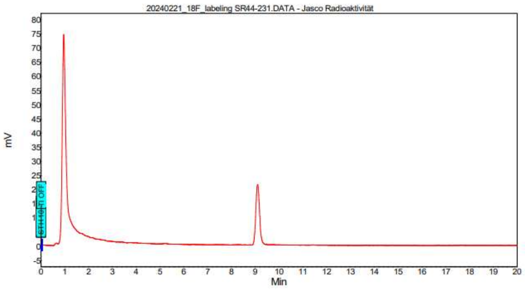

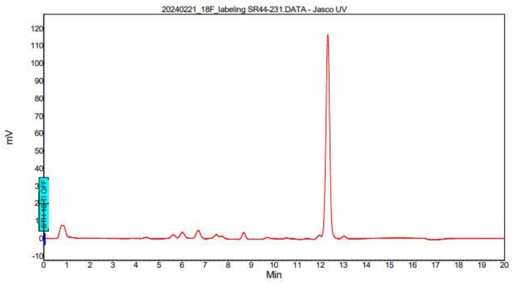

Figure S66. Copy of semi-preparative HPLC chromatograms (System 3; left: signal of gamma-detector; right: UV-signal) obtained for purification of compound **[^{18}F]3b** after labeling of **3a** with [^{18}F]fluoride under optimized conditions. Product was collected between 8.9and 9.6 min.

	#1	#2	#3	#4	#5	#6	Mean ± SD (n)
RCY [%]	12	13	14				13.0 ± 0.8 (n=3)

Table S2. Detailed results of RCY and Mean ± SD (n) for the radiosynthesis and isolation of **[^{18}F]3b**.

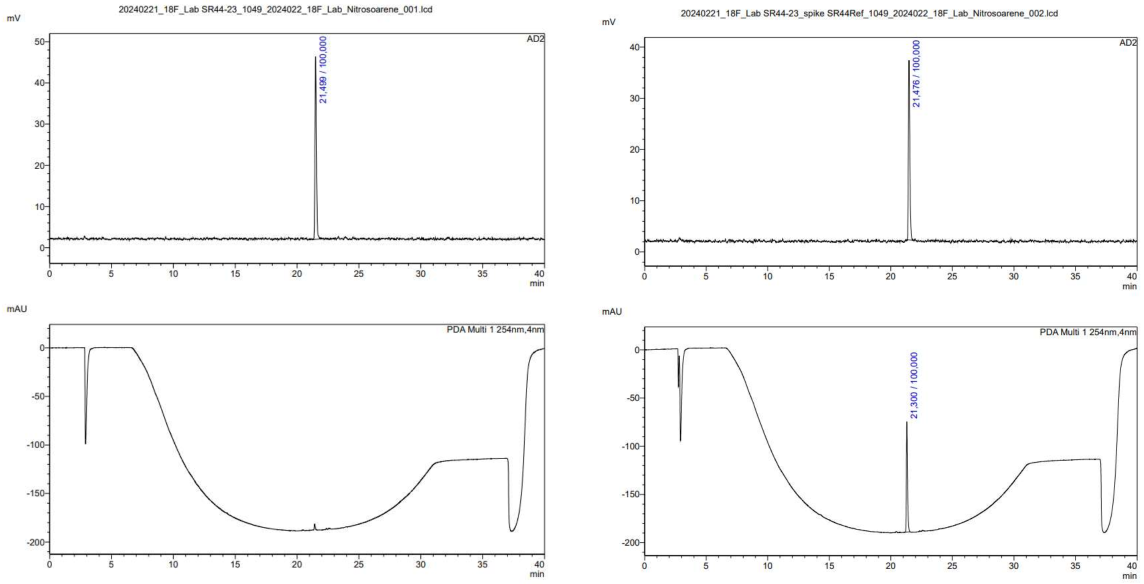

Figure S67. Copy of analytical HPLC chromatograms (System 1; upper panel: signal of gamma-detector; lower panel: UV-signal) obtained for compound **[^{18}F]3b** after semi-preparative purification without (left) and with (right) addition of the authentic non-radioactive reference. In the HPLC setup, the UV detector is in row before the γ-detector with Δt_R of 0.17-0.18 min between both detectors.

Radiolabeling of compound 4a

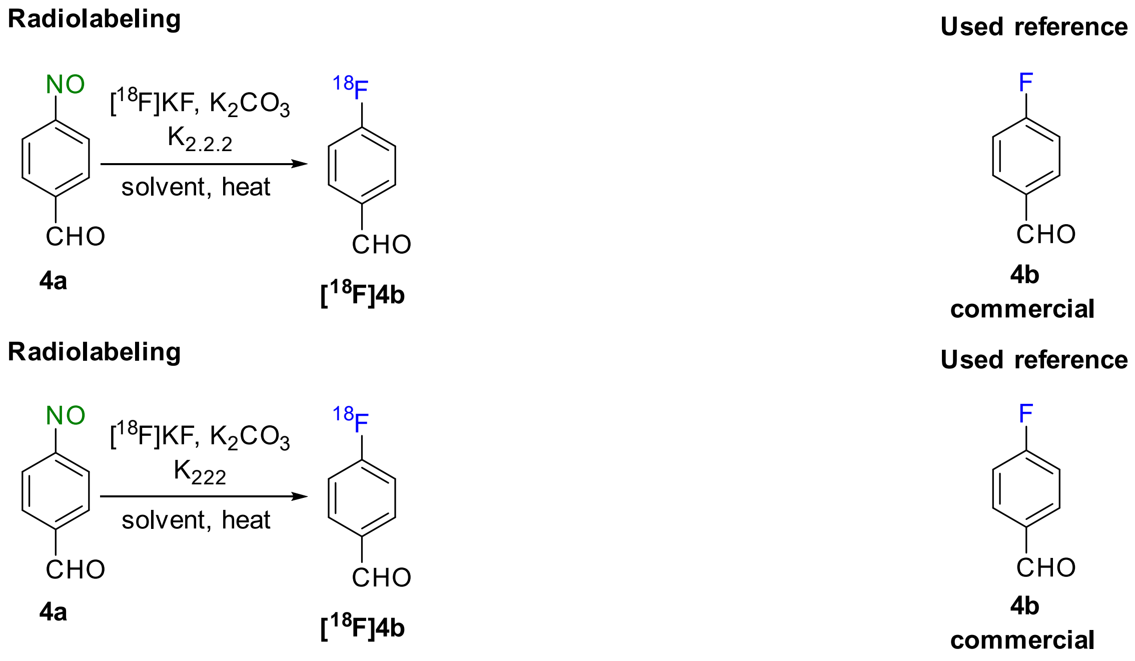

Figure 68. Overview for radiolabeling and used references

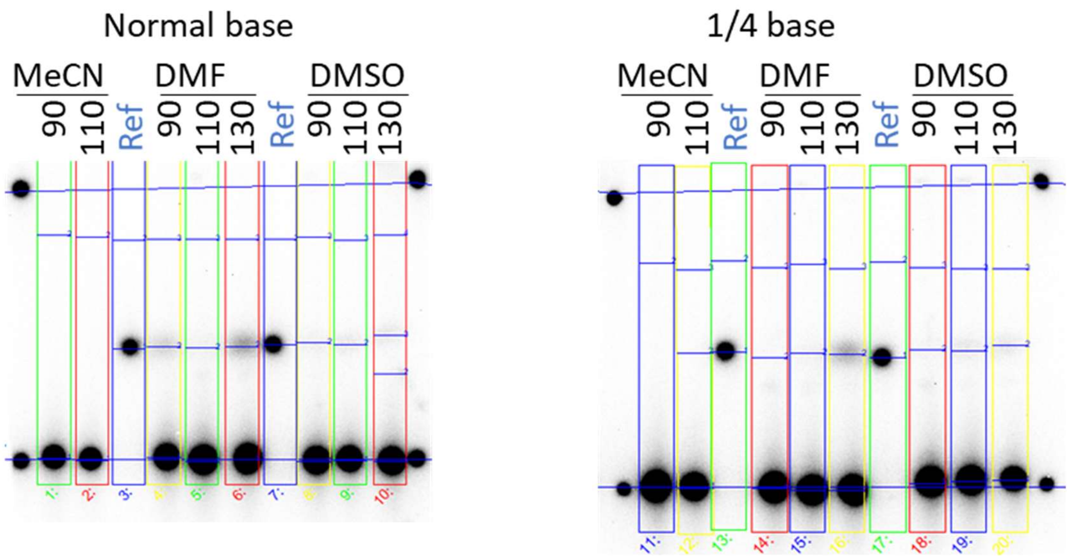

Figure 69. Copy of radio-TLC obtained for ^{18}F-labeling of compound **4a** using normal base (left) and ¼ base (right).

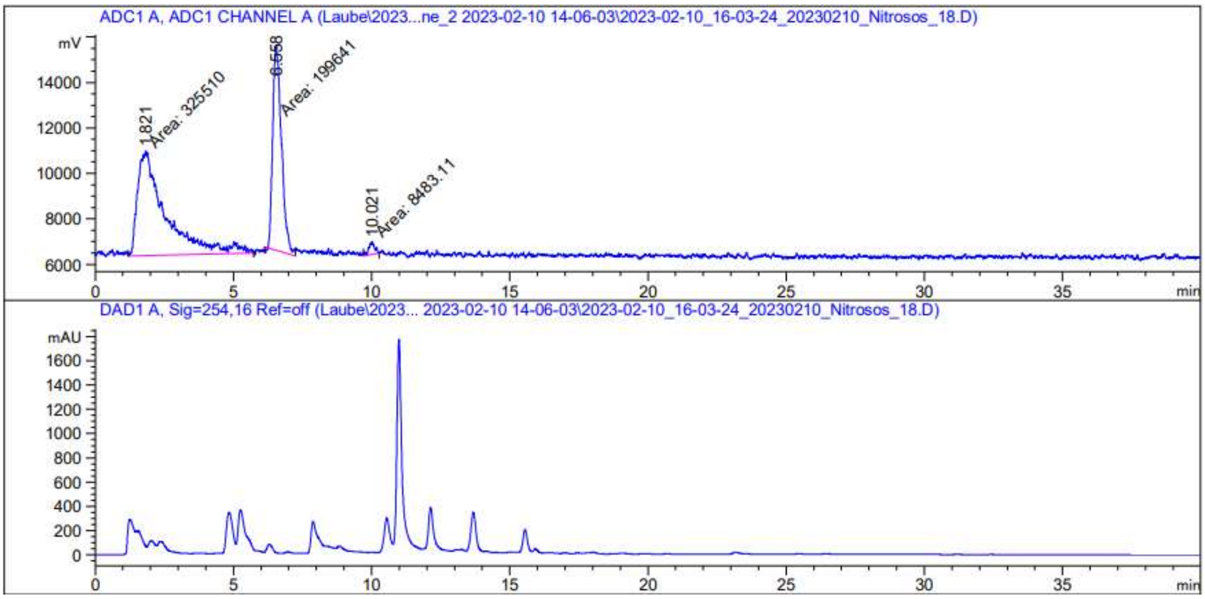

Figure S70. Exemplary analytical radio-HPLC chromatogram (System 2, gradient 1; upper panel: signal of gamma-detector; lower panel: UV-signal) of the crude reaction mixture obtained for ^{18}F-labeling of compound **4a** and reaction with normal base at 110°C in DMF.

A)			TLC		Set	E	F			
			1	2	3	4	5	6	7	8
	**SR185F1**		**MeCN**		**DMF**			**DMSO**		
			90	110	90	110	130	90	110	130
E	**Normal**	Product	0	0	3,8	1,2	6,2	1,5	1,7	1,4
		Side Products								
F	**1/4 Base**	Product	0	0,9	0,5	0,9	5	0,8	1,3	1,7
		Side Products								

B)			HPLC							
			1	2	3	4	5	6	7	8
	**SR185F1**		**MeCN**		**DMF**			**DMSO**		
			90	110	90	110	130	90	110	130
E	**Normal**	Product	0	0	4,2	8,1	40,8	n.d.	2,5	2,4
		Side Products	0,7	1,7	1,6	1,7	0		2,3	1,8
F	**1/4 Base**	Product	0	0	0	0	6,3	0	2,7	3,1
		Side Products								

Figure S71. Detailed results of optimization experiments for ^{18}F-labeling of compound **4a** obtained by radio-TLC (A) and radio-HPLC (B) analysis. n.d. not determined.

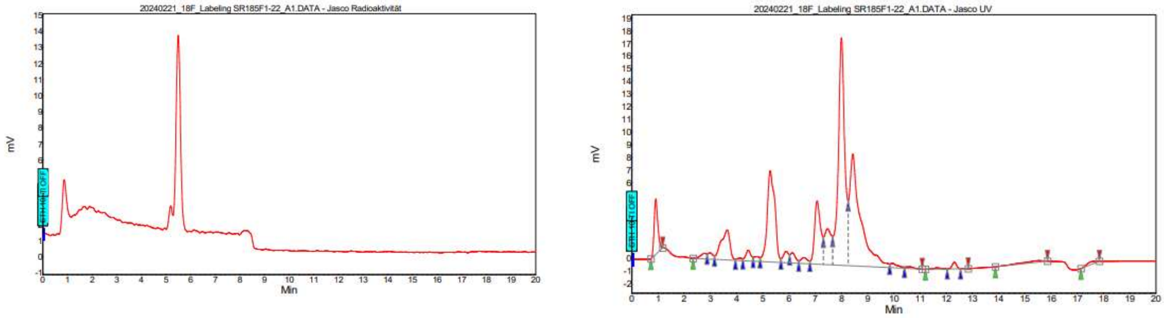

Figure S72. Copy of semi-preparative HPLC chromatograms (System 3; left: signal of gamma-detector; right: UV-signal) obtained for purification of compound **[^{18}F]4b** after labeling of **4a** with [^{18}F]fluoride under optimized conditions. Product was collected between 5.0 and 6.0 min.

	#1	#2	#3	#4	#5	#6	Mean ± SD (n)
RCY [%]	22	19	63	48			38.0 ± 18.3 (n=4)

Table S3. Detailed results of RCY and Mean ± SD (n) for the radiosynthesis and isolation of **[^{18}F]4b**.

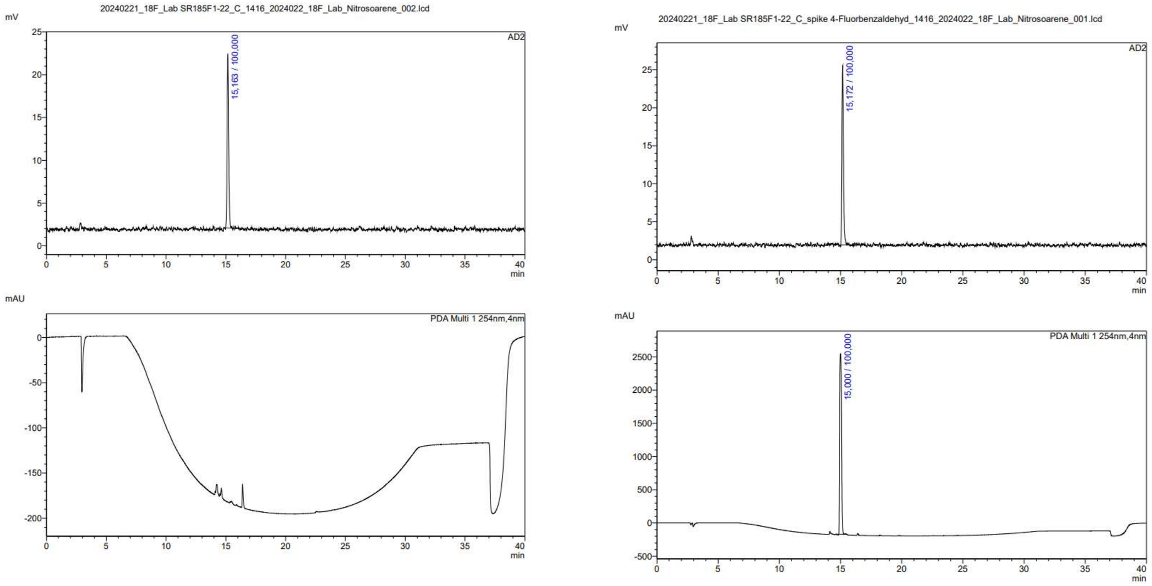

Figure 73. Copy of analytical HPLC chromatograms (System 1; upper panel: signal of gamma-detector; lower panel: UV-signal) obtained for compound **[^{18}F]4b** after semi-preparative purification without (left) and with (right) addition of the authentic non-radioactive reference 4-fluorobenzaldehyde. In the HPLC setup, the UV detector is in row before the γ-detector with Δt_R of 0.17-0.18 min between both detectors.

Radiolabeling of compound 5a

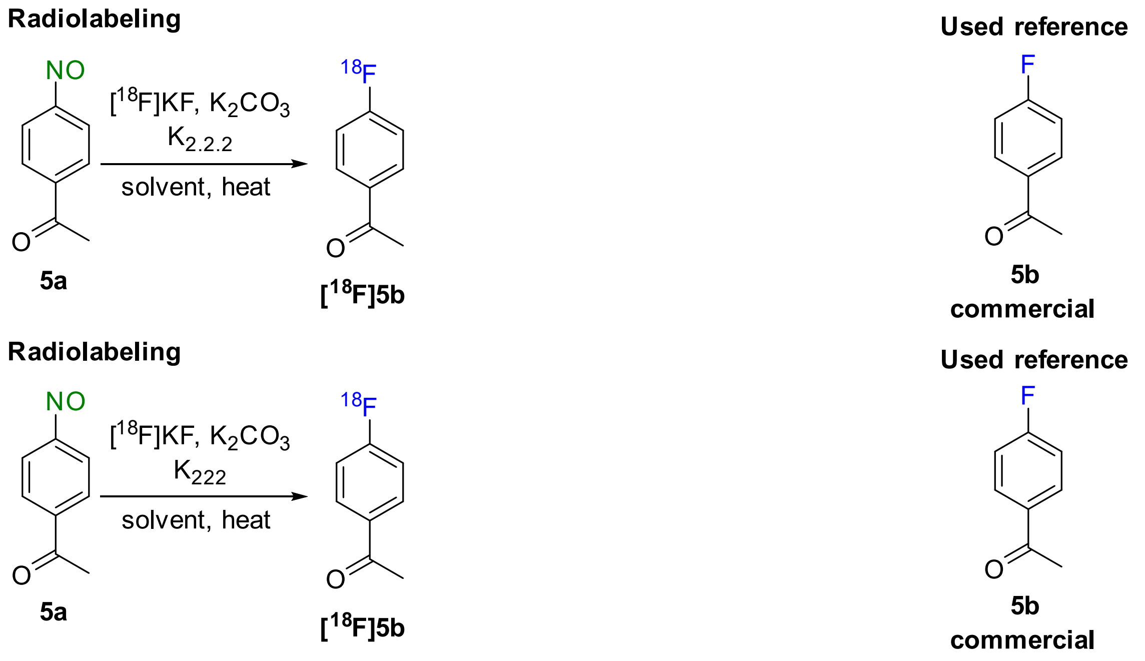

Figure S74. Overview for radiolabeling and used references

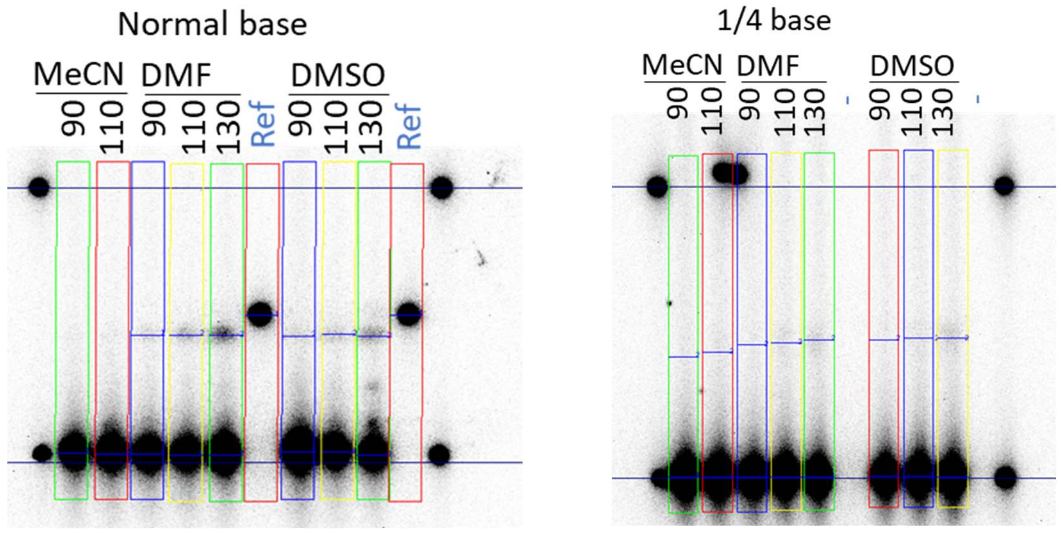

Figure S75. Copy of radio-TLC obtained for ^{18}F-labeling of compound **5a** using normal base (left) and ¼ base (right). Of note, the wrong reference 4-fluorobenzonitrile (**9b**) was spotted on TLC and can not be regarded as suitable reference for this radio-TLC.

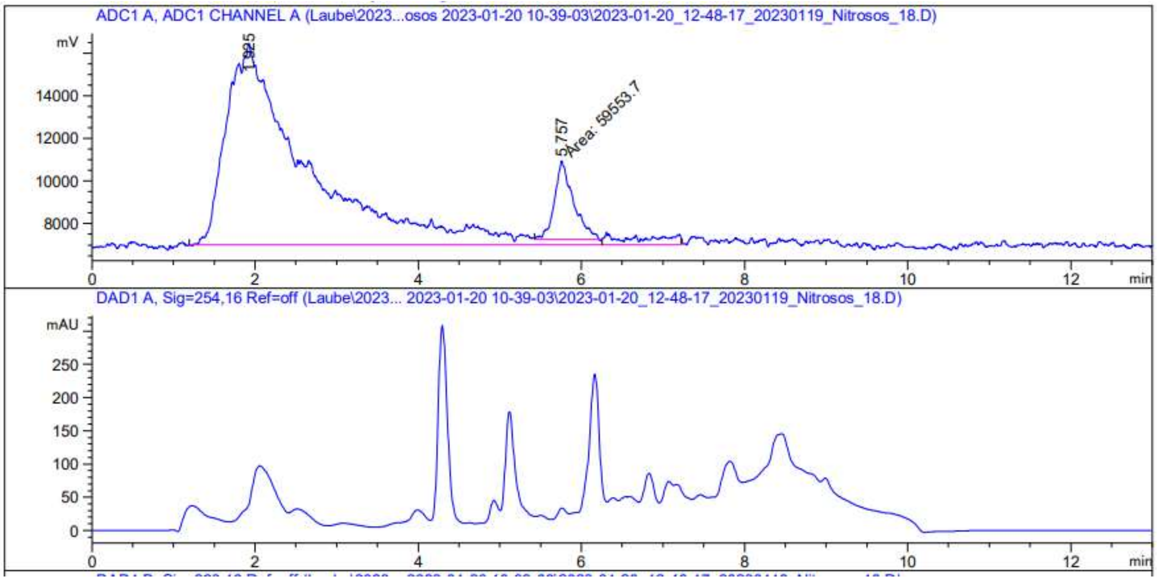

Figure S76. Exemplary analytical radio-HPLC chromatogram (System 2, gradient 2; upper panel: signal of gamma-detector; lower panel: UV-signal) of the crude reaction mixture obtained for ^{18}F-labeling of compound **5a** and reaction with normal base at 130°C in DMSO.

A)				TLC		Set	C	D		
			1	2	3	4	5	6	7	8
	**SR177F1/22**		**MeCN**		**DMF**			**DMSO**		
			90	110	90	110	130	90	110	130
	**Normal**	Product	0	0	0,6	1	1,8	0,4	0,9	1,5
		Side Products	0	0	0	0	0	0	0	0
	**1/4 Base**	Product	0,1	0,1	0,1	0,2	0,2	0,1	0,1	0,3
		Side Products	0	0	0	0	0	0	0	0

B)				HPLC						
			1	2	3	4	5	6	7	8
	**SR177F1/22**		**MeCN**		**DMF**			**DMSO**		
			90	110	90	110	130	90	110	130
	**Normal**	Product	0,6	2,9	1,8	3,3	5,7	1,4	3,3	8,2
		Side Products	0	1,7	0	0	0	0	0	0
	**1/4 Base**	Product	0	0	0	0	1,7	0	0	1,7
		Side Products	0	0	0	0	0	0	0	0

Figure 77. Detailed results of optimization experiments for ^{18}F-labeling of compound **5a** obtained by radio-TLC (A) and radio-HPLC (B) analysis. n.d. not determined.

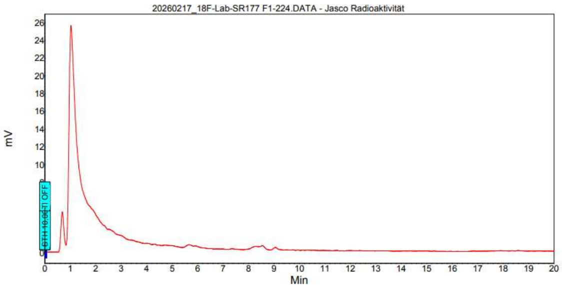

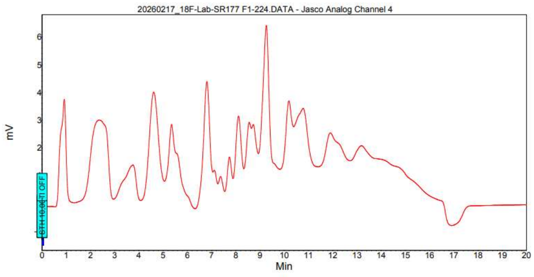

Figure S78. Copy of semi-preparative HPLC chromatograms (System 3; left: signal of gamma-detector; right: UV-signal) obtained for purification of compound **[^{18}F]5b** after labeling of **5a** with [^{18}F]fluoride under optimized conditions leading to 2% isolated RCY. Product was collected between 5.5 and 6.2 min.

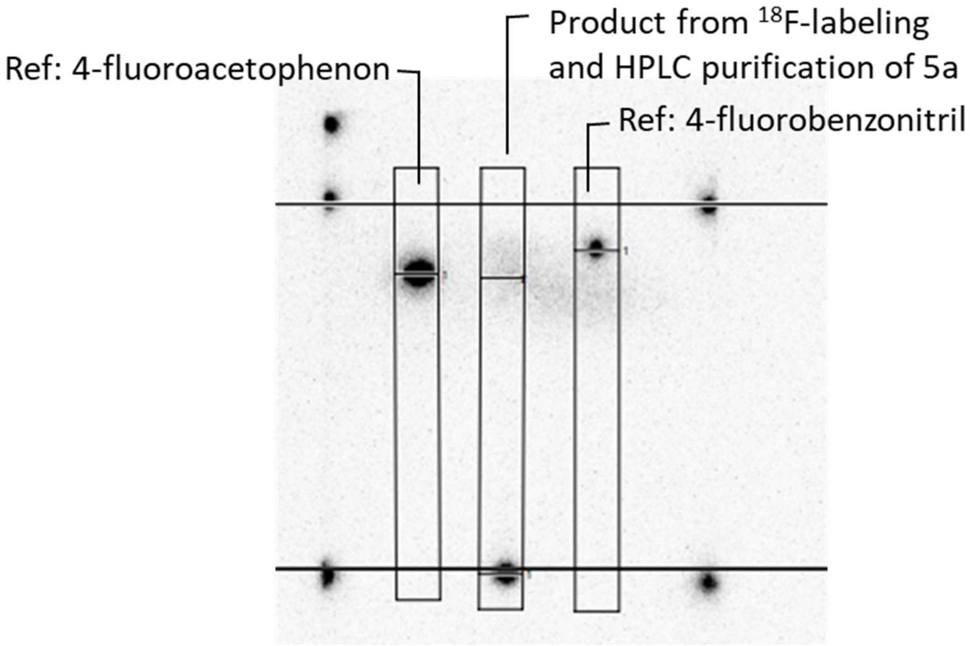

Figure S10.
Figure S11. Copy of radio-TLC obtained after labeling of **5a** with [^{18}F]fluoride using normal base, DMSO and 130°C leading to 2% isolated RCY. The collected fraction showed mainly [^{18}F]fluoride and no clear signal for **[^{18}F]5b**.

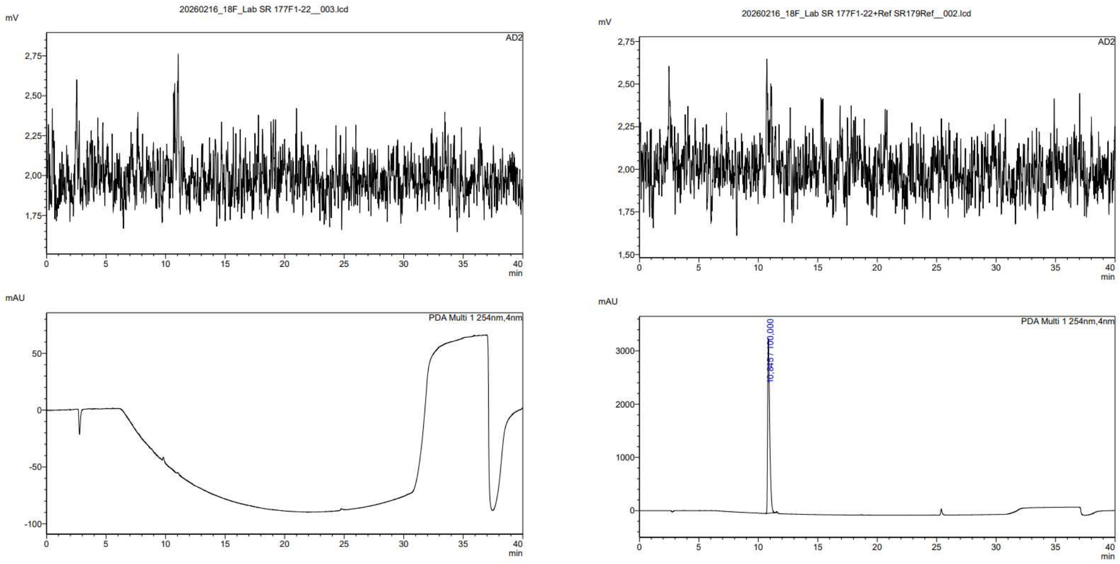

Figure S*12*. Copy of analytical HPLC chromatograms (System 1, gradient 2; upper panel: signal of gamma-detector; lower panel: UV-signal) obtained for collected fraction from the reaction using normal base, DMSO at 130°C and after semi-preparative purification without (left) and with (right) addition of the authentic non-radioactive reference. In the HPLC setup, the UV detector is in row before the γ-detector with Δt_R of 0.17-0.18 min between both detectors. The collected fraction showed no clear signal of compound **[^{18}F]5b**.

Radiolabeling of compound 6a

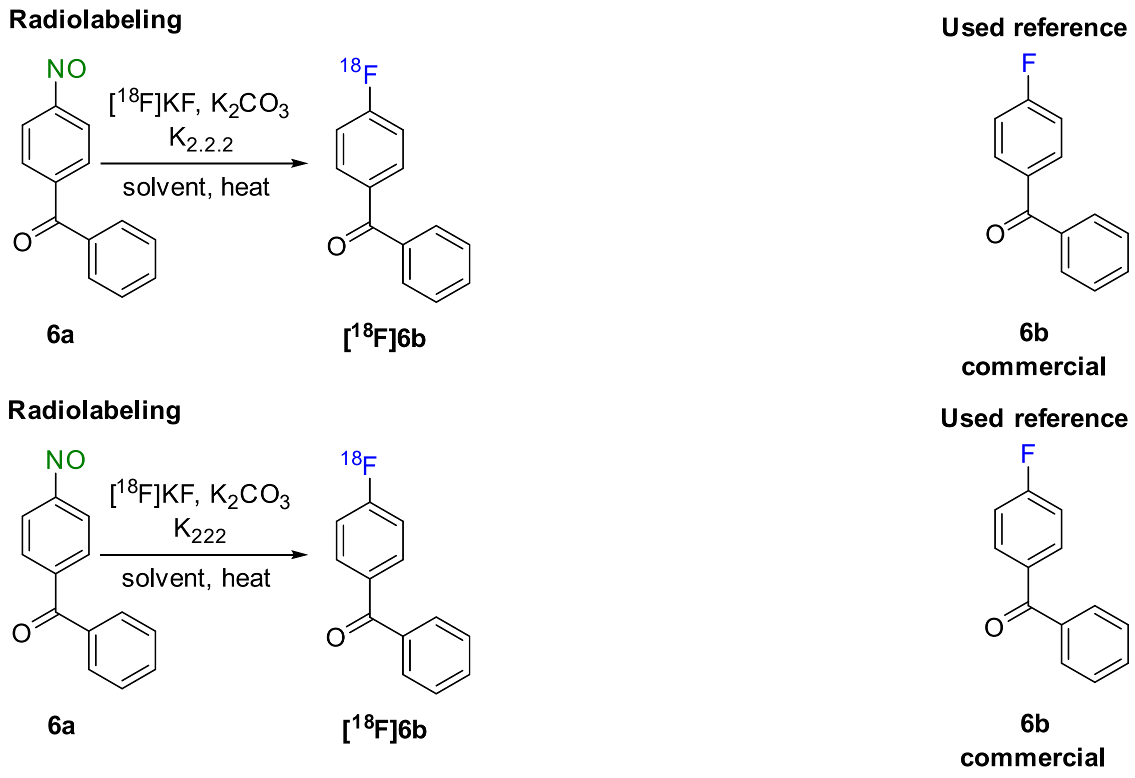

Figure S*13*. Overview for radiolabeling and used references

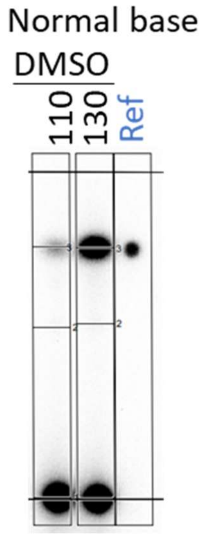

Figure S14. Copy of radio-TLC obtained for ^{18}F-labeling of compound **6a** using normal base. TLC conditions silica gel; n-hexan/EtOAC 50/50.

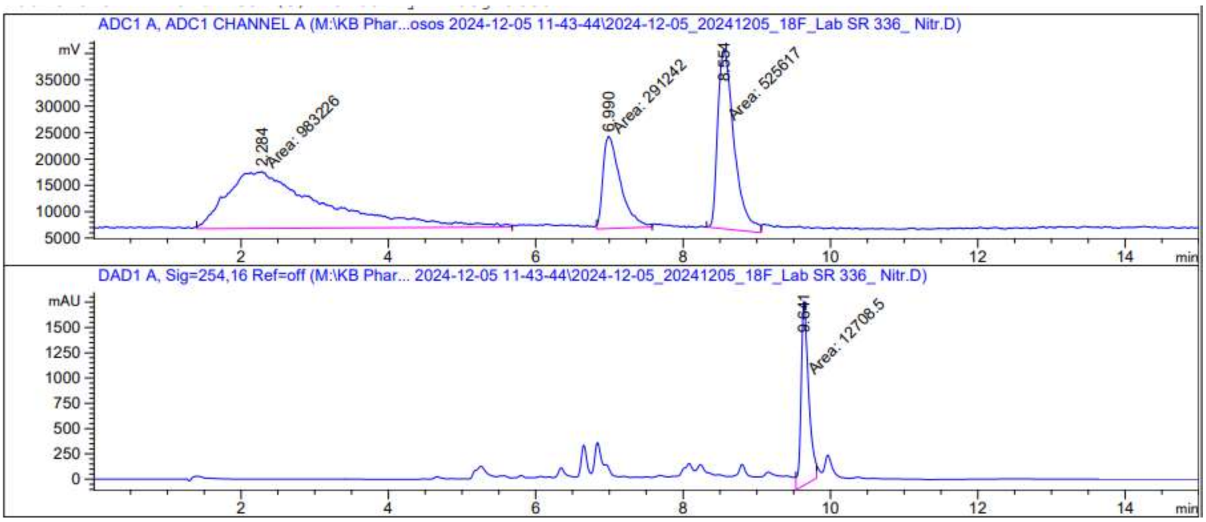

Figure S15. Exemplary analytical radio-HPLC chromatogram (System 2, gradient 3; upper panel: signal of gamma-detector; lower panel: UV-signal) of the crude reaction mixture obtained for ^{18}F-labeling of compound **6a** and reaction with normal base at 130°C in DMSO.

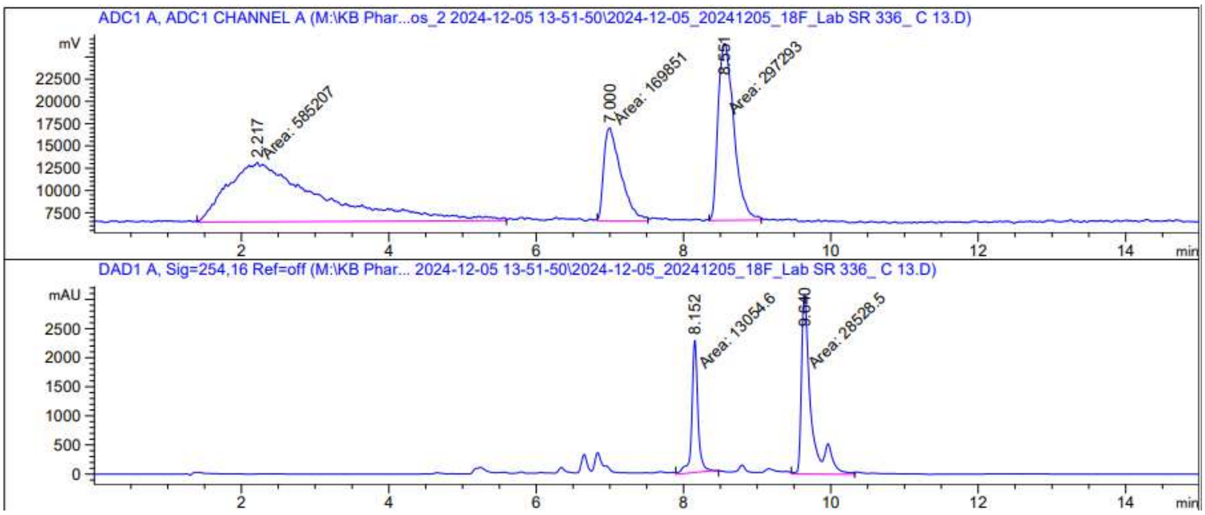

Figure S16. Exemplary analytical radio-HPLC chromatogram (System 2, gradient 3; upper panel: signal of gamma-detector; lower panel: UV-signal) of the crude reaction mixture spiked with authentic non-radioactive reference (t_R 8.152 min) obtained for ^{18}F-labeling of compound **6a** and reaction with normal base at 130°C in DMSO.

A)			TLC		Set				
		1	2	3	4	5	6	7	8
**SR336/24**		**MeCN**		**DMF**			**DMSO**		
		90	110	90	110	130	90	110	130
**Normal**	Product	n.d.	n.d.	n.d.	n.d.	n.d.	n.d.	4	29
	Side Products							0	0
**1/4 Base**	Product	n.d.	n.d.	n.d.	n.d.	n.d.	n.d.	n.d.	n.d.
	Side Products								

B)			HPLC						
		1	2	3	4	5	6	7	8
**SR336/24**		**MeCN**		**DMF**			**DMSO**		
		90	110	90	110	130	90	110	130
**Normal**	Product	n.d.	n.d.	n.d.	n.d.	n.d.	n.d.	n.d.	29
	Side Products								0
**1/4 Base**	Product	n.d.	n.d.	n.d.	n.d.	n.d.	n.d.	n.d.	n.d.
	Side Products								

Figure S17. Detailed results of optimization experiments for ^{18}F-labeling of compound **6a** obtained by radio-TLC (A) and radio-HPLC (B) analysis. n.d. not determined.

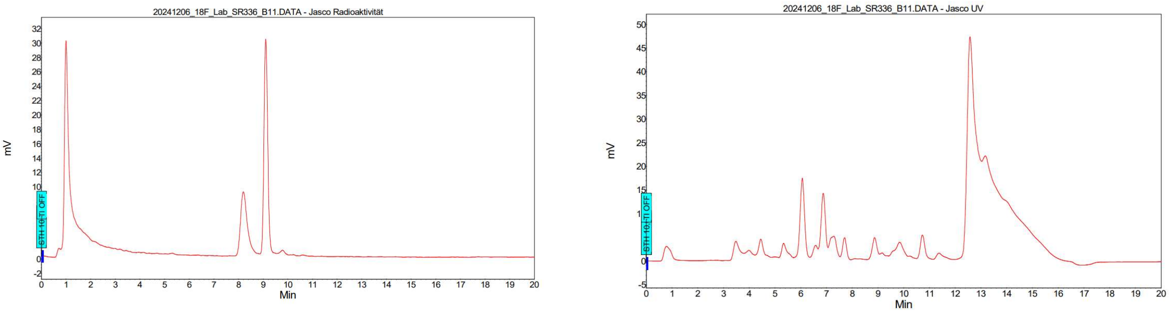

Figure S18. Copy of semi-preparative HPLC chromatograms (System 3; left: signal of gamma-detector; right: UV-signal) obtained for purification of compound **[^{18}F]6b** after labeling of **6a** with [^{18}F]fluoride under optimized conditions. Product was collected between 8.9 and 9.6 min.

	#1	#2	#3	#4	#5	#6	Mean ± SD (n)
RCY [%]	18	21	12	13			16.0 ± 3.7 (n=4)

Table S4. Detailed results of RCY and Mean ± SD (n) for the radiosynthesis and isolation of **[^{18}F]6b**.

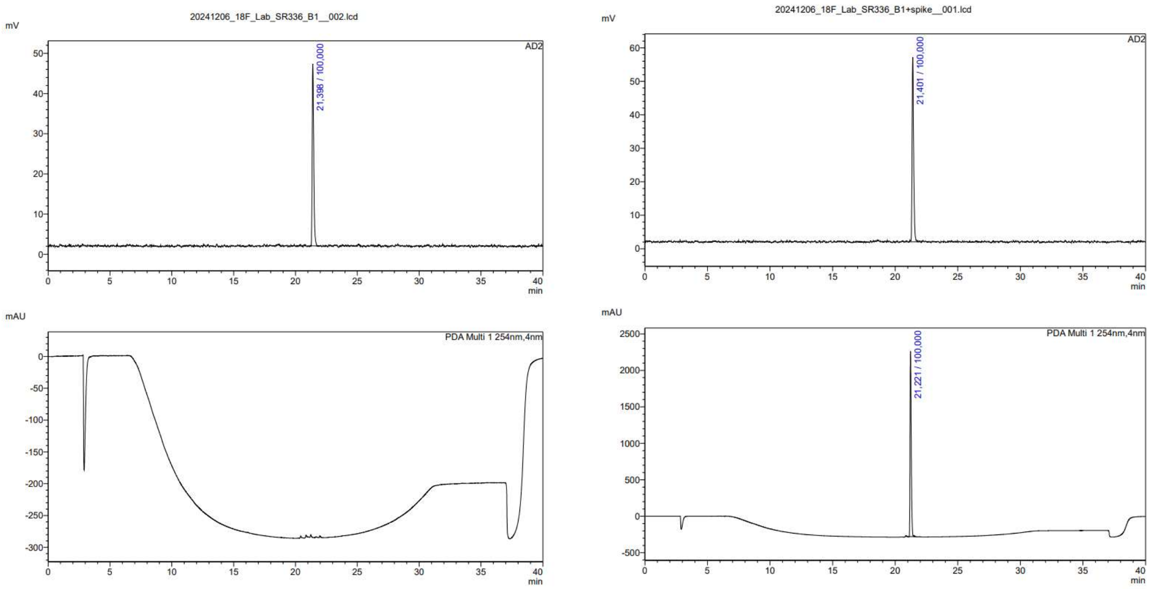

Figure S19. Copy of analytical HPLC chromatograms (System 1; upper panel: signal of gamma-detector; lower panel: UV-signal) obtained for compound **[^{18}F]6b** after semi-preparative purification without (left) and with (right) addition of the authentic non-radioactive reference. In the HPLC setup, the UV detector is in row before the γ-detector with Δt_R of 0.17-0.18 min between both detectors.

Radiolabeling of compound 7a

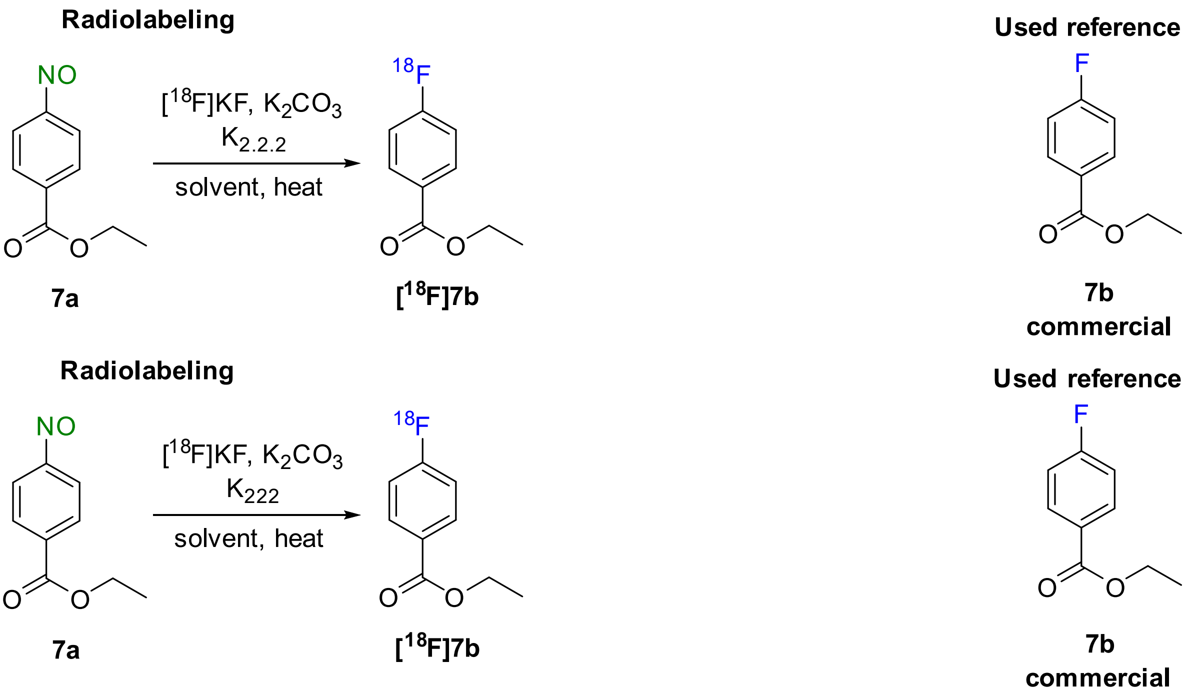

Figure S*20*. Overview for radiolabeling and used references

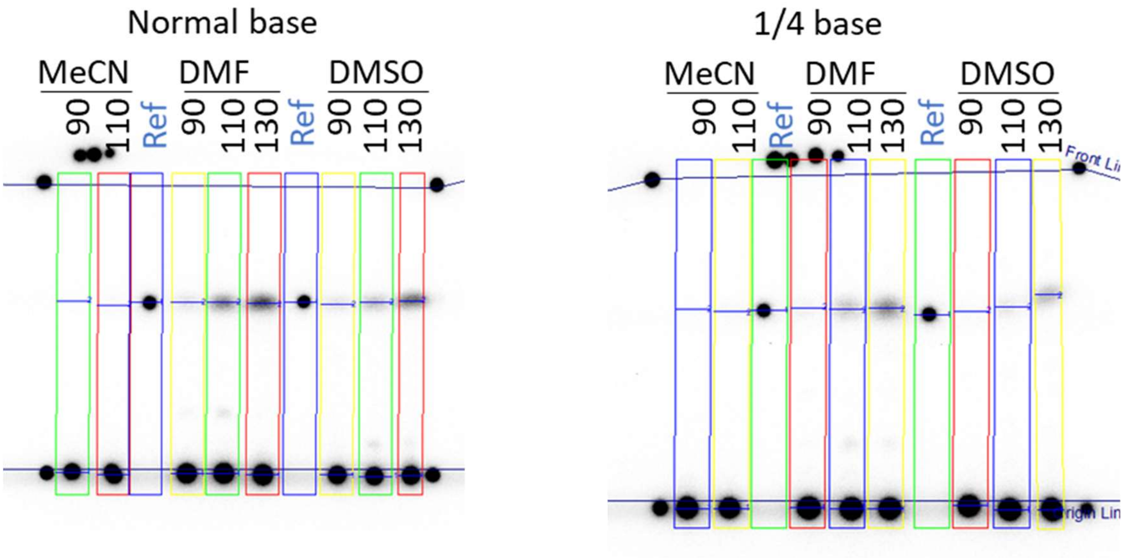

Figure S21. Copy of radio-TLC obtained for ^{18}F-labeling of compound **7a** using normal base (left) and ¼ base (right).

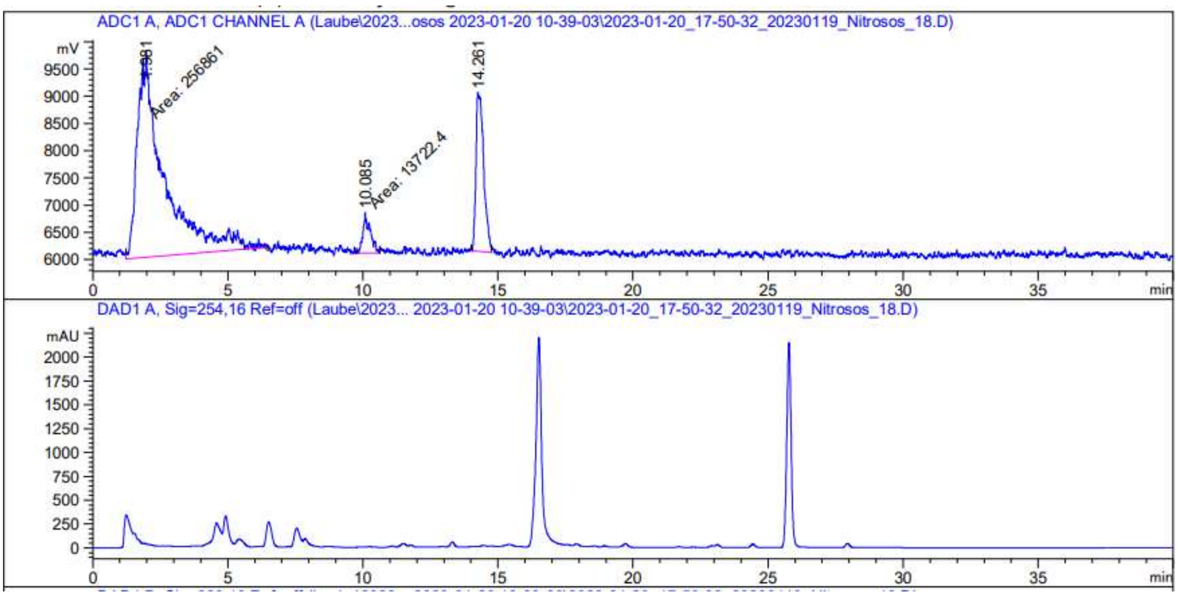

Figure S22. Exemplary analytical radio-HPLC chromatogram (System 2, gradient 1; upper panel: signal of gamma-detector; lower panel: UV-signal) of the crude reaction mixture obtained for ^{18}F-labeling of compound **7a** and reaction with normal base at 130°C in DMF.

A)				TLC		Set	I	K		
			1	2	3	4	5	6	7	8
	SR180F1/22		**MeCN**		**DMF**			**DMSO**		
			90	110	90	110	130	90	110	130
I	**Normal**	Product	0,3	1	3,1	9,3	13,7	3,3	6,6	11,8
		Side Products								
K	**1/4 Base**	Product	0,3	1	1,4	5,2	10,9	1	2,6	6,3
		Side Products								

B)				HPLC						
			1	2	3	4	5	6	7	8
	SR180F1/22		**MeCN**			**DMF**			**DMSO**	
			90	110	90	110	130	90	110	130
I	**Normal**	Product	n.d.	n.d.	2,7	13	18,7	9,5	8	19
		Side Products			4	7,5	3,8	3,1	11,3	13,8
K	**1/4 Base**	Product	n.d.	n.d.	1	6,3	15,6	n.d.	n.d.	n.d.
		Side Products			2,7	3	2,2			

Figure S23. Detailed results of optimization experiments for ^{18}F-labeling of compound **7a** obtained by radio-TLC (A) and radio-HPLC (B) analysis. n.d. not determined.

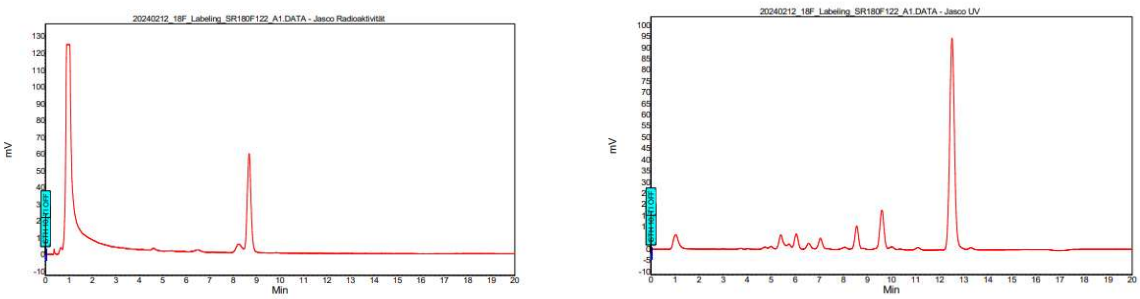

Figure S24. Copy of semi-preparative HPLC chromatograms (System 3; left: signal of gamma-detector; right: UV-signal) obtained for purification of compound **[^{18}F]7b** after labeling of **7a** with [^{18}F]fluoride under optimized conditions. Product was collected between 8.5and 9.25 min.

	#1	#2	#3	#4	#5	#6	Mean ± SD (n)
RCY [%]	4	21	28	41			23.5 ± 13.4 (n=4)

Table S5. Detailed results of RCY and Mean ± SD (n) for the radiosynthesis and isolation of **[^{18}F]7b**.

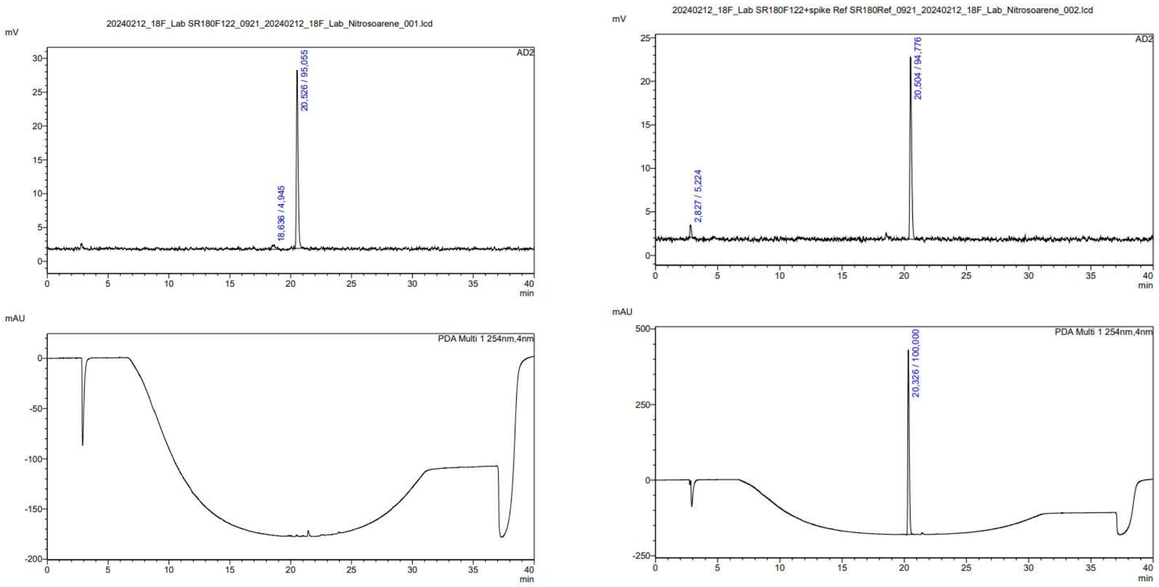

Figure S25. Copy of analytical HPLC chromatograms (System 1; upper panel: signal of gamma-detector; lower panel: UV-signal) obtained for compound **[^{18}F]7b** after semi-preparative purification without (left) and with (right) addition of the authentic non-radioactive reference. In the HPLC setup, the UV detector is in row before the γ-detector with Δt_R of 0.17-0.18 min between both detectors.

Radiolabeling of compound 8a

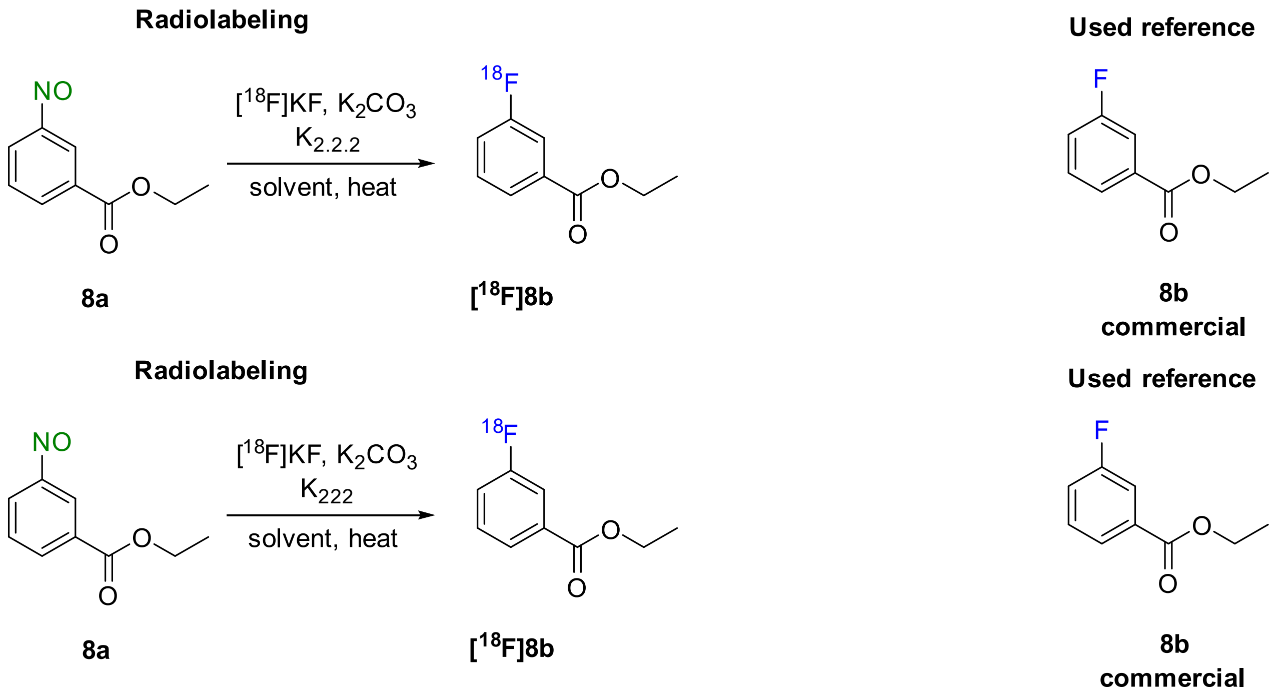

Figure S*26*. Overview for radiolabeling and used references

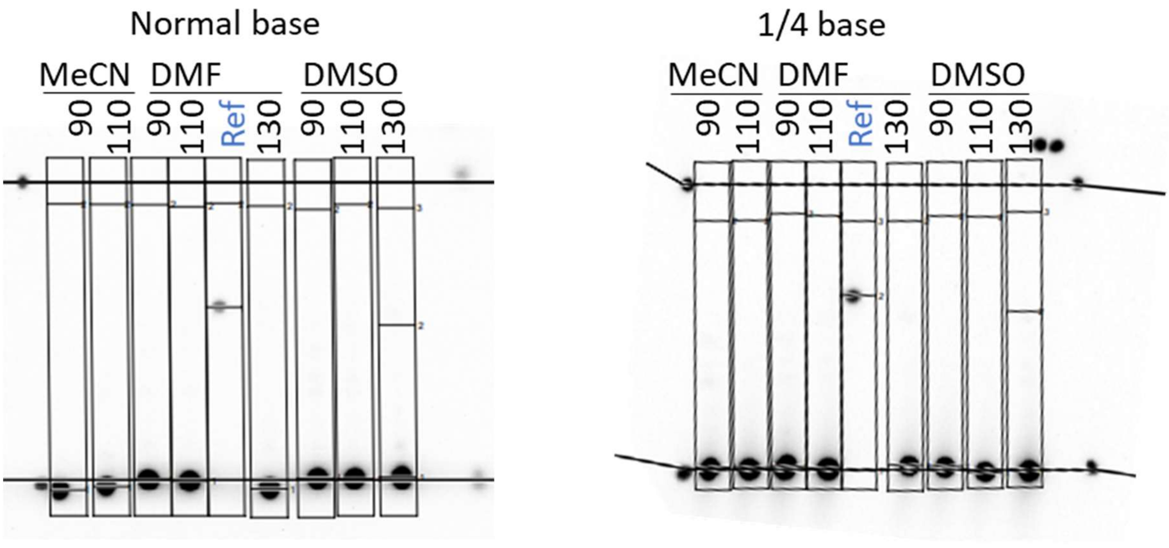

Figure S27. Copy of radio-TLC obtained for ^{18}F-labeling of compound **8a** using normal base (left) and ¼ base (right).

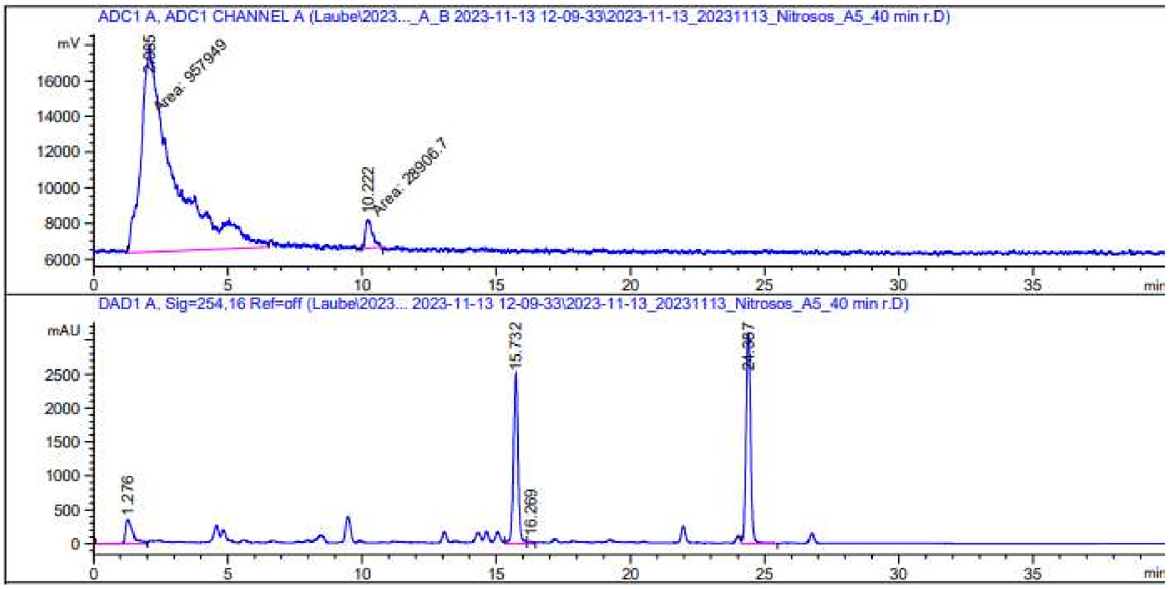

Figure S*28*. Exemplary analytical radio-HPLC chromatogram (System 2, gradient 1; upper panel: signal of gamma-detector; lower panel: UV-signal) of the crude reaction mixture obtained for ^{18}F-labeling of compound **8a** and reaction with normal base at 1*3*0°C in DMF.

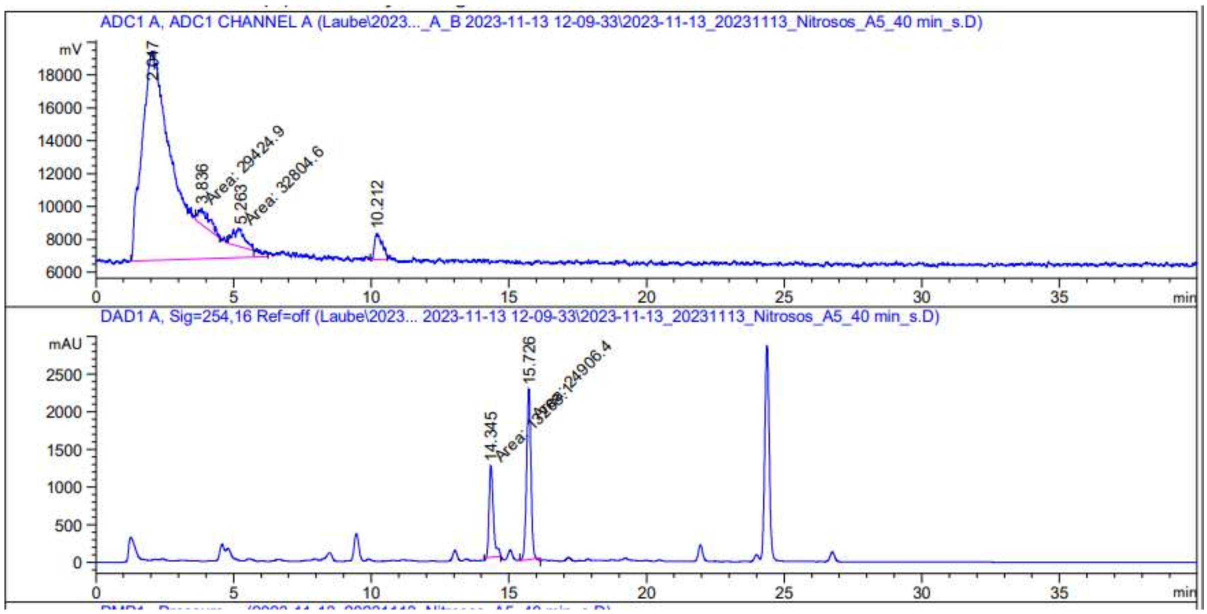

Figure S29. Exemplary analytical radio-HPLC chromatogram (System 2, gradient 1; upper panel: signal of gamma-detector; lower panel: UV-signal) of the crude reaction mixture obtained for ^{18}F-labeling of compound **8a** and reaction with normal base at 130°C in DMF. Authentic non-radioactive reference **8b** was added to the reaction mixture (tR 14.345 min) verifying that **[^{18}F]8b** was not formed in the reaction.

A)				TLC		Set	A	B		
			1	2	3	4	5	6	7	8
	**SR 39/23**		**MeCN**		**DMF**			**DMSO**		
			90	110	90	110	130	90	110	130
A)	**Normal**	Product	0	0	0	0	0	0	0	0,3
		Side Products	0	0	0	0	0	0	0	0
B	**1/4 Base**	Product	0	0	0	0	0	0	0	0,3
		Side Products	0	0	0	0	0	0	0	0

B)				HPLC						
			1	2	3	4	5	6	7	8
	**SR 39/23**		**MeCN**		**DMF**			**DMSO**		
			90	110	90	110	130	90	110	130
A	**Normal**	Product	0	0	0	0	0	0	0	0
		Side Products	3,5	5,4	2	2,7	8,3	1,7	1,8	3,2
B	**1/4 Base**	Product	0	0	0	0	0	0	0	0
		Side Products	0,5	1,6	1	2,3	1,8	0	0	1

Figure S30. Detailed results of optimization experiments for ^{18}F-labeling of compound **8a** obtained by radio-TLC (A) and radio-HPLC (B) analysis.

Radiolabeling of compound 9a

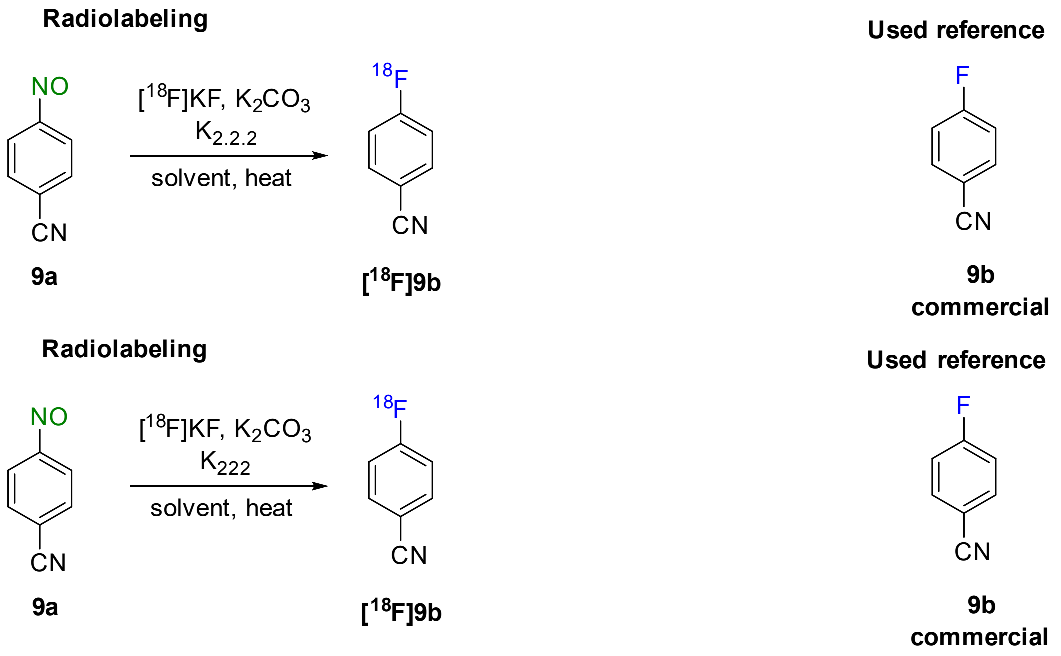

Figure S*31*. Overview for radiolabeling and used references

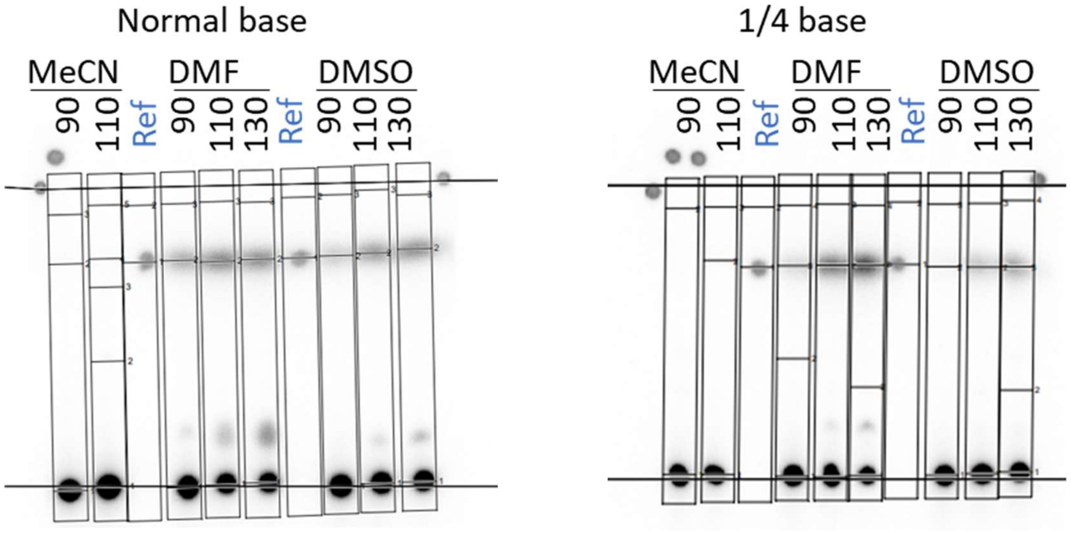

Figure S32. Copy of radio-TLC obtained for ^{18}F-labeling of compound **9a** using normal base (left) and ¼ base (right). Note: Initial inconclusive results for product identification via TLC have been clarified after semi-preparative HPLC by standard addition.

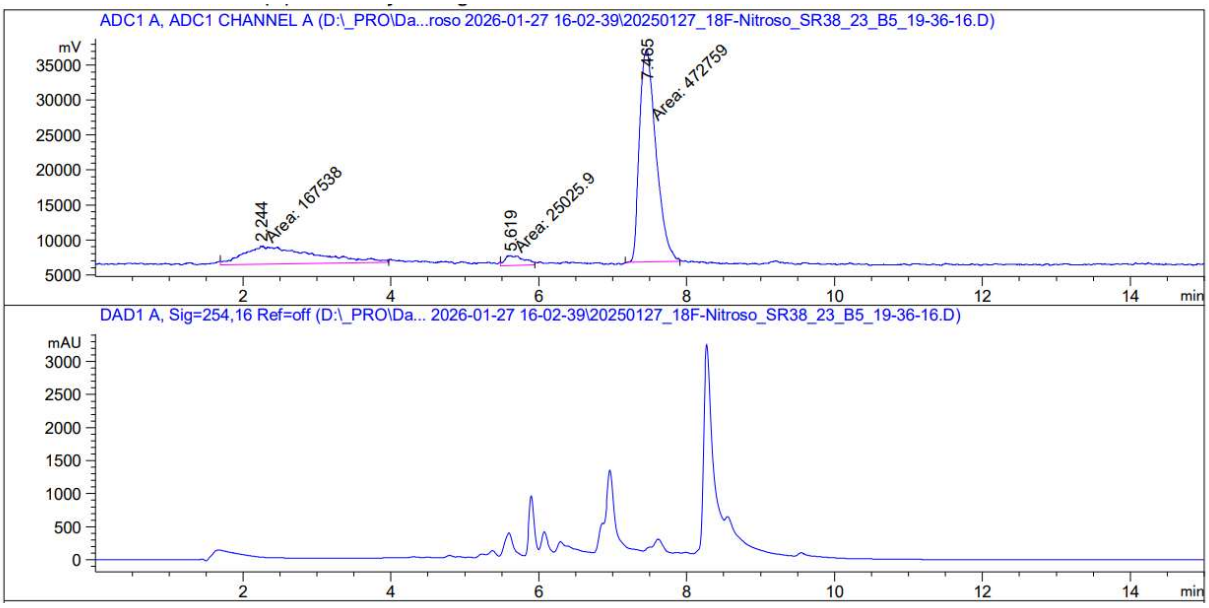

Figure S33. Exemplary analytical radio-HPLC chromatogram (System 2, gradient 3; upper panel: signal of gamma-detector; lower panel: UV-signal) of the crude reaction mixture obtained for ^{18}F-labeling of compound **9a** and reaction with 1/4 base at 130°C in DMF.

A)									
			**TLC**		Set	A	B		
		1	2	3	4	5	6	7	8
**SR 38/23**		**MeCN**		**DMF**			**DMSO**		
		90	110	90	110	130	90	110	130
**Normal**	Product	0,4	0,4	14,5	26,9	26	10	19,2	24,4
	Side Products	0	0	0	0	0	0	0	0
**1/4 Base**	Product	0	0,6	7,1	29,6	45,3	2,7	11,6	15,3
	Side Products	0	0	0	0	0	0	0	0

B)									
			**HPLC**						
		1	2	3	4	5	6	7	8
**SR 38/23**		**MeCN**		**DMF**			**DMSO**		
		90	110	90	110	130	90	110	130
**Normal**	Product	2,9	2,2	30,1	44,1	47,1	14,6	36,3	45,4
	Side Products	0	0	2,2	7,5	17,1	0	1,9	4,5
**1/4 Base**	Product	0	0	9,6	49,9	71,1	2,9	18,4	28,9
	Side Products	0	0	0	0	3,8	0	0	0

Figure S34. Detailed results of optimization experiments for ^{18}F-labeling of compound **9a** obtained by radio-TLC (A) and radio-HPLC (B) analysis.

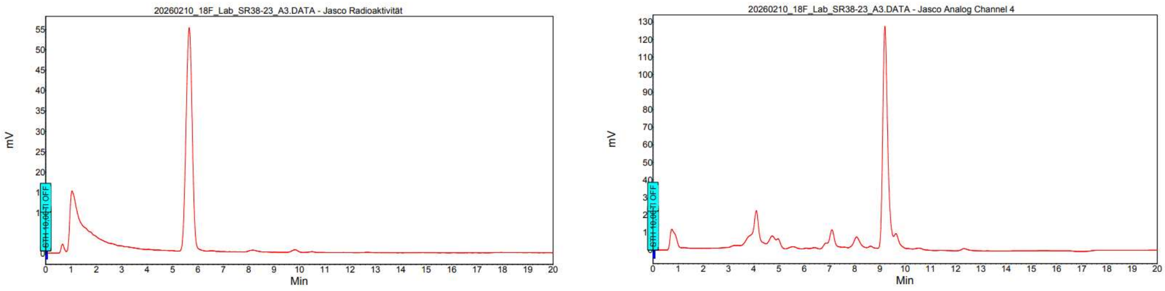

Figure S35. Copy of semi-preparative HPLC chromatograms (System 3; left: signal of gamma-detector; right: UV-signal) obtained for purification of compound **[^{18}F]9b** after labeling of **9a** with [^{18}F]fluoride using 1/4 base, DMF, and 130°C leading to 46% isolated RCY. Product was collected between 5.4 and 6.2 min.

**SR 38-23, 1/4, DMF, 130**	#1	#2	#3	#4	#5	#6	Mean ± SD (n)
RCY [%]	45,7	43,8	44				44.5 ± 0.9 (n=3)

Table S6. Detailed results of RCY and Mean ± SD (n) for the radiosynthesis and isolation of **[^{18}F]9b** using Normal base, DMSO, and 130°C

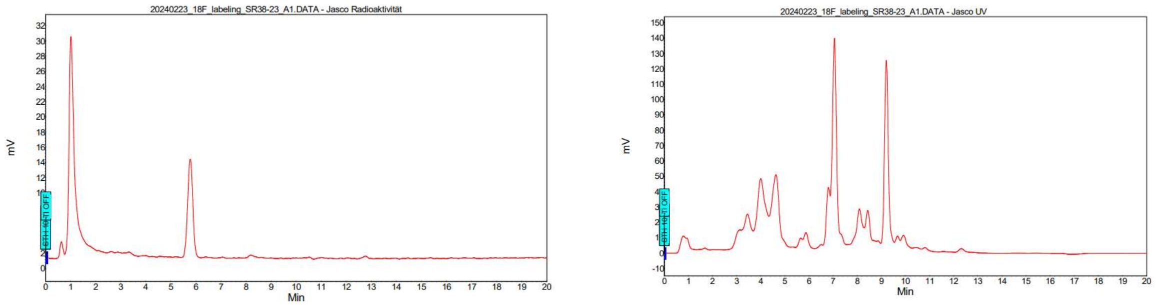

Figure S36. Copy of semi-preparative HPLC chromatograms (System 3; left: signal of gamma-detector; right: UV-signal) obtained for purification of compound **[^{18}F]9b** after labeling of **9a** with [^{18}F]fluoride using normal base, DMSO, and 130°C leading to 18% isolated RCY. Product was collected between 5.5and 6.3 min.

**SR 38-23, N, DMSO, 130**	#1	#2	#3	#4	#5	#6	Mean ± SD (n)
RCY [%]	18	27	25				23.3 ± 3.9 (n=3)

Table S7. Detailed results of RCY and Mean ± SD (n) for the radiosynthesis and isolation of **[^{18}F]9b** using Normal base, DMSO, and 130°C

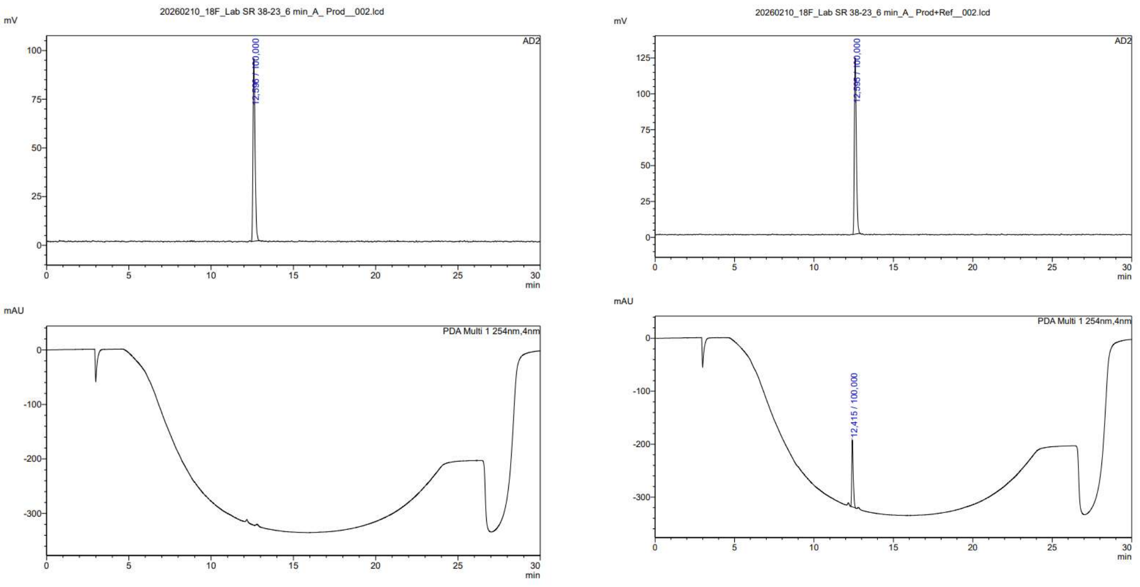

Figure S37. Copy of analytical HPLC chromatograms (System 1, gradient 3; upper panel: signal of gamma-detector; lower panel: UV-signal) obtained for compound **[^{18}F]9b** from the reaction using ¼ base, DMF at 130°C and after semi-preparative purification without (left) and with (right) addition of the authentic non-radioactive reference. In the HPLC setup, the UV detector is in row before the γ-detector with Δt_R of 0.17-0.18 min between both detectors.

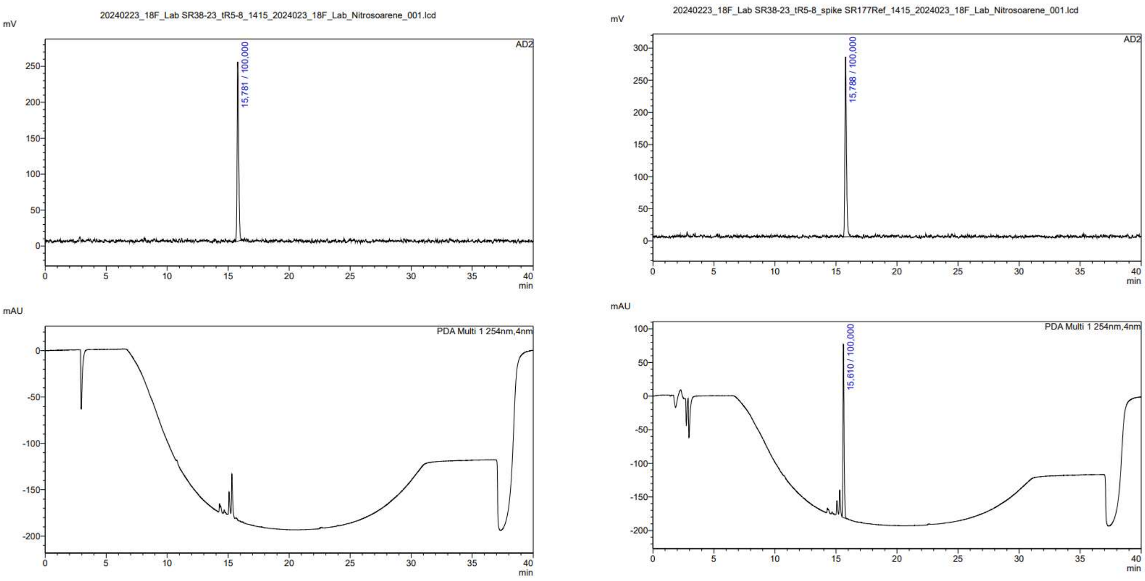

Figure S38. Copy of analytical HPLC chromatograms (System 1, gradient 1; upper panel: signal of gamma-detector; lower panel: UV-signal) obtained for compound **[^{18}F]9b** from the reaction using normal base, DMSO at 130°C and after semi-preparative purification without (left) and with (right) addition of the authentic non-radioactive reference. In the HPLC setup, the UV detector is in row before the γ-detector with Δt_R of 0.17-0.18 min between both detectors.

Radiolabeling of compound 10a

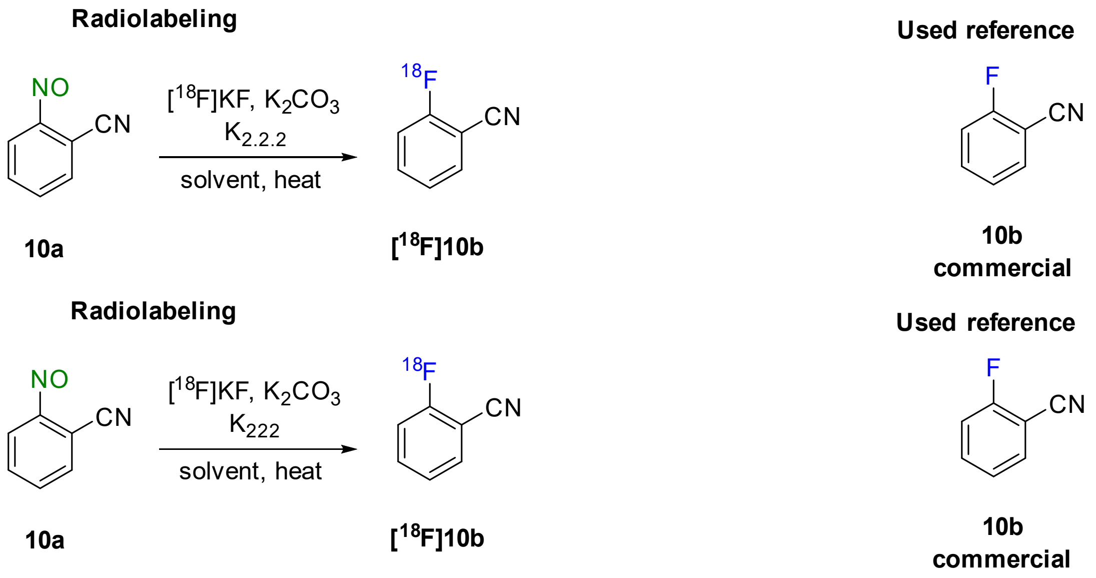

Figure S*39*. Overview for radiolabeling and used references

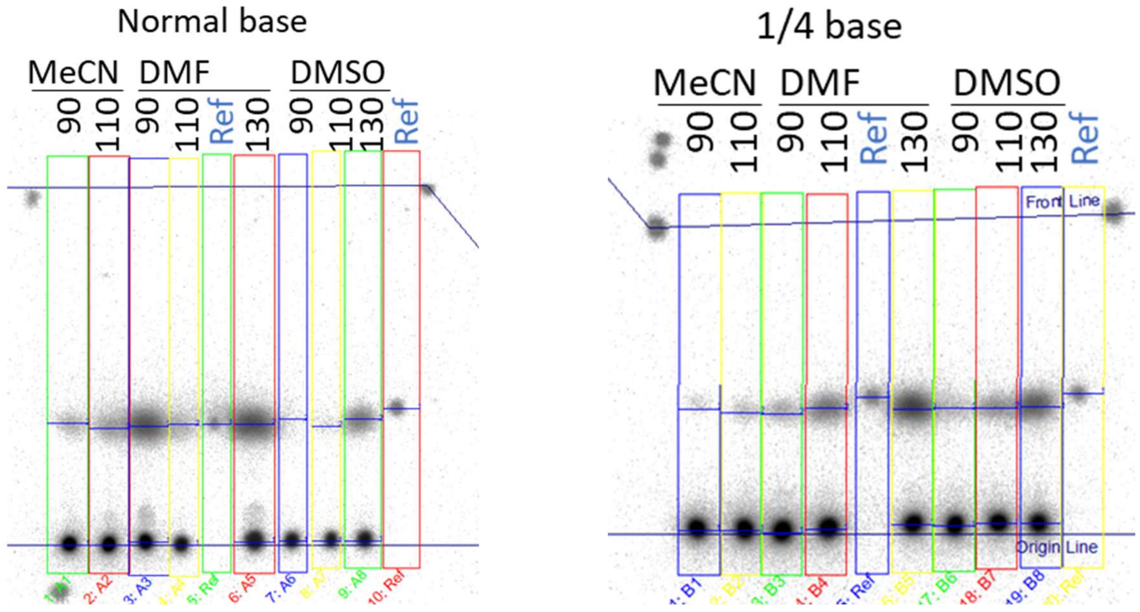

Figure S40. Copy of radio-TLC obtained for ^{18}F-labeling of compound **10a** using normal base (left) and ¼ base (right).

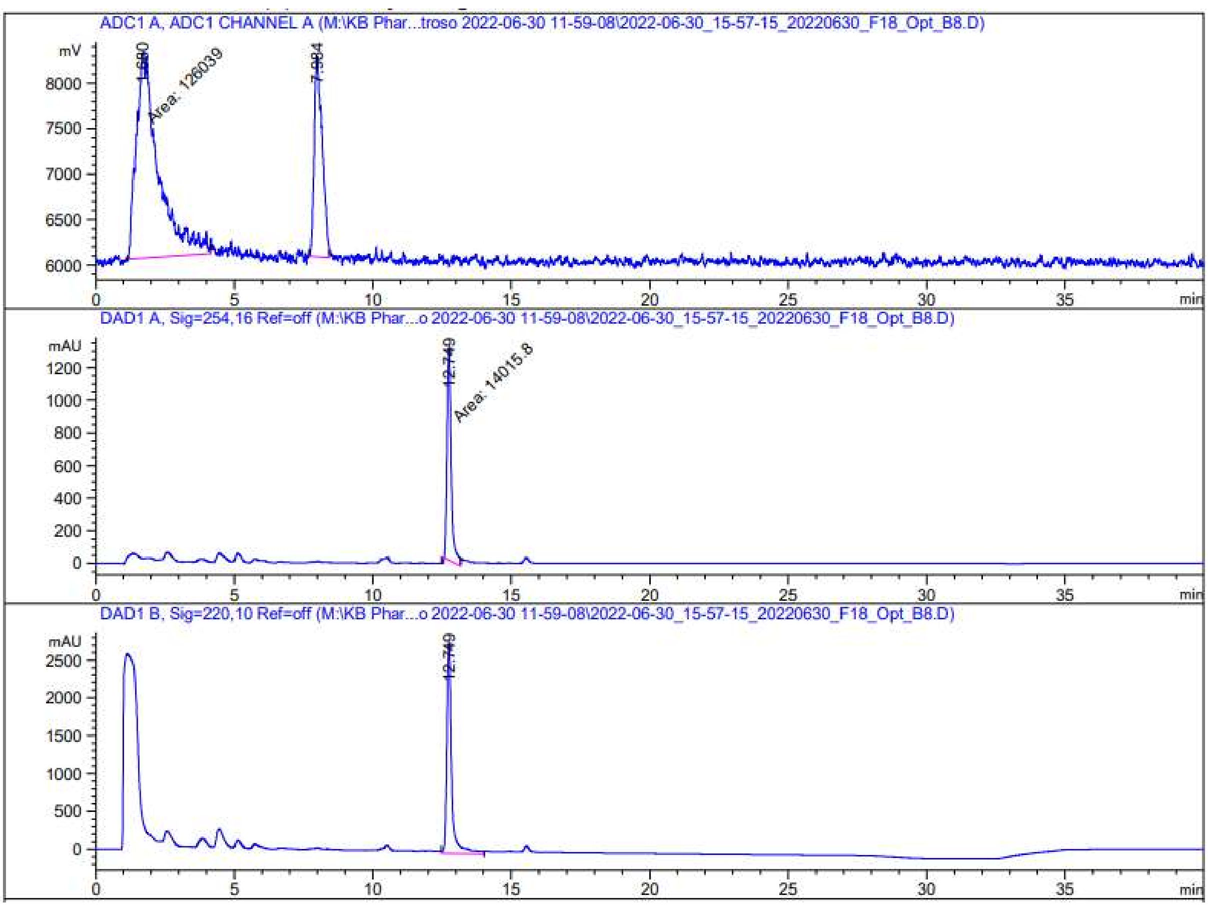

Figure S41. Exemplary analytical radio-HPLC chromatogram (System 2, gradient 1; upper panel: signal of gamma-detector; middle and lower panel: UV-signal at 254 and 220 nm, respectively) of the crude reaction mixture obtained for ^{18}F-labeling of compound **10a** and reaction with 1/4 base at 130°C in DMSO.

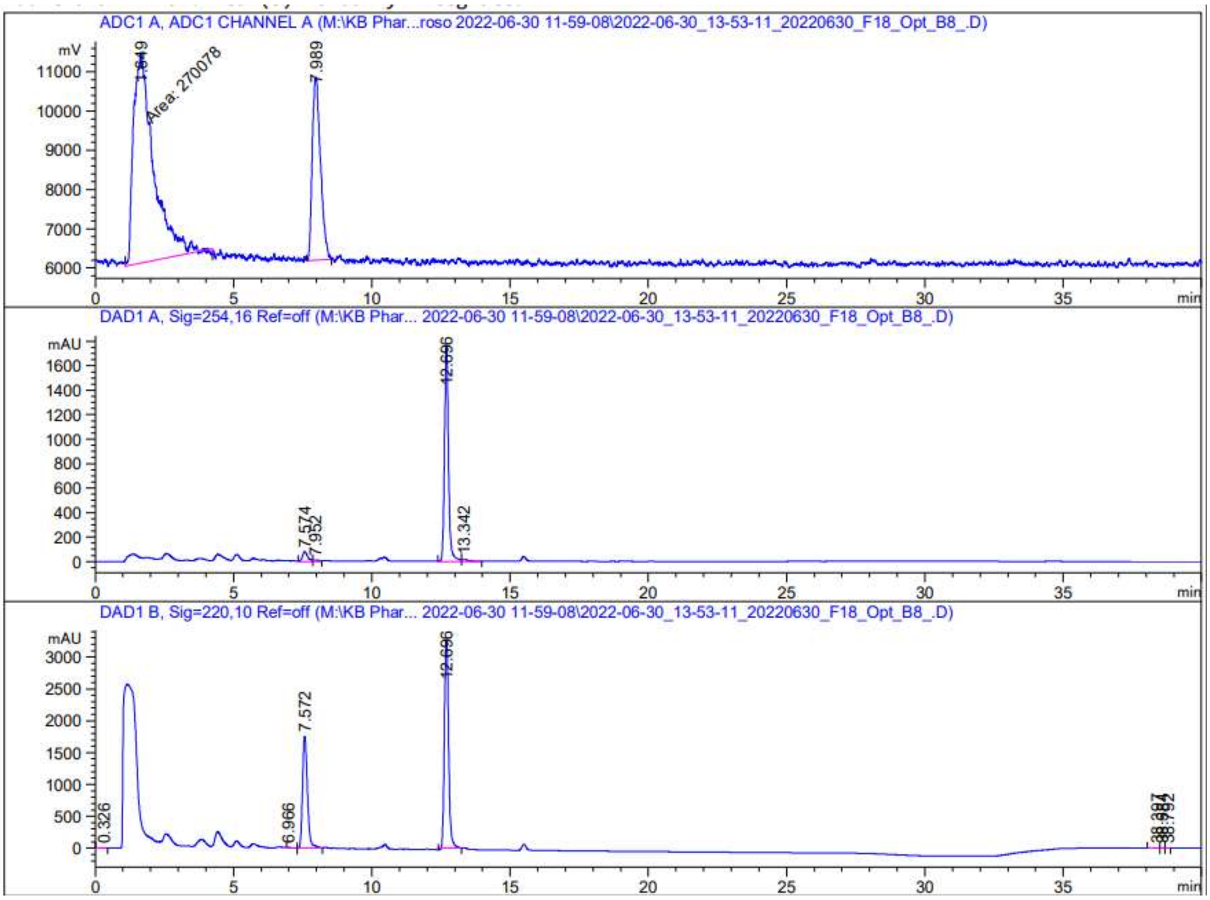

Figure S42. Exemplary analytical radio-HPLC chromatogram (System 2, gradient 1; upper panel: signal of gamma-detector; middle and lower panel: UV-signal at 254 and 220 nm, respectively) of the crude reaction mixture spiked with authentic non-radioactive reference (t_R 7.572 min) obtained for ^{18}F-labeling of compound **10a** and reaction with 1/4 base at 130°C in DMSO.

A)				**TLC**		Set	A	B		
			1	2	3	4	5	6	7	8
	**SRIM1F2**		**MeCN**		**DMF**			**DMSO**		
			90	110	90	110	130	90	110	130
	**Normal**	Product	2,9	7,4	29,7	11,8	45,3	6,3	4,4	21,8
		Side Products	0	0	0	0	0	0	0	0
	**1/4 Base**	Product	0,8	1,5	2,3	7,9	28,3	4,3	7,2	19,7
		Side Products	0	0	0	0	0	0	0	0

B)				**HPLC**						
			1	2	3	4	5	6	7	8
	**SRIMF2**		**MeCN**		**DMF**			**DMSO**		
			90	110	90	110	130	90	110	130
	**Normal**	Product	n.d.	n.d.	n.d.	n.d.	64	n.d.	n.d.	n.d.
		Side Products								
	**1/4 Base**	Product	n.d.	n.d.	n.d.	n.d.	39	n.d.	n.d.	26
		Side Products								

Figure S43. Detailed results of optimization experiments for ^{18}F-labeling of compound **10a** obtained by radio-TLC (A) and radio-HPLC (B) analysis. n.d. not determined.

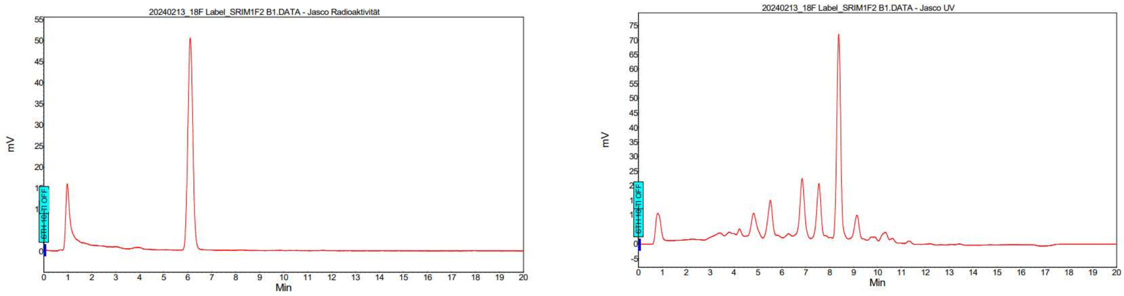

Figure S44. Copy of semi-preparative HPLC chromatograms (System 3; left: signal of gamma-detector; right: UV-signal) obtained for purification of compound **[^{18}F]10b** after labeling of **10a** with [^{18}F]fluoride under optimized conditions. Product was collected between 5.9and 7.0 min.

	#1	#2	#3	#4	#5	#6	Mean ± SD (n)
RCY [%]	34	52	60				48.7 ± 10.9 (n=3)

Table S8. Detailed results of RCY and Mean ± SD (n) for the radiosynthesis and isolation of **[^{18}F]10b**.

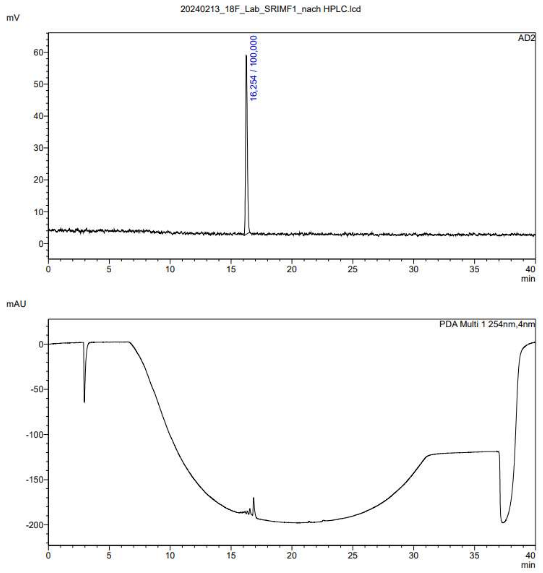

Figure S45. Copy of analytical HPLC chromatograms (System 1; upper panel: signal of gamma-detector; lower panel: UV-signal) obtained for compound **[^{18}F]10b** after semi-preparative purification without (left) addition of the authentic non-radioactive reference. In the HPLC setup, the UV detector is in row before the γ-detector with Δt_R of 0.17-0.18 min between both detectors.

Radiolabeling of compound 11a

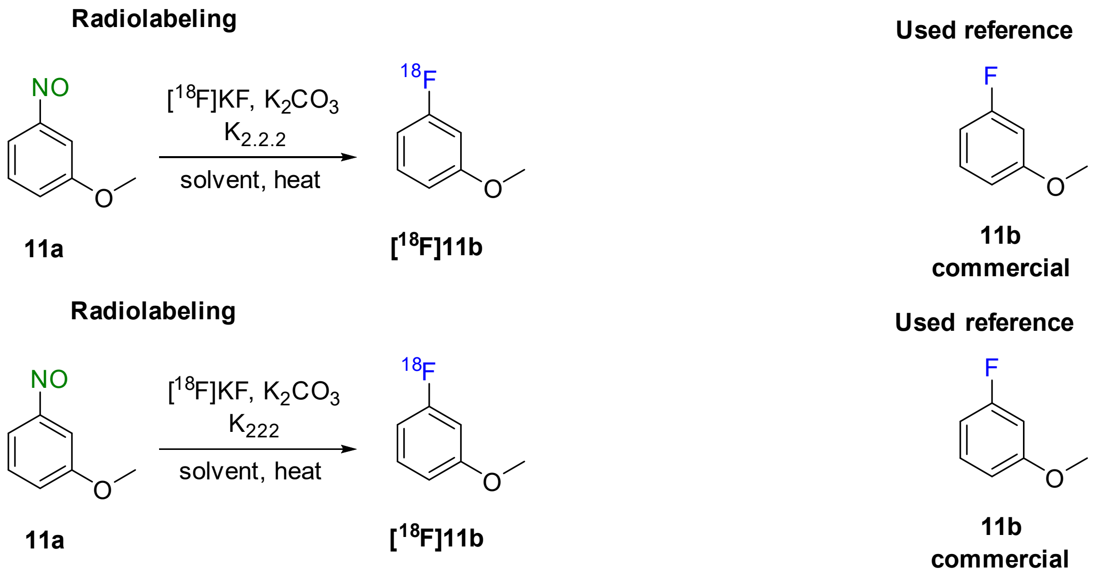

Figure S*46*. Overview for radiolabeling and used references

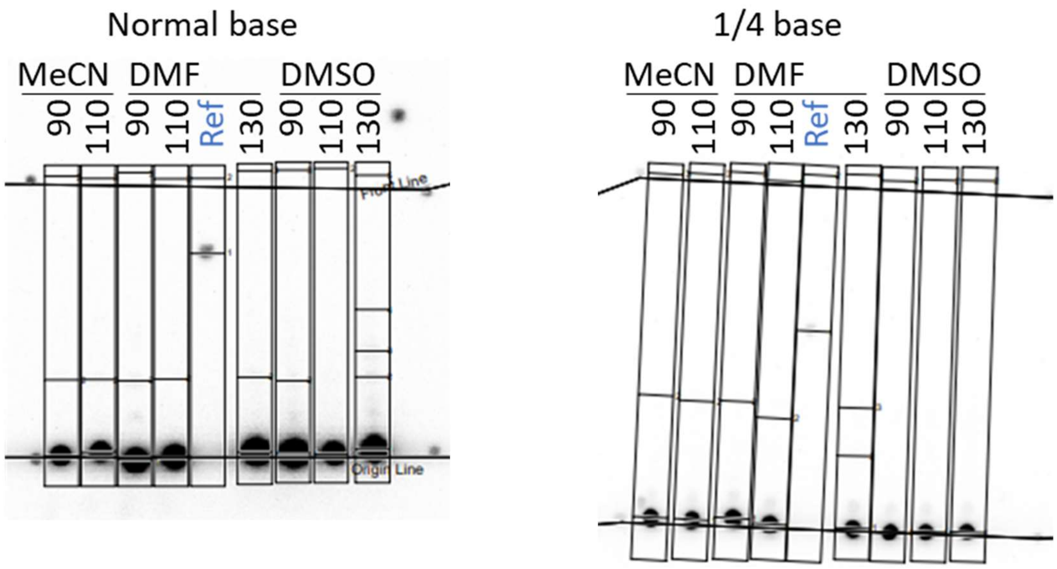

Figure S47. Copy of radio-TLC obtained for ^{18}F-labeling of compound **11a** using normal base (left) and ¼ base (right).

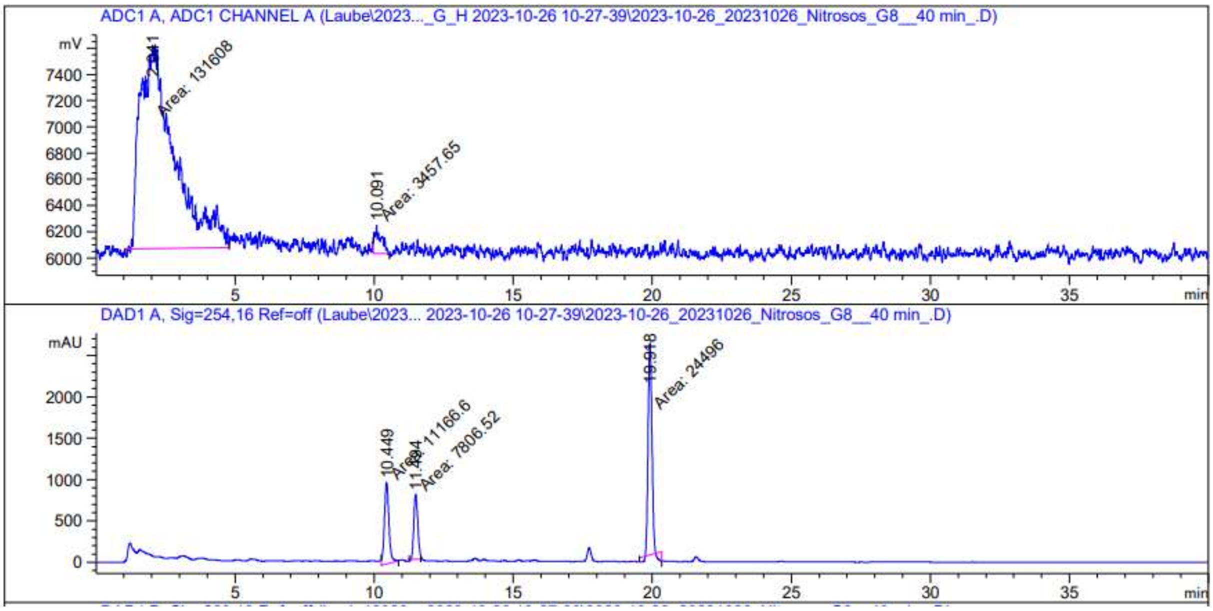

Figure S48. Exemplary analytical radio-HPLC chromatogram (System 2, gradient 1; upper panel: signal of gamma-detector; lower panel: UV-signal) of the crude reaction mixture spiked with authentic non-radioactive reference (t_R 11.494 min) obtained for ^{18}F-labeling of compound **11a** and reaction with normal base at 130°C in DMSO.

A)				TLC		Set	G	H		
			1	2	3	4	5	6	7	8
	**SR 33/23**		**MeCN**		**DMF**			**DMSO**		
			90	110	90	110	130	90	110	130
	**Normal**	Product	0	0	0	0	0	0	0	0
		Side Products	0,3	0,4	0,4	0,2	0,3	0,3	0,4	1,3
	**1/4 Base**	Product	0	0	0	0	0	0	0	0
		Side Products	0,1	0,3	0,1	0,1	0	0,4	0	0
B)				HPLC						
			1	2	3	4	5	6	7	8
	**SR 33/23**		**MeCN**		**DMF**			**DMSO**		
			90	110	90	110	130	90	110	130
	**Normal**	Product	0	0	0	0	0	0	0	0
		Side Products	0,8	0,4	1,2	1,1	0	0,9	1,7	2,2
	**1/4 Base**	Product	0	0	0	0	0	0	0	0
		Side Products	0	0	0	0	0	0	0	0

Figure S49. Detailed results of optimization experiments for ^{18}F-labeling of compound **11a** obtained by radio-TLC (A) and radio-HPLC (B) analysis. Side Prod. Side Product(s); n.d. not determined.

Radiolabeling of compound 12a

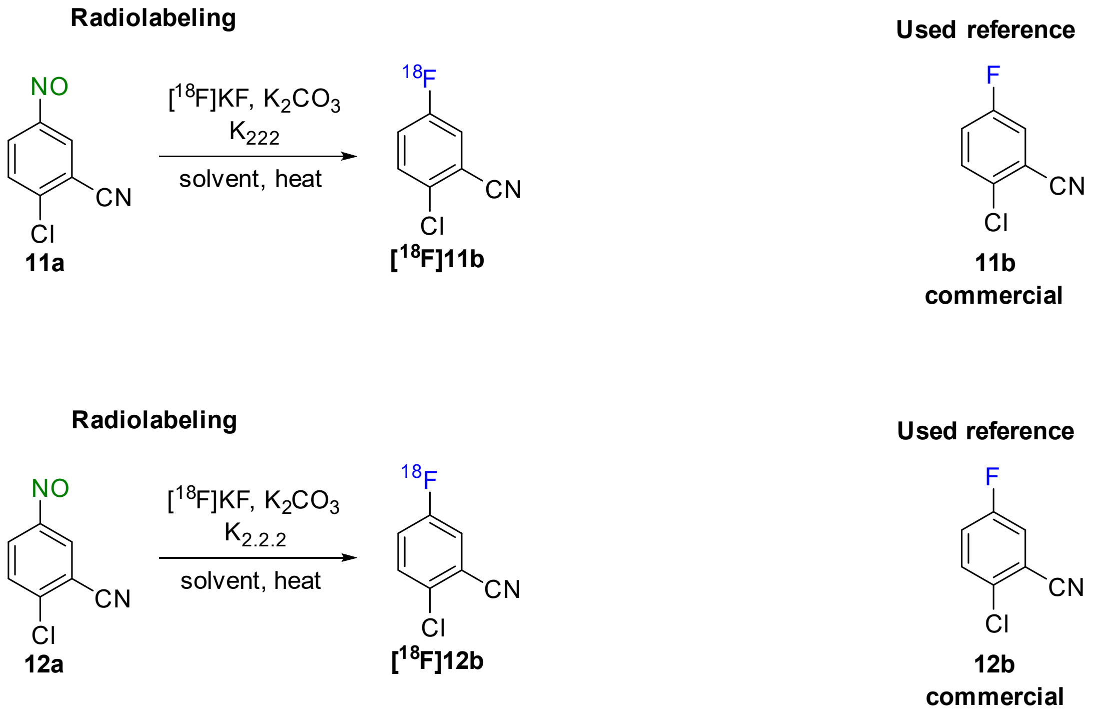

Figure S*50*. Overview for radiolabeling and used references

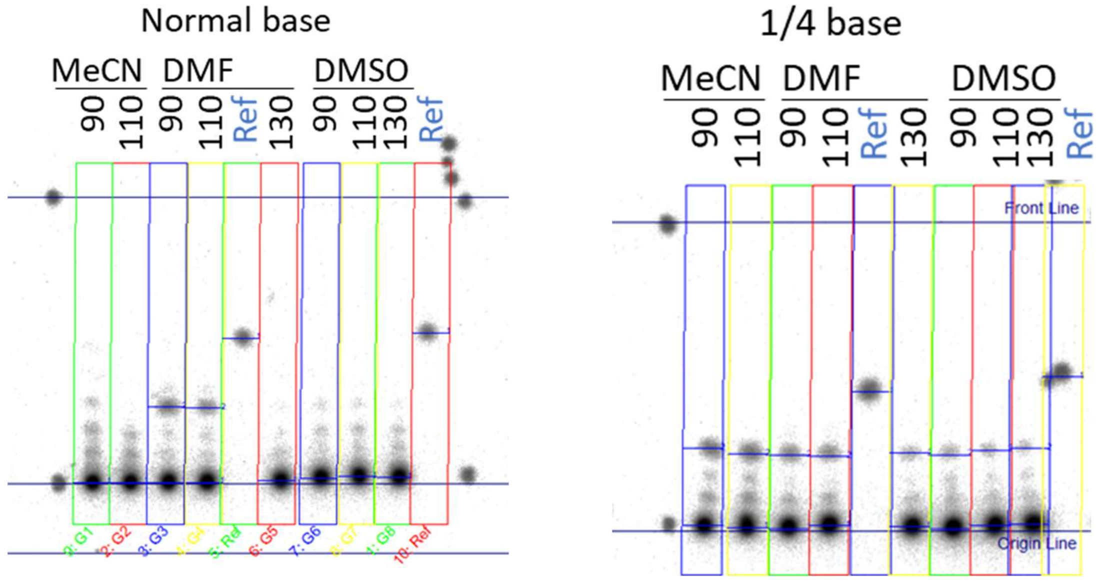

Figure S51. Copy of radio-TLC obtained for ^{18}F-labeling of compound **12a** using normal base (left) and ¼ base (right).

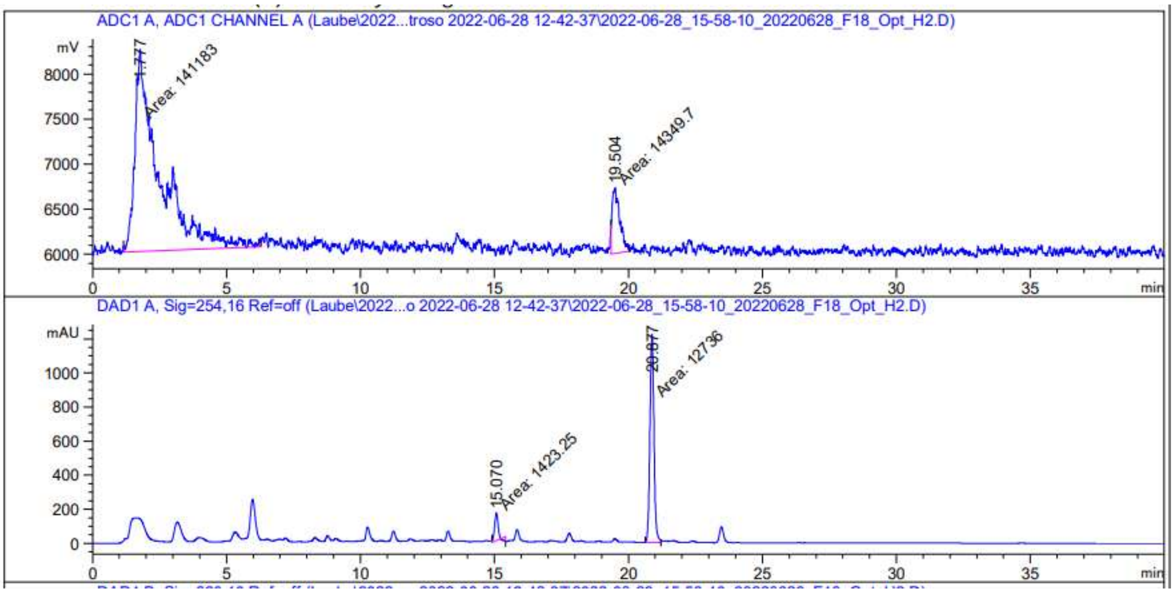

Figure S52. Exemplary analytical radio-HPLC chromatogram (System 2, gradient 1; upper panel: signal of gamma-detector; lower panel: UV-signal) of the crude reaction mixture obtained for ^{18}F-labeling of compound **12a** and reaction with 1/4 base at 110°C in MeCN.

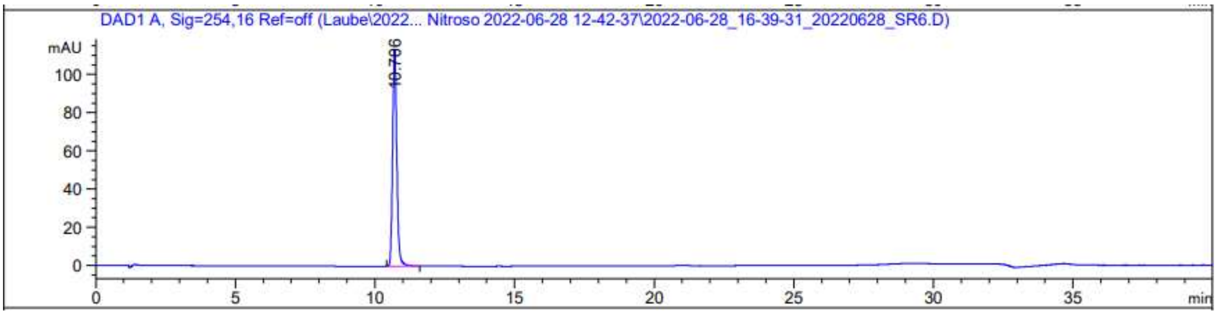

Figure S53. Analytical HPLC chromatogram (System 2, gradient 1) of the authentic non-radioactive reference **12b** (t_R 10.706 min).

A)			TLC		Set	G	H		
			1	2	3	4	5	6	7	8

	SRIM6F1		1	2	3	4	5	6	7	8
			**MeCN**		**DMF**			**DMSO**		
			90	110	90	110	130	90	110	130
	**Normal**	Product	0	0	0	0	0	0	0	0
		Side Products	0	0	6,4	3,6	0	0	0	0
	**1/4 Base**	Product	0	0	0	0	0	0	0	0
		Side Products	6,7	6,7	4,3	3,2	1,4	1,1	1,1	0,9

B)			HPLC			G	H		

	SRIM6F1		1	2	3	4	5	6	7	8
			**MeCN**		**DMF**			**DMSO**		
			90	110	90	110	130	90	110	130
	**Normal**	Product	n.d.	0	n.d.	n.d.	n.d.	n.d.	n.d.	n.d.
		Side Products		0						
	**1/4 Base**	Product	n.d.	0	n.d.	n.d.	n.d.	n.d.	n.d.	n.d.
		Side Products		9						

Figure S54. Detailed results of optimization experiments for ^{18}F-labeling of compound **12a** obtained by radio-TLC (A) and radio-HPLC (B) analysis. n.d. not determined.

Radiolabeling of compound 13a

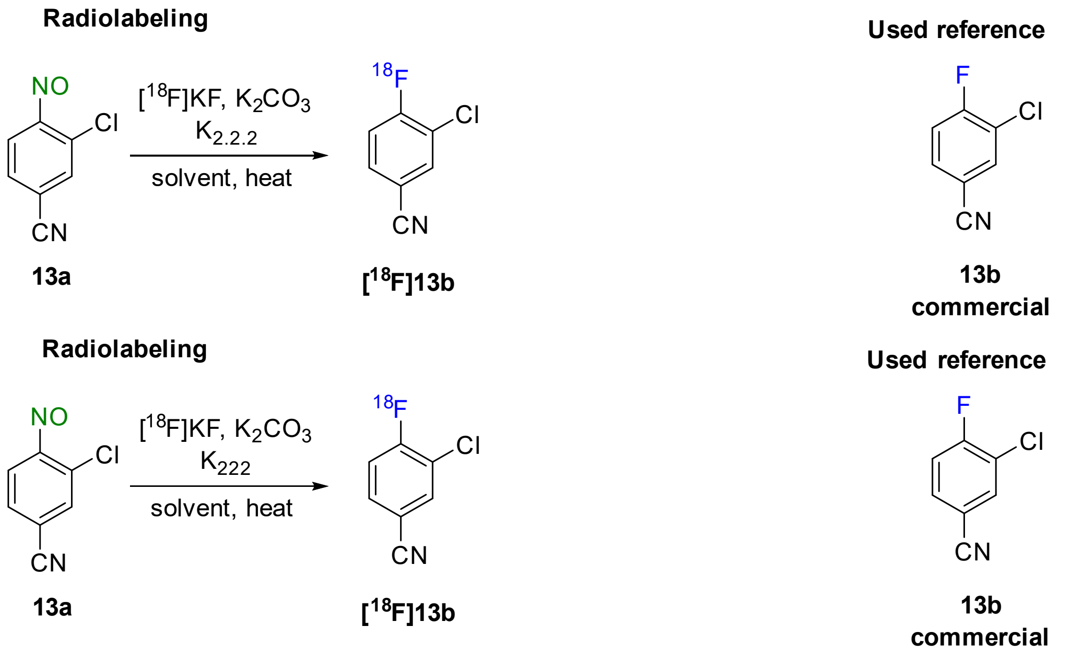

Figure S55. Overview for radiolabeling and used references

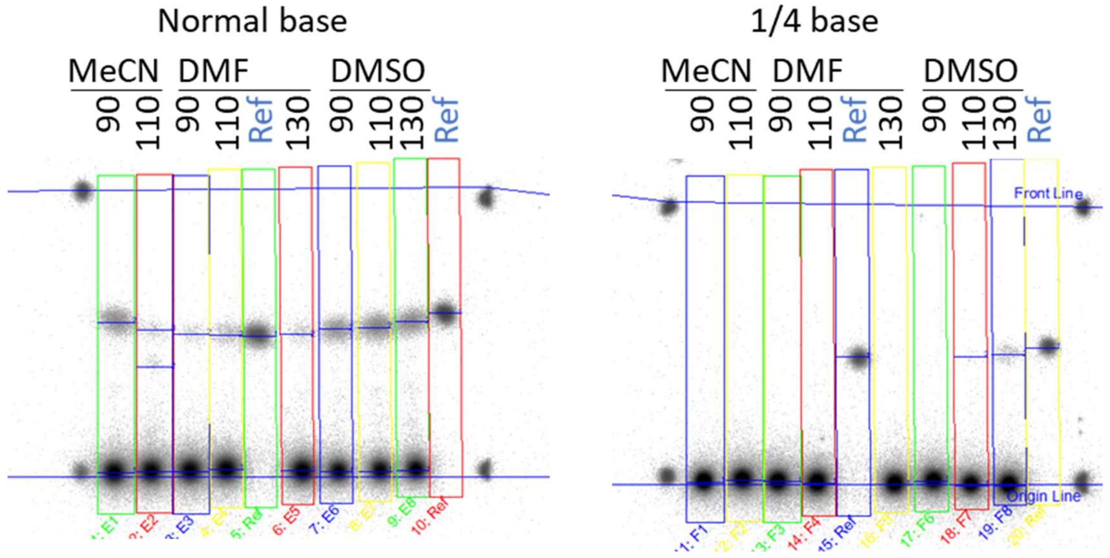

Figure S56. Copy of radio-TLC obtained for ^{18}F-labeling of compound **13a** using normal base (left) and ¼ base (right).

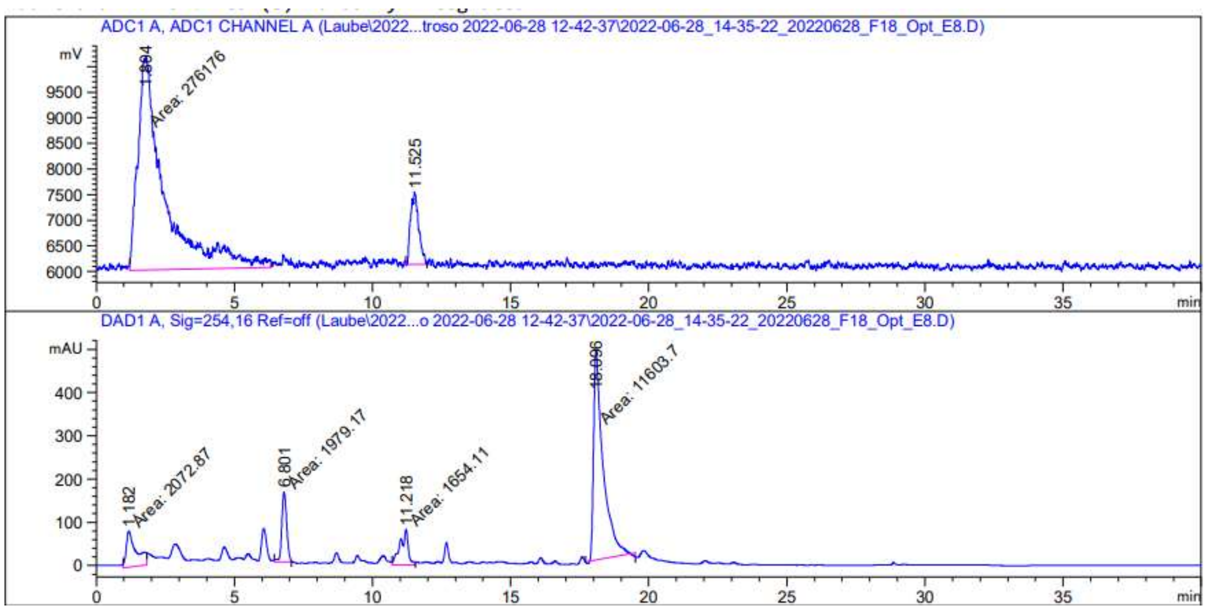

Figure S57. Exemplary analytical radio-HPLC chromatogram (System 2, gradient 1; upper panel: signal of gamma-detector; lower panel: UV-signal) of the crude reaction mixture obtained for ^{18}F-labeling of compound **13a** and reaction with normal base at 130°C in DMSO.

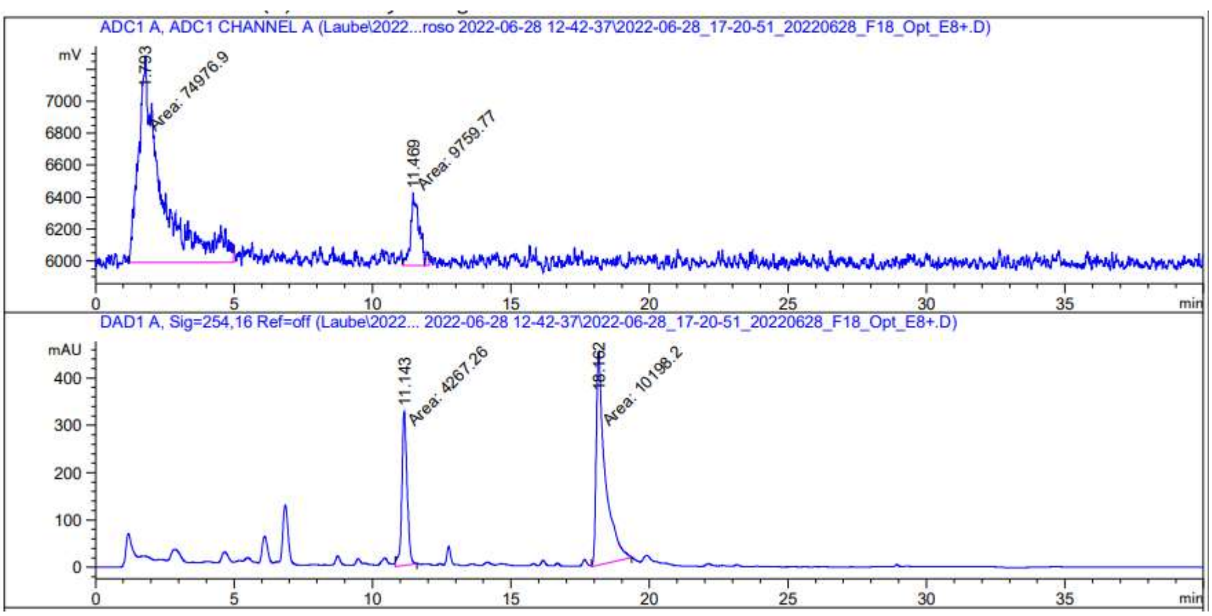

Figure S58. Exemplary analytical radio-HPLC chromatogram (System 2, gradient 1; upper panel: signal of gamma-detector; lower panel: UV-signal) of the crude reaction mixture spiked with authentic non-radioactive reference (t_R 11.143 min) obtained for ^{18}F-labeling of compound **13a** and reaction with normal base at 130°C in DMSO.

A)				TLC		Set	E	F		
			1	2	3	4	5	6	7	8
	**SRIM4BF1**		**MeCN**		**DMF**			**DMSO**		
			90	110	90	110	130	90	110	130
	**Normal**	Product	5,4	0,4	0,8	1,8	0,8	5,6	8,2	6,8
		Side Products	0	0	0	0	0	0	0	0
	**1/4 Base**	Product	0	0	0	0	0	0	0	0
		Side Products	0	0	0	0	0	0	0	0

B)				HPLC			E	F		
			1	2	3	4	5	6	7	8
	**SRIM4BF1**		**MeCN**		**DMF**			**DMSO**		
			90	110	90	110	130	90	110	130
	**Normal**	Product	10,2	n.d.	n.d.	n.d.	n.d.	n.d.	n.d.	9,4
		Side Products	0							0
	**1/4 Base**	Product	n.d.	n.d.	n.d.	n.d.	n.d.	n.d.	n.d.	n.d.
		Side Products								

Figure S59. Detailed results of optimization experiments for ^{18}F-labeling of compound **13a** obtained by radio-TLC (A) and radio-HPLC (B) analysis. n.d. not determined.

Radiolabeling of compound 14a

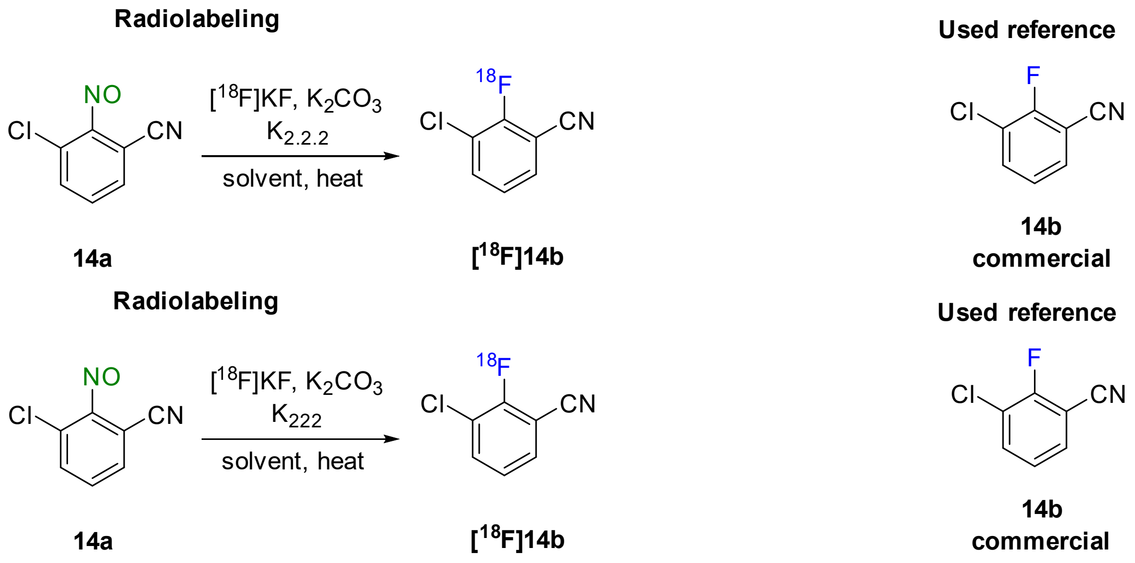

Figure S*60*. Overview for radiolabeling and used references

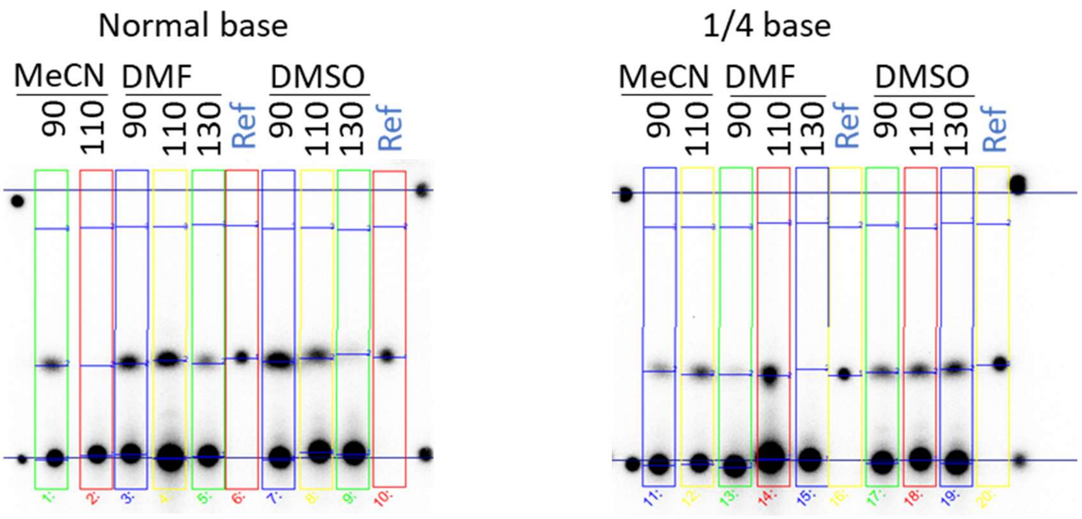

Figure S61. Copy of radio-TLC obtained for ^{18}F-labeling of compound **14a** using normal base (left) and ¼ base (right).

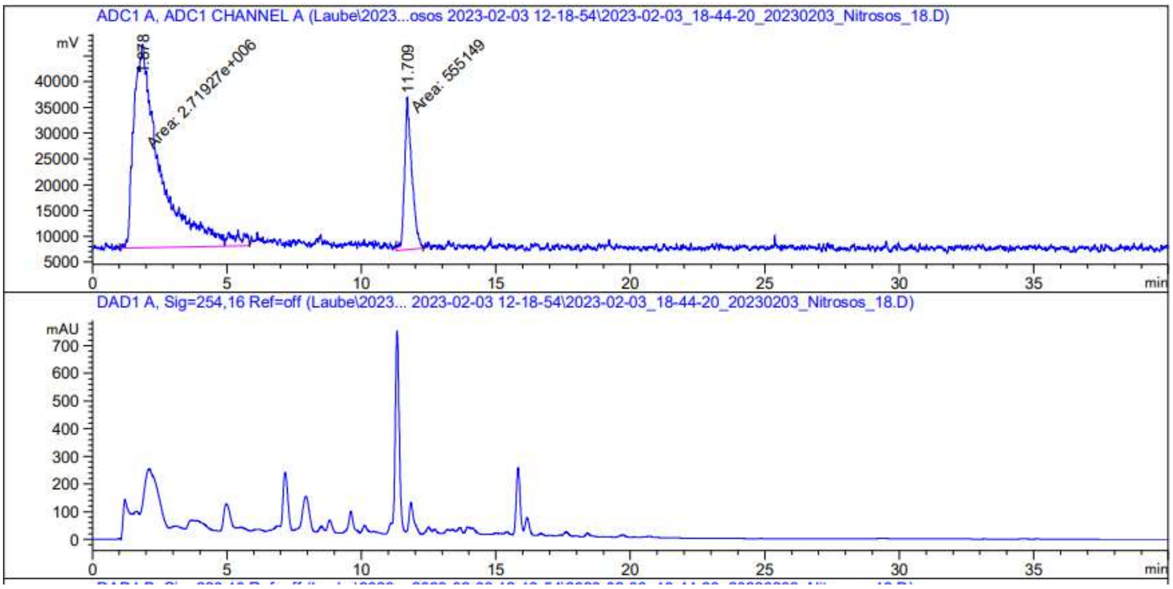

Figure S62. Exemplary analytical radio-HPLC chromatogram (System 2, gradient 1; upper panel: signal of gamma-detector; lower panel: UV-signal) of the crude reaction mixture obtained for ^{18}F-labeling of compound **14a** and reaction with normal base at 110°C in DMSO.

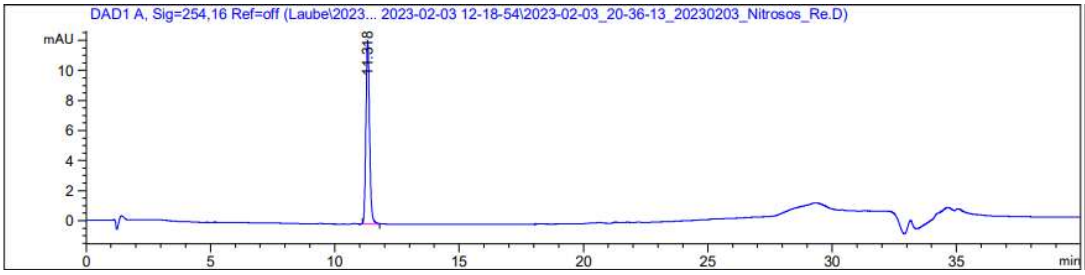

Figure S63. Analytical HPLC chromatogram (System 2, gradient 1) of the authentic non-radioactive reference **14b** (tR 11.318 min).

A)				TLC		Set	N	O		
			1	2	3	4	5	6	7	8
	**SR212F2/22**		**MeCN**		**DMF**			**DMSO**		
			90	110	90	110	130	90	110	130
	**Normal**	Product	18,4	1,4	14,8	10,8	7,7	22,1	13,7	3,6
		Side Products								
	**1/4 Base**	Product	8,1	17,8	3,2	8,9	0,7	9,1	11,2	11,7
		Side Products								

B)				HPLC						
			1	2	3	4	5	6	7	8
	**SR212F2/22**		**MeCN**			**DMF**			**DMSO**	
			90	110	90	110	130	90	110	130
	**Normal**	Product	n.d.	n.d.	18,2	n.d.	n.d.	32,2	17,7	n.d.
		Side Products								
	**1/4 Base**	Product	n.d.	16,2	n.d.	n.d.	n.d.	n.d.	13,4	14,8
		Side Products								

Figure S64. Detailed results of optimization experiments for ^{18}F-labeling of compound **14a** obtained by radio-TLC (A) and radio-HPLC (B) analysis. n.d. not determined.

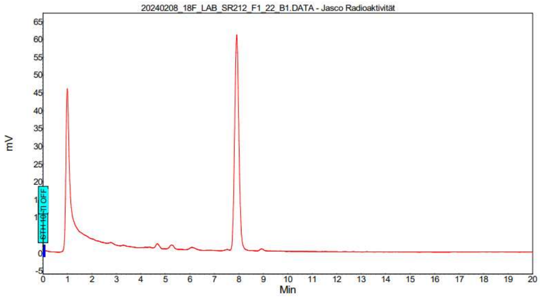

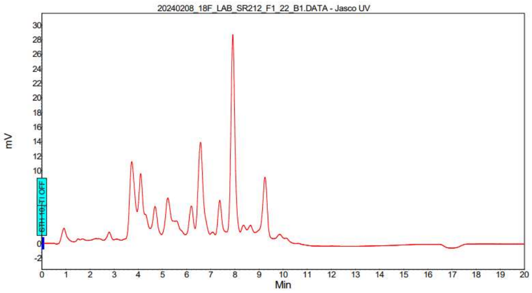

Figure S65. Copy of semi-preparative HPLC chromatograms (System 3; left: signal of gamma-detector; right: UV-signal) obtained for purification of compound **[^{18}F]14b** after labeling of **14a** with [^{18}F]fluoride under optimized conditions. Product was collected between 7.7 and 8.4 min.

	#1	#2	#3	#4	#5	#6	Mean ± SD (n)
RCY [%]	23	27	19				23.0 ± 3.3 (n=3)

Table S9. Detailed results of RCY and Mean ± SD (n) for the radiosynthesis and isolation of **[^{18}F]14b**.

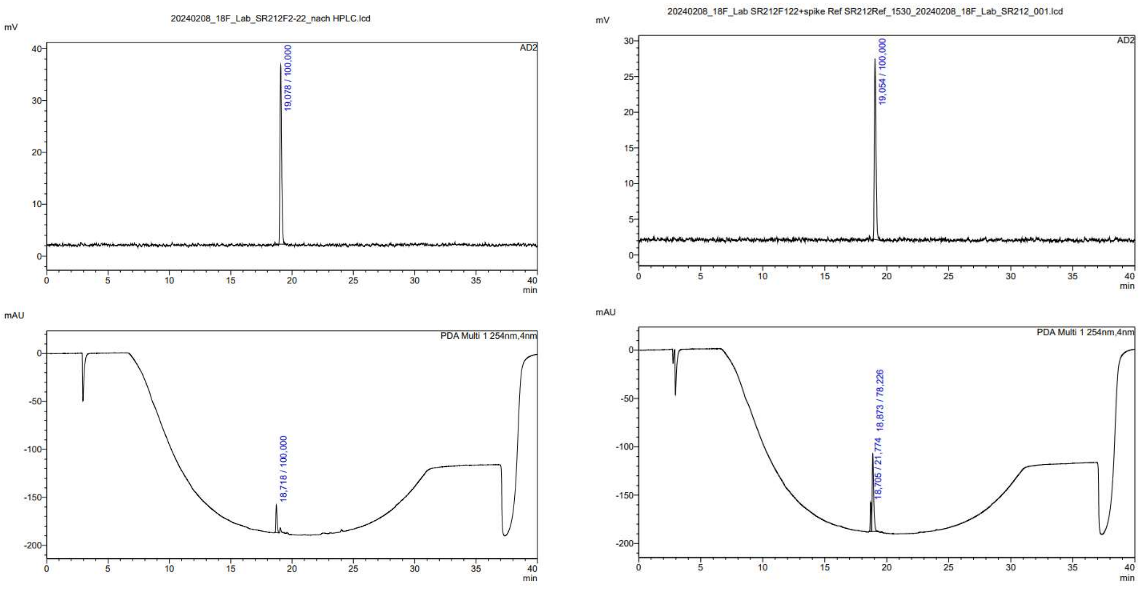

Figure S66. Copy of analytical HPLC chromatograms (System 1; upper panel: signal of gamma-detector; lower panel: UV-signal) obtained for compound **[^{18}F]14b** after semi-preparative purification without (left) and with (right) addition of the authentic non-radioactive reference. In the HPLC setup, the UV detector is in row before the γ-detector with Δt_R of 0.17-0.18 min between both detectors. The UV signal of the impurity at with t_R 18.718 min (left panel) is integrated to demonstrate additional signal observed after standard addition (right panel).

Radiolabeling of compound 15a

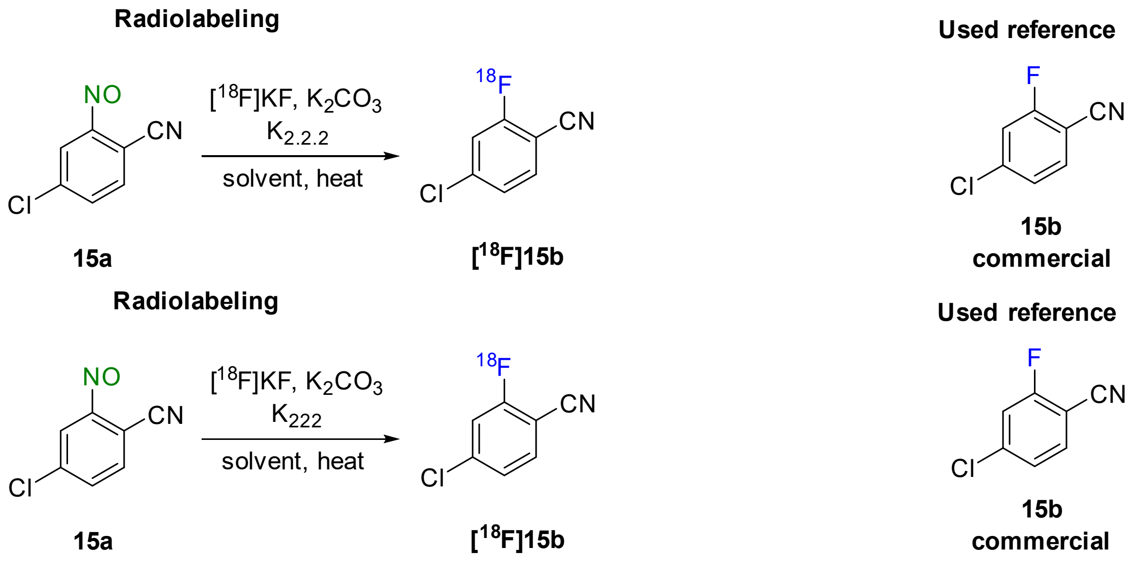

Figure S*67*. Overview for radiolabeling and used references

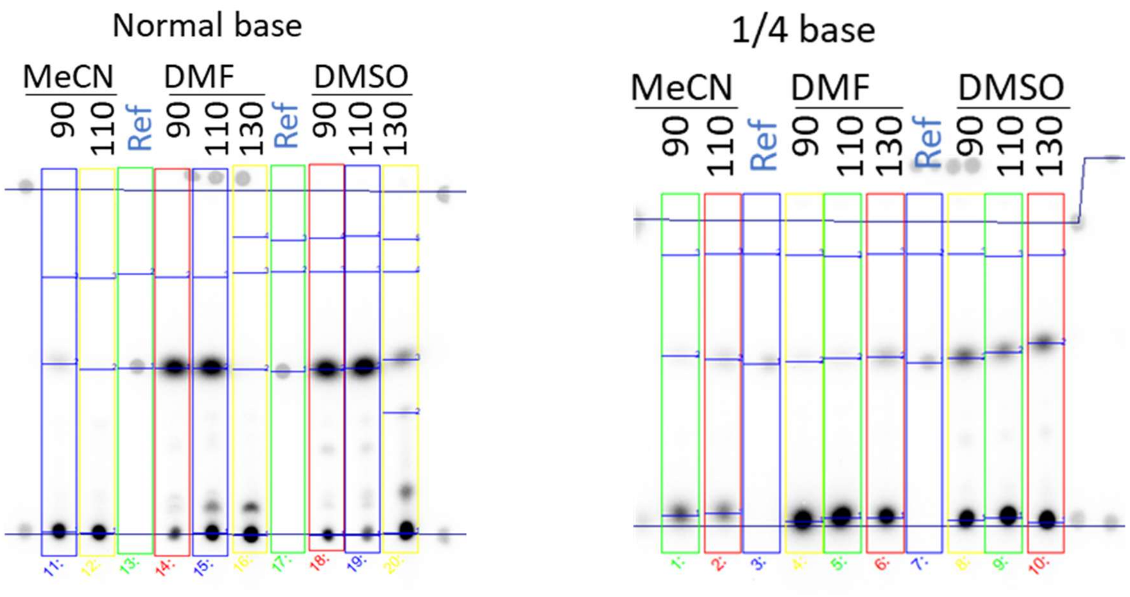

Figure S68. Copy of radio-TLC obtained for ^{18}F-labeling of compound **15a** using normal base (left) and ¼ base (right).

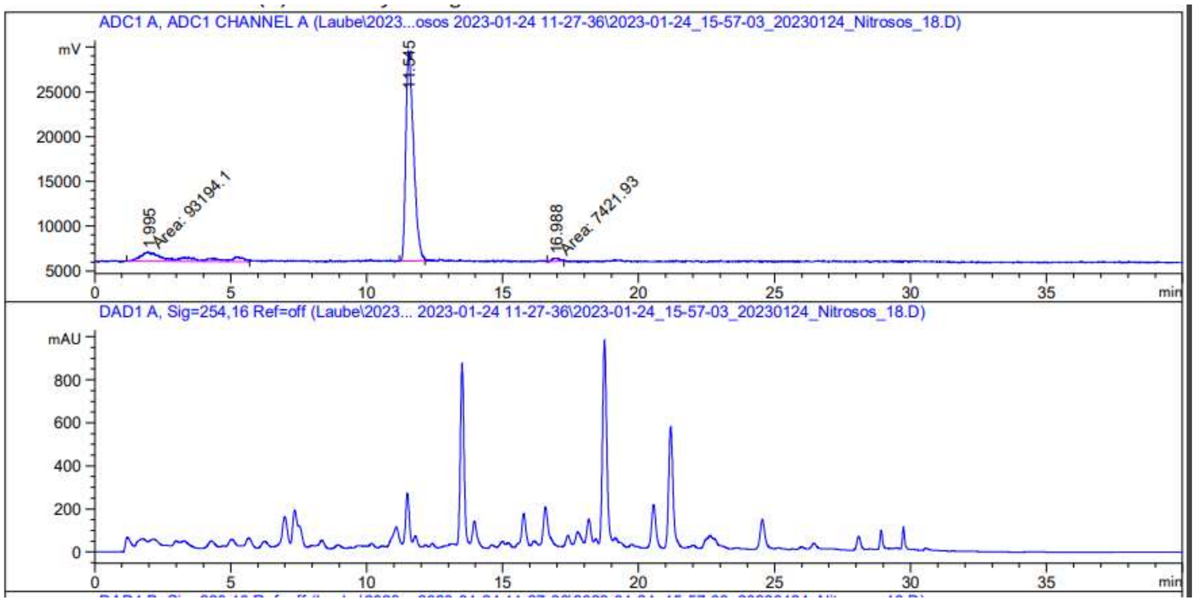

Figure S69. Exemplary analytical radio-HPLC chromatogram (System 2, gradient 1; upper panel: signal of gamma-detector; lower panel: UV-signal) of the crude reaction mixture obtained for ^{18}F-labeling of compound **15a** and reaction with normal base at 110°C in DMSO.

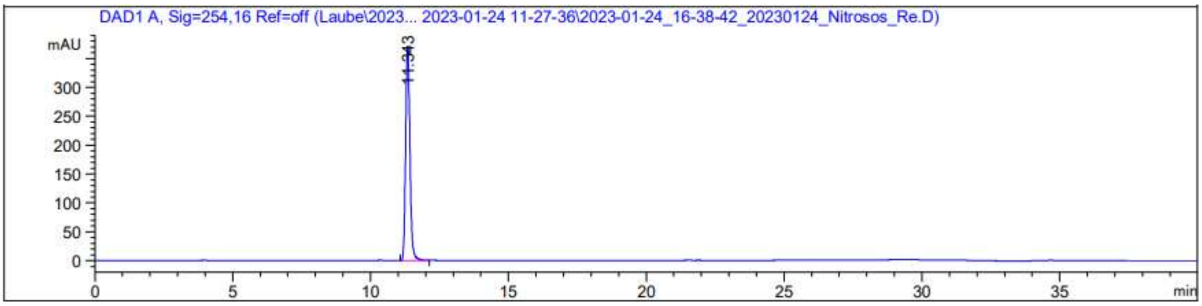

Figure S70. Analytical HPLC chromatogram (System 2, gradient 1) of the authentic non-radioactive reference **15b** (t_R 11.343 min).

A)				TLC		Set	P	Q		
			1	2	3	4	5	6	7	8
	SR292F1/22		**MeCN**		**DMF**			**DMSO**		
			90	110	90	110	130	90	110	130
	**Normal**	Product	14,8	2,6	74,9	53,6	5	73,7	77,8	23,8
		Side Products								
	**1/4 Base**	Product	9,8	22,9	4	4,9	20,4	36,4	25,1	29,9
		Side Products								

B)				HPLC						
			1	2	3	4	5	6	7	8
	SR292F1/22		**MeCN**		**DMF**			**DMSO**		
			90	110	90	110	130	90	110	130
	**Normal**	Product	n.d.	n.d.	n.d.	n.d.	n.d.	n.d.	82,8	n.d.
		Side Products								
	**1/4 Base**	Product	n.d.	n.d.	n.d.	n.d.	n.d.	37,6	n.d.	n.d.
		Side Products								

Figure S71. Detailed results of optimization experiments for ^{18}F-labeling of compound **15a** obtained by radio-TLC (A) and radio-HPLC (B) analysis. Side Prod. Side Product(s); n.d. not determined.

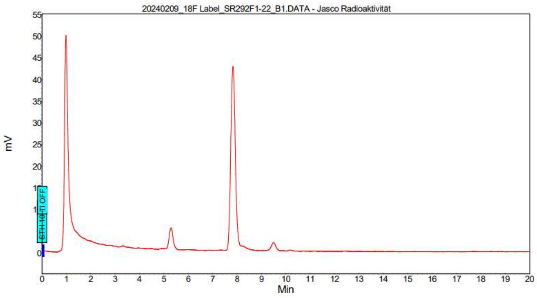

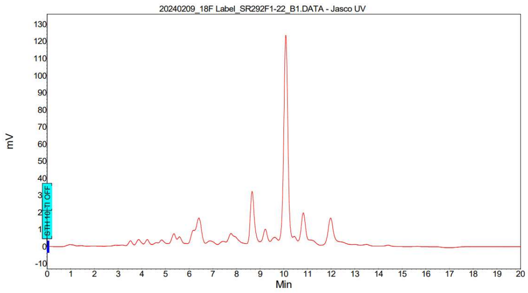

Figure S72. Copy of semi-preparative HPLC chromatograms (System 3; left: signal of gamma-detector; right: UV-signal) obtained for purification of compound **[^{18}F]15b** after labeling of **15a** with [^{18}F]fluoride under optimized conditions. Product was collected between 7.6 and 8.4 min.

	#1	#2	#3	#4	#5	#6	Mean ± SD (n)
RCY [%]	17	45	49	57			42.0 ± 15.1 (n=4)

Table S10. Detailed results of RCY and Mean ± SD (n) for the radiosynthesis and isolation of **[^{18}F]15b**.

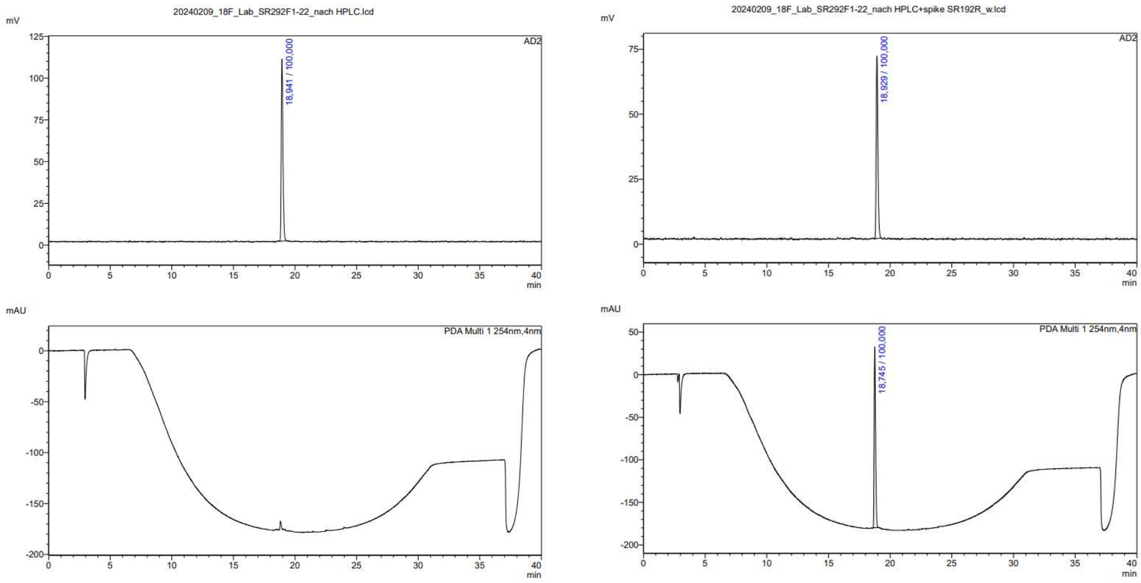

Figure S73. Copy of analytical HPLC chromatograms (System 1; upper panel: signal of gamma-detector; lower panel: UV-signal) obtained for compound **[^{18}F]15b** after semi-preparative purification without (left) and with (right) addition of the authentic non-radioactive reference. In the HPLC setup, the UV detector is in row before the γ-detector with Δt_R of 0.17-0.18 min between both detectors.

Radiolabeling of compound 16a

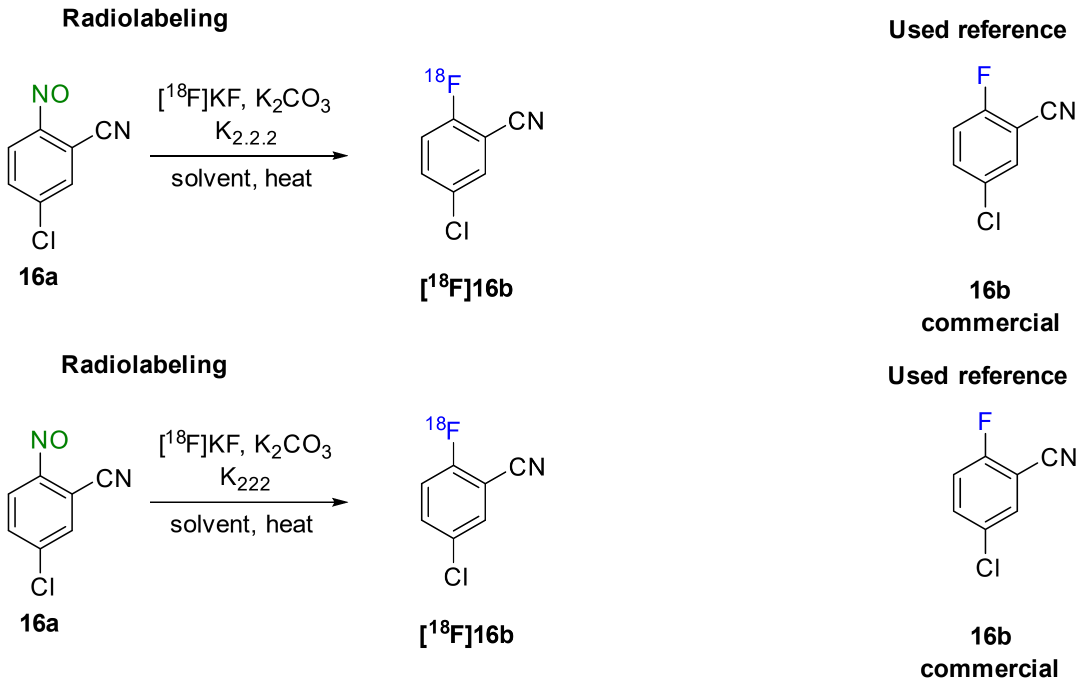

Figure S74. Overview for radiolabeling and used references

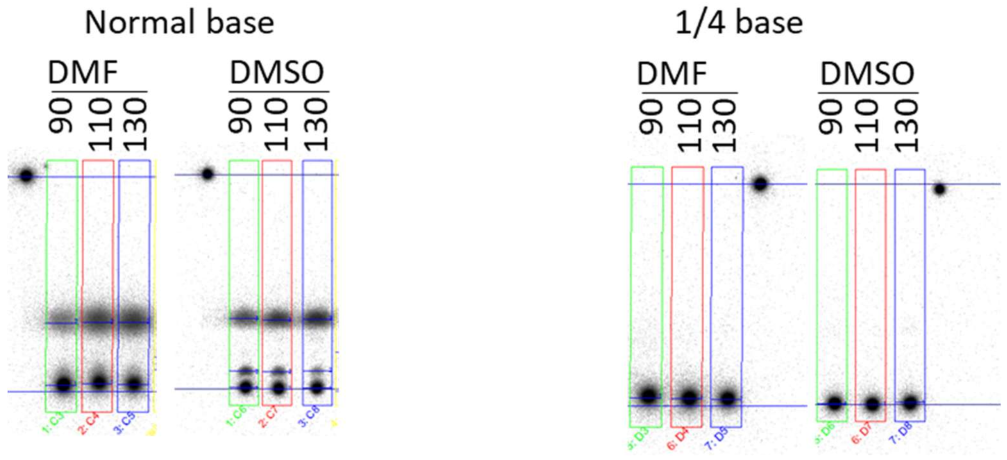

Figure S75. Copy of radio-TLC obtained for ^{18}F-labeling of compound **16a** using normal base (left) and ¼ base (right).

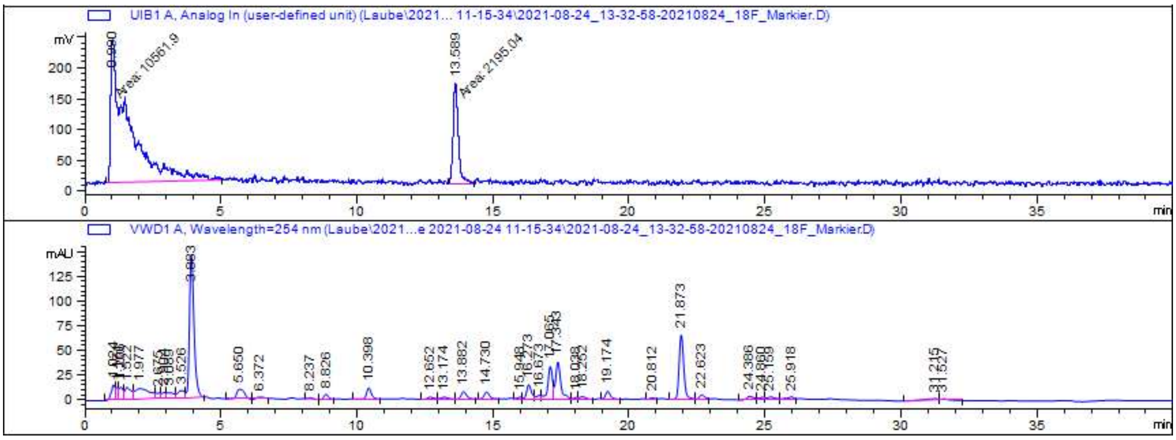

Figure S76. Exemplary analytical radio-HPLC chromatogram (System 2, gradient 1; upper panel: signal of gamma-detector; lower panel: UV-signal) of the crude reaction mixture obtained for ^{18}F-labeling of compound **16a** and reaction with normal base at 130°C in DMF.

A)				**TLC**		Set		C	D	
			1	2	3	4	5	6	7	8
	**SR188F3/20**		**MeCN**		**DMF**			**DMSO**		
			90	110	90	110	130	90	110	130
	**Normal**	Product	n.d.	n.d.	11	36	43	20	32	45
		Side Products			0	0	0	0	0	0
	**1/4 Base**	Product	n.d.	n.d.	0	0	0	0	0	0
		Side Products			0	0	0	0	0	0

B)				**HPLC**						
			1	2	3	4	5	6	7	8
	**SR188F3/20**		**MeCN**		**DMF**			**DMSO**		
			90	110	90	110	130	90	110	130
	**Normal**	Product	n.d.	n.d.	n.d.	n.d.	n.d.	n.d.	n.d.	n.d.
		Side Products								
	**1/4 Base**	Product	n.d.	n.d.	n.d.	n.d.	n.d.	n.d.	n.d.	n.d.
		Side Products								

Figure S77. Detailed results of optimization experiments for ^{18}F-labeling of compound **6a** obtained by radio-TLC (A) and radio-HPLC (B) analysis. not determined.

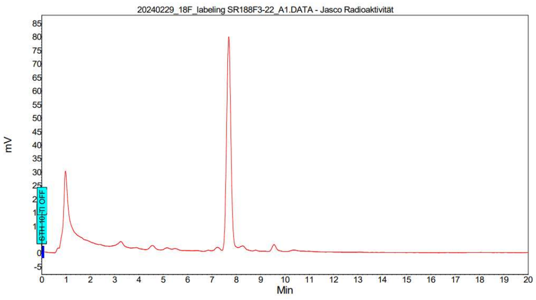

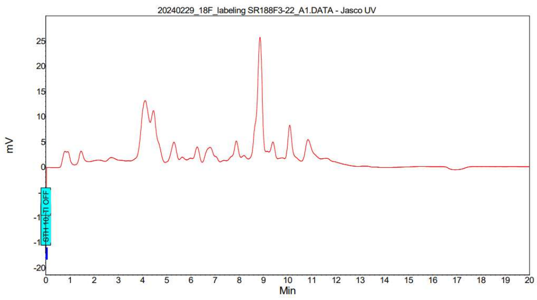

Figure S78. Copy of semi-preparative HPLC chromatograms (System 3; left: signal of gamma-detector; right: UV-signal) obtained for purification of compound **[^{18}F]16b** after labeling of **16a** with [^{18}F]fluoride under optimized conditions. Product was collected between 7.5 and 8.4 min.

	#1	#2	#3	#4	#5	#6	Mean ± SD (n)
RCY [%]	40	43	24	16	33		31.2 ± 10.0 (n=5)

Table S11. Detailed results of RCY and Mean ± SD (n) for the radiosynthesis and isolation of **[^{18}F]16b**.

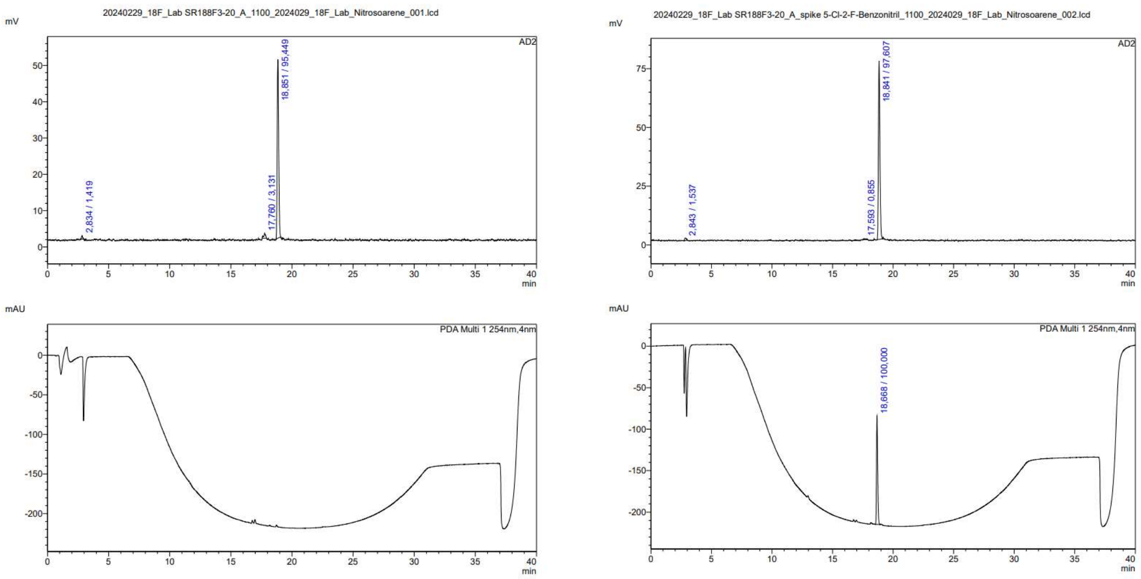

Figure S79. Copy of analytical HPLC chromatograms (System 1; upper panel: signal of gamma-detector; lower panel: UV-signal) obtained for compound **[^{18}F]16b** after semi-preparative purification without (left) and with (right) addition of the authentic non-radioactive reference. In the HPLC setup, the UV detector is in row before the γ-detector with Δt_R of 0.17-0.18 min between both detectors.

Radiolabeling of compound 17a

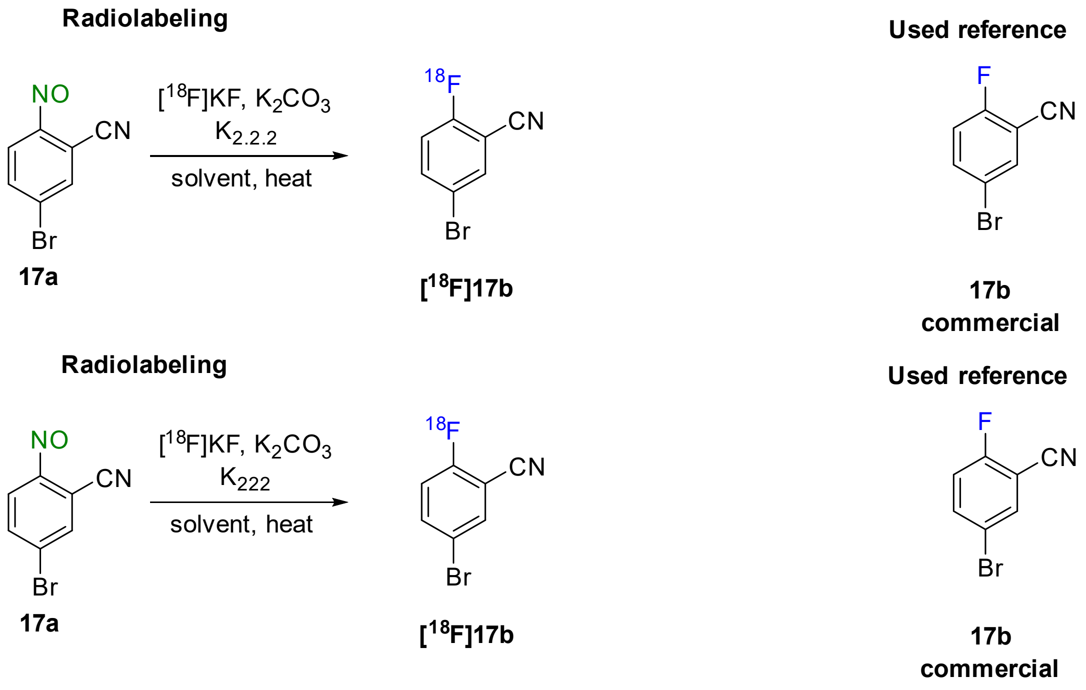

Figure S*80*. Overview for radiolabeling and used references

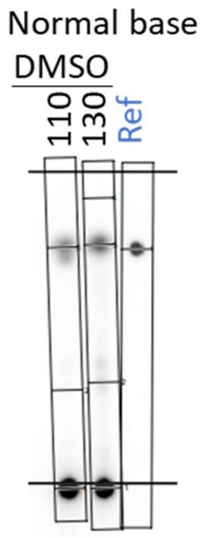

Figure S81. Copy of radio-TLC obtained for ^{18}F-labeling of compound **17a** normal base. TLC conditions silica gel; n-hexan/EtOAC 50/50.

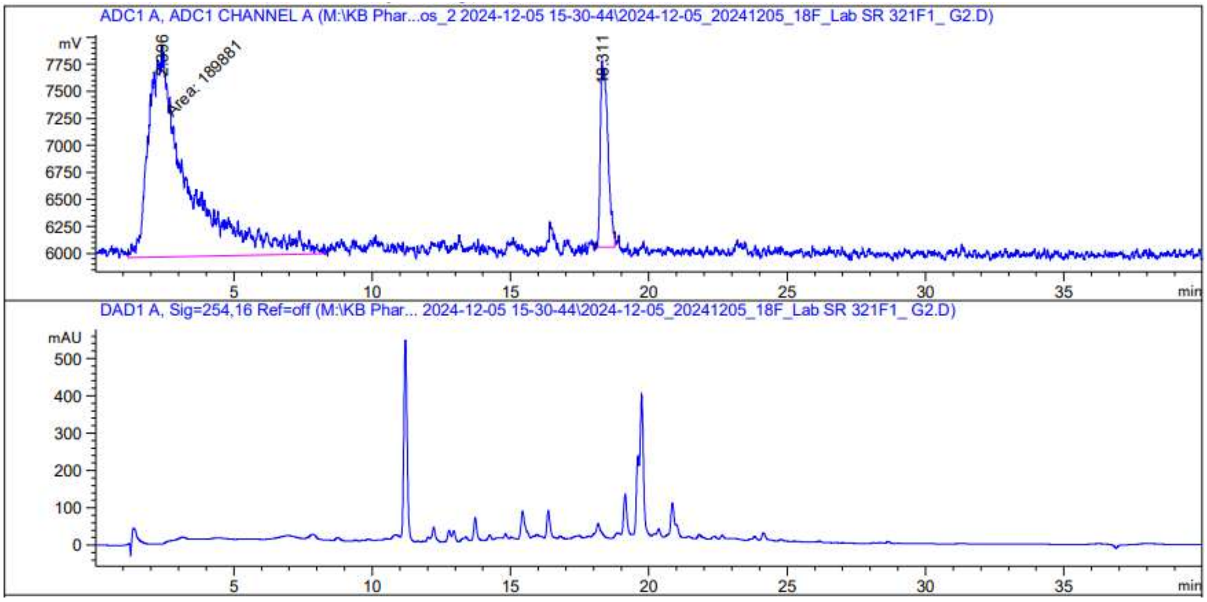

Figure S82. Exemplary analytical radio-HPLC chromatogram (System 2, gradient 4; upper panel: signal of gamma-detector; lower panel: UV-signal) of the crude reaction mixture obtained for ^{18}F-labeling of compound **17a** and reaction with normal base at 130°C in DMSO.

A)				TLC		Set				
			1	2	3	4	5	6	7	8
	SR321F1/24		MeCN		DMF			DMSO		
			90	110	90	110	130	90	110	130
	Normal	Product	n.d.	n.d.	n.d.	n.d.	n.d.	n.d.	14	18
		Side Products							0	0
	1/4 Base	Product	n.d.	n.d.	n.d.	n.d.	n.d.	n.d.	n.d.	n.d.
		Side Products								

B)				HPLC						
			1	2	3	4	5	6	7	8
	SR321F1/24		MeCN			DMF			DMSO	
			90	110	90	110	130	90	110	130
	Normal	Product	n.d.	n.d.	n.d.	n.d.	n.d.	n.d.	n.d.	14
		Side Products								0
	1/4 Base	Product	n.d.	n.d.	n.d.	n.d.	n.d.	n.d.	n.d.	n.d.
		Side Products								

Figure S83. Detailed results of optimization experiments for ^{18}F-labeling of compound **17a** obtained by radio-TLC (A) and radio-HPLC (B) analysis. n.d. not determined.

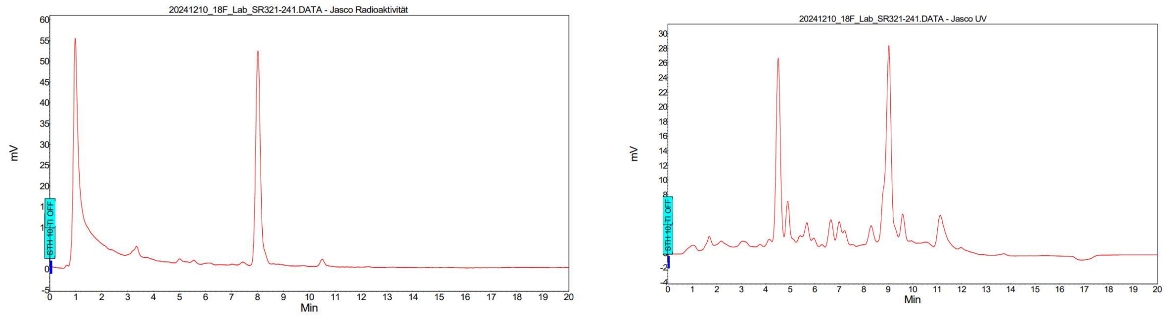

Figure S84. Copy of semi-preparative HPLC chromatograms (System 3;left: signal of gamma-detector; right: UV-signal) obtained for purification of compound **[^{18}F]17b** after labeling of **17a** with [^{18}F]fluoride under optimized conditions. Product was collected between 7.4 and 8.5 min.

	#1	#2	#3	#4	#5	#6	Mean ± SD (n)
RCY [%]	21	22	21				21.3 ± 0.5 (n=3)

Table S12. Detailed results of RCY and Mean ± SD (n) for the radiosynthesis and isolation of **[^{18}F]17b**.

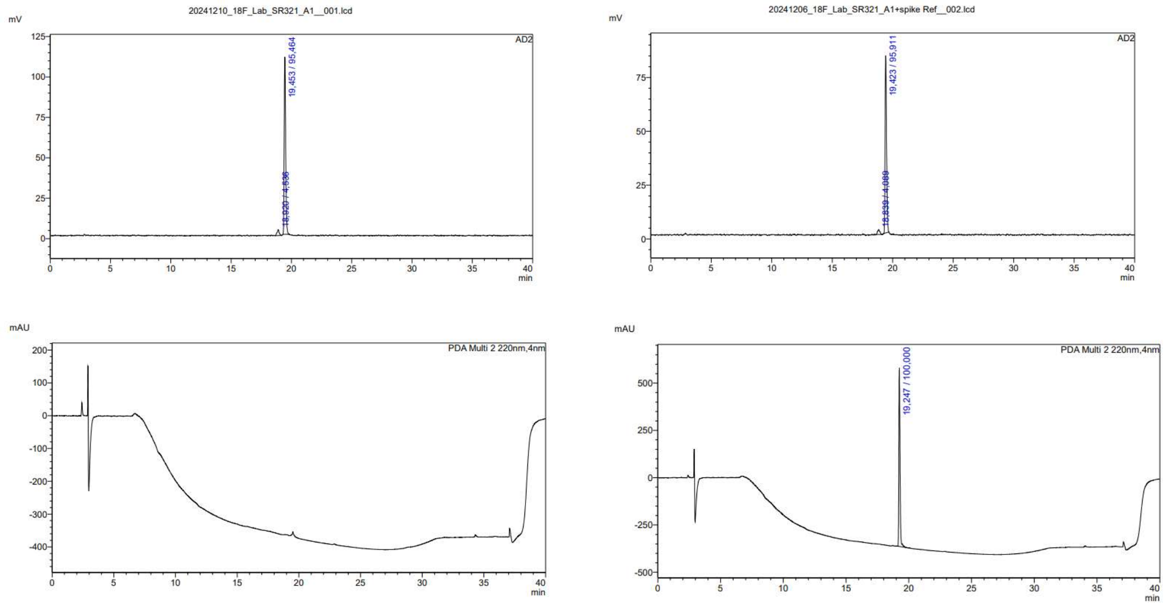

Figure S85. Copy of analytical HPLC chromatograms (System 1;upper panel: signal of gamma-detector; lower panel: UV-signal at 220 nm) obtained for compound **[^{18}F]17b** after semi-preparative purification without (left) and with (right) addition of the authentic non-radioactive reference. In the HPLC setup, the UV detector is in row before the γ-detector with Δt_R of 0.17-0.18 min between both detectors.

Radiolabeling of compound 18a

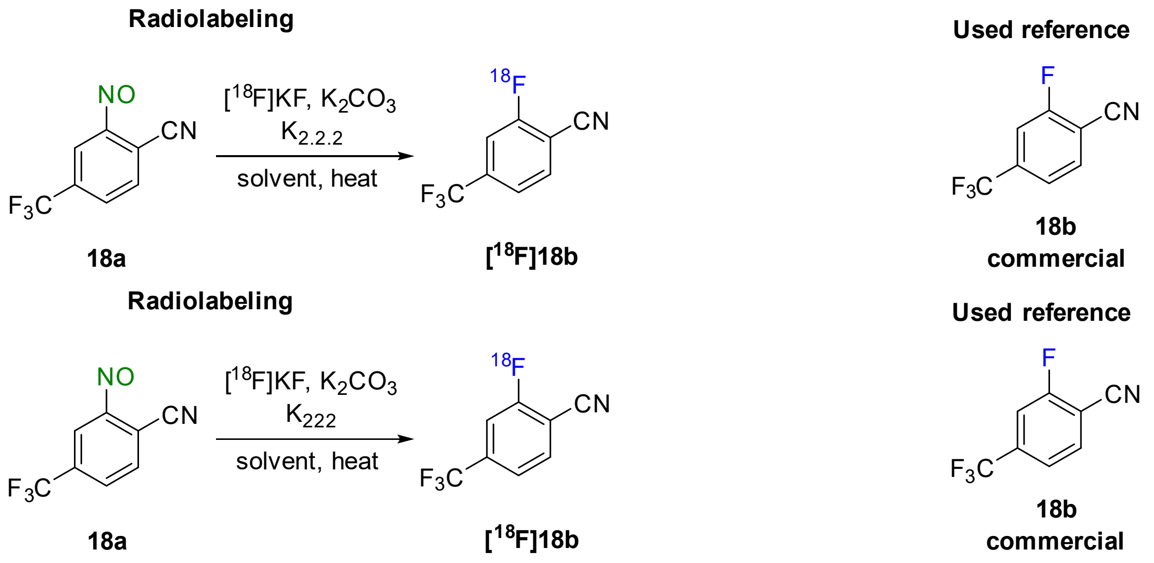

Figure S*86*. Overview for radiolabeling and used references

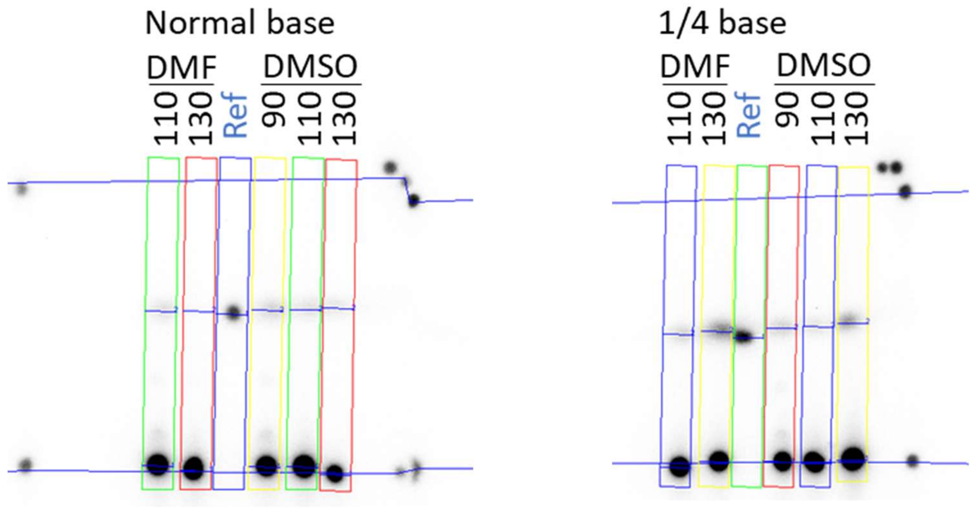

Figure S87. Copy of radio-TLC obtained for ^{18}F-labeling of compound **18a** using normal base (left) and ¼ base (right). Note: A selected set of experiments as indicated in the table below was performed for compound **18a**. The order of spotted samples is consistent with the general scheme starting however from experiment 4 -DMF, 110°C.

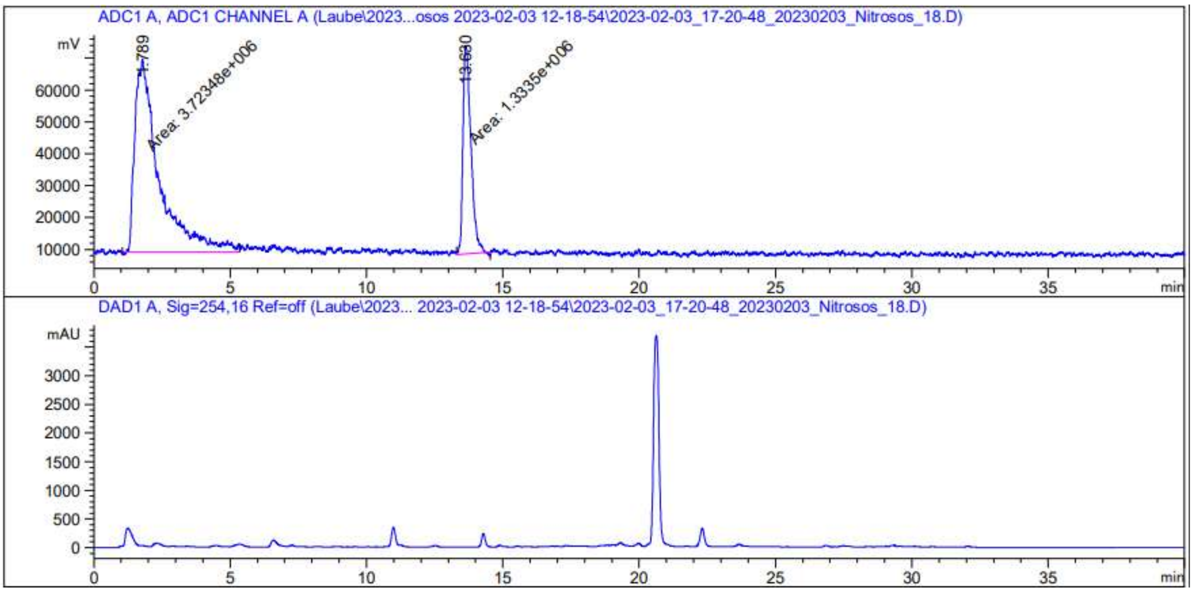

Figure S88. Exemplary analytical radio-HPLC chromatogram (System 2, gradient 1; upper panel: signal of gamma-detector; lower panel: UV-signal) of the crude reaction mixture obtained for ^{18}F-labeling of compound **18a** and reaction with normal base at 130°C in DMF.

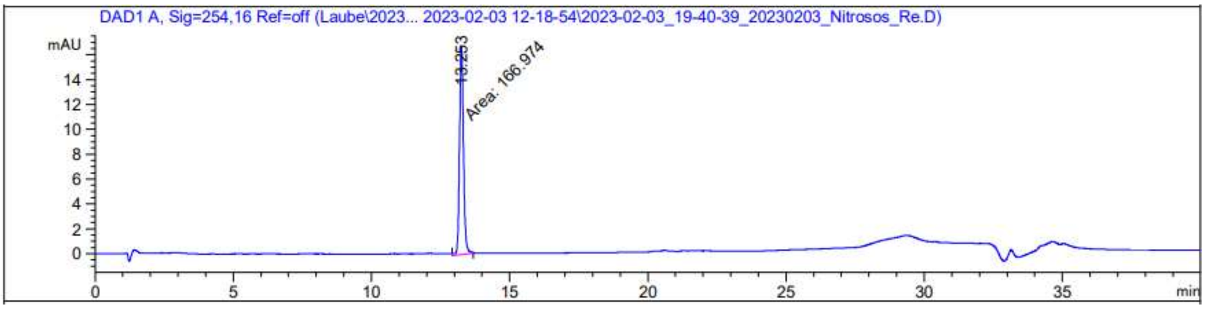

Figure S89. Analytical HPLC chromatogram (System 2, gradient 1) of the authentic non-radioactive reference **18b** (t_R 13.253 min).

A)				TLC		Set	T	U		
			1	2	3	4	5	6	7	8
	**SR199F1/22**		**MeCN**		**DMF**			**DMSO**		
			90	110	90	110	130	90	110	130
	**Normal**	Product	n.d.	n.d.	n.d.	3,4	1	5,3	3,4	7,6
		Side Products								
	**1/4 Base**	Product	n.d.	n.d.	n.d.	4,6	13,8	5,7	3,3	5,4
		Side Products								

B)				HPLC			T	U		
			1	2	3	4	5	6	7	8
	**SR199F1/22**		**MeCN**		**DMF**			**DMSO**		
			90	110	90	110	130	90	110	130
	**Normal**	Product	n.d.	n.d.	n.d.	7,5	0	11,9	6,7	7
		Side Products								
	**1/4 Base**	Product	n.d.	n.d.	n.d.	10,4	27,1	10,4	3,3	5,5
		Side Products								

Figure S90. Detailed results of optimization experiments for ^{18}F-labeling of compound **18a** obtained by radio-TLC (A) and radio-HPLC (B) analysis. n.d. not determined.

Radiolabeling of compound 19a

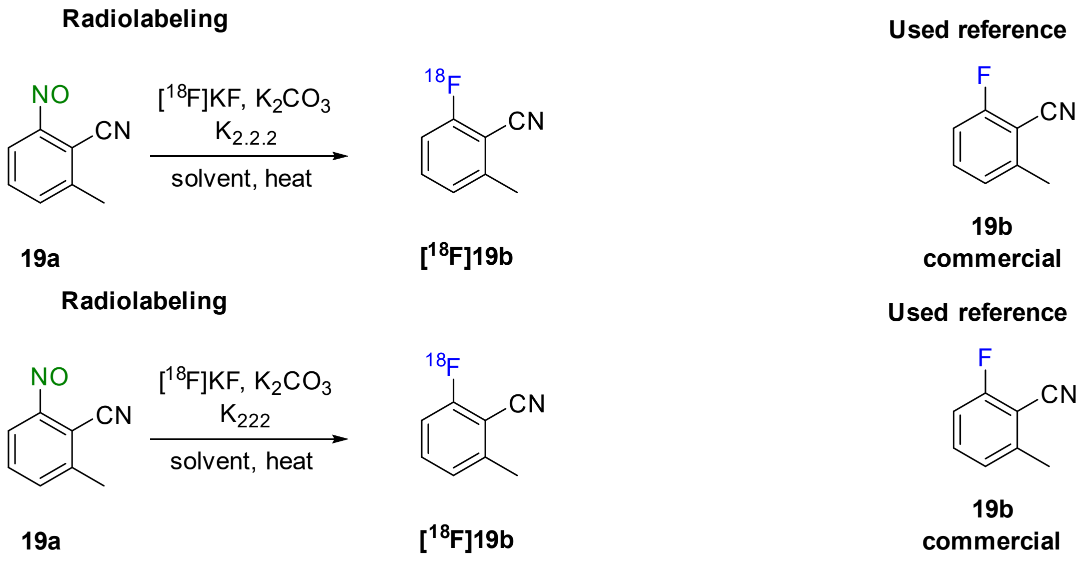

Figure S*91*. Overview for radiolabeling and used references

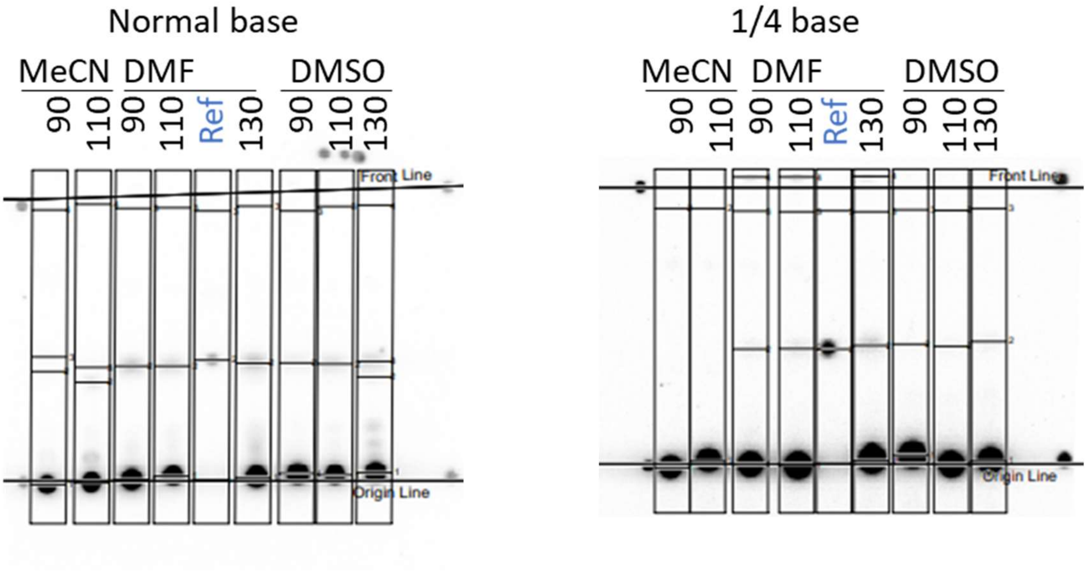

Figure S92. Copy of radio-TLC obtained for ^{18}F-labeling of compound **19a** using normal base (left) and ¼ base (right).

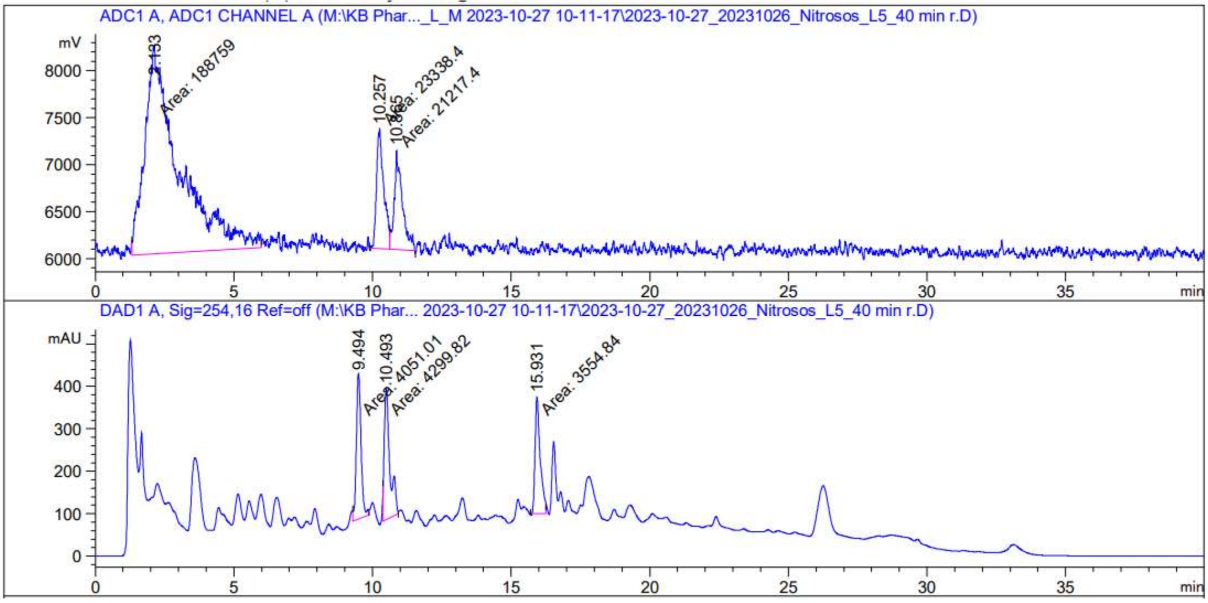

Figure S93. Exemplary analytical radio-HPLC chromatogram (System 2, gradient 1; upper panel: signal of gamma-detector; lower panel: UV-signal) of the crude reaction mixture obtained for ^{18}F-labeling of compound **19a** and reaction with normal base at 130°C in DMF.

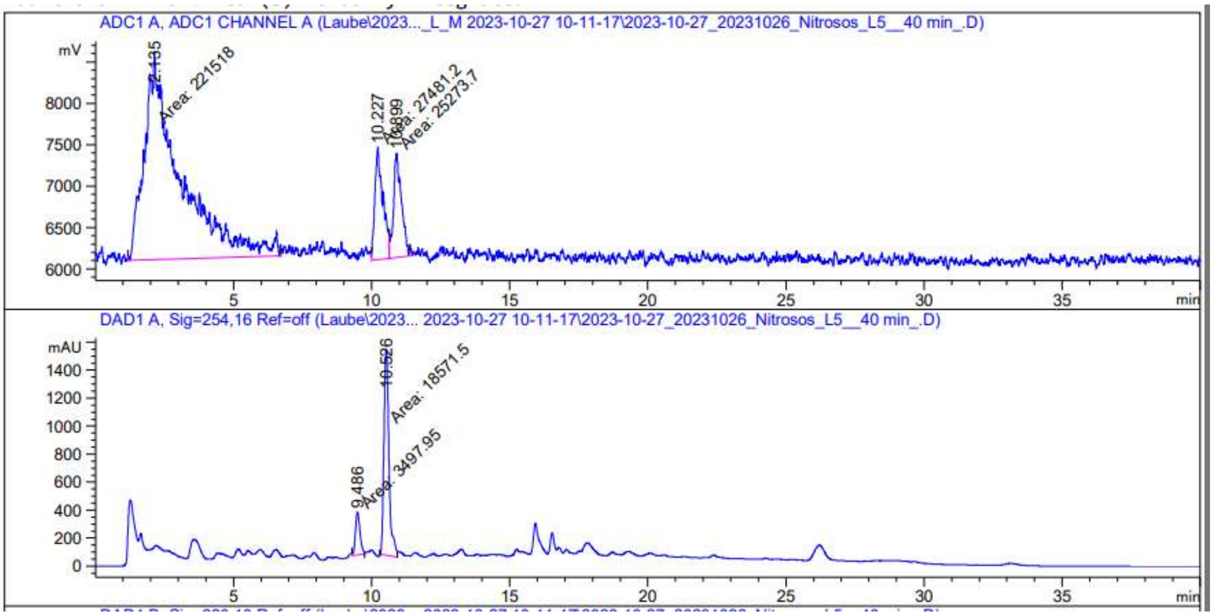

Figure S94. Exemplary analytical radio-HPLC chromatogram (System 2, gradient 1; upper panel: signal of gamma-detector; lower panel: UV-signal) of the crude reaction mixture spiked with authentic non-radioactive reference (t_R 10.526 min) obtained for ^{18}F-labeling of compound **19a** and reaction with normal base at 130°C in DMF.

A)				TLC		Set	L	M		
			1	2	3	4	5	6	7	8
	**SR 43/23**		**MeCN**		**DMF**			**DMSO**		
			90	110	90	110	130	90	110	130
	**Normal**	Product	0,6	0,9	3,9	4,4	5,6	2	4,2	4,5
		Side Products	0,2	2,7	0	0	0	0	0	0,4
	**1/4 Base**	Product	0	0	0,6	0,9	1,8	0,2	0,5	0,7
		Side Products	0	0	0,3	0,4	0,5	0	0	0

B)				HPLC			L	M		
			1	2	3	4	5	6	7	8
	**SR 43/23**		**MeCN**		**DMF**			**DMSO**		
			90	110	90	110	130	90	110	130
	**Normal**	Product	0	0,7	8,9	7,2	10	3,1	7,3	8
		Side Products	25	39	5,5	4,3	10,7	4,1	7,3	12,6
	**1/4 Base**	Product	0	0	0	0	0	0	0	n.d.
		Side Products	0	0	0	0	0	0	0	n.d.

Figure S95. Detailed results of optimization experiments for ^{18}F-labeling of compound **19a** obtained by radio-TLC (A) and radio-HPLC (B) analysis. n.d. not determined.

Radiolabeling of compound 20a

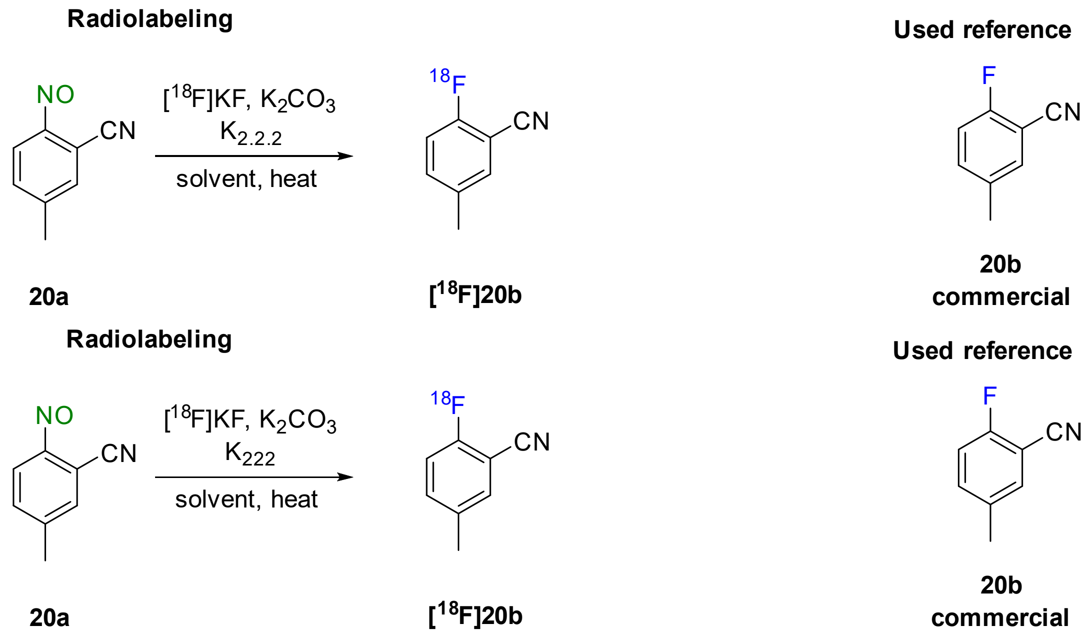

Figure S*96*. Overview for radiolabeling and used references

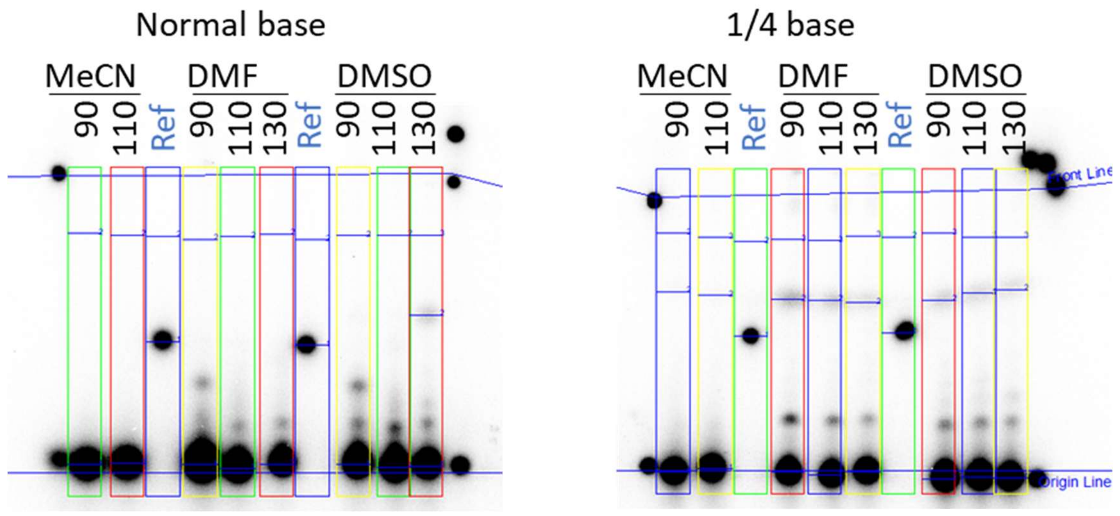

Figure S97. Copy of radio-TLC obtained for ^{18}F-labeling of compound **20a** using normal base (left) and ¼ base (right).

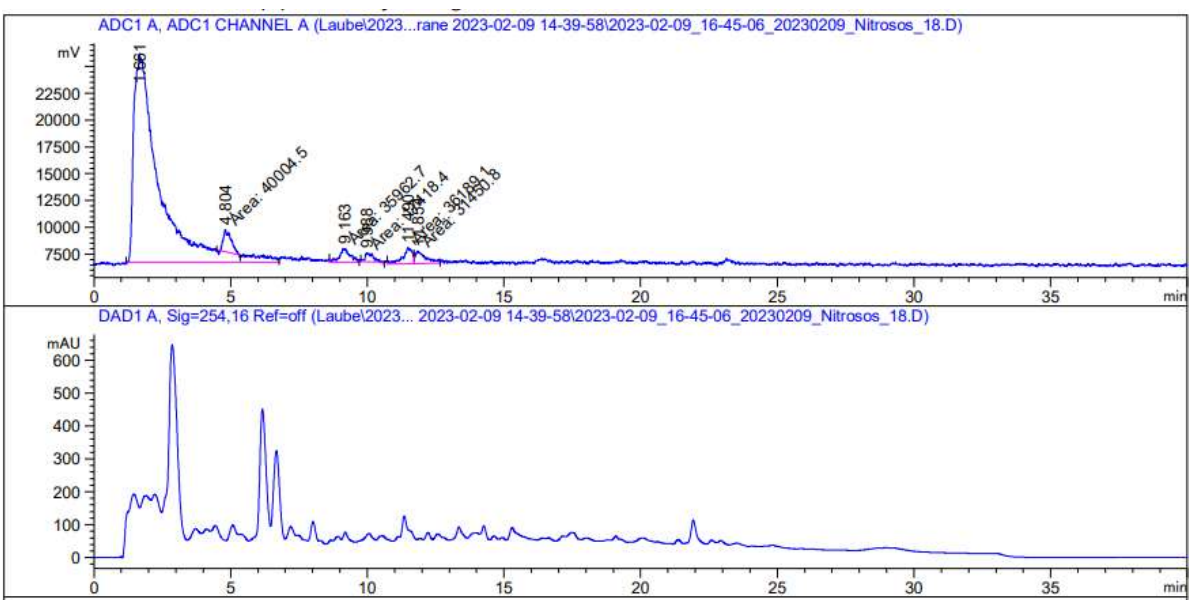

Figure S98. Exemplary analytical radio-HPLC chromatogram (System 2, gradient 1; upper panel: signal of gamma-detector; lower panel: UV-signal) of the crude reaction mixture obtained for ^{18}F-labeling of compound **20a** and reaction with normal base at 130°C in DMSO.

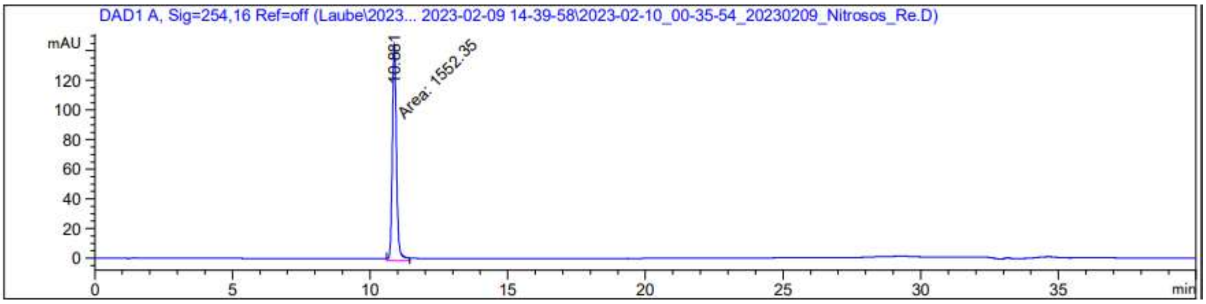

Figure S99. Analytical HPLC chromatogram (System 2, gradient 1) of the authentic non-radioactive reference **20b** (tR 10.881 min).

A)				TLC		Set	R	S		
			1	2	3	4	5	6	7	8
	**SR188F1/22**		**MeCN**		**DMF**			**DMSO**		
			90	110	90	110	130	90	110	130
	**Normal**	Product	0	0	0	0	0	0	0	0
		Side Products								1,9
	**1/4 Base**	Product	0	0	0	0	0	0	0	0
		Side Products	0,1	0,3	3,7	2,7	2,2	2	2	1,7

B)				HPLC			R	S		
			1	2	3	4	5	6	7	8
	**SR188F1/22**		**MeCN**		**DMF**			**DMSO**		
			90	110	90	110	130	90	110	130
	**Normal**	Product	n.d.	n.d.	n.d.	n.d.	n.d.	n.d.	n.d.	0
		Side Products								12
	**1/4 Base**	Product	n.d.	n.d.	0	n.d.	n.d.	0	n.d.	n.d.
		Side Products			6,3					

Figure S100. Detailed results of optimization experiments for ^{18}F-labeling of compound **20a** obtained by radio-TLC (A) and radio-HPLC (B) analysis. n.d. not determined.

Radiolabeling of compound 21a

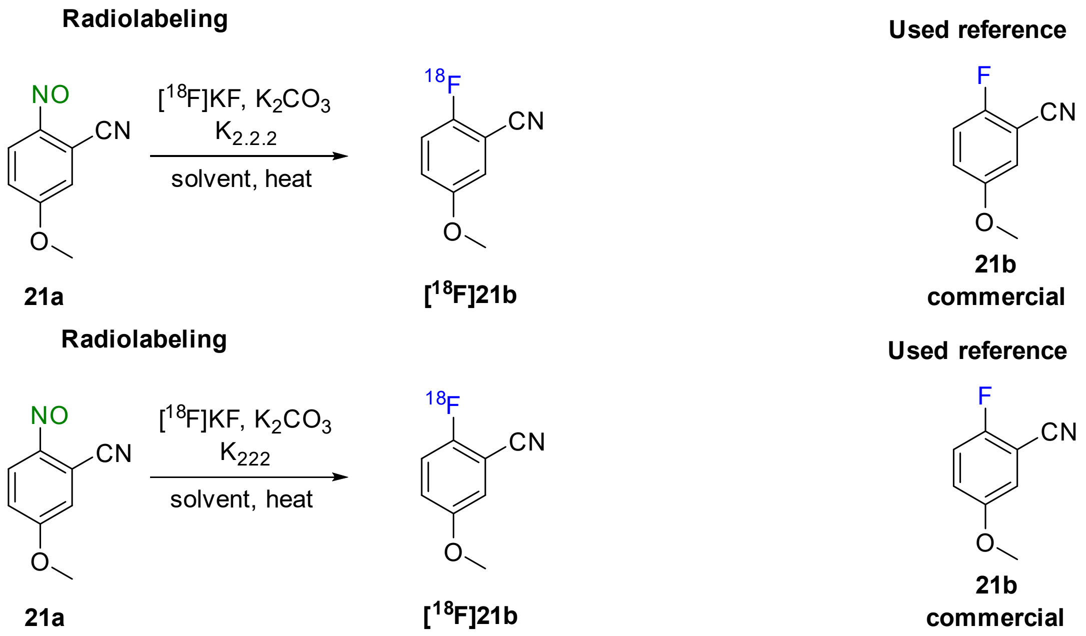

Figure S*101*. Overview for radiolabeling and used references

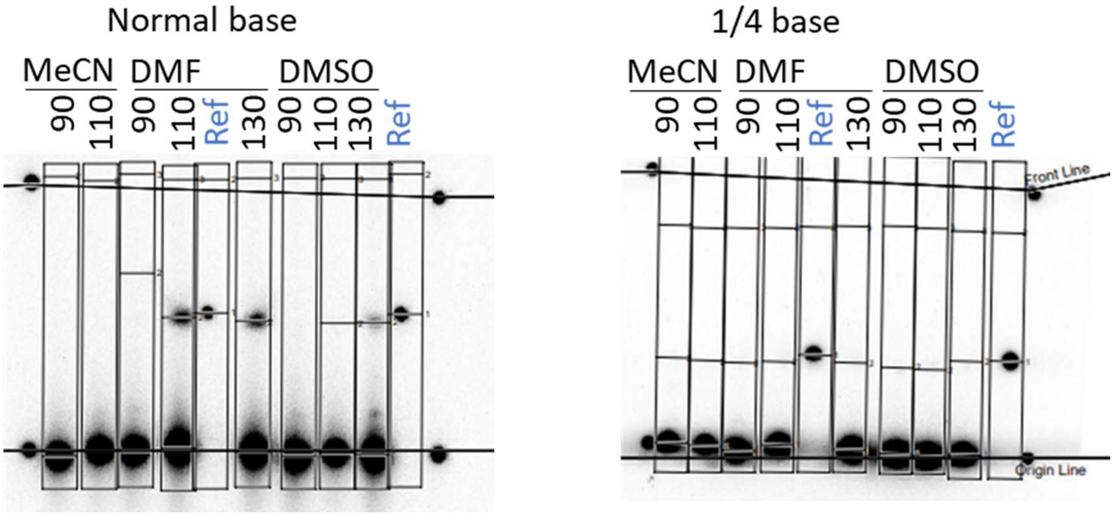

Figure S102. Copy of radio-TLC obtained for ^{18}F-labeling of compound **21a** using normal base (left) and ¼ base (right). The minor difference in R_f between reference compound and radiolabeled products was attributed to differences in sample composition and hence radiolabeled compound identified as product.

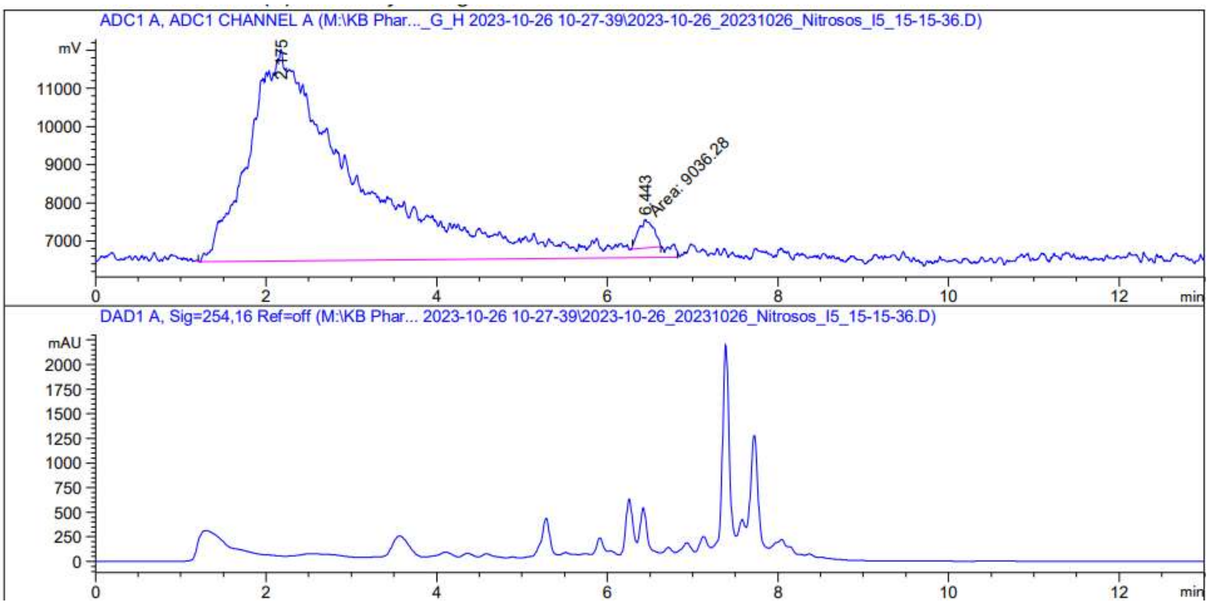

Figure S103. Exemplary analytical radio-HPLC chromatogram (System 2, gradient 2; upper panel: signal of gamma-detector; lower panel: UV) of the crude reaction mixture obtained for ^{18}F-labeling of compound **21a** and reaction with normal base at 130°C in DMF.

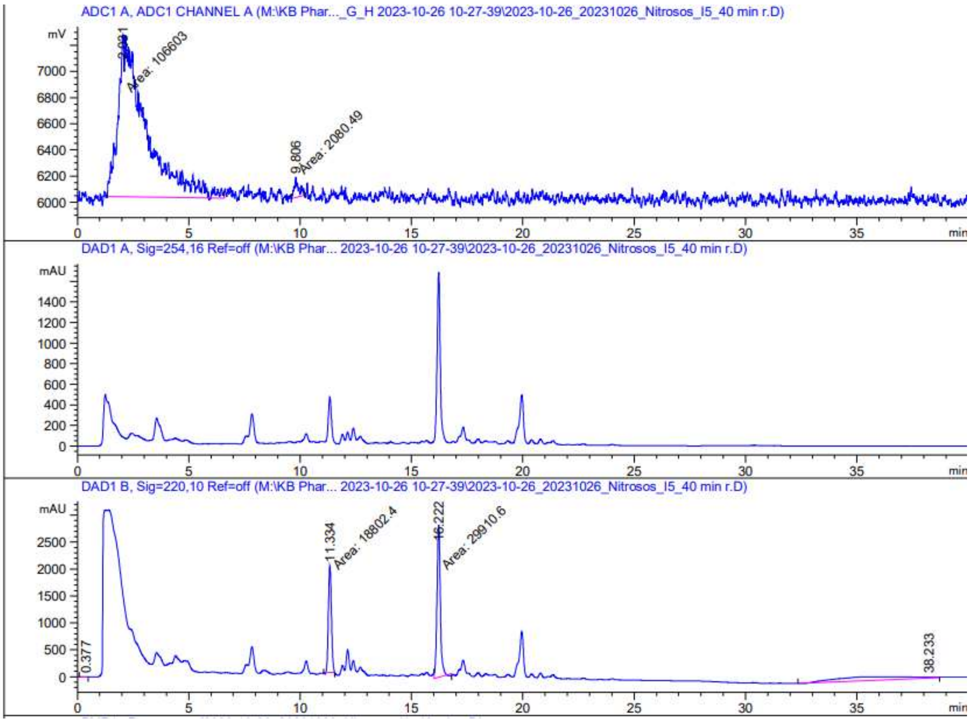

Figure S104. Exemplary analytical radio-HPLC chromatogram (System 2, gradient 1; upper panel: signal of gamma-detector; middle and lower panel: UV-signal at 254 and 220 nm, respectively) of the crude reaction mixture obtained for ^{18}F-labeling of compound **21a** and reaction with normal base at 130°C in DMF.

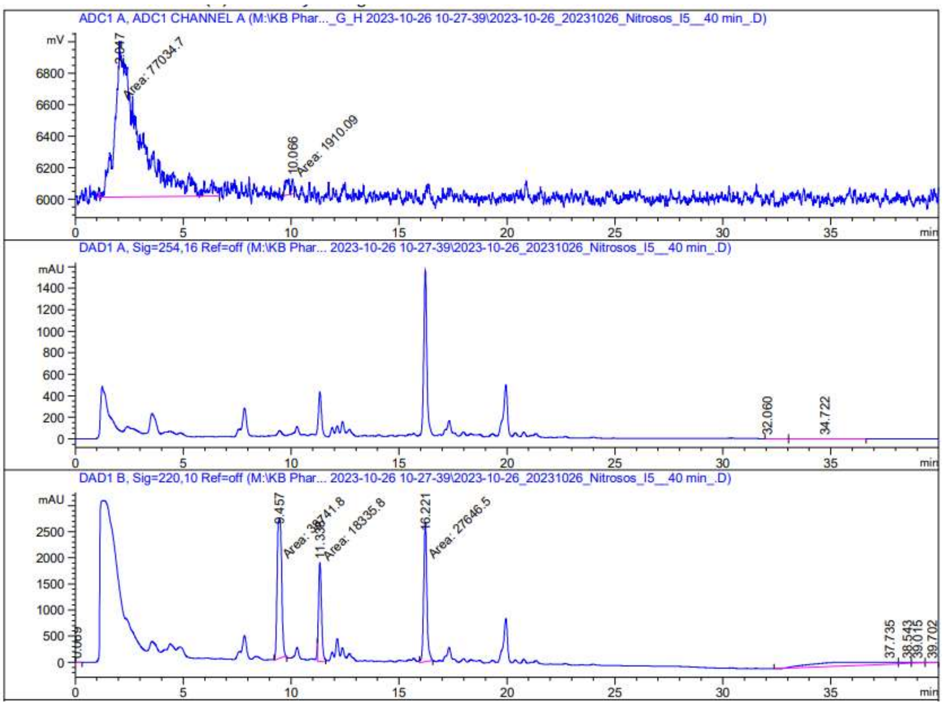

Figure S105. Exemplary analytical radio-HPLC chromatogram (System 2, gradient 1; upper panel: signal of gamma-detector; middle and lower panel: UV-signal at 254 and 220 nm, respectively) of the crude reaction mixture mixture spiked with authentic non-radioactive reference (t_R 9.457 min) obtained for ^{18}F-labeling of compound **21a** and reaction with normal base at 130°C in DMF.

A)				**TLC**		Set	I	K		
			1	2	3	4	5	6	7	8
	**SR 45/23**		**MeCN**		**DMF**			**DMSO**		
			90	110	90	110	130	90	110	130
	**Normal**	Product	0	0	0	2,7	2,7	0	0,2	1
		Side Products	0	0	0	0	0	0	0	0
	**1/4 Base**	Product	0,2	0,2	0,3	0,4	0,4	0,2	0,3	0,4
		Side Products	0	0	0	0	0	0	0	0

B)				**HPLC**			I	K		
			1	2	3	4	5	6	7	8
	**SR 45/23**		**MeCN**		**DMF**			**DMSO**		
			90	110	90	110	130	90	110	130
	**Normal**	Product	0	0	0	0	2,3	0	0	0,5
		Side Products	0,6	0	0	0	0	0	1	1,2
	**1/4 Base**	Product	0	0	0	0	0	0	0	0
		Side Products	0	0	0	0	0	0	0	0

Figure S106. Detailed results of optimization experiments for ^{18}F-labeling of compound **21a** obtained by radio-TLC (A) and radio-HPLC (B) analysis. n.d. not determined.

Radiolabeling of compound 22a

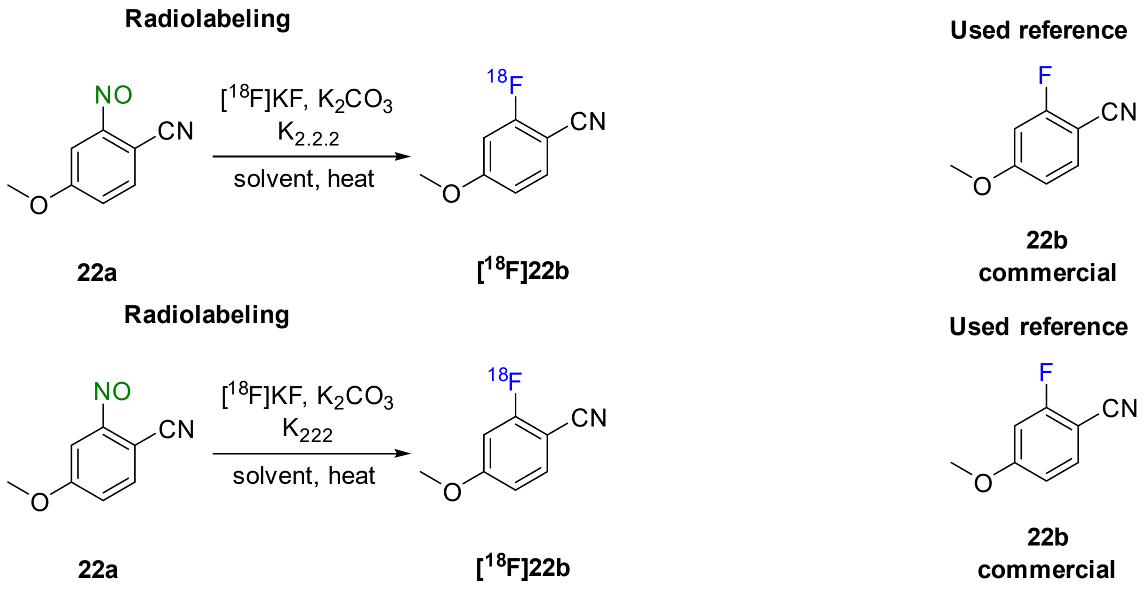

Figure S*107*. Overview for radiolabeling and used references

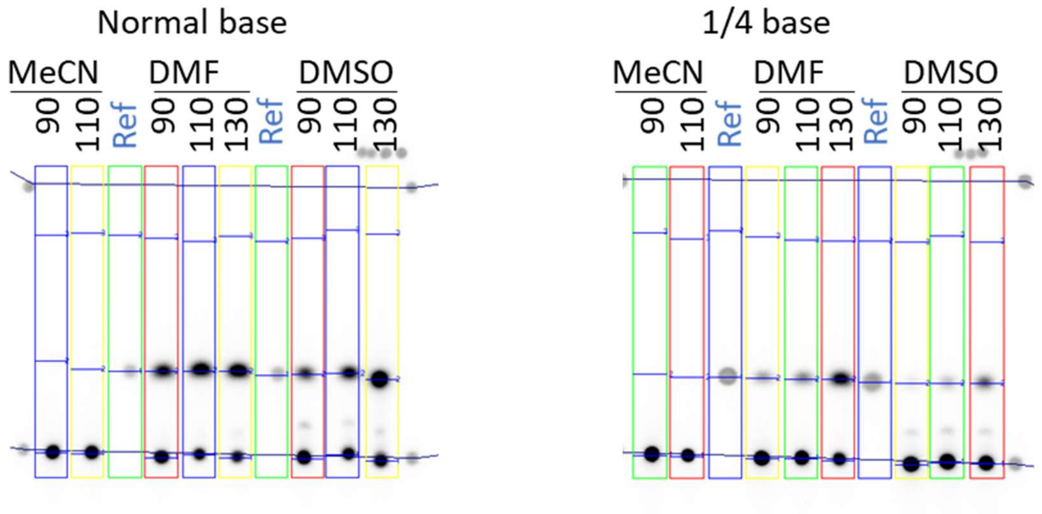

Figure S108. Copy of radio-TLC obtained for ^{18}F-labeling of compound **22a** using normal base (left) and ¼ base (right).

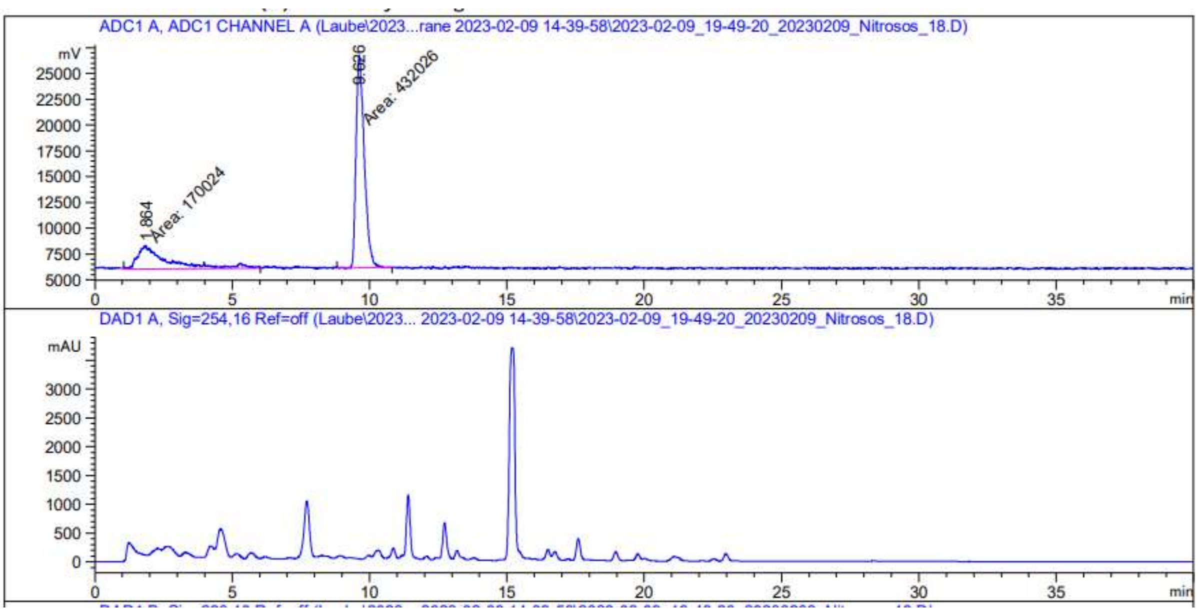

Figure S109. Exemplary analytical radio-HPLC chromatogram (System 2, gradient 1; upper panel: signal of gamma-detector; lower panel: UV-signal) of the crude reaction mixture obtained for ^{18}F-labeling of compound **22a** and reaction with normal base at 130°C in DMF.

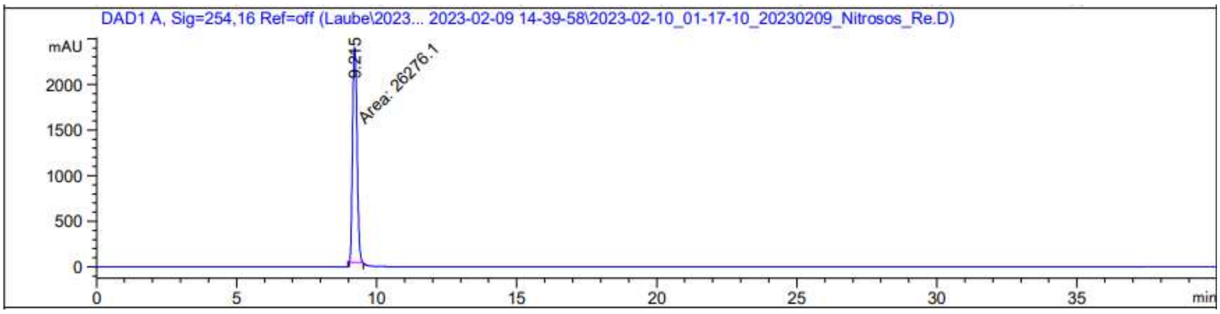

Figure S110. Analytical HPLC chromatogram (System 2, gradient 1) of the authentic non-radioactive reference **22b** (t_R 9.215 min).

A)				TLC		Set	V	W		
			1	2	3	4	5	6	7	8
	**SR 206F1**		**MeCN**		**DMF**			**DMSO**		
			90	110	90	110	130	90	110	130
	**Normal**	Product	3	2,8	50,8	66,7	70,1	38,2	51,5	63
		Side Products								
	**1/4 Base**	Product	1,6	2,2	19,5	26,7	56,3	6,4	12,8	28,7
		Side Products								

B)				HPLC			V	W		
			1	2	3	4	5	6	7	8
	**SR 206F1**		**MeCN**		**DMF**			**DMSO**		
			90	110	90	110	130	90	110	130
	**Normal**	Product	n.d.	n.d.	n.d.	n.d.	83,5	n.d.	n.d.	79
		Side Products								
	**1/4 Base**	Product	n.d.	n.d.	n.d.	n.d.	62,8	n.d.	n.d.	28,4
		Side Products								

Figure S111. Detailed results of optimization experiments for ^{18}F-labeling of compound **22a** obtained by radio-TLC (A) and radio-HPLC (B) analysis. n.d. not determined.

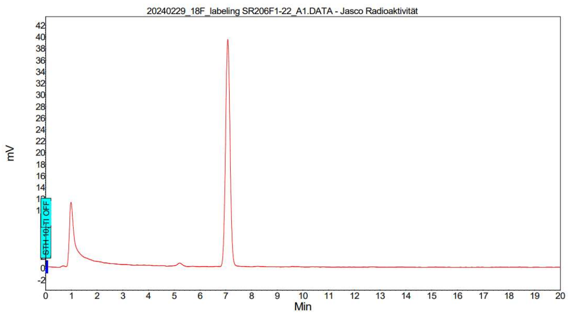

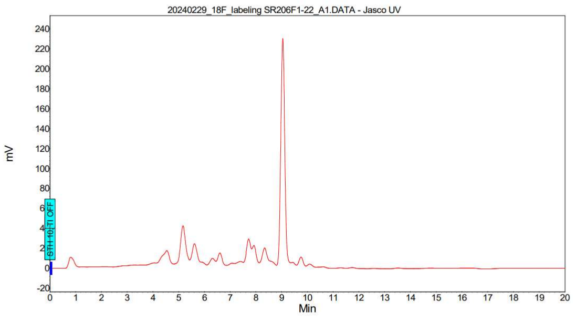

Figure S112. Copy of semi-preparative HPLC chromatograms (System 3;left: signal of gamma-detector; right: UV-signal) obtained for purification of compound **[^{18}F]22b** after labeling of **22a** with [^{18}F]fluoride under optimized conditions. Product was collected between 6.8 and 7.6 min.

	#1	#2	#3	#4	#5	#6	Mean ± SD (n)
RCY [%]	50	54	62				55.3 ± 5.0 (n=3)

Table S13. Detailed results of RCY and Mean ± SD (n) for the radiosynthesis and isolation of **[^{18}F]22b**.

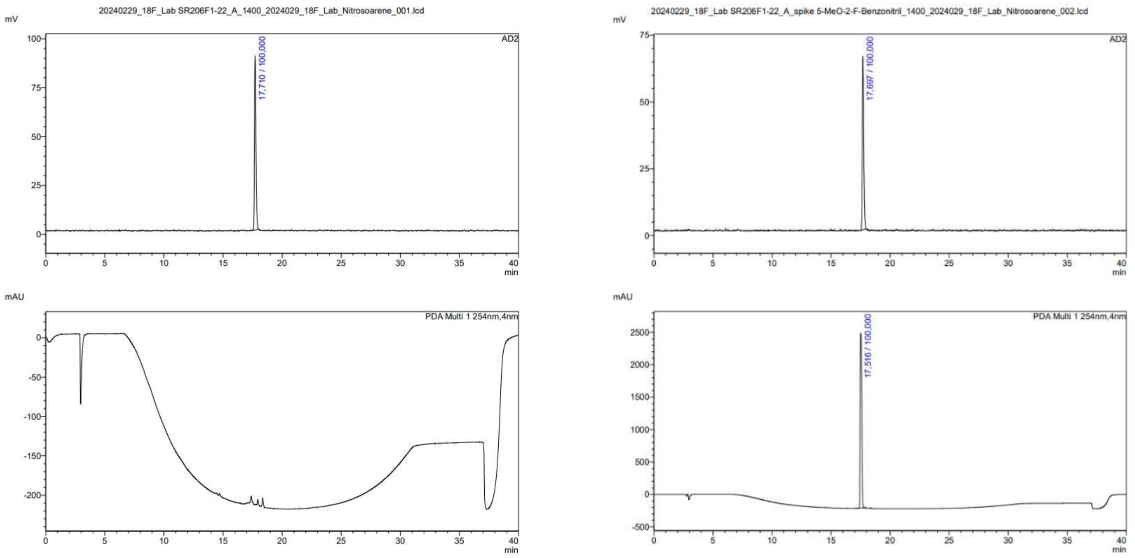

Figure S113. Copy of analytical HPLC chromatograms (System 1; upper panel: signal of gamma-detector; lower panel: UV-signal) obtained for compound **[^{18}F]22b** after semi-preparative purification without (left) and with (right) addition of the authentic non-radioactive reference. In the HPLC setup, the UV detector is in row before the γ-detector with Δt_R of 0.17-0.18 min between both detectors.

Radiolabeling of compound 23a

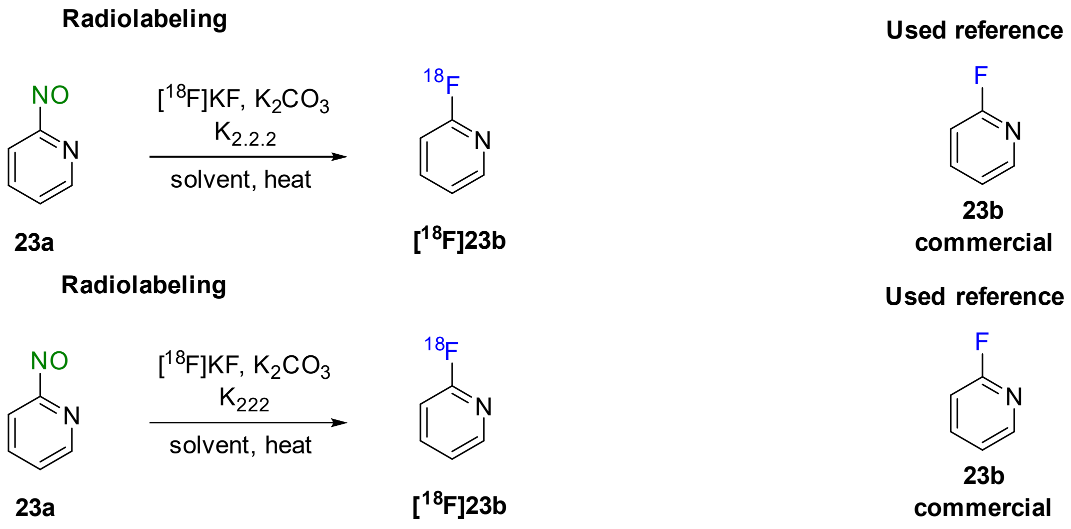

Figure S*114*. Overview for radiolabeling and used references

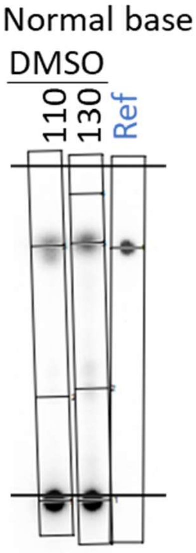

Figure S115. Copy of radio-TLC obtained for ^{18}F-labeling of compound **23a** using using normal base. From left to right: (i) DMSO, 110°C; (ii) DMSO, 130°C; (iii) authentic reference. TLC conditions silica gel; n-hexan/EtOAC 50/50.

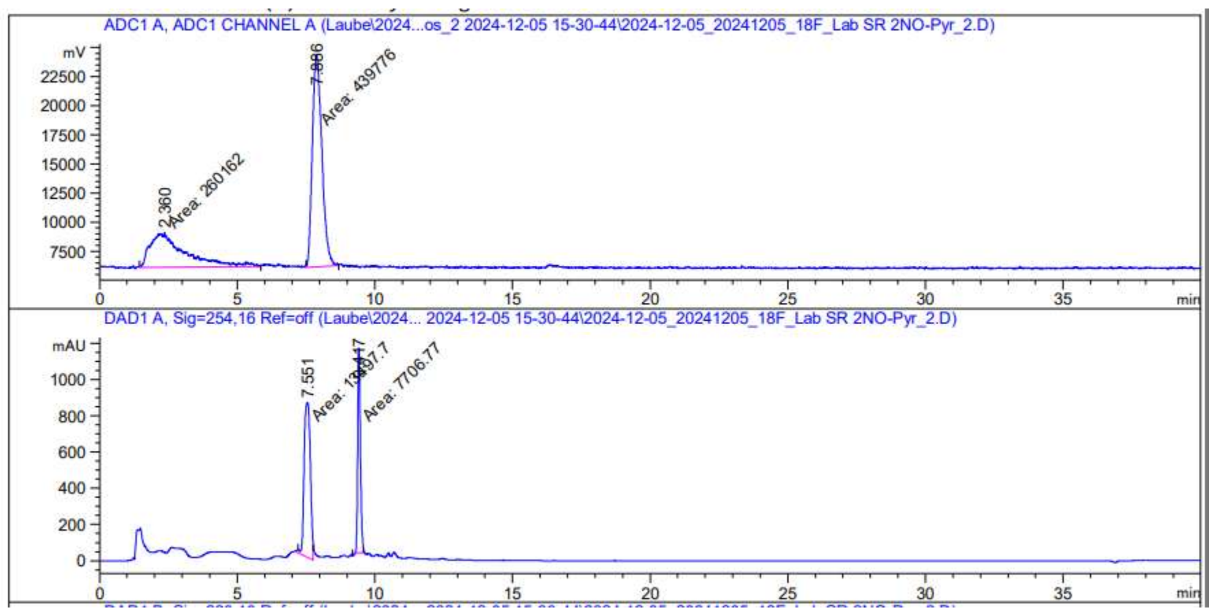

Figure S116. Exemplary analytical radio-HPLC chromatogram (System 2, gradient 4; upper panel: signal of gamma-detector; lower panel: UV-signal) of the crude reaction mixture obtained for ^{18}F-labeling of compound **23a** and reaction with normal base at 130°C in DMSO.

A)				TLC		Set				
			1	2	3	4	5	6	7	8
	2-Nitrosopyridine		MeCN		DMF			DMSO		
			90	110	90	110	130	90	110	130
	Normal	Product	n.d.	n.d.	n.d.	n.d.	n.d.	n.d.	3	10
		Side Products							0	0
	1/4 Base	Product	n.d.	n.d.	n.d.	n.d.	n.d.	n.d.	n.d.	n.d.
		Side Products								

B)				HPLC						
			1	2	3	4	5	6	7	8
	2-Nitrosopyridine		MeCN		DMF			DMSO		
			90	110	90	110	130	90	110	130
	Normal	Product	n.d.	n.d.	n.d.	n.d.	n.d.	n.d.	n.d.	62
		Side Products								0
	1/4 Base	Product	n.d.	n.d.	n.d.	n.d.	n.d.	n.d.	n.d.	n.d.
		Side Products								

Figure S117. Detailed results of optimization experiments for ^{18}F-labeling of compound **23a** obtained by radio-TLC (A) and radio-HPLC (B) analysis. n.d. not determined.

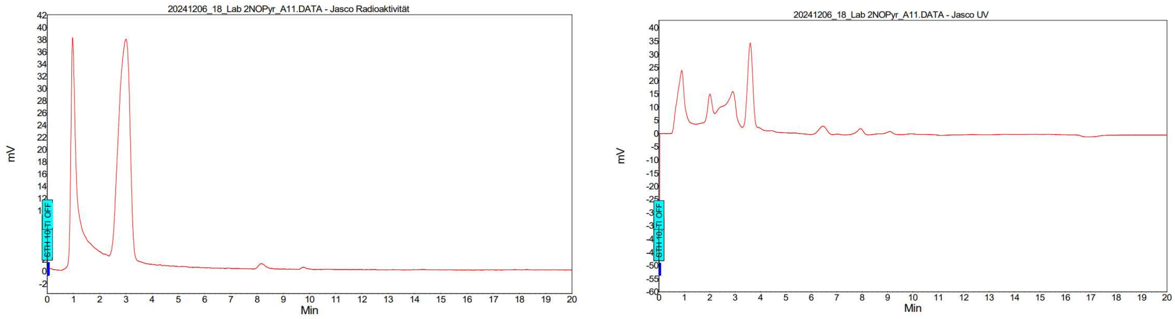

Figure S118. Copy of semi-preparative HPLC chromatograms (System 3; left: signal of gamma-detector; right: UV-signal) obtained for purification of compound **[^{18}F]23b** after labeling of **23a** with [^{18}F]fluoride under optimized conditions. Product was collected between 2.5 and 3.5 min.

	#1	#2	#3	#4	#5	#6	Mean ± SD (n)
RCY [%]	45	46	29	27			36.8 ± 8.8 (n=4)

Table S14. Detailed results of RCY and Mean ± SD (n) for the radiosynthesis and isolation of **[^{18}F]23b**.

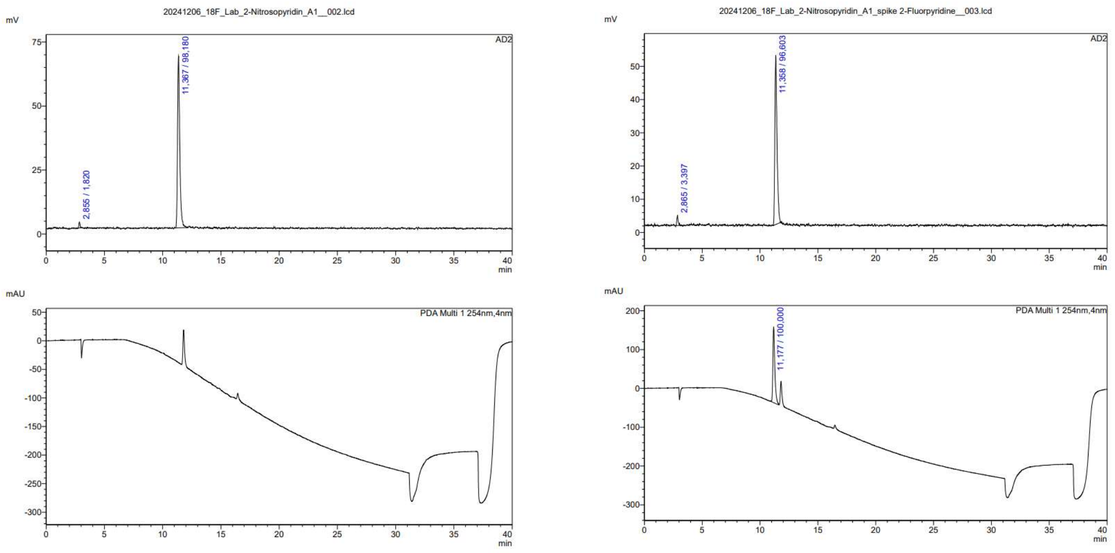

Figure S119. Copy of analytical HPLC chromatograms (System 1; upper panel: signal of gamma-detector; lower panel: UV-signal) obtained for compound **[^{18}F]23b** after semi-preparative purification without (left) and with (right) addition of the authentic non-radioactive reference. In the HPLC setup, the UV detector is in row before the γ-detector with Δt_R of 0.17-0.18 min between both detectors.

Radiolabeling of compound 24a

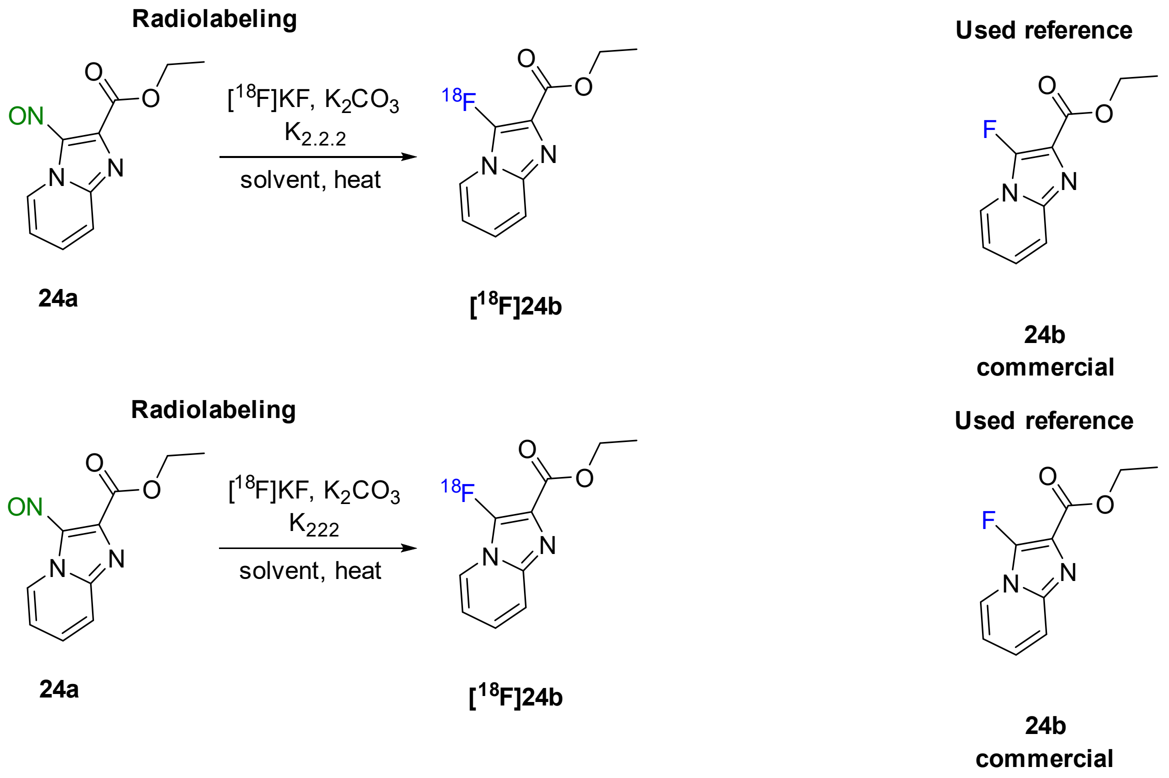

Figure S*120*. Overview for radiolabeling and used references

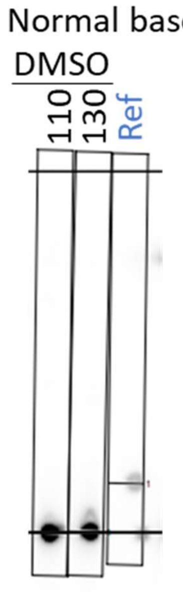

Figure S121. Copy of radio-TLC obtained for ^{18}F-labeling of compound **24a** using normal base. TLC conditions silica gel; 1st elution: n-hexan/EtOAC 50/5, 2nd elution DCM/MeOH 90/10.

A)

				TLC		Set			
		1	2	3	4	5	6	7	8
**SR 307/24**		**MeCN**		**DMF**			**DMSO**		
		90	110	90	110	130	90	110	130
**Normal**	Product	n.d.	n.d.	n.d.	n.d.	n.d.	n.d.	0	0
	Side Products							0	0
**1/4 Base**	Product	n.d.	n.d.	n.d.	n.d.	n.d.	n.d.	n.d.	n.d.
	Side Products								

B)

				HPLC					
		1	2	3	4	5	6	7	8
**SR 307/24**		**MeCN**		**DMF**			**DMSO**		
		90	110	90	110	130	90	110	130
**Normal**	Product	n.d.	n.d.	n.d.	n.d.	n.d.	n.d.	n.d.	n.d.
	Side Products								0
**1/4 Base**	Product	n.d.	n.d.	n.d.	n.d.	n.d.	n.d.	n.d.	n.d.
	Side Products								

Figure S122. Detailed results of optimization experiments for ^{18}F-labeling of compound **24a** obtained by radio-TLC (A) and radio-HPLC (B) analysis. n.d. not determined.

Radiolabeling of compound 25a

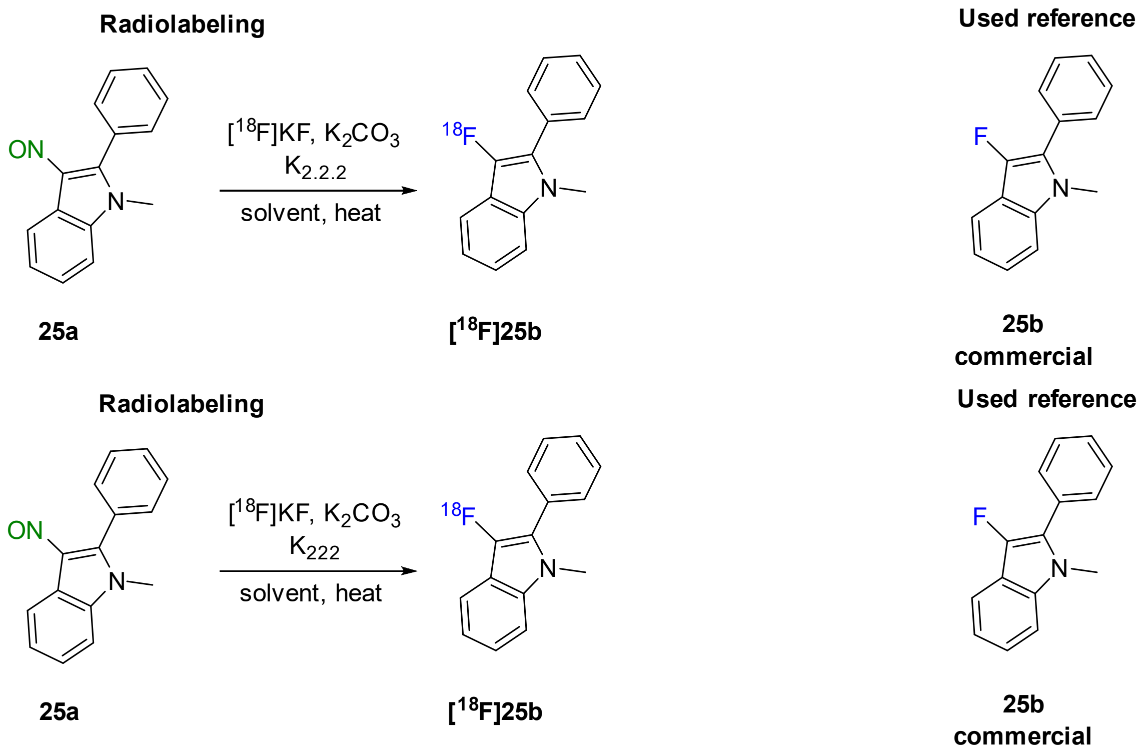

Figure S*123*. Overview for radiolabeling and used references

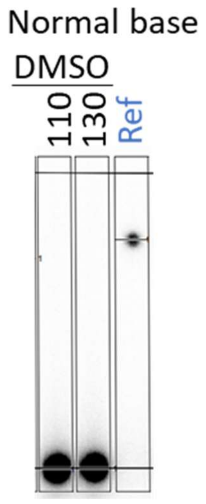

Figure S124. Copy of radio-TLC obtained for ^{18}F-labeling of compound **25a** using normal base. TLC conditions silica gel; n-hexan/EtOAC 50/50.

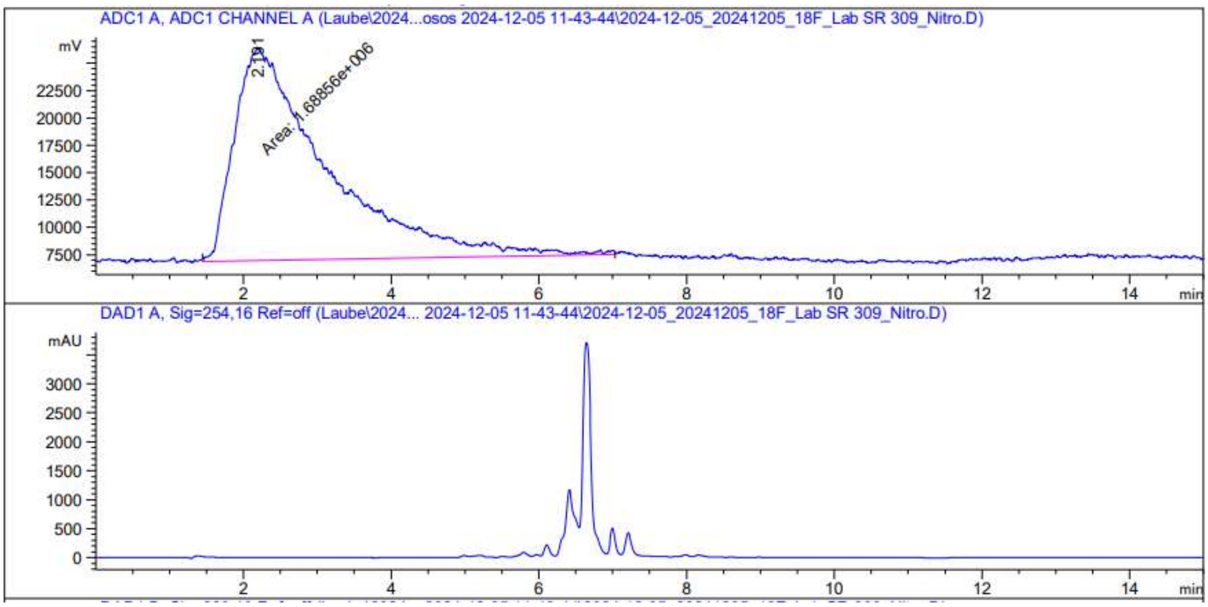

Figure S125. Exemplary analytical radio-HPLC chromatogram (System 2, gradient 3; upper panel: signal of gamma-detector; lower panel: UV-signal) of the crude reaction mixture obtained for ^{18}F-labeling of compound **25a** and reaction with normal base at 130°C in DMSO.

A) TLC Set

		1	2	3	4	5	6	7	8
**SR 309/24**		**MeCN**		**DMF**			**DMSO**		
		90	110	90	110	130	90	110	130
**Normal**	Product	n.d.	n.d.	n.d.	n.d.	n.d.	n.d.	0	0
	Side Products							0	0
**1/4 Base**	Product	n.d.	n.d.	n.d.	n.d.	n.d.	n.d.	n.d.	n.d.
	Side Products								

B) HPLC

		1	2	3	4	5	6	7	8
**SR 309/24**		**MeCN**		**DMF**			**DMSO**		
		90	110	90	110	130	90	110	130
**Normal**	Product	n.d.	n.d.	n.d.	n.d.	n.d.	n.d.	n.d.	0
	Side Products								0
**1/4 Base**	Product	n.d.	n.d.	n.d.	n.d.	n.d.	n.d.	n.d.	n.d.
	Side Products								

Figure S126. Detailed results of optimization experiments for ^{18}F-labeling of compound **25a** obtained by radio-TLC (A) and radio-HPLC (B) analysis. n.d. not determined.

9. Radiosyntheses followed by subsequent HPLC-DAD-γ-HRMS analysis

9.1. Radiosynthesis starting from 4a and functionalization using Girard's reagent T

^{18}F-Fluorination was performed as described in section 7 either no-carrier added (n.c.a.; without further addition of KF) or carrier added (c.a.; with addition of KF (1 µL, 0.5 M, 0.5 µmol, ~0.27 equiv. relative to **4a**) to the 50 µL aliquot of [^{18}F]fluoride containing eluate before evaporation). At the end of radiosynthesis and for functionalization with Girard's reagent T, 20 µL of a 10 mg/mL solution of Girard reagent T in H_2O was added to 180 µL of the collected HPLC fraction containing **[^{18}F]4b** in 0.1% TFA in MeCN/H_2O. The reaction was allowed to stand for at least 10 min at room temperature before analysis. Analyses were performed using system 1 (gradient 3) and radio-HPLC-DAD-γ-HRMS (System 4).

As a result of both n.c.a. and c.a. reactions (Figures S145 and S146), addition of Girard's reagent T resulted in an almost complete conversion of **[^{18}F]4b** (t_R = 12.06 min, System 1, gradient 3) to a more hydrophilic radioactive compound (t_R = 9.04 min, System 1, gradient 3) which is in line with the expected retention time shift for conversion from **[^{18}F]4b** to the product **[^{18}F]4b-hydrazone**. Further, radio-HPLC-DAD-γ-HRMS analysis (System 4) was performed after 24 h (time for complete radioactive decay). For the collected HPLC fraction containing only **[^{18}F]4b** and resulting from the n.c.a and c.a. reactions, no MS signal could be detected of **4b** (m/z calculated for $[M+H]^+$: 124.0423) or **4b-hydrazone** (m/z calculated for $[M+H]^+$: 238.1350) using extracted ion chromatogram (EIC) functionality which filters selected m/z from total ion chromatograms (TIC; data not shown). For reactions without or with KF addition and resulting from conversion to **[^{18}F]4b-hydrazone**, an EIC signal of **4b-hydrazone** (m/z calculated for $[M+H]^+$: 238.1350) could be observed at 4.35-4.40 min and extraction of the MS spectrum at this time point resulted in a clear MS signal of **4b** in the MS spectrum extracted from the TIC.

In conclusion, low ionizability of **4b** hindered detection via non-targeted mass spectrometry in the observed concentration range. Of note, we aimed for detection of **4b** which is present in **[^{18}F]4b** because also for n.c.a. reactions the in-house produced [^{18}F]fluoride contains non-radioactive fluoride from production sources which leads usually to molar activities of 50-100 GBq/µmol for in-house produced radiotracers and is lower compared to the theoretical molar activity of ~63400 GBq/µmol. This means that also in n.c.a reactions the expected molar ratio of **[^{18}F]4b** to **4b** is at around 1:1000. However, after conversion to the constantly positively charged **4b-hydrazone** a clear signal of the formed product was observed in samples resulting from n.c.a. and c.a. radiosyntheses which confirms identification of **[^{18}F]4b** as 4-[^{18}F]fluorobenzaldehyde formed by [^{18}F]fluoro-for-nitroso exchange beside the chromatographic comparison to the non-radioactive reference as performed throughout this work.

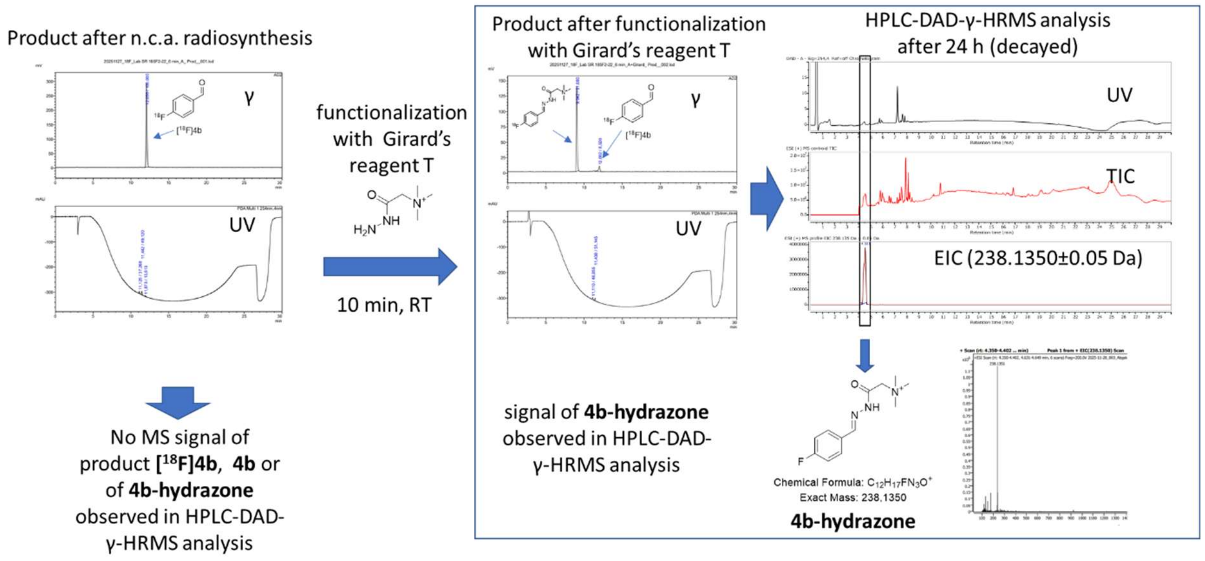

Figure S127. Results obtained for functionalization of **[^{18}F]4b** with Girard's reagent T. HPLC-DAD-γ-HRMS was performed after decay (24h after synthesis), so that no radioactivity signal was observed.

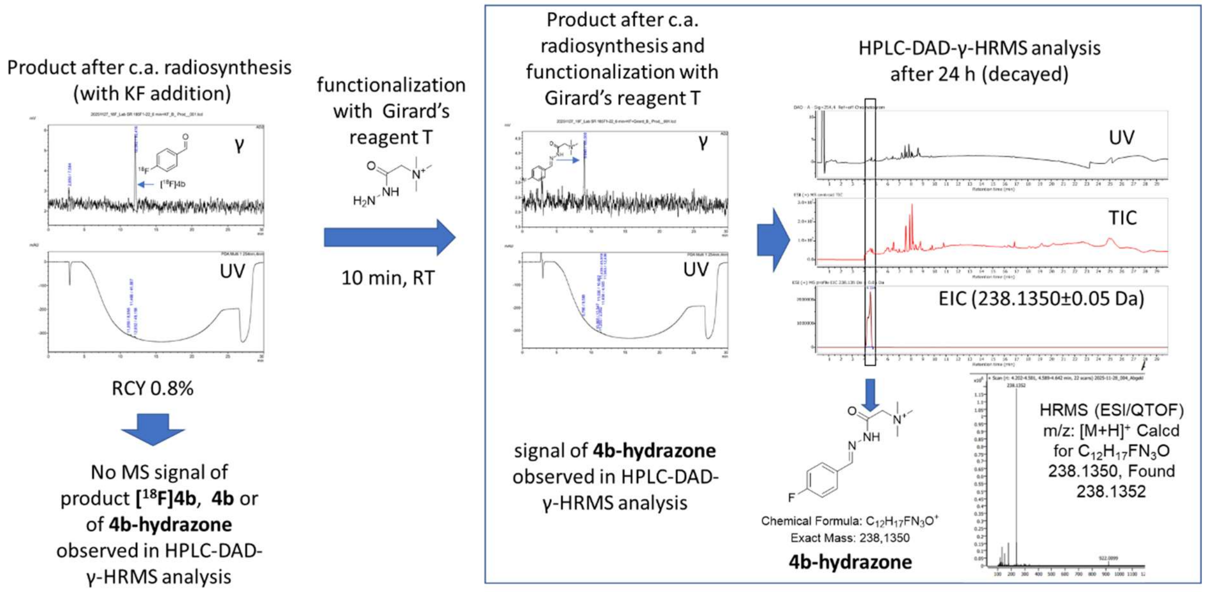

Figure S128. Results obtained for functionalization of **[^{18}F]4b** obtained after c.a. radiosynthesis (with KF addition) and with Girard's reagent T. HPLC-DAD-γ-HRMS was performed after decay (24h after synthesis), so that no radioactivity signal was observed.

9.2. Radiosynthesis starting from 6a

^{18}F-Fluorination was performed as described in section 7 either no-carrier added (n.c.a.; without further addition of KF) or carrier added (c.a.; with addition of KF (1 µL, 0.5 M, 0.5 µmol, ~0.42 equiv. relative to **6a**) to the 50 µL aliquot of [^{18}F]fluoride containing eluate before evaporation). Analyses were performed using system 1 (gradient 3) or radio-HPLC-DAD-γ-HRMS (System 4) and the purified products in HPLC eluent.

Both products obtained from n.c.a. and c.a. reactions (Figures S147), showed the same retention time for **[^{18}F]6b** in radio-HPLC (t_R = 10.92 min, System 1, gradient 3) while RCY for the c.a. reaction was found to be lower. Further, radio-HPLC-DAD-γ-HRMS analysis (System 4) was performed using directly the radioactive sample. For both reactions, the same retention time for **[^{18}F]6b** (t_R = 13.41 min, System 4) was observed. For **[^{18}F]6b** resulting from the n.c.a reaction, no MS signal could be detected of **6b** (m/z calculated for $[M+H]^+$: 201.0710; data not shown). However, for the product obtained from the c.a. reaction the signal of **6b** (m/z calculated for $[M+H]^+$: 201.0710) could be clearly detected using extracted ion chromatogram (EIC) functionality which filters selected m/z from total ion chromatograms (TIC). The EIC signal could be observed at 13.28-13.53 min and extraction of the MS spectrum from the TIC at this time point resulted in a clear MS signal of **6b** beside other yet unidentified MS-signals in the MS spectrum. The UV-signal (13.35 min), the radioactivity signal (14.42 min) and HRMS signal (13.43 min) corresponded to the expected retention time shift observed in this chromatographic system.

As discussed above, we aimed for detection of **6b** which is present in the product of n.c.a reaction due to usually observed lower molar activity compared to theoretical carrier-free condition and hence higher presence of **6b** compared to **[^{18}F]6b**. In conclusion, low ionizability of **6b** only allowed detection via non-targeted mass spectrometry for the product obtained from c.a. reactions and hence further lowered molar activity as well as increased amount of **6b** in relation to **[^{18}F]6b**. This however further confirms identification of **[^{18}F]6b** as 4-[^{18}F]fluorobenzophenone formed by [^{18}F]fluoro-for-nitroso exchange beside the chromatographic comparison to the non-radioactive reference as performed throughout this work.

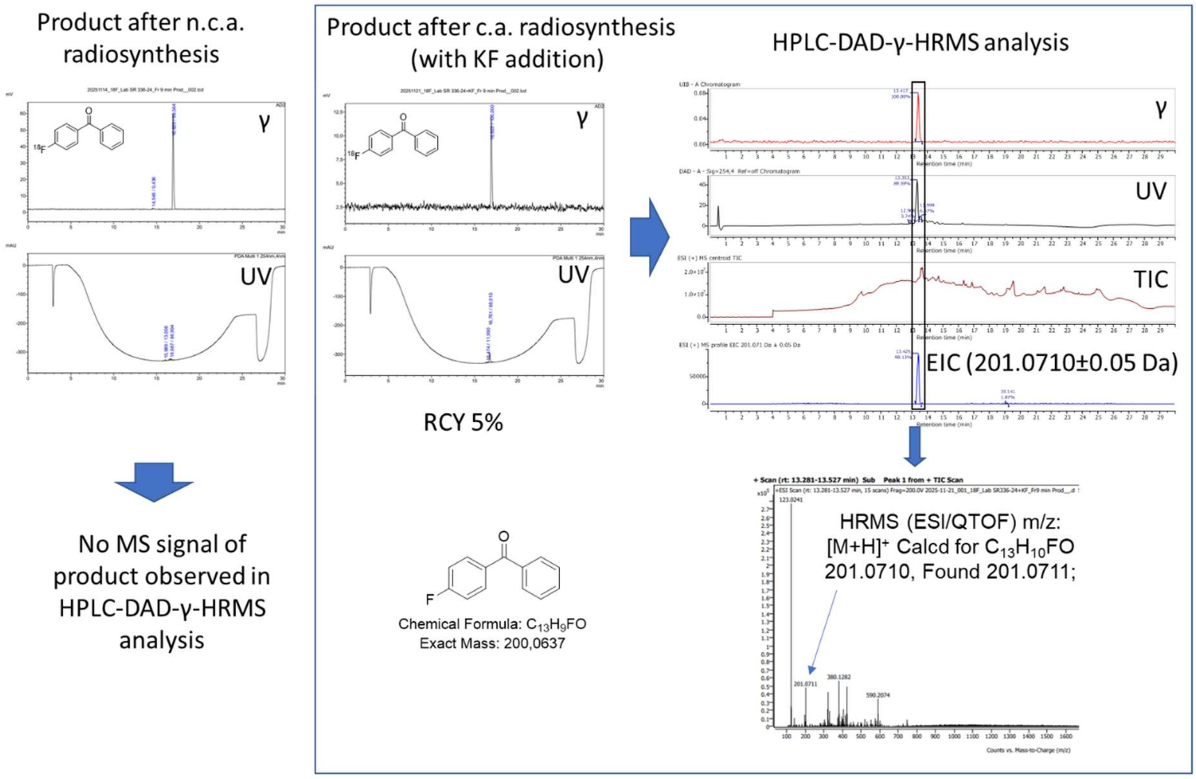

Figure S129. Results obtained for **[^{18}F]6b** obtained after radiosynthesis without and with KF addition. HPLC-DAD-γ-HRMS was performed using the radioactive sample.

10. Radiosynthesis followed by subsequent NMR analysis

10.1. Radiosynthesis starting from 9a for product identification using ^{19}F-NMR

^{18}F-Fluorination was performed as described in section 6 with addition of aqueous KF (carrier added , c.a.; 2 µL, 0.5 M, 1 µmol, ~0.24 equiv. relative to **9a**) to a 100 µL aliquot of [^{18}F]fluoride (~22 MBq) containing HYFE ¼ eluate before evaporation. The reaction was performed using **9a** (100 µL, 5 mg/mL in DMF) at 130°C for 15 min. At the end of the reaction, the mixture was diluted with 200 µL D_2O and 300 µL MeCN. Radio-TLC was performed (siliga gel, n-hexan/EtOAc 1/1). The HPLC vial was placed in a lead container and stored for 2 days in a freezer to wait for decay. ^{19}F-NMR was recorded on a Bruker Avance III 400 MHz/Agilent DD2-400 MHz (^{19}F: 376 MHz) with 1 s relaxation time and 2500 scans to determine conversion of fluoride into 4-fluorobenzonitrile. After that, sequentially trifluoroacetic acid and 4-fluorobenzonitrile was added for reference and identification, respectively, and ^{19}F-NMR spectra were recorded. The evaluation of the NMR spectra was carried out using the program Mestrelab MestReNova (version 15.0.1).

For carrier-added radiosynthesis using **9a** a radiochemical conversion of 24.2% was determined by radio-TLC at the day of synthesis. After 2 days waiting for the complete decay of ^{18}F, the ^{19}F-NMR showed two signals (Figure S149, left) which were assigned to fluoride (123.45 ppm) and 4-fluorobenzonitrile (104.9 ppm) in a ratio of 3.47 to 1.00 corresponding to 22.4% conversion of fluoride to 4-fluorobenzonitrile. Of note, peaks were referenced to the signal of added trifluoroacetic acid (1 µmol, set to 76.5 ppm, Figure S149, middle). Further, product identification was confirmed by subsequent addition of 4-fluorobenzonitril (1 µmol, Figure S149, right) which increased the signal intensity but gave no second signal.

In conclusion, this could verify the formation of 4-fluorobenzonitrile by [^{18}F]fluoro-for-nitroso exchange starting from 4-nitrosobenzonitrile (**9a**) at carrier added fluoride concentration level beside the chromatographic comparison to the non-radioactive reference as performed throughout this work.

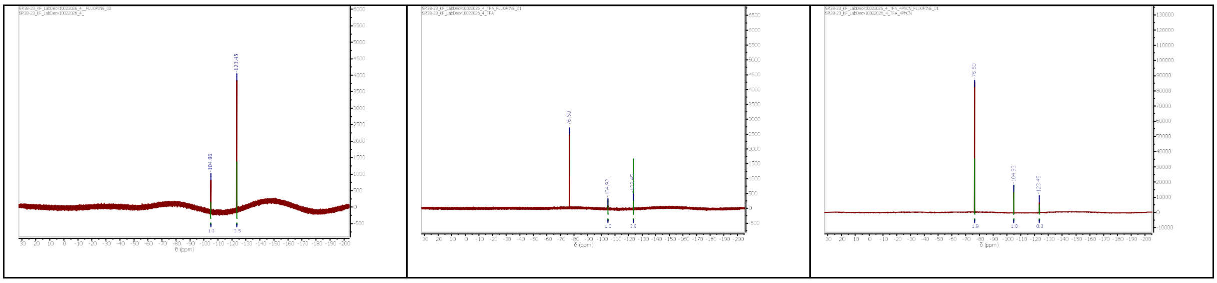

Figure S130. Results of ^{19}F-NMR analysis after carrier added labeling of **9a** and decay (middle), addition of trifluoroacetic acid as reference (middle), and addition of 4-fluorobenzonitrile for product identification (left).

10.2. Comparison of carrier-added (radio)synthesis starting from 9a with or without QMA elution using ^{19}F-NMR and radio-TLC

As a preceeding of this experiment, KF (1 µL, 0.5 M, 0.5 µmol) was added to HYFE ¼ (50 µL), the reaction was dried, reacted with **9a** (5 mg/mL in DMF, 50 µL) at 130° for 15 min, quenched, and analyzed analogously to the described procedure in section 10.1. This experiments showed considerably lower conversion of fluoride into 4-fluorobenzonitrile compared to the radioactive experiment, i.e. only 3 % (Table S15, entry #1) compared to 22.4% determined by NMR in the radiosynthesis experiment (see section 10.1).

The following experiment was performed to investigate if the either the elution of HYFE ¼ through the QMA cartridge or the use of ^{18}F in the mixture had impact on the conversion of (radio)fluoride to the product **[^{18}F]9b** (/**9b**). For that, approx. 3 mL HYFE ¼ solution was prepared which was used for all subsequent experiments. Further, a solution of **9a** (5 mg/mL in DMF) was prepared to use for all subsequent experiment. Two Sep-Pak AccellPlus QMA Carbonate Plus Light Cartridge cartridges were equilibrated in the same manner as given in section 6. At first part, one QMA was washed with 1 mL dry MeCN and then 1 mL HYFE ¼ was passed through the cartridge and the eluate was used for further experiments (denoted as HYFE ¼ 'inactive'). Accordingly to section 6, the second QMA was loaded with [^{18}F]fluoride, washed with 1 mL MeCN (dry) and eluted with HYFE ¼ to give [^{18}F]fluoride/HYFE 1/4. In parallel and for each reaction, KF (2 µL, 0.5 M, 1 µmol) was added to 100 µL aliquots of HYFE ¼ 'inactive' (100 µL) and [^{18}F]fluoride/HYFE 1/4, respectively. According to the general procedure in section 6, the solutions were dried in parallel (5 min, 90°C, He), the precursor **9a** (100 µL 5 mg/mL in DMF) was added, the mixtures were heated at 130°C for 15 min, and the reactions were quenched after cooling with 200 µL D_2O and 300 µL MeCN. Reactions from HYFE ¼ 'inactive' were immediately (within one day, intermediate storage in the fridge) analyzed by ^{19}F-NMR as described in 9.1 to analyze for chemical conversion (Table S15, entry #2-4). Reactions resulting from [^{18}F]fluoride/HYFE ¼ were analyzed by radio-TLC (n-hexane/EtOAc 1/1) for radiochemical conversion and by radio-HPLC to confirm identity (only Table S15, entry #5), and analyzed by ^{19}F-NMR after 2 days waiting for decay as described in section 10.1 to analyze for chemical conversion (Table S15, entry #5-7).

As a second set of experiment that was performed in parallel, KF (2 µL, 0.5 M, 1 µmol) was added to 100 µL aliquots of the HYFE ¼ solution which was not used for QMA elution and the HYFE ¼ solution that was passed through the QMA (HYFE ¼ 'inactive') and further treated and reacted with **9a** according to the procedure described above for HYFE ¼ 'inactive'. Reactions were immediately analyzed by ^{19}F-NMR as described in 9.1 to analyze for chemical conversion (Table S15, entry #8-9).

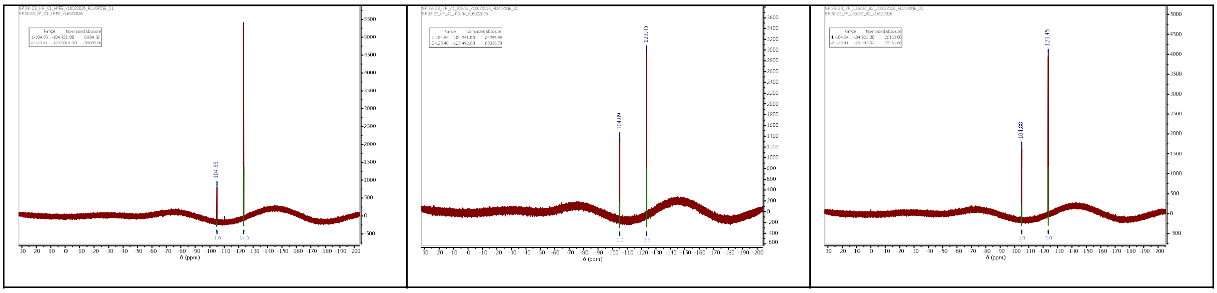

Figure S131. Exemplary ^{19}F-NMR spectra obtained from (carrier-added radio)fluorination of **9a** using HYFE ¼ (left, result of Table S15, entry #9), HYFE ¼ 'inactive' (middle, result of Table S15, entry #2), and [^{18}F]fluoride/HYFE ¼ after decay (right, results of Table S15, entry #6).

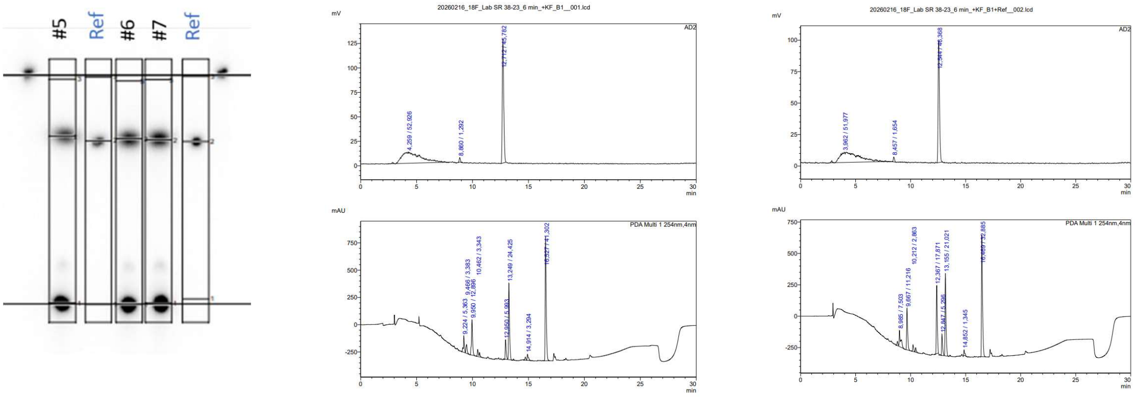

Figure S132. Copy of radio-TLC (left) obtained for carrier-added ^{18}F-labeling of compound **9a** (compare Table S15, entries #5-7) and copy of exemplary radio-HPLC chromatograms of the crude reaction mixture (compare Table S15, entries #5) without (middle) and with addition (right) of the non-radioactive authentic reference **9b** (System 1; gradient 3; upper panel: signal of gamma-detector; lower panel: UV-signal at 220 nm). In the HPLC setup, the UV detector is in row before the γ-detector with Δt_R of 0.17-0.18 min between both detectors.

As a result, using HYFE ¼ directly provided low conversion of 3% and 7% observed by ^{19}F-NMR (Table S15, entry #1 & 8). In a direct comparison using the same precursor and reaction conditions(Table S15, entry #8-9), the use of HYFE ¼ 'inactive' provided expected conversion of 20% what showed that the pass through the QMA had an impact on conversion in this case. The in parallel comparison of HYFE ¼ 'inactive' and [^{18}F]fluoride/HYFE ¼ (after ^{18}F-decay) provided very comparable conversion results of 27.0 ± 1.4 (n=3) and 27.0 ± 1.5 (n=3), which was in the latter case also very consistent with the results obtained directly by radio-TLC showing RCC of 26.4 ± 1.3 (n=3). Hence, the presence of [^{18}F]fluoride and hence ionizing radiation in the carrier-added reactions had no further impact on (radio)chemical conversion of the fluoro-for-nitroso exchange.

In conclusion, the lower conversion observed with the HYFE ¼ solution before the passage through the QMA cartridge indicates a yet unraveled influence of this process. The QMA cartridge is in beforehand equilibrated with a sequence of water, 1 M $NaHCO_3$ and water which indicates a potential influence of $NaHCO_3$ in this reaction which however has to be investigated in future experiments taking the K_2CO_3 introduced by HYFE ¼ or HYFE normal into account.

Table S15. (Radio)chemical conversion determined by ^{19}F-NMR or radio-TLC for (carrier-added radio)fluorination of **9a**. Reactions of entry #2-7 and #8-9, respectively, were performed in parallel as discussed above.

	% Conversion of fluoride to **9b** analyzed by ^{19}F-NMR			% Conversion of [^{18}F]fluoride to **[^{18}F]9b** analyzed by radio-TLC
#	HYFE ¼	HYFE ¼ 'inactive'	[^{18}F]Fluoride/HYFE ¼	[^{18}F]Fluoride/HYFE ¼
1	3.1			
2		27.7		
3		28.3		
4		25.1		
5			28.3	24.6
6			24.9	27.5
7			27.8	27.0
8	6.5			
9		20.4		

Table S16. Mean ± SD (n) for the conversion determined by ^{19}F-NMR for entries 2-4 of Table S15

	#1	#2	#3	#4	#5		#6	Mean ± SD (n)
[%]	27,7	28,3	25,1					27.0 ± 1.4 (n=3)

Table S17. Mean ± SD (n) for the conversion determined by ^{19}F-NMR for entries 5-8 of Table S15

	#1	#2	#3	#4	#5		#6	Mean ± SD (n)
[%]	28,3	24,9	27,8					27.0 ± 1.5 (n=3)

Table S18. Mean ± SD (n) for the conversion determined by radio-TLC for entries 5-8 of Table S15

	#1	#2	#3	#4	#5		#6	Mean ± SD (n)
RCC [%]	24,6	27,5	27					26.4 ± 1.3 (n=3)

11. Analysis of RCC(TLC) at different precursor concentrations for selected substrates

^{18}F-Fluorinations in this work were all performed at a precursor concentration of 5 mg/mL based on previous experiences in concentration dependence for radiofluorinations and practical issues. The resulting precursor concentrations were hence between ~20 mM (25a) and ~45 mM (23a) but most often at around 30-35 mM. For selected precursors **6a**, **9a** and **22a**, a more detailed analysis of dependence of RCC (TLC) on precursor concentration was performed. For this, ^{18}F-Fluorination was performed as described in section 6 using **6a**, **9a** and **22a** at a concentration of 5, 10, 20, 30, and 40 mM, respectively in the given solvent (Figure S153) at 130°C for 15 min. At the end of the reaction, the mixture was diluted with 200 µL water/MeCN 1/1 and analyzed by radio-TLC (silica gel, n-hexan/EtOAc 1/1).

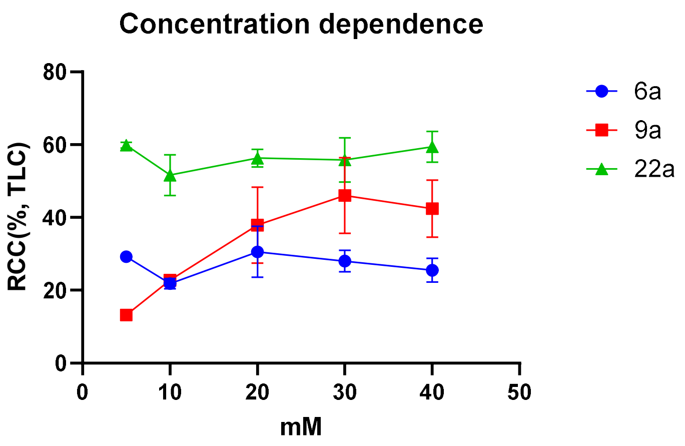

Figure S*133*. Concentration dependence of RCC (TLC) for ^{18}F-fluorination of compounds **6a**, **9a** and **22a** given as mean ± SD (n=2).

			1	2	3	4	5	6	7	8	7	8
6a	SR336/24		40 mM		30 mM		20 mM		10 mM		5 mM	
N, DMSO 130°C		Product	27,8	23,2	25,9	30,1	25,6	35,5	22,8	20,8	28,9	29,6

			1	2	3	4	5	6	7	8	7	8
9a	SR38/23		40 mM		30 mM		20 mM		10 mM		5 mM	
1/4, DMF 130°C		Product	48	36,9	53,4	38,7	45,3	30,5	23,2	22,4	13,1	13,4

			1	2	3	4	5	6	7	8	7	8
22a	SR206F1		40 mM		30 mM		20 mM		10 mM		5 mM	
N, DMF 130°C		Product	62,4	56,4	51,5	60,1	58	54,6	55,6	47,7	59,2	60,4

Figure S*134*. Detailed results of concentration dependence of RCC (TLC) for ^{18}F-fluorination of compounds **6a**, **9a** and **22a**

^{18}F-fluorination using **6a** and **22a**, respectively, provided comparable RCC in the concentration range of 5-40 mM. For **9a** comparable RCC were observed in the concentration range of 20-40 mM while ^{18}F-

fluorination furnished markedly lower RCC at lower concentrations of 5 and 10 mM (Figure S152 & S153).

These results indicate that for [^{18}F]fluoro-for-nitroso exchange the RCC is stable in concentration ranges of 20-40 mM and also ^{18}F-fluorination can proceed at lower concentrations with different efficacy. However, this is in general substrate dependent and should be optimized for each specific precursor molecule.

12. Analysis of different phase transfer agents for selected substrates

^{18}F-Fluorinations in this work were all performed using $K_{2.2.2}/K_2CO_3$ as phase transfer catalyst and base. Other PTCs were not generally applied. To investigate if in principal also other phase transfer catalysts can be applied, both tetraethylammonium bicarbonate (TEAB) and tetrabutylammonium tosylate (TBAOTs) were tested for the ^{18}F-fluorination using **5a**, **9a**, **22a** and **25a**.

For this, ^{18}F-Fluorination was performed as described in section 6 but with the following difference: [^{18}F]fluoride (200-300 MBq) was adsorbed on the QMA light, washed with 1 mL of EtOH, and eluted using a either a solution of TEAB or TBAOTs (4 mg/mL in EtOH). Elution efficiencies in both cases exceeds 95%. Further processing was performed as described and all reactions were performed at a precursor concentration of 40 mM in DMF at 130°C for 15 min. . At the end of the reaction, the mixture was diluted with 200 µL water/MeCN 1/1 and analyzed by radio-TLC (silica gel, n-hexan/EtOAc 1/1).

5a	SR177F1/22		TEAB		TBAOTs	
**DMF**		Product	0	0	0	0
130°C						

9a	SR38/23		TEAB		TBAOTs	
**DMF**		Product	24,7		0	
130°C						

22a	SR206F1		TEAB		TBAOTs	
**DMF**		Product	45,3	49,1	0	0
130°C						

25a	SR309/24		TEAB		TBAOTs	
**DMF**		Product	0,4	0	0,4	0
130°C						

Figure S*135*. Detailed results of RCC (TLC) for ^{18}F-fluorination of compounds **5a**, **9a, 22a** and **25a** using TEAB or TBAOTs as phase transfer catalysts.

For all tested precursors, no [^{18}F]fluoro-for-nitroso exchange was observed with TBAOTs. In comparison, ^{18}F-fluorination proceeded effectively for **9a** and **25a** using TEAB, although with slightly lower RCC compared to the optimal conditions using $K_{2.2.2}/K_2CO_3$. Both, **5a** and **25a** showed no reactivity using TEAB which is in line with the very low respectively no radiochemical conversion obtained using $K_{2.2.2}/K_2CO_3$. This indicates, that a base is necessary for effective [^{18}F]fluoro-for-nitroso exchange.
